# Supplementary material for: Electrochemical aromatic C–H hydroxylation in continuous flow
Source: Nat Commun. 2022 Jul 8;13:3945. doi: 10.1038/s41467-022-31634-4 (PMC9270493; doi:10.1038/s41467-022-31634-4)
Supplement: Supplementary file 1 — Supplementary Information [file 41467_2022_31634_MOESM1_ESM.pdf]

Supplementary Information

Electrochemical Aromatic C–H Hydroxylation in Continuous Flow

Hao Long,[1,2] Tian-Sheng Chen,[1] Jinshuai Song,[3] Shaobin Zhu[4] and Hai-Chao Xu[1,2]

[1]State Key Laboratory of Physical Chemistry of Solid Surfaces, College of Chemistry and Chemical Engineering, Xiamen University, Xiamen 361005, China

[2]Key Laboratory of Chemical Biology of Fujian Province, Xiamen University, Xiamen 361005, China

[3]Green Catalysis Center, College of Chemistry, Zhengzhou University, Zhengzhou 450001, China

[4]NanoFCM INC., Xiamen Pioneering Park for Overseas Chinese Scholars, Xiamen 361006, P. R. China

Correspondence: haichao.xu@xmu.edu.cn

Contents

Supplementary Methods 2

1. **General considerations** 2
2. **Additional optimization of reaction conditions** 3
3. **Detailed steps for the continuous flow electrolysis** 5
4. **Characterization data for the electrolysis products** 7
5. **Synthesis and characterization of new substrates.** 29
6. **Limitations** 33
7. **Mechanistic studies** 34
8. **Computational studies** 40
9. **NMR spectra** 42

Supplementary References 155

Supplementary Methods

1. General considerations

The commercially available reagents were used without purification. Acetonitrile (99.9%, extra dry, with molecular sieves, $H_2O \leq 50$ ppm, resealable bottle), *tert*-butyl methyl ether (99.9%, extra dry, with molecular sieves, $H_2O \leq 50$ ppm, resealable bottle), trifluoroacetic acid and 2,6-lutidine were purchased from Energy Chemical. Flash column chromatography was performed with silica gel (200–300 mesh). NMR spectra were recorded on Bruker AV-400, Bruker AV-500 and Bruker AV-600 instruments. Data were reported as chemical shifts in ppm relative to $CDCl_3$ (7.27 ppm) for ^1H and $CDCl_3$ (77.2 ppm) for ^{13}C. The abbreviations used for explaining the multiplicities were as follows: s = singlet, d = doublet, t = triplet, q = quartet, m = multiplet. Infrared spectra were recorded on a Nicolet AVATER FTIR 330 spectrometer. High-resolution mass spectra (ESI) were recorded by the instrumentation center of Department of Chemistry, Xiamen University, on a Micromass QTOF2 Quadruple/Time-of-Flight Tandem mass spectrometer.

2. Additional optimization of reaction conditions

Supplementary Table 1. More optimization of conditions for C–H hydroxylation.[a]

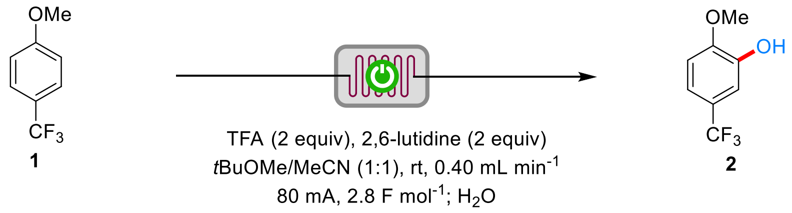

Entry	Deviation from standard conditions	Yield of **2** (%)[b]
1	None	83 (9), 77[c]
2	65 mA	73 (13)
3	70 mA	72 (10)
4	75 mA	72 (10)
5	85 mA	83 (5)
6	0.20 mL min^{-1}	58 (20)
7	0.30 mL min^{-1}	79 (6)
8	0.50 mL min^{-1}	64 (19)
9	Ni cathode	36 (21)
10	Stainless steel cathode	32 (25)
11	TFA (3 equiv) + 2,6-lutidine (2 equiv)	65 (25)
12	TFA (2 equiv) + 2,6-lutidine (3 equiv)	70 (30)
13	THF as solvent	4 (51)
14	MeCN/THF (1:1) as solvent	8 (58)

[a]Reaction conditions: graphite anode, Pt cathode, fluorinated ethylene propylene (FEP) foil spacer (0.15 mm thickness). **1** (0.045 M), 2.0 mL of the outlet solution was collected and analyzed. [b]Yield determined by ^{1}H-NMR analysis using 1,3,5-Trimethoxybenzene as the internal standard. Unreacted **1** was shown in brackets. [c]Isolated yield.

Pt cathode is more efficient than Ni and stainless steel because of its relatively low overptential for H_2 evolution.

Supplementary Table 2. Optimization of reaction conditions[a]

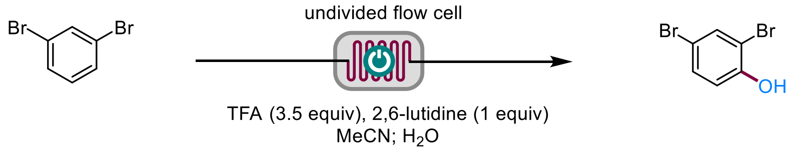

Entry	Deviation from standard conditions	Yield of (%)[b]
1	None	72 (11), 70[c]
2	Conditions of Table S1, entry 1	0 (88)
3	Graphite anode	0 (82)
4	*i*Pr_2NEt instead of 2,6-lutidine	69 (8)
5	0.40 mL min^{-1} instead of 0.3 mL min^{-1}	70 (12)
6	Contrentration = 0.045 M instead of 0.06 M	70 (13)
7	TFA (3.5 equiv) + 2,6-lutidine (2.0 equiv)	66 (19)
8	TFA (3.5 equiv) + 2,6-lutidine (3.5 equiv)	10 (81)

[a]Reaction conditions: graphite anode, Pt cathode, fluorinated ethylene propylene (FEP) foil spacer (0.15 mm thickness). **1** (0.060 M), flow rate = 0.30 mL min^{-1}, I = 64 mA, 2.2 F mol^{-1}. 2.0 mL of the outlet solution was collected. [b]Yield determined by ^{1}H-NMR analysis using 1,3,5-Trimethoxybenzene as the internal standard. [c]Isolated yield.

3. Detailed steps for the continuous flow electrolysis

General procedure A for C–H hydroxylation of arenes in figure 2. The electrolysis was conducted under air for convenience. The reactions employed a flow electrolytic cell equipped with a graphite anode and a Pt cathode with exposed surface area of 10 cm^2 and interelectrode distance of 150 μm (Supplementary Figure 1). The solution containing arene substrate (0.045 M), TFA (2.0 equiv), 2,6-lutidine (2.0 equiv) in dry MeCN/*t*BuOMe (1:1) was pushed using a syringe pump to pass through the flow cell operated with a flow rate of 0.40 mL min^{-1} and a constant current in the range of 52–98 mA. Note that a quick screening of the constant current was performed for each substrate to ensure a maximum yield. The outlet solution was collected for 20 min (8 mL) and quenched with saturated $NaHCO_3$ and extracted with ethyl acetate. To remove 2,6-lutidine, the organic extracts were treated with 2 N HCl, extracted with ethyl acetate, and concentrated under reduced pressure. The residue was chromatographed through silica gel eluting with ethyl acetate/hexanes or methanol/dichloromethane to give the product.

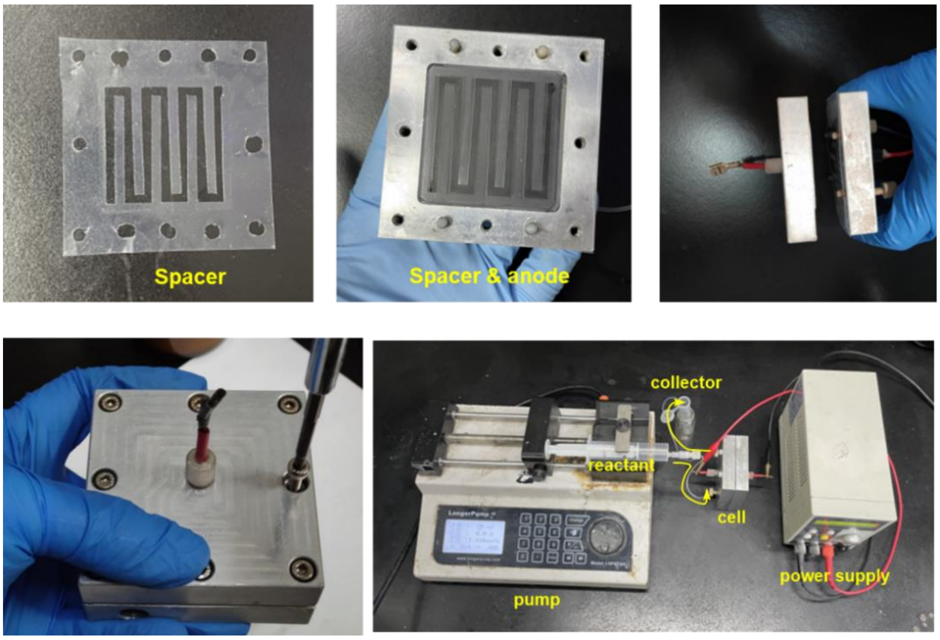

Supplementary Fig. 1. Assembling of the flow reactor and setup.

General procedure B for C–H hydroxylation of electron-deficlent arenes in figure 3. The electrolysis was conducted using a flow electrolytic cell equipped with a Pt anode and a Pt cathode with exposed surface area of 10 cm^2 and interelectrode distance of 150 μm (Supplementary Figure 2). The solution containing arene substrate (0.06 M), TFA (1.26 mmol, 3.5 equiv), 2,6-lutidine (0.36 mmol, 1.0 equiv) in dry MeCN was pushed using a syringe pump to pass through the flow electrolytic cell operated with a flow rate of 0.30 mL min^{-1} and a constant current (61–173 mA). The outlet solution was collected for 20 min (6 mL). The workup procedure was the same as described above.

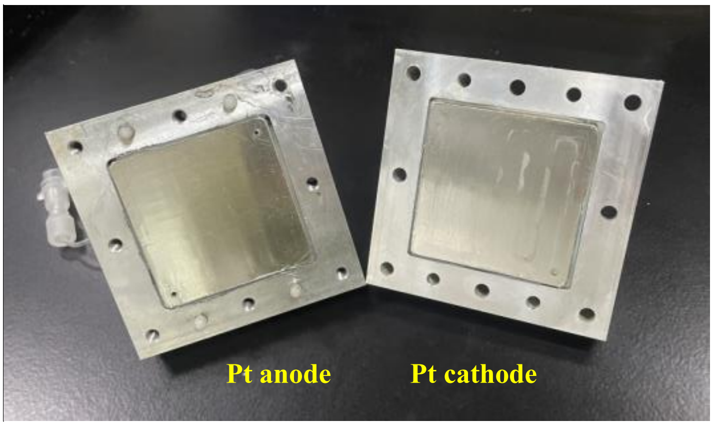

Supplementary Fig. 2. Exploded view of the flow reactor with a Pt anode and a Pt cathode.

Procedure for large scale synthesis of 2: Compound **1** (253 g, 1.44 mol, 1.0 equiv), TFA (2.88 mol, 2.0 equiv), 2,6-lutidine (2.88 mol, 2.0 equiv) were dissolved in MeCN (14.4 L). The resulting solution was pumped through twenty flow cells with a flow rate of 0.5 mL min^{-1} for each reactor (Supplementary Figure 3). The constant current for each reactor was 200 mA. Unpon complete passing of the reactant solution in 24 h, the collected outlet solution was concentrated under reduced pressure on a rotary evaporator, treated with saturated $NaHCO_3$, and extracted with ethyl acetate. The organic extraction was treated with 2 N HCl and extracted with ethyl acetate (to remove 2,6-lutidine). The organic solution was concentrated under reduced pressure on a rotary evaporator. The residue was divided to 8 parts of 20–30 grams and each part was chromatographed through silica gel eluting with ethyl acetate/hexanes. The product **2** was obtained as a yellow oil (204 g, 1.06 mol, yield = 74%).

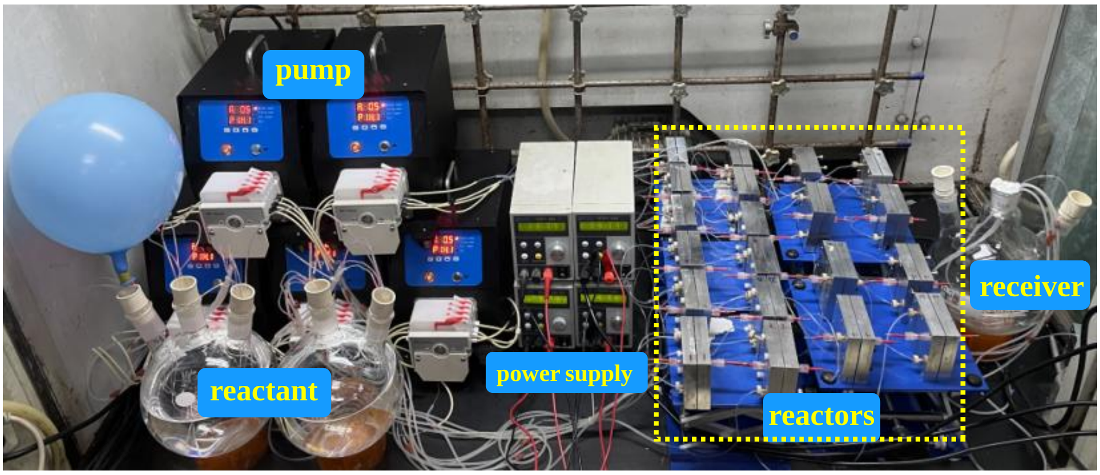

Supplementary Fig. 3. Reaction setup for scale-up with twenty parrell reactors.

4. Characterization data for the electrolysis products

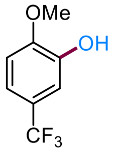

2-Methoxy-5-(trifluoromethyl)phenol (**2**). The title compound was obtained by following the **General Procedure A** and known in the literature.[1] Pale yellow oil, yield = 77% (53 mg), current = 81 mA, electricity = 2.8 F mol^{-1}. ^{1}H NMR (500 MHz, $CDCl_3$) δ 7.22 – 7.17 (m, 1H), 7.17 – 7.13 (m, 1H), 6.90 (d, J = 8.3 Hz, 1H), 5.80 (s, 1H), 3.94 (s, 3H). ^{13}C NMR (126 MHz, $CDCl_3$) δ 149.2, 145.9, 124.4 (q, $J_{C\text{-}F}$ = 271.2 Hz), 123.8 (q, $J_{C\text{-}F}$ = 32.8 Hz), 117.8 (q, $J_{C\text{-}F}$ = 4.3 Hz), 111.8 (q, $J_{C\text{-}F}$ = 3.7 Hz), 110.3, 56.2. ^{19}F NMR (471 MHz, $CDCl_3$) δ −61.8.

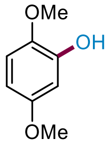

2,5-Dimethoxyphenol (3). The title compound was obtained by following the **General Procedure A** and known in the literature.[2] Colorless oil, yield = 70% (39 mg), current = 67 mA, electricity = 2.3 F mol^{-1}. ^{1}H NMR (500 MHz, $CDCl_3$) δ 6.78 (d, J = 8.8 Hz, 1H), 6.58 (d, J = 3.0 Hz, 1H), 6.39 (dd, J = 8.8, 2.9 Hz, 1H), 5.72 (s, 1H), 3.85 (s, 3H), 3.76 (s, 3H). ^{13}C NMR (126 MHz, $CDCl_3$) δ 154.8, 146.6, 141.2, 111.7, 104.5, 102.0, 56.8, 55.8.

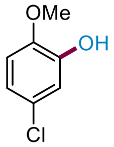

5-Chloro-2-methoxyphenol (4). The title compound was obtained by following the **General Procedure A** and known in the literature.[3] White solid, yield = 51% (29 mg), current = 98 mA, electricity = 3.4 F mol^{-1}. ^{1}H NMR (400 MHz, $CDCl_3$) δ 6.94 (d, J = 2.4 Hz, 1H), 6.83 (dd, J = 8.6, 2.4 Hz, 1H), 6.76 (d, J = 8.6 Hz, 1H), 5.69 (s, 1H), 3.88 (s, 3H). ^{13}C NMR (101 MHz, $CDCl_3$) δ 146.5, 145.6, 126.4, 120.0, 115.2, 111.5, 56.3.

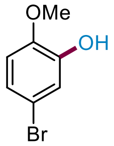

5-Bromo-2-methoxyphenol (5). The title compound was obtained by following the **General Procedure A** and known in the literature.[4] White solid, yield = 74% (54 mg), current = 98 mA, electricity = 3.4 F mol^{-1}. ^{1}H NMR (400 MHz, $CDCl_3$) δ 7.07 (d, J = 2.4 Hz, 1H), 6.97 (dd,

J = 8.6, 2.4 Hz, 1H), 6.72 (d, *J* = 8.5 Hz, 1H), 5.69 (s, 1H), 3.88 (s, 3H). ^{13}C NMR (101 MHz, $CDCl_3$) δ 146.7, 146.0, 123.0, 118.0, 113.4, 112.0, 56.3.

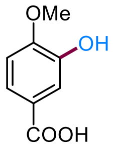

3-Hydroxy-4-methoxybenzoic acid (**6**). The title compound was obtained by following the **General Procedure A** and known in the literature.[5] White solid, yield = 44% (27 mg), current = 75 mA, electricity = 2.6 F mol^{-1}. ^{1}H NMR (500 MHz, DMSO) δ 12.48 (s, 1H), 9.34 (s, 1H), 7.41 (dd, *J* = 8.4, 2.1 Hz, 1H), 7.37 – 7.30 (m, 1H), 6.99 (d, *J* = 8.4 Hz, 1H), 3.82 (s, 3H). ^{13}C NMR (126 MHz, DMSO) δ 167.6, 152.1, 146.6, 123.5, 122.1, 116.5, 111.8, 56.1.

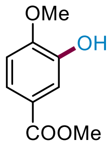

Methyl 3-hydroxy-4-methoxybenzoate (7). The title compound was obtained by following the **General Procedure A** and known in the literature.[6] White solid, yield = 96% (63 mg), current = 87 mA, electricity = 3.0 F mol^{-1}. ^{1}H NMR (500 MHz, $CDCl_3$) δ 7.72 – 7.49 (m, 2H), 6.85 (d, *J* = 8.2 Hz, 1H), 3.92 (s, 3H), 3.87 (s, 3H). ^{13}C NMR (126 MHz, $CDCl_3$) δ 167.1, 150.7, 145.4, 123.4, 122.9, 115.8, 110.0, 56.1, 52.1.

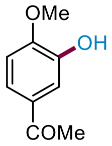

1-(3-Hydroxy-4-methoxyphenyl)ethan-1-one (8). The title compound was obtained by following the **General Procedure A** and known in the literature.[7] White solid, yield = 95% (57 mg), current = 81 mA, electricity = 2.8 F mol^{-1}. ^{1}H NMR (500 MHz, $CDCl_3$) δ 7.65 – 7.49 (m, 2H), 6.97 – 6.77 (m, 1H), 5.87 (s, 1H), 3.95 (s, 3H), 2.54 (s, 3H). ^{13}C NMR (126 MHz, $CDCl_3$) δ 197.2, 150.9, 145.6, 131.2, 122.0, 114.6, 110.0, 56.2, 26.5.

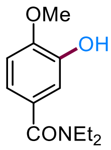

***N*,*N*-Diethyl-3-hydroxy-4-methoxybenzamide (9)**. The title compound was obtained by following the **General Procedure A** and known in the literature.[8] Pale yellow solid, yield = 58% (47 mg), current = 93 mA, electricity = 3.2 F mol^{-1}. ^{1}H NMR (500 MHz, $CDCl_3$) δ 6.96

(d, J = 2.0 Hz, 1H), 6.88 (dd, J = 8.3, 2.0 Hz, 1H), 6.83 (d, J = 8.3 Hz, 1H), 6.38 (s, 1H), 3.88 (s, 3H), 3.52 – 3.21 (m, 4H), 1.23 – 1.08 (m, 6H). ^{13}C NMR (126 MHz, $CDCl_3$) δ 171.3, 147.8, 145.7, 130.2, 118.7, 113.5, 110.7, 56.1, 43.7, 39.5, 14.2, 13.2.

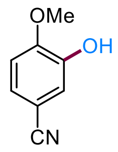

3-Hydroxy-4-methoxybenzonitrile (10). The title compound was obtained by following the **General Procedure A** and known in the literature.[9] Pale yellow oil, yield = 75% (41 mg), current = 81 mA, electricity = 2.8 F mol^{-1}. ^{1}H NMR (500 MHz, $CDCl_3$) δ 7.20 (dd, J = 8.4, 2.0 Hz, 1H), 7.16 (d, J = 2.0 Hz, 1H), 6.89 (d, J = 8.4 Hz, 1H), 5.91 (s, 1H), 3.96 (s, 3H). ^{13}C NMR (126 MHz, $CDCl_3$) δ 150.5, 146.1, 125.8, 119.2, 117.8, 111.0, 104.6, 56.3.

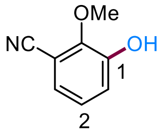

3-Hydroxy-2-methoxybenzonitrile (11). The title compound was obtained by following the **General Procedure A** and known in the literature.[10] Two regioisomers (**C1**:**C2** = 1.3:1) were formed as determined by ^{1}H NMR analysis of the crude reaction mixture. White solid, yield = 66% (35 mg), current = 81 mA, electricity = 2.8 F mol^{-1}. **C1** and **C2** were separated by chromatography. **C1**: ^{1}H NMR (500 MHz, $CDCl_3$) δ 7.18 (dd, J = 8.1, 1.7 Hz, 1H), 7.12 (dd, J = 7.9, 1.7 Hz, 1H), 7.07 – 7.02 (m, 1H), 5.98 (s, 1H), 4.15 (s, 3H). ^{13}C NMR (126 MHz, $CDCl_3$) δ 149.2, 149.0, 125.2, 124.8, 120.6, 116.6, 104.2, 62.0. **C2**: ^{1}H NMR (500 MHz, $CDCl_3$) δ 7.10 – 7.03 (m, 2H), 6.91 – 6.82 (m, 1H), 5.58 (s, 1H), 3.89 (s, 3H). ^{13}C NMR (126 MHz, $CDCl_3$) δ 156.0, 149.5, 122.2, 119.8, 116.5, 112.9, 101.7, 56.6.

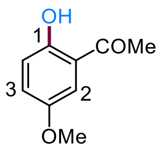

1-(2-Hydroxy-5-methoxyphenyl)ethan-1-one (12). The title compound was obtained by following the **General Procedure A** and known in the literature.[11] Three regioisomers (C1:C2:C3 = 3.6:2.4:1) were formed as determined by ^{1}H NMR analysis of the crude reaction mixture. Pale yellow oil, yield = 91% (55 mg), current = 61 mA, electricity = 2.1 F mol^{-1}. **C1**, **C2** and **C3** were separated by chromatography. **C1:** ^{1}H NMR (400 MHz, $CDCl_3$) δ 11.86 (s, 1H), 7.18 (d, J = 3.1 Hz, 1H), 7.12 (dd, J = 9.0, 3.0 Hz, 1H), 6.94 (d, J = 9.0 Hz, 1H), 3.82 (s, 3H), 2.63 (s, 3H). ^{13}C NMR (101 MHz, $CDCl_3$) δ 204.2, 157.0, 151.9, 124.3, 119.5, 119.4,

113.8, 56.2, 26.9. **C2:** ^{1}H NMR (500 MHz, $CDCl_3$) δ 12.56 (s, 1H), 7.34 (dd, J = 8.2, 1.4 Hz, 1H), 7.06 (dd, J = 8.0, 1.4 Hz, 1H), 6.85 (dd, J = 8.1, 8.0 Hz, 1H), 3.90 (s, 3H), 2.64 (s, 3H). ^{13}C NMR (126 MHz, $CDCl_3$) δ 205.1, 153.0, 149.1, 122.0, 119.9, 118.4, 117.2, 56.4, 27.2. **C3:** ^{1}H NMR (500 MHz, $CDCl_3$) δ 7.61 – 7.45 (m, 2H), 7.00 – 6.89 (m, 1H), 6.13 (s, 1H), 3.96 (s, 3H), 2.57 (s, 3H). ^{13}C NMR (126 MHz, $CDCl_3$) δ 197.0, 150.6, 146.8, 130.4, 124.2, 114.0, 109.9, 56.3, 26.4.

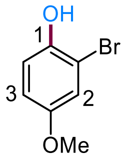

2-Bromo-4-methoxyphenol (13). The title compound was obtained by following the **General Procedure A** and known in the literature.[12] Three regioisomers (C1:C2:C3 = 2.8:1:2.4) were formed as determined by ^{1}H NMR analysis of the crude reaction mixture. Colorless oil, yield = 77% (57 mg), current = 64 mA, electricity = 2.2 F mol^{-1}. ^{1}H NMR (500 MHz, $CDCl_3$) δ 7.10 (dd, J = 8.1, 1.4 Hz, 0.17H), 7.03 (d, J = 2.9 Hz, 0.36H), 7.01 – 6.91 (m, 1.22H), 6.85 – 6.78 (m, 0.97H), 6.74 (m, 0.17H), 6.10 (s, 0.17H), 5.76 (s, 0.43H), 5.38 (s, 0.38H), 3.86 (s, 0.53H), 3.84 (s, 1.27H), 3.75 (s, 1.15H). ^{13}C NMR (126 MHz, $CDCl_3$) δ 153.8, 147.4, 147.3, 146.6, 144.9, 143.2, 124.9, 124.2, 120.7, 117.0, 116.5, 115.9, 115.3, 114.3, 111.7, 110.0 (2C), 108.5, 56.4, 56.2, 56.0.

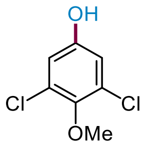

3,5-Dichloro-4-methoxyphenol (14). The title compound was obtained by following the **General Procedure A** and known in the literature.[13] White solid, yield = 41% (29 mg), current = 90 mA, electricity = 3.1 F mol^{-1}. ^{1}H NMR (400 MHz, $CDCl_3$) δ 6.81 (s, 2H), 5.79 (s, 1H), 3.86 (s, 3H). ^{13}C NMR (101 MHz, $CDCl_3$) δ 152.3, 146.1, 129.6, 116.2, 61.1.

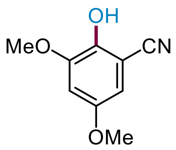

2-Hydroxy-3,5-dimethoxybenzonitrile (15). The title compound was obtained by following the **General Procedure A**. White solid, yield = 89% (57 mg), current = 67 mA, electricity = 2.3 F mol^{-1}. ^{1}H NMR (500 MHz, $CDCl_3$) δ 6.66 (d, J = 2.8 Hz, 1H), 6.52 (d, J = 2.8 Hz, 1H), 5.91 (s, 1H), 3.90 (s, 3H), 3.77 (s, 3H). ^{13}C NMR (126 MHz, $CDCl_3$) δ 153.3, 147.7, 144.0, 116.1, 105.2, 104.9, 98.0, 56.5, 56.1. IR (neat, cm^{-1}): 3393, 2919, 2220, 1621, 1266, 1046, 805.

ESI HRMS *m/z* $(M+Na)^+$ calcd 202.0475, obsd 202.0479.

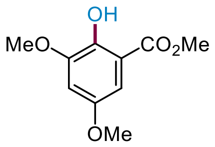

Methyl 2-hydroxy-3,5-dimethoxybenzoate (16). The title compound was obtained by following the **General Procedure A**. White solid, yield = 91% (69 mg), current = 64 mA, electricity = 2.2 F mol^{-1}. ^{1}H NMR (500 MHz, $CDCl_3$) δ 10.63 (s, 1H), 6.83 (d, J = 2.9 Hz, 1H), 6.68 (d, J = 2.9 Hz, 1H), 3.94 (s, 3H), 3.87 (s, 3H), 3.77 (s, 3H). ^{13}C NMR (126 MHz, $CDCl_3$) δ 170.8, 151.9, 149.5, 147.2, 111.6, 107.0, 101.4, 56.3, 55.8, 52.5. IR (neat, cm^{-1}): 3206, 2958, 1671, 1610, 1436, 1052, 735. ESI HRMS *m/z* $(M+Na)^+$ calcd 235.0577, obsd 235.0565.

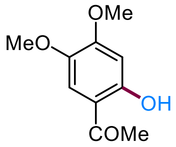

1-(2-Hydroxy-4,5-dimethoxyphenyl)ethan-1-one (17). The title compound was obtained by following the **General Procedure A** and known in the literature.[14] White solid, yield = 53% (38 mg), current = 93 mA, electricity = 3.2 F mol^{-1}. ^{1}H NMR (500 MHz, $CDCl_3$) δ 12.63 (s, 1H), 7.04 (s, 1H), 6.43 (s, 1H), 3.90 (s, 3H), 3.85 (s, 3H), 2.55 (s, 3H). ^{13}C NMR (126 MHz, $CDCl_3$) δ 202.2, 160.2, 156.9, 142.0, 111.8, 111.7, 100.7, 56.8, 56.3, 26.5.

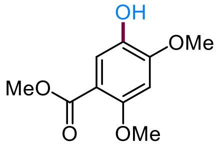

Methyl 5-hydroxy-2,4-dimethoxybenzoate (18). The title compound was obtained by following the **General Procedure A**. White solid, yield = 56% (43 mg), current = 87 mA, electricity = 3.0 F mol^{-1}. ^{1}H NMR (500 MHz, $CDCl_3$) δ 7.44 (s, 1H), 6.49 (s, 1H), 5.47 (s, 1H), 3.92 (s, 3H), 3.86 (s, 3H), 3.84 (s, 3H). ^{13}C NMR (126 MHz, $CDCl_3$) δ 166.1, 155.0, 150.9, 139.1, 117.4, 111.9, 97.3, 57.3, 56.1, 51.9. IR (neat, cm^{-1}): 3352, 2920, 1690, 1626, 1522, 1204, 829. ESI HRMS *m/z* $(M+Na)^+$ calcd 235.0577, obsd 235.0583.

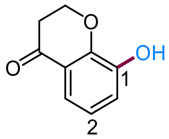

8-Hydroxychroman-4-one (19). The title compound was obtained by following the **General Procedure A** and known in the literature.[15] Two regioisomers (C1:C2 = 1:1) were formed as

determined by ^{1}H NMR analysis of the crude reaction mixture. White solid, yield = 72% (42 mg), current = 81 mA, electricity = 2.8 F mol^{-1}. **C1** and **C2** were separated by chromatography. **C1**: ^{1}H NMR (500 MHz, $CDCl_3$) δ 7.45 (dd, *J* = 8.0, 1.5 Hz, 1H), 7.13 (dd, *J* = 7.8, 1.6 Hz, 1H), 6.98 – 6.90 (m, 1H), 5.50 (s, 1H), 4.64 (t, *J* = 6.4 Hz, 2H), 2.86 (t, *J* = 6.4 Hz, 2H). ^{13}C NMR (126 MHz, $CDCl_3$) δ 191.5, 149.3, 145.4, 121.8, 121.7, 120.7, 118.1, 68.0, 38.1. **C2**: ^{1}H NMR (500 MHz, $CDCl_3$) δ 7.40 (d, *J* = 3.1 Hz, 1H), 7.08 (dd, *J* = 8.9, 3.1 Hz, 1H), 6.90 (d, *J* = 8.9 Hz, 1H), 6.00 (s, 1H), 4.49 (t, *J* = 6.5 Hz, 2H), 2.81 (t, *J* = 6.5 Hz, 2H). ^{13}C NMR (126 MHz, $CDCl_3$) δ 192.9, 156.6, 150.4, 125.1, 121.4, 119.4, 111.4, 67.2, 37.9.

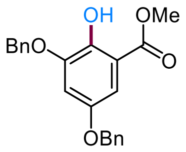

Methyl 3,5-bis(benzyloxy)-2-hydroxybenzoate (20). The title compound was obtained by following the **General Procedure A**. White solid, yield = 77% (101 mg), current = 67 mA, electricity = 2.3 F mol^{-1}. ^{1}H NMR (500 MHz, $CDCl_3$) δ 10.69 (s, 1H), 7.50 – 7.28 (m, 10H), 7.00 (d, *J* = 2.9 Hz, 1H), 6.81 (d, *J* = 2.9 Hz, 1H), 5.15 (s, 2H), 4.97 (s, 2H), 3.96 (s, 3H). ^{13}C NMR (126 MHz, $CDCl_3$) δ 170.7, 150.9, 148.4, 148.0, 136.9, 136.7, 128.8 (2C), 128.3, 128.2, 127.8, 127.6, 111.9, 110.1, 104.1, 71.4, 70.9, 52.6. IR (neat, cm^{-1}): 3360, 2920, 2849, 1675, 1605, 1439, 1217, 1058, 696. ESI HRMS *m/z* $(M+Na)^+$ calcd 387.1203, obsd 387.1214.

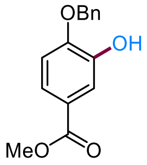

Methyl 4-(benzyloxy)-3-hydroxybenzoate (21). The title compound was obtained by following the **General Procedure A** and known in the literature.[16] White solid, yield = 62% (58 mg), current = 61 mA, electricity = 2.1 F mol^{-1}. ^{1}H NMR (500 MHz, $CDCl_3$) δ 7.63 (d, *J* = 2.1 Hz, 1H), 7.60 (dd, *J* = 8.4, 2.1 Hz, 1H), 7.46 – 7.35 (m, 5H), 6.95 (d, *J* = 8.4 Hz, 1H), 5.82 (s, 1H), 5.17 (s, 2H), 3.88 (s, 3H). ^{13}C NMR (126 MHz, $CDCl_3$) δ 166.9, 149.8, 145.6, 135.7, 129.0, 128.8, 128.0, 123.8, 122.8, 116.0, 111.4, 71.3, 52.1.

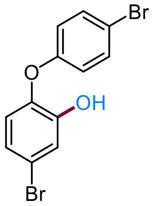

5-Bromo-2-(4-bromophenoxy)phenol (22). The title compound was obtained by following the **General Procedure A**. Colorless oil, yield = 81% (100 mg), current = 64 mA, electricity =

2.2 F mol^{-1}. ^{1}H NMR (500 MHz, $CDCl_3$) δ 7.46 (d, J = 8.5 Hz, 2H), 7.21 (d, J = 2.3 Hz, 1H), 6.98 (dd, J = 8.7, 2.3 Hz, 1H), 6.90 (d, J = 8.6 Hz, 2H), 6.74 (d, J = 8.6 Hz, 1H), 5.70 (s, 1H). ^{13}C NMR (126 MHz, $CDCl_3$) δ 155.7, 148.4, 142.7, 133.1, 123.9, 120.1, 119.9 (2C), 117.4, 116.7. IR (neat, cm^{-1}): 3507, 2921, 1596, 1483, 1209, 1058, 849.

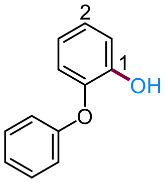

4-Phenoxyphenol (23). The title compound was obtained by following the **General Procedure A** and known in the literature.[17] Two regioisomers (C1:C2 = 2.4:1) were formed as determined by ^{1}H NMR analysis of the crude reaction mixture. White solid, yield = 83% (56 mg), current = 61 mA, electricity = 2.1 F mol^{-1}. **C1** and **C2** were separated by chromatography. **C1:** ^{1}H NMR (500 MHz, $CDCl_3$) δ 7.39 – 7.33 (m, 2H), 7.17 – 7.11 (m, 1H), 7.09 – 7.03 (m, 4H), 6.93 – 6.84 (m, 2H), 5.63 (s, 1H). ^{13}C NMR (126 MHz, $CDCl_3$) δ 157.0, 147.7, 143.7, 130.1, 125.0, 123.8, 120.8, 119.1, 118.2, 116.4. **C2:** ^{1}H NMR (500 MHz, $CDCl_3$) δ 7.34 – 7.29 (m, 2H), 7.08 – 7.04 (m, 1H), 6.98 – 6.92 (m, 4H), 6.85 – 6.80 (m, 2H), 4.90 (s, 1H). ^{13}C NMR (126 MHz, $CDCl_3$) δ 158.6, 151.9, 150.4, 129.8, 122.7, 121.2, 117.8, 116.5.

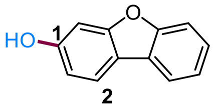

Dibenzo[*b*,*d*]furan-3-ol (24). The title compound was obtained by following the **General Procedure A** and known in the literature.[18] Two regioisomers (C1:C2 = 4.0:1) were formed as determined by ^{1}H NMR analysis of the crude reaction mixture. White solid, yield = 81% (54 mg), current = 69 mA, electricity = 2.4 F mol^{-1}. **C1** and **C2** were separated by chromatography. **C1:** ^{1}H NMR (500 MHz, MeOD) δ 7.85 (dd, J = 7.6, 1.3 Hz, 1H), 7.77 (d, J = 8.4 Hz, 1H), 7.47 (d, J = 8.1 Hz, 1H), 7.37 – 7.30 (m, 1H), 7.31 – 7.20 (m, 1H), 6.95 (d, J = 2.1 Hz, 1H), 6.83 (dd, J = 8.4, 2.1 Hz, 1H). ^{13}C NMR (126 MHz, MeOD) δ 159.1, 159.0, 157.5, 126.5, 125.9, 123.7, 122.0, 120.5, 117.5, 112.6, 112.0, 99.1. **C2:** ^{1}H NMR (500 MHz, MeOD) δ 8.09 (dd, J = 7.6, 1.3 Hz, 1H), 7.50 (d, J = 8.2 Hz, 1H), 7.43 – 7.36 (m, 1H), 7.35 – 7.28 (m, 1H), 7.28 – 7.18 (m, 1H), 7.03 (d, J = 8.1 Hz, 1H), 6.73 (d, J = 8.0 Hz, 1H). ^{13}C NMR (126 MHz, MeOD) δ 159.1, 156.8, 155.1, 128.9, 127.0, 125.1, 123.7 (2C), 113.7, 111.7, 109.4, 103.5.

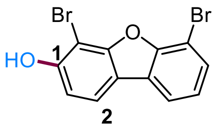

4,6-Dibromodibenzo[*b*,*d*]furan-3-ol (25). The title compound was obtained by following the **General Procedure A** and known in the literature. Two regioisomers (C1:C2 = 1.3:1) were formed as determined by ^{1}H NMR analysis of the crude reaction mixture. The title compound was obtained as a mixture of regioisomers (1.1:1). Yellow solid, yield = 69% (85 mg), current = 96 mA, electricity = 3.3 F mol^{-1}. Major isomer **C1:** ^{1}H NMR (500 MHz, DMSO) δ 10.88 (s, 1H), 7.99 – 7.93 (m, 1H), 7.89 (d, *J* = 8.4 Hz, 1H), 7.60 (d, *J* = 7.9 Hz, 1H), 7.30 – 7.23 (m, 1H), 7.06 (d, *J* = 8.4 Hz, 1H). Minor isomer **C2:** ^{1}H NMR (500 MHz, DMSO) δ 10.96 (s, 1H), 8.03 (d, *J* = 7.6 Hz, 1H), 7.68 (d, *J* = 8.0 Hz, 1H), 7.53 (d, *J* = 8.6 Hz, 1H), 7.36 – 7.31 (m, 1H), 6.81 (d, *J* = 8.6 Hz, 1H). ^{13}C NMR (126 MHz, DMSO) δ 155.3, 154.2, 153.6, 153.2, 152.1, 151.4, 131.3, 129.5, 128.8, 125.9, 125.3, 125.0, 124.9, 121.9, 120.8, 119.8, 116.2, 113.4, 112.8, 111.2, 103.3, 103.2, 91.9, 91.6.

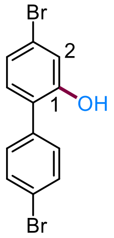

4,4'-Dibromo-[1,1'-biphenyl]-2-ol (26). The title compound was obtained by following the **General Procedure A**. Two regioisomers (C1:C2 = 9.0:1) were formed as determined by ^{1}H NMR analysis of the crude reaction mixture. The title compound was obtained as a mixture of regioisomers (8.3:1). White solid, yield = 94% (111 mg), current = 84 mA, electricity = 2.9 F mol^{-1}. Major isomer **C1:** ^{1}H NMR (500 MHz, $CDCl_3$) δ 7.64 – 7.59 (m, 2H), 7.35 – 7.31 (m, 2H), 7.16 – 7.13 (m, 2H), 7.11 – 7.07 (m, 1H), 5.23 (s, 1H). Minor isomer **C2:** ^{1}H NMR (500 MHz, $CDCl_3$) δ 7.58 – 7.55 (m, 2H), 7.52 (d, *J* = 8.3 Hz, 1H), 7.44 – 7.40 (m, 2H), 7.21 (d, *J* = 2.2 Hz, 1H), 7.01 (dd, *J* = 8.3, 2.2 Hz, 1H), 5.61 (s, 1H). ^{13}C NMR (126 MHz, $CDCl_3$) δ 153.2, 152.8, 135.3, 132.5, 132.2, 131.4, 130.8, 128.7, 126.4, 124.4, 122.5, 120.6, 119.5, 114.6.

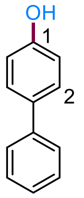

[1,1'-Biphenyl]-4-ol (27). The title compound was obtained by following the **General Procedure A** and known in the literature.[19] Two regioisomers (C1:C2 = 2.0:1) were formed as determined by ^{1}H NMR analysis of the crude reaction mixture. White solid, yield = 81% (49 mg), current = 84 mA, electricity = 2.9 F mol^{-1}. **C1** and **C2** were separated by chromatography. **C1:** ^{1}H NMR (500 MHz, $CDCl_3$) δ 7.58 – 7.54 (m, 2H), 7.52 – 7.47 (m, 2H), 7.46 – 7.41 (m, 2H), 7.35 – 7.30 (m, 1H), 6.95 – 6.90 (m, 2H), 5.04 (s, 1H). ^{13}C NMR (126 MHz, $CDCl_3$) δ

155.3, 141.0, 134.2, 128.9, 128.6, 126.9 (2C), 115.8. **C2:** ^{1}H NMR (500 MHz, $CDCl_3$) δ 7.49 – 7.42 (m, 4H), 7.41 – 7.33 (m, 1H), 7.27 – 7.19 (m, 2H), 7.00 – 6.93 (m, 2H), 5.22 (s, 1H). ^{13}C NMR (126 MHz, $CDCl_3$) δ 152.6, 137.3, 130.4, 129.5, 129.3 (2C), 128.3, 128.1, 121.0, 116.0.

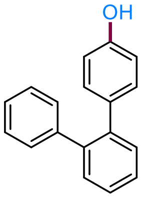

[1,1':2',1''-Terphenyl]-4-ol (28). The title compound was obtained by following the **General Procedure A**. White solid, yield = 50% (45 mg), current = 87 mA, electricity = 3.0 F mol^{-1}. ^{1}H NMR (500 MHz, $CDCl_3$) δ 7.45 – 7.37 (m, 4H), 7.28 – 7.19 (m, 3H), 7.18 – 7.13 (m, 2H), 7.05 – 6.98 (m, 2H), 6.72 – 6.65 (m, 2H), 4.69 (s, 1H). ^{13}C NMR (126 MHz, $CDCl_3$) δ 154.4, 141.9, 140.7, 140.3, 134.3, 131.3, 130.8, 130.7, 130.1, 128.1, 127.6, 127.3, 126.6, 115.0.

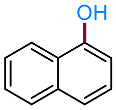

Naphthalen-1-ol (29). The title compound was obtained by following the **General Procedure A** and known in the literature.[20] White solid, yield = 85% (44 mg), current = 61 mA, electricity = 2.1 F mol^{-1}. ^{1}H NMR (500 MHz, $CDCl_3$) δ 8.25 – 8.18 (m, 1H), 7.87 – 7.83 (m, 1H), 7.59 – 7.48 (m, 2H), 7.48 (d, J = 8.2 Hz, 1H), 7.39 – 7.30 (m, 1H), 6.83 (d, J = 7.5 Hz, 1H). ^{13}C NMR (126 MHz, $CDCl_3$) δ 151.6, 134.9, 127.9, 126.6, 126.0, 125.4, 124.6, 121.7, 120.8, 108.8.

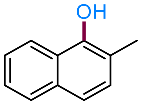

2-Methylnaphthalen-1-ol (30). The title compound was obtained by following the **General Procedure A** and known in the literature.[21] White solid, yield = 52% (30 mg), current = 61 mA, electricity = 2.1 F mol^{-1}. ^{1}H NMR (400 MHz, $CDCl_3$) δ 8.19 – 8.12 (m, 1H), 7.83 – 7.78 (m, 1H), 7.54 – 7.41 (m, 2H), 7.41 (d, J = 8.3 Hz, 1H), 7.27 (d, J = 8.3 Hz, 1H), 5.16 (s, 1H), 2.43 (s, 3H). ^{13}C NMR (101 MHz, $CDCl_3$) δ 148.7, 133.6, 129.2, 127.8, 125.5 (2C), 124.4, 121.0, 120.3, 116.5, 15.8.

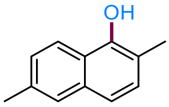

2,6-Dimethylnaphthalen-1-ol (31). The title compound was obtained by following the **General Procedure A** and known in the literature.[22] White solid, yield = 64% (40 mg), current = 61 mA, electricity = 2.1 F mol^{-1}. ^{1}H NMR (500 MHz, $CDCl_3$) δ 8.04 (d, J = 8.5 Hz, 1H), 7.57

(s, 1H), 7.37 – 7.30 (m, 2H), 7.23 (d, J = 8.3 Hz, 1H), 5.10 (s, 1H), 2.52 (s, 3H), 2.41 (s, 3H). ^{13}C NMR (126 MHz, $CDCl_3$) δ 148.7, 135.1, 133.9, 129.2, 127.7, 126.8, 122.7, 120.9, 119.7, 115.6, 21.7, 15.7.

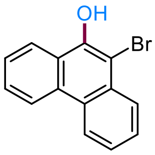

10-Bromophenanthren-9-ol (32). The title compound was obtained by following the **General Procedure A** and known in the literature.[23] White solid, yield = 63% (62 mg), current = 61 mA, electricity = 2.1 F mol^{-1}. ^{1}H NMR (500 MHz, $CDCl_3$) δ 8.63 (d, J = 8.3 Hz, 1H), 8.60 (d, J = 8.2 Hz, 1H), 8.39 (dd, J = 8.0, 1.5 Hz, 1H), 8.14 (dd, J = 8.2, 1.2 Hz, 1H), 7.76 – 7.68 (m, 1H), 7.70 – 7.62 (m, 2H), 7.60 – 7.53 (m, 1H), 6.31 (s, 1H). ^{13}C NMR (126 MHz, $CDCl_3$) δ 147.2, 130.5 (2C), 128.0, 127.9, 127.4, 127.2, 126.4, 125.1, 125.0, 123.4, 122.8, 103.8.

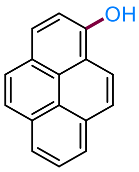

Pyren-1-ol (33). The title compound was obtained by following the **General Procedure A** and known in the literature.[24] White solid, yield = 60% (47 mg), current = 52 mA, electricity = 1.8 F mol^{-1}. ^{1}H NMR (600 MHz, MeOD) δ 8.38 (d, J = 9.1 Hz, 1H), 8.07 – 8.03 (m, 2H), 8.01 (d, J = 8.2 Hz, 1H), 7.97 (d, J = 9.1 Hz, 1H), 7.93 – 7.89 (m, 2H), 7.82 (d, J = 8.9 Hz, 1H), 7.51 (d, J = 8.2 Hz, 1H). ^{13}C NMR (151 MHz, MeOD) δ 151.9, 131.9, 131.8, 127.0, 125.9, 125.6, 125.4, 125.2, 124.9, 124.5, 123.7, 123.5, 123.3, 121.1, 118.6, 112.4.

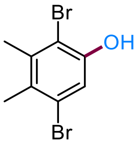

2,5-Dibromo-3,4-dimethylphenol (34). The title compound was obtained by following the **General Procedure A**. Yellow solid, yield = 30% (30 mg), current = 87 mA, electricity = 3.0 F mol^{-1}. ^{1}H NMR (500 MHz, $CDCl_3$) δ 7.17 (s, 1H), 5.52 (s, 1H), 2.45 (s, 3H), 2.38 (s, 3H). ^{13}C NMR (126 MHz, $CDCl_3$) δ 150.6, 137.7, 129.2, 124.8, 117.2, 113.4, 21.5, 20.4.

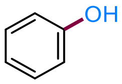

Phenol (35). The title compound was obtained by following the **General Procedure B** and

known in the literature.[25] GC analysis was obtained on an Agilent 6890A GC. Yield = 96% (determined via GC), current = 64 mA, electricity = 2.2 F mol^{-1}.

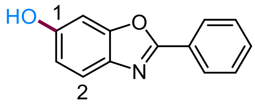

2-Phenylbenzo[*d*]oxazol-6-ol (36). The title compound was obtained by following the **General Procedure A** and known in the literature.[26] Two regioisomers (C1:C2 = 13:1) were formed as determined by ^{1}H NMR analysis of the crude reaction mixture. White solid, yield = 69% (53mg), current = 64 mA, electricity = 2.2 F mol^{-1}. **C1** and **C2** were separated by chromatography. **C1:** ^{1}H NMR (500 MHz, MeOD) δ 8.18 – 8.10 (m, 2H), 7.56 – 7.51 (m, 3H), 7.50 (d, *J* = 8.6 Hz, 1H), 7.04 (d, *J* = 2.2 Hz, 1H), 6.87 (dd, *J* = 8.6, 2.3 Hz, 1H). ^{13}C NMR (126 MHz, MeOD) δ 163.2, 157.8, 153.0, 135.5, 132.4, 130.1, 128.3, 128.1, 120.5, 114.7, 98.2. **C2:** ^{1}H NMR (500 MHz, MeOD) δ 8.28 – 8.19 (m, 2H), 7.61 – 7.53 (m, 3H), 7.26 – 7.17 (m, 1H), 7.13 (d, *J* = 8.1 Hz, 1H), 6.78 (d, *J* = 8.0 Hz, 1H). ^{13}C NMR (126 MHz, MeOD) δ 162.8, 153.7, 150.8, 132.6, 131.7, 130.1, 128.4, 128.3, 127.3, 111.4, 102.7.

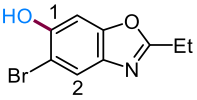

5-Bromo-2-ethylbenzo[*d*]oxazol-6-ol (37). The title compound was obtained by following the **General Procedure A**. Two regioisomers (C1:C2 = 11:1) were formed as determined by ^{1}H NMR analysis of the crude reaction mixture. The title compound was obtained as a mixture of regioisomers (11:1). White solid, yield = 50% (44 mg), current = 72 mA, electricity = 2.5 F mol^{-1}. Major isomer **C1:** ^{1}H NMR (400 MHz, MeOD) δ 7.67 (s, 1H), 7.06 (s, 1H), 2.90 (q, *J* = 7.6 Hz, 2H), 1.38 (t, *J* = 7.6 Hz, 3H). ^{13}C NMR (126 MHz, MeOD) δ 169.6, 153.3, 152.1, 135.6, 123.1, 107.8, 98.7, 22.7, 11.1.

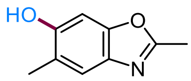

2,5-Dimethylbenzo[*d*]oxazol-6-ol (38). The title compound was obtained by following the **General Procedure A**. White solid, yield = 62% (36 mg), current = 70 mA, electricity = 2.4 F mol^{-1}. ^{1}H NMR (500 MHz, MeOD) δ 7.26 (s, 1H), 6.91 (s, 1H), 2.54 (s, 3H), 2.26 (s, 3H). ^{13}C NMR (126 MHz, MeOD) δ 162.5, 153.6, 149.9, 132.8, 122.2, 118.7, 95.7, 15.3, 12.6. IR (neat, cm^{-1}): 3449, 2919, 1632, 1470. ESI HRMS *m/z* (M+H)$^+$ calcd 164.0706, obsd 164.0702.

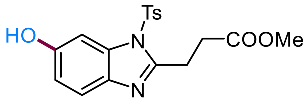

Methyl 3-(6-hydroxy-1-tosyl-1*H*-benzo[*d*]imidazol-2-yl)propanoate (39). The title compound was obtained by following the **General Procedure A without treatment of 2N HCl**. White solid, yield = 50% (67 mg), current = 81 mA, electricity = 2.8 F mol^{-1}. ^{1}H NMR (500 MHz, DMSO) δ 9.71 (s, 1H), 7.91 – 7.85 (m, 2H), 7.48 (d, *J* = 8.1 Hz, 2H), 7.41 (d, *J* = 8.7 Hz, 1H), 7.30 (d, *J* = 2.4 Hz, 1H), 6.77 (dd, *J* = 8.6, 2.3 Hz, 1H), 3.59 (s, 3H), 3.39 (t, *J* = 6.7 Hz, 2H), 2.92 (t, *J* = 6.7 Hz, 2H), 2.37 (s, 3H). ^{13}C NMR (126 MHz, DMSO) δ 172.9, 152.0, 146.7, 134.8, 133.7, 131.0, 127.1, 120.5, 113.9, 99.7, 51.9, 30.5, 25.0, 21.5. IR (neat, cm^{-1}): 3416, 2981, 2830, 2255, 1654, 1026, 703. ESI HRMS *m/z* $(M+Na)^+$ calcd 397.0829, obsd 397.0833.

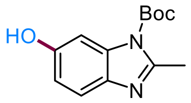

***tert*-Butyl 6-hydroxy-2-methyl-1*H*-benzo[*d*]imidazole-1-carboxylate (40).** The title compound was obtained by following the **General Procedure A without treatment of 2N HCl**. White solid, yield = 44% (40 mg), current = 61 mA, electricity = 2.1 F mol^{-1}. ^{1}H NMR (500 MHz, MeOD) δ 7.40 (d, *J* = 2.4 Hz, 1H), 7.34 (d, *J* = 8.6 Hz, 1H), 6.80 (dd, *J* = 8.6, 2.4 Hz, 1H), 2.71 (s, 3H), 1.71 (s, 9H). ^{13}C NMR (126 MHz, MeOD) δ 156.5, 153.2, 150.2, 135.8, 135.0, 119.6, 114.1, 102.5, 86.9, 28.3, 18.2. IR (neat, cm^{-1}): 3358, 2920, 2850, 1748, 1346, 1151. ESI HRMS *m/z* $(M+Na)^+$ calcd 271.1053, obsd 271.1059.

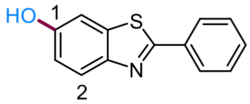

2-Phenylbenzo[*d*]thiazol-6-ol (41). The title compound was obtained by following the **General Procedure A** and known in the literature.[27] Two regioisomers (C1:C2 = 2.7:1) were formed as determined by ^{1}H NMR analysis of the crude reaction mixture. White solid, yield = 57% (47 mg), current = 61 mA, electricity = 2.1 F mol^{-1}. **C1** and **C2** were separated by chromatography. **C1:** ^{1}H NMR (500 MHz, MeOD) δ 8.04 – 7.97 (m, 2H), 7.82 (d, *J* = 8.8 Hz, 1H), 7.55 – 7.47 (m, 3H), 7.33 (d, *J* = 2.4 Hz, 1H), 7.01 (dd, *J* = 8.8, 2.4 Hz, 1H). ^{13}C NMR (101 MHz, MeOD) δ 166.6, 157.3, 148.8, 137.5, 134.8, 131.8, 130.2, 128.1, 124.3, 117.3, 107.5. **C2:** ^{1}H NMR (500 MHz, MeOD) δ 8.13 – 8.08 (m, 2H), 7.54 – 7.47 (m, 3H), 7.41 (dd, *J* = 8.0, 1.0 Hz, 1H), 7.29 – 7.19 (m, 1H), 6.90 (dd, *J* = 7.9, 1.0 Hz, 1H). ^{13}C NMR (101 MHz, MeOD) δ 167.3, 152.7, 144.8, 137.6, 134.9, 132.0, 130.2, 128.5, 127.8, 113.5, 112.3.

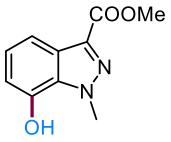

Methyl 4-hydroxy-1-methyl-1*H*-indazole-3-carboxylate (42). The title compound was obtained by following the **General Procedure A**. White solid, yield = 58% (43 mg), current = 72 mA, electricity = 2.5 F mol^{-1}. ^{1}H NMR (500 MHz, $CDCl_3$) δ 7.73 (d, *J* = 8.2 Hz, 1H), 7.13 – 7.04 (m, 1H), 6.86 – 6.68 (m, 2H), 4.42 (s, 3H), 4.03 (s, 3H). 13C NMR (126 MHz, $CDCl_3$) δ 163.5, 142.7, 134.2, 132.3, 126.6, 124.3, 114.0, 110.7, 52.3, 40.0. IR (neat, cm^{-1}): 3226, 2953, 1713, 1588, 1482, 1237, 1132, 735. ESI HRMS *m/z* $(M+Na)^+$ calcd 229.0584, obsd 229.0584.

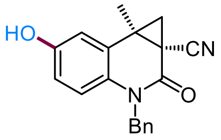

(1a*R*,7b*R*)-3-Benzyl-6-hydroxy-7b-methyl-2-oxo-1,2,3,7b-tetrahydro-1a*H*-cyclopropa[*c*]quinoline-1a-carbonitrile (43). The title compound was obtained by following the **General Procedure A**. White solid, yield = 40% (44 mg), current = 64 mA, electricity = 2.2 F mol^{-1}. ^{1}H NMR (500 MHz, DMSO) δ 9.43 (s, 1H), 7.34 – 7.29 (m, 2H), 7.25 – 7.18 (m, 3H), 7.01 (d, *J* = 2.7 Hz, 1H), 6.76 (d, *J* = 9.0 Hz, 1H), 6.59 (dd, *J* = 9.0, 2.7 Hz, 1H), 5.20 – 4.92 (m, 2H), 2.27 (d, *J* = 5.2 Hz, 1H), 1.76 (s, 3H), 1.53 (d, *J* = 5.2 Hz, 1H). ^{13}C NMR (126 MHz, DMSO) δ 162.5, 153.7, 137.0, 129.1, 127.4, 127.1, 126.8, 126.1, 117.8, 117.6, 114.9, 114.0, 45.8, 31.2, 26.4, 25.9, 20.2. IR (neat, cm^{-1}): 3448, 2257, 2129, 1648, 995, 653. ESI HRMS *m/z* $(M+Na)^+$ calcd 327.1104, obsd 327.1105.

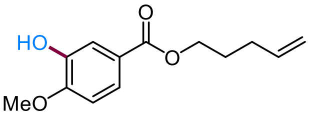

Pent-4-en-1-yl 3-hydroxy-4-methoxybenzoate (44). The title compound was obtained by following the **General Procedure A**. Colorless oil, yield = 90% (76 mg), current = 64 mA, electricity = 2.2 F mol^{-1}. ^{1}H NMR (500 MHz, $CDCl_3$) δ 7.64 – 7.58 (m, 2H), 6.87 (d, *J* = 8.4 Hz, 1H), 5.90 – 5.81 (m, 1H), 5.80 (s, 1H), 5.11 – 5.03 (m, 1H), 5.03 – 4.95 (m, 1H), 4.30 (t, *J* = 6.5 Hz, 2H), 3.93 (s, 3H), 2.25 – 2.16 (m, 2H), 1.91 – 1.81 (m, 2H). ^{13}C NMR (126 MHz, $CDCl_3$) δ 166.5, 150.6, 145.4, 137.7, 123.8, 122.9, 115.7, 115.4, 110.0, 64.3, 56.2, 30.3, 28.1. IR (neat, cm^{-1}): 3411, 2922, 1709, 1511, 1280, 1125, 752. ESI HRMS *m/z* $(M+Na)^+$ calcd 259.0941, obsd 259.0947.

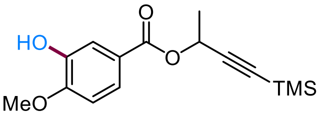

4-(Trimethylsilyl)but-3-yn-2-yl 3-hydroxy-4-methoxybenzoate (45). The title compound was obtained by following the **General Procedure A**. Colorless oil, yield = 68% (71 mg), current = 70 mA, electricity = 2.4 F mol^{-1}. ^{1}H NMR (500 MHz, $CDCl_3$) δ 7.69 – 7.59 (m, 2H),

6.87 (d, *J* = 8.4 Hz, 1H), 5.73 (s, 1H), 5.69 (q, *J* = 6.7 Hz, 1H), 3.95 (s, 3H), 1.59 (d, *J* = 6.7 Hz, 3H), 0.18 (s, 9H). ^{13}C NMR (126 MHz, $CDCl_3$) δ 165.2, 150.7, 145.4, 123.4, 123.2, 116.0, 110.0, 104.0, 89.6, 61.2, 56.2, 21.8, 0.0. IR (neat, cm^{-1}): 3434, 2959, 2177, 1716, 1511, 1279, 751. ESI HRMS *m/z* $(M+Na)^+$ calcd 315.1023, obsd 315.1024.

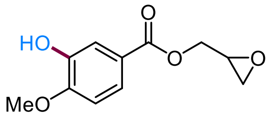

Oxiran-2-ylmethyl 3-hydroxy-4-methoxybenzoate (46). The title compound was obtained by following the **General Procedure A**. Colorless oil, yield = 90% (72 mg), current = 75 mA, electricity = 2.6 F mol^{-1}. ^{1}H NMR (500 MHz, $CDCl_3$) δ 7.64 (dd, *J* = 8.4, 2.1 Hz, 1H), 7.61 (d, *J* = 2.1 Hz, 1H), 6.87 (d, *J* = 8.4 Hz, 1H), 5.81 (s, 1H), 4.61 (dd, *J* = 12.3, 3.1 Hz, 1H), 4.15 (dd, *J* = 12.3, 6.2 Hz, 1H), 3.95 (s, 3H), 3.36 – 3.30 (m, 1H), 2.89 (t, *J* = 4.5 Hz, 1H), 2.73 (dd, *J* = 4.9, 2.6 Hz, 1H). ^{13}C NMR (126 MHz, $CDCl_3$) δ 166.1, 150.9, 145.4, 123.2, 122.9, 115.9, 110.0, 65.4, 56.2, 49.7, 44.9. IR (neat, cm^{-1}): 3362, 2920, 2849, 1708, 1512, 1281, 1022, 762. ESI HRMS *m/z* $(M+Na)^+$ calcd 247.0577, obsd 247.0577.

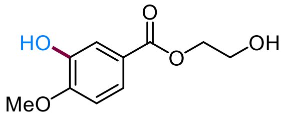

2-Hydroxyethyl 3-hydroxy-4-methoxybenzoate (47). The title compound was obtained by following the **General Procedure A**. Colorless oil, yield = 71% (54 mg), current = 75 mA, electricity = 2.6 F mol^{-1}. ^{1}H NMR (500 MHz, $CDCl_3$) δ 7.63 (dd, *J* = 8.4, 2.1 Hz, 1H), 7.60 (d, *J* = 2.1 Hz, 1H), 6.87 (d, *J* = 8.4 Hz, 1H), 5.77 (s, 1H), 4.46 – 4.37 (m, 2H), 3.95 (s, 5H), 2.23 (s, 1H). ^{13}C NMR (126 MHz, $CDCl_3$) δ 166.8, 150.8, 145.4, 123.2, 123.1, 115.8, 110.1, 66.7, 61.7, 56.2. IR (neat, cm^{-1}):3416, 2844, 2036, 1616, 1441, 1296, 1018, 764. ESI HRMS *m/z* $(M+Na)^+$ calcd 235.0577, obsd 235.0577.

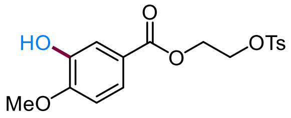

2-(Tosyloxy)ethyl 3-hydroxy-4-methoxybenzoate (48). The title compound was obtained by following the **General Procedure A**. White solid, yield = 85% (112 mg), current = 70 mA, electricity = 2.4 F mol^{-1}. ^{1}H NMR (500 MHz, $CDCl_3$) δ 7.79 (d, *J* = 8.3 Hz, 2H), 7.54 (dd, *J* = 8.4, 2.1 Hz, 1H), 7.44 (d, *J* = 2.1 Hz, 1H), 7.30 (d, *J* = 8.0 Hz, 2H), 6.86 (d, *J* = 8.5 Hz, 1H), 4.49 – 4.40 (m, 2H), 4.37 – 4.31 (m, 2H), 3.96 (s, 3H), 2.40 (s, 3H). ^{13}C NMR (126 MHz, $CDCl_3$) δ 165.9, 150.9, 145.4, 145.2, 132.9, 130.1, 128.1, 123.3, 122.7, 115.8, 110.0, 68.0, 62.1, 56.2, 21.8. IR (neat, cm^{-1}): 3395, 2073, 1645, 1295, 1035, 689. ESI HRMS *m/z* $(M+Na)^+$ calcd 389.0665, obsd 389.0666.

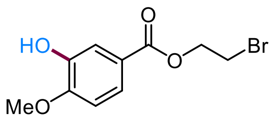

2-Bromoethyl 3-hydroxy-4-methoxybenzoate (49). The title compound was obtained by following the **General Procedure A**. Yellow oil, yield = 82% (81 mg), current = 70 mA, electricity = 2.4 F mol^{-1}. ^{1}H NMR (500 MHz, $CDCl_3$) δ 7.64 (dd, *J* = 8.4, 2.1 Hz, 1H), 7.61 (d, *J* = 2.1 Hz, 1H), 6.88 (d, *J* = 8.4 Hz, 1H), 5.82 (s, 1H), 4.58 (t, *J* = 6.1 Hz, 2H), 3.94 (s, 3H), 3.62 (t, *J* = 6.1 Hz, 2H). ^{13}C NMR (126 MHz, $CDCl_3$) δ 165.9, 150.9, 145.5, 123.2, 122.9, 115.9, 110.1, 64.2, 56.2, 29.0. IR (neat, cm^{-1}): 3427, 1699, 1512, 1440, 1215, 752. ESI HRMS *m/z* $(M+Na)^+$ calcd 296.9733, obsd 296.9738.

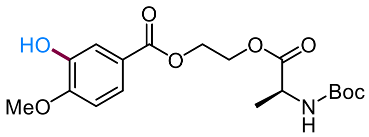

2-(((*tert*-Butoxycarbonyl)-*L*-alanyl)oxy)ethyl 3-hydroxy-4-methoxybenzoate (50). The title compound was obtained by following the **General Procedure A**. Colorless oil, yield = 82% (113 mg), current = 70 mA, electricity = 2.4 F mol^{-1}. ^{1}H NMR (500 MHz, $CDCl_3$) δ 7.65 – 7.44 (m, 2H), 6.85 (d, *J* = 8.4 Hz, 1H), 6.18 (s, 1H), 5.21 – 4.84 (m, 1H), 4.50 – 4.40 (m, 4H), 4.38 – 4.16 (m, 1H), 3.92 (s, 3H), 1.42 (s, 9H), 1.37 (d, *J* = 7.2 Hz, 3H). ^{13}C NMR (126 MHz, $CDCl_3$) δ 173.3, 166.1, 155.3, 151.0, 145.5, 123.0, 122.8, 115.9, 110.2, 80.1, 63.0, 62.3, 56.1, 49.3, 28.4, 18.6. IR (neat, cm^{-1}): 3368, 2978, 2037, 1711, 1614, 1518, 1282, 753. ESI HRMS *m/z* $(M+Na)^+$ calcd 406.1472, obsd 406.1473.

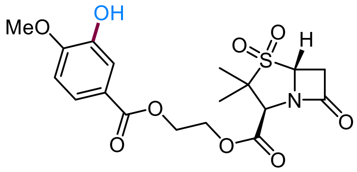

2-((3-Hydroxy-4-methoxybenzoyl)oxy)ethyl (2*S*,5*R*)-3,3-dimethyl-7-oxo-4-thia-1-azabicyclo[3.2.0]heptane-2-carboxylate 4,4-dioxide (51). The title compound was obtained by following the **General Procedure A**. Colorless oil, yield = 82% (126 mg), current = 70 mA, electricity = 2.4 F mol^{-1}. ^{1}H NMR (500 MHz, $CDCl_3$) δ 7.57 (dd, *J* = 8.5, 2.1 Hz, 1H), 7.52 (d, *J* = 2.1 Hz, 1H), 6.87 (d, *J* = 8.5 Hz, 1H), 5.94 (s, 1H), 4.62 – 4.43 (m, 5H), 4.41 (s, 1H), 3.93 (s, 3H), 3.50 – 3.38 (m, 2H), 1.57 (s, 3H), 1.38 (s, 3H). ^{13}C NMR (126 MHz, $CDCl_3$) δ 170.9, 167.0, 165.9, 151.1, 145.5, 123.1, 122.5, 115.7, 110.2, 64.2, 63.3, 62.8, 61.9, 61.2, 56.2, 38.4, 20.3, 18.5. IR (neat, cm^{-1}): 3444, 2848, 2037, 1794, 1615, 1280, 763. ESI HRMS *m/z* $(M+Na)^+$ calcd 450.0829, obsd 450.0831.

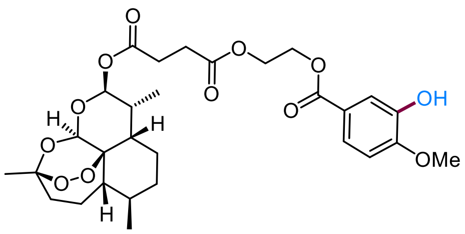

2-((3-Hydroxy-4-methoxybenzoyl)oxy)ethyl ((3*R*,5a*S*,6*R*,8a*S*,9*R*,10*S*,12*R*,12a*R*)-3,6,9-trimethyldecahydro-12*H*-3,12-epoxy[1,2]dioxepino[4,3-*i*]isochromen-10-yl) succinate (52). The title compound was obtained by following the **General Procedure A**. Pale yellow oil, yield = 70% (146 mg), current = 75 mA, electricity = 2.6 F mol^{-1}. ^{1}H NMR (500 MHz, $CDCl_3$) δ 7.66 – 7.52 (m, 2H), 6.88 (d, *J* = 8.4 Hz, 1H), 5.87 (s, 1H), 5.77 (d, *J* = 9.8 Hz, 1H), 5.41 (s, 1H), 4.50 – 4.38 (m, 4H), 3.94 (s, 3H), 2.80 – 2.65 (m, 4H), 2.59 – 2.51 (m, 1H), 2.40 – 2.31 (m, 1H), 2.06 – 2.00 (m, 1H), 1.88 – 1.81 (m, 1H), 1.76 – 1.66 (m, 2H), 1.62 – 1.56 (m, 1H), 1.42 (s, 4H), 1.36 – 1.26 (m, 3H), 1.01 – 0.91 (m, 4H), 0.83 (d, *J* = 7.1 Hz, 3H). ^{13}C NMR (126 MHz, $CDCl_3$) δ 172.1, 171.2, 166.1, 150.9, 145.5, 123.1 (2C), 115.9, 110.1, 104.6, 92.4, 91.6, 80.2, 62.7, 62.5, 56.2, 51.7, 45.4, 37.4, 36.4, 34.2, 31.9, 29.3, 28.9, 26.1, 24.7, 22.1, 20.3, 12.1. IR (neat, cm^{-1}): 3443, 2925, 1740, 1511, 1280, 1016, 763. ESI HRMS *m/z* $(M+H)^+$ calcd 601.2255, obsd 601.2252.

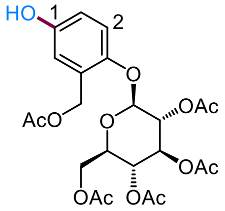

(2*R*,3*R*,4*S*,5*R*,6*S*)-2-(Acetoxymethyl)-6-(2-(acetoxymethyl)-4-hydroxyphenoxy)tetrahydro-2*H*-pyran-3,4,5-triyl triacetate (53). The title compound was obtained by following the **General Procedure A** and known in the literature.[19a] Two regioisomers (C1:C2 = 2.6:1) were formed as determined by ^{1}H NMR analysis of the crude reaction mixture. Only the major regioisomer was shown. White solid, yield = 47% (86.1 mg), current = 81 mA, electricity = 2.8 F mol^{-1}. ^{1}H NMR (500 MHz, $CDCl_3$) δ 6.99 (d, *J* = 8.8 Hz, 1H), 6.83 (d, *J* = 3.0 Hz, 1H), 6.73 (dd, *J* = 8.8, 3.1 Hz, 1H), 5.98 (s, 1H), 5.30 – 5.25 (m, 2H), 5.20 – 5.14 (m, 1H), 5.08 (d, *J* = 13.0 Hz, 1H), 5.00 (d, *J* = 13.0 Hz, 1H), 4.95 – 4.90 (m, 1H), 4.29 – 4.25 (m, 1H), 4.22 – 4.17 (m, 1H), 3.82 – 3.76 (m, 1H), 2.11 (s, 3H), 2.10 (s, 3H), 2.09 (s, 3H), 2.04 (s, 3H), 2.04 (s, 3H).

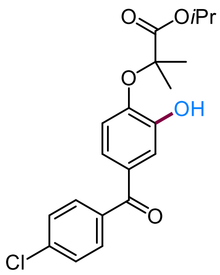

Isopropyl 2-(4-(4-chlorobenzoyl)-2-hydroxyphenoxy)-2-methylpropanoate (54). The title compound was obtained by following the **General Procedure A**. White solid, yield = 63% (85 mg), current = 64 mA, electricity = 2.2 F mol^{-1}. ^{1}H NMR (500 MHz, $CDCl_3$) δ 7.97 (s, 1H), 7.73 (d, J = 8.5 Hz, 2H), 7.44 (d, J = 8.5 Hz, 2H), 7.38 (d, J = 2.1 Hz, 1H), 7.24 (dd, J = 8.3, 2.2 Hz, 1H), 6.94 (d, J = 8.4 Hz, 1H), 5.18 – 5.08 (m, 1H), 1.61 (s, 6H), 1.28 (d, J = 6.3 Hz, 6H). ^{13}C NMR (126 MHz, $CDCl_3$) δ 194.6, 175.5, 150.1, 146.1, 138.8, 136.3, 133.9, 131.5, 128.7, 122.0, 121.7, 118.1, 81.8, 70.7, 25.7, 21.7. IR (neat, cm^{-1}): 3358, 2919, 1655, 1504, 1101, 757. ESI HRMS *m/z* (M+H)$^+$ calcd 399.0970, obsd 399.0969.

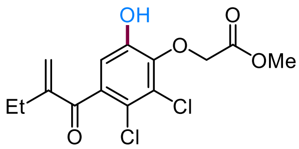

Methyl 2-(2,3-dichloro-6-hydroxy-4-(2-methylenebutanoyl)phenoxy)acetate (55). The title compound was obtained by following the **General Procedure A**. White solid, yield = 41% (49 mg), current = 75 mA, electricity = 2.6 F mol^{-1}. ^{1}H NMR (500 MHz, $CDCl_3$) δ 8.77 (s, 1H), 6.83 (s, 1H), 6.00 – 5.95 (m, 1H), 5.66 (s, 1H), 4.72 (s, 2H), 3.87 (s, 3H), 2.46 (q, J = 7.4 Hz, 2H), 1.15 (t, J = 7.4 Hz, 3H). ^{13}C NMR (126 MHz, $CDCl_3$) δ 195.6, 173.5, 149.9, 149.6, 145.0, 137.6, 129.5, 127.7, 120.4, 115.3, 70.9, 53.5, 23.4, 12.6. IR (neat, cm^{-1}): 3268, 2927, 1731, 1668, 1460, 1057, 879. ESI HRMS *m/z* (M+Na)$^+$ calcd 355.0111, obsd 355.0112.

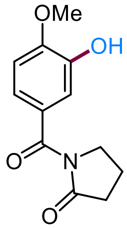

1-(3-Hydroxy-4-methoxybenzoyl)pyrrolidin-2-one (56). The title compound was obtained by following the **General Procedure A** and known in the literature.[28] White solid, yield = 87% (74 mg), current = 61 mA, electricity = 2.1 F mol^{-1}. ^{1}H NMR (500 MHz, $CDCl_3$) δ 7.25 – 7.16 (m, 2H), 6.83 (d, J = 8.2 Hz, 1H), 5.96 (s, 1H), 3.93 – 3.86 (m, 5H), 2.58 (t, J = 7.9 Hz, 2H), 2.15 – 2.05 (m, 2H). ^{13}C NMR (126 MHz, $CDCl_3$) δ 174.8, 170.2, 150.2, 145.0, 127.1, 122.7, 115.9, 109.7, 56.0, 46.9, 33.4, 17.8.

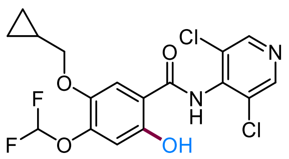

5-(Cyclopropylmethoxy)-*N*-(3,5-dichloropyridin-4-yl)-4-(difluoromethoxy)-2-hydroxybenzamide (57). The title compound was obtained by following the **General Procedure A**. Brown solid, yield = 35% (53 mg), current = 81 mA, electricity = 2.8 F mol^{-1}. ^{1}H NMR (500 MHz, $CDCl_3$) δ 8.57 (s, 2H), 7.37 (s, 1H), 6.85 (s, 1H), 6.74 (t, $J_{H\text{-}F}$ = 74.3 Hz, 1H). 3.85 (d, J = 7.0 Hz, 2H), 1.34 – 1.21 (m, 1H), 0.68 – 0.60 (m, 2H), 0.36 – 0.29 (m, 2H). ^{13}C NMR (126 MHz, $CDCl_3$) δ 166.3, 156.3, 148.4, 147.2, 143.4, 139.6, 129.7, 115.7 (t, $J_{C\text{-}F}$ = 261.7 Hz), 114.7, 111.1 (2C), 76.0, 10.4, 3.4. ^{19}F NMR (376 MHz, $CDCl_3$) δ –82.1, –82.3. IR (neat, cm^{-1}): 3251, 2926, 1655, 1494, 1208, 1001. ESI HRMS *m/z* (M+Na)$^{+}$ calcd 441.0191, obsd 441.0201.

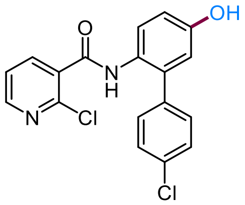

2-Chloro-*N*-(4'-chloro-5-hydroxy-[1,1'-biphenyl]-2-yl)nicotinamide (58). The title compound was obtained by following the **General Procedure A** and known in the literature.[10] White solid, yield = 50% (64 mg), current = 61 mA, electricity = 2.1 F mol^{-1}. ^{1}H NMR (500 MHz, MeOD) δ 8.41 (dd, J = 4.9, 1.9 Hz, 1H), 7.73 (dd, J = 7.6, 1.9 Hz, 1H), 7.47 – 7.37 (m, 5H), 7.35 (d, J = 8.6 Hz, 1H), 6.87 (dd, J = 8.6, 2.8 Hz, 1H), 6.81 (d, J = 2.8 Hz, 1H). ^{13}C NMR (126 MHz, MeOD) δ 167.5, 158.0, 151.5, 148.5, 140.5, 139.3, 139.1, 134.6, 134.1, 131.7, 130.1, 129.4, 126.2, 124.0, 117.8, 116.3.

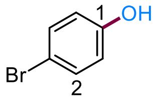

4-Bromophenol (59). The title compound was obtained by following the **General Procedure B** and known in the literature.[29] Two regioisomers (C1:C2 = 3.5:1) were formed as determined by ^{1}H NMR analysis of the crude reaction mixture. Colorless oil, yield = 72% (45 mg), current = 61 mA, electricity = 2.1 F mol^{-1}. **C1** and **C2** were separated by chromatography. **C1:** ^{1}H NMR (500 MHz, $CDCl_3$) δ 7.38 – 7.30 (m, 2H), 6.77 – 6.70 (m, 2H), 5.19 (s, 1H). ^{13}C NMR (126 MHz, $CDCl_3$) δ 154.8, 132.6, 117.4, 113.0. **C2:** ^{1}H NMR (500 MHz, $CDCl_3$) δ 7.47 (dd, J = 8.0, 1.5 Hz, 1H), 7.27 – 7.19 (m, 1H), 7.04 (dd, J = 8.2, 1.5 Hz, 1H), 6.86 – 6.79 (m, 1H). ^{13}C NMR (126 MHz, $CDCl_3$) δ 152.4, 132.2, 129.4, 122.0, 116.3, 110.4.

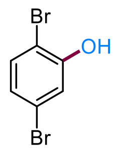

2,5-Dibromophenol (60). The title compound was obtained by following the **General Procedure B** and known in the literature.[30] White solid, yield = 71% (65 mg), current = 64 mA, electricity = 2.2 F mol^{-1}. ^{1}H NMR (500 MHz, $CDCl_3$) δ 7.32 (d, J = 8.5 Hz, 1H), 7.20 (d, J = 2.3 Hz, 1H), 6.96 (dd, J = 8.5, 2.3 Hz, 1H), 5.59 (s, 1H). ^{13}C NMR (126 MHz, $CDCl_3$) δ 153.2, 133.0, 125.1, 122.3, 119.6, 109.2.

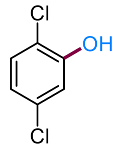

2,5-Dichlorophenol (61). The title compound was obtained by following the **General Procedure B** and known in the literature.[31] White solid, yield = 65% (38 mg), current = 64 mA, electricity = 2.2 F mol^{-1}. ^{1}H NMR (500 MHz, $CDCl_3$) δ 7.24 (d, J = 8.6 Hz, 1H), 7.05 (d, J = 2.4 Hz, 1H), 6.87 (dd, J = 8.6, 2.4 Hz, 1H), 5.68 (s, 1H). ^{13}C NMR (126 MHz, $CDCl_3$) δ 152.1, 133.9, 129.7, 121.8, 118.5, 116.9.

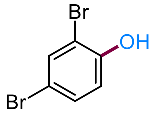

2,4-Dibromophenol (62). The title compound was obtained by following the **General Procedure B** and known in the literature.[32] White solid, yield = 70% (63 mg), current = 64 mA, electricity = 2.2 F mol^{-1}. ^{1}H NMR (500 MHz, $CDCl_3$) δ 7.60 (d, J = 2.3 Hz, 1H), 7.33 (dd, J = 8.7, 2.3 Hz, 1H), 6.92 (d, J = 8.7 Hz, 1H), 5.53 (s, 1H). ^{13}C NMR (126 MHz, $CDCl_3$) δ 151.8, 134.2, 132.3, 117.6, 112.8, 111.0.

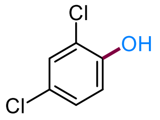

2,4-Dichlorophenol (63). The title compound was obtained by following the **General Procedure B** and known in the literature.[33] White solid, yield = 58% (34 mg), current = 64 mA, electricity = 2.2 F mol^{-1}. ^{1}H NMR (500 MHz, $CDCl_3$) δ 7.33 (d, J = 2.5 Hz, 1H), 7.16 (dd, J = 8.7, 2.4 Hz, 1H), 6.96 (d, J = 8.7 Hz, 1H), 5.53 (s, 1H). ^{13}C NMR (126 MHz, $CDCl_3$) δ 150.4, 128.8, 128.7, 125.8, 120.6, 117.3.

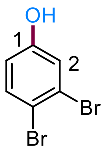

3,4-Dibromophenol (64). The title compound was obtained by following the **General Procedure B** and known in the literature.[34] Two regioisomers (C1:C2 = 7.6:1) were formed as determined by ^{1}H NMR analysis of the crude reaction mixture.White solid, yield = 74% (67 mg), current = 67 mA, electricity = 2.3 F mol^{-1}. Major isomer **C1:** ^{1}H NMR (500 MHz, $CDCl_3$) δ 7.45 (d, J = 8.7 Hz, 1H), 7.15 (d, J = 2.9 Hz, 1H), 6.69 (dd, J = 8.7, 2.9 Hz, 1H), 5.43 (s, 1H). ^{13}C NMR (126 MHz, $CDCl_3$) δ 155.4, 134.2, 125.1, 120.9, 116.4, 115.5. Minor isomer **C2:** ^{1}H NMR (500 MHz, $CDCl_3$) δ 7.21 (dd, J = 7.9, 1.5 Hz, 1H), 7.11 (dd, J = 8.1 Hz, 8.0 Hz 1H), 6.97 (dd, J = 8.3, 1.4 Hz, 1H), 5.72 (s, 1H).

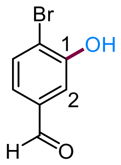

4-Bromo-3-hydroxybenzaldehyde (65). The title compound was obtained by following the **General Procedure B** and known in the literature.[35] Two regioisomers (C1:C2 = 2.8:1) were formed as determined by ^{1}H NMR analysis of the crude reaction mixture. White solid, yield = 42% (30 mg), current = 93 mA, electricity = 3.2 F mol^{-1}. **C1** and **C2** were separated by chromatography. **C1:** ^{1}H NMR (500 MHz, $CDCl_3$) δ 9.94 (s, 1H), 7.67 (d, J = 8.1 Hz, 1H), 7.51 (d, J = 1.9 Hz, 1H), 7.34 (dd, J = 8.2, 1.9 Hz, 1H), 5.98 (s, 1H). ^{13}C NMR (126 MHz, $CDCl_3$) δ 191.3, 153.3, 137.5, 133.2, 122.9, 117.6, 116.5. **C2:** ^{1}H NMR (500 MHz, $CDCl_3$) δ 11.13 (s, 1H), 9.87 (s, 1H), 7.42 (d, J = 8.2 Hz, 1H), 7.21 (d, J = 1.7 Hz, 1H), 7.18 (dd, J = 8.2, 1.8 Hz, 1H). ^{13}C NMR (126 MHz, $CDCl_3$) δ 196.0, 162.2, 134.7, 132.2, 123.7, 121.3, 119.7.

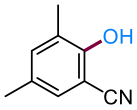

2-Hydroxy-3,5-dimethylbenzonitrile (66). The title compound was obtained by following the **General Procedure B** and known in the literature.[36] White solid, yield = 60% (32 mg), current = 64 mA, electricity = 2.2 F mol^{-1}. ^{1}H NMR (500 MHz, $CDCl_3$) δ 7.16 (d, J = 2.1 Hz, 1H), 7.11 (d, J = 2.1 Hz, 1H), 6.15 (s, 1H), 2.25 (s, 3H), 2.25 (s, 3H). ^{13}C NMR (126 MHz, $CDCl_3$) δ 154.9, 137.2, 130.4, 129.8, 126.0, 117.1, 98.7, 20.3, 16.0.

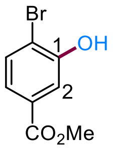

Methyl 4-bromo-3-hydroxybenzoate (67). The title compound was obtained by following the **General Procedure B** and known in the literature.[37] Two regioisomers (C1:C2 = 4.6:1) were formed as determined by ^{1}H NMR analysis of the crude reaction mixture. White solid, yield = 77% (64 mg), current = 93 mA, electricity = 3.2 F mol^{-1}. **C1** and **C2** were separated by chromatography. **C1:** ^{1}H NMR (500 MHz, CDCl$_3$) δ 7.69 (d, *J* = 2.0 Hz, 1H), 7.54 (d, *J* = 8.3 Hz, 1H), 7.48 (dd, *J* = 8.4, 2.0 Hz, 1H), 5.97 (s, 1H), 3.92 (s, 3H). ^{13}C NMR (126 MHz, CDCl$_3$) δ 166.5, 152.7, 132.4, 131.3, 122.8, 117.3, 115.8, 52.6. **C2:** ^{1}H NMR (500 MHz, CDCl$_3$) δ 10.82 (s, 1H), 7.68 (d, *J* = 8.5 Hz, 1H), 7.18 (d, *J* = 2.0 Hz, 1H), 7.02 (dd, *J* = 8.5, 1.9 Hz, 1H), 3.95 (s, 3H). ^{13}C NMR (126 MHz, CDCl$_3$) δ 170.3, 162.2, 131.1, 130.2, 122.9, 121.1, 111.6, 52.7.

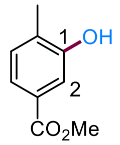

Methyl 3-hydroxy-4-methylbenzoate (68). The title compound was obtained by following the **General Procedure B** and known in the literature.[38] Two regioisomers (C1:C2 = 3.5:1) were formed as determined by ^{1}H NMR analysis of the crude reaction mixture. White solid, yield = 56% (33 mg), current = 61 mA, electricity = 2.1 F mol^{-1}. **C1** and **C2** were separated by chromatography. **C1:** ^{1}H NMR (500 MHz, CDCl$_3$) δ 7.60 (d, J = 1.6 Hz, 1H), 7.52 (dd, J = 7.8, 1.7 Hz, 1H), 7.18 (d, J = 7.8 Hz, 1H), 6.11 (s, 1H), 3.92 (s, 3H), 2.31 (s, 3H). ^{13}C NMR (126 MHz, CDCl$_3$) δ 167.8, 154.3, 131.1, 130.6, 128.9, 121.9, 116.0, 52.4, 16.3. **C2:** ^{1}H NMR (500 MHz, CDCl$_3$) δ 10.71 (s, 1H), 7.72 (d, *J* = 8.1 Hz, 1H), 6.80 (s, 1H), 6.73 – 6.68 (m, 1H), 3.94 (s, 3H), 2.35 (s, 3H). ^{13}C NMR (126 MHz, CDCl$_3$) δ 170.8, 161.7, 147.2, 129.9, 120.6, 117.9, 110.0, 52.3, 22.0.

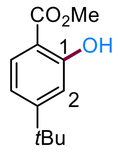

Methyl 4-(*tert*-butyl)-2-hydroxybenzoate (69). The title compound was obtained by following the **General Procedure B** and known in the literature.[39] Two regioisomers (C1:C2 = 1.6:1) were formed as determined by ^{1}H NMR analysis of the crude reaction mixture. White solid, yield = 78% (58 mg), current = 81 mA, electricity = 2.8 F mol^{-1}. **C1** and **C2** were separated by chromatography. **C1:** ^{1}H NMR (500 MHz, CDCl$_3$) δ 10.69 (s, 1H), 7.75 (d, *J* = 8.4 Hz, 1H), 7.00 (d, *J* = 1.8 Hz, 1H), 6.93 (dd, *J* = 8.4, 1.9 Hz, 1H), 3.94 (s, 3H), 1.31 (s, 9H). ^{13}C NMR (126 MHz, CDCl$_3$) δ 170.7, 161.6, 160.3, 129.6, 117.0, 114.5, 109.9, 52.3, 35.4, 31.0. **C2:** ^{1}H NMR (500 MHz, CDCl$_3$) δ 7.56 (d, *J* = 1.7 Hz, 1H), 7.53 (dd, *J* = 8.1, 1.8 Hz, 1H), 7.33 (d, *J* = 8.1 Hz, 1H), 6.01 (s, 1H), 3.92 (s, 3H), 1.43 (s, 9H). ^{13}C NMR (126 MHz, CDCl$_3$) δ 167.7, 154.8, 142.2, 128.8, 127.3, 121.8, 117.6, 52.4, 35.2, 29.4.

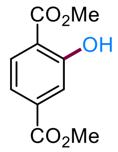

Dimethyl 2-hydroxyterephthalate (70). The electrolysis was conducted using a modified procedure in DCM (0.045 M) in the presence of TFA (2.16 mmol, 6.0 equiv) and 2,6-lutidine (0.72 mmol, 2.0 equiv). The title compound is known in the literature.[40] White solid, yield = 45% (34 mg), current = 173 mA, electricity = 8.0 F mol^{-1}. ^{1}H NMR (500 MHz, $CDCl_3$) δ 10.74 (s, 1H), 7.89 (d, *J* = 8.3 Hz, 1H), 7.62 (d, *J* = 1.6 Hz, 1H), 7.51 (dd, *J* = 8.3, 1.6 Hz, 1H), 3.97 (s, 3H), 3.92 (s, 3H). ^{13}C NMR (126 MHz, $CDCl_3$) δ 170.1, 166.1, 161.5, 136.6, 130.2, 119.8, 119.1, 115.8, 52.8, 52.6.

5. Synthesis and characterization of new substrates.

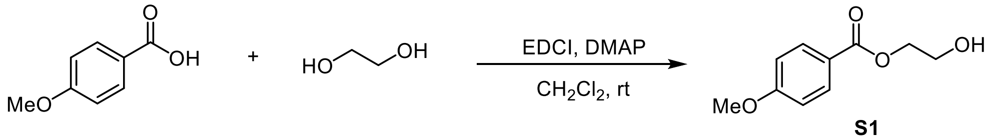

S1

2-Hydroxyethyl 4-methoxybenzoate (S1). To an oven-dried round bottom flask was added 4-methoxybenzoic acid (3.1 g, 20 mmol, 1.0 equiv), EDCI (5.7 g, 30 mmol, 1.5 equiv), DMAP (0.50 g, 4.0 mmol, 20 mol%), ethane-1,2-diol (1.7 mL, 30 mmol, 1.5 equiv) and CH_2Cl_2 (80 mL). The mixture was stirred at rt for 8 h. The resulting mixture was stirred until complete consumption of 4-methoxybenzoic acid and then diluted with H_2O and CH_2Cl_2. The combined organic phase was concentrated under reduced pressure. The residue was purified by column chromatography to afford **S1** (2.7 g, 69% yield) as colorless oil. ^1H NMR (500 MHz, $CDCl_3$) δ 7.99 (d, J = 8.9 Hz, 2H), 6.90 (d, J = 8.9 Hz, 2H), 4.44 – 4.36 (m, 2H), 3.96 – 3.89 (m, 2H), 3.84 (s, 3H), 2.54 (s, 1H). ^{13}C NMR (126 MHz, $CDCl_3$) δ 166.9, 163.6, 131.9, 122.3, 113.8, 66.5, 61.5, 55.6. IR (neat, cm^{-1}): 3439, 1707, 1649, 1100, 647. ESI HRMS m/z $(M+Na)^+$ calcd 219.0626, obsd 219.0626.

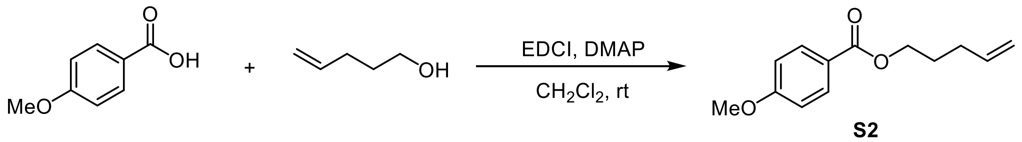

S2

Pent-4-en-1-yl 4-methoxybenzoate (S2). To an oven-dried round bottom flask was added 4-methoxybenzoic acid (1.4 g, 9.0 mmol, 1.5 equiv), EDCI (2.1 g, 12 mmol, 2.0 equiv), DMAP (0.28 g, 1.2 mmol, 20 mol%), pent-4-en-1-ol (0.62 mL, 6.0 mmol, 1.0 equiv) and CH_2Cl_2 (20 mL). The mixture was stirred at rt. The resulting mixture was stirred until complete consumption of pent-4-en-1-ol and then concentrated under reduced pressure. The residue was purified by column chromatography to afford **S2** (1.0 g, 75% yield) as colorless oil. ^1H NMR (500 MHz, $CDCl_3$) δ 8.01 (d, J = 8.9 Hz, 2H), 6.92 (d, J = 8.9 Hz, 2H), 5.93 – 5.77 (m, 1H), 5.15 – 5.04 (m, 1H), 5.03 – 4.97 (m, 1H), 4.31 (t, J = 6.6 Hz, 2H), 3.86 (s, 3H), 2.27 – 2.18 (m, 2H), 1.91 – 1.83 (m, 2H). ^{13}C NMR (126 MHz, $CDCl_3$) δ 166.5, 163.5, 137.7, 131.7, 123.1, 115.4, 113.7, 113.7, 64.2, 55.6, 30.4, 28.2. IR (neat, cm^{-1}): 3360, 2919, 2849, 1719, 1512, 1278, 1026, 762. ESI HRMS m/z $(M+Na)^+$ calcd 243.0992, obsd 243.0996.

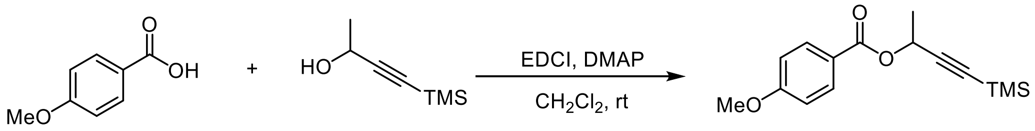

S3

4-(Trimethylsilyl)but-3-yn-2-yl 4-methoxybenzoate (S3). To an oven-dried round bottom flask was added 4-methoxybenzoic acid (1.4 g, 9.0 mmol, 1.5 equiv), EDCI (2.1 g, 12 mmol, 2.0 equiv), DMAP (0.28 g, 1.2 mmol, 20 mol%), 4-(trimethylsilyl)but-3-yn-2-ol (1.0 mL, 6.0 mmol, 1.0 equiv) and CH_2Cl_2 (20 mL). The mixture was stirred at rt The resulting mixture was

stirred until complete consumption of 4-(trimethylsilyl)but-3-yn-2-ol and then concentrated under reduced pressure. The residue was purified by column chromatography to afford **S3** (1.4 g, 85% yield) as colorless oil. ^{1}H NMR (500 MHz, $CDCl_3$) δ 8.02 (d, *J* = 8.9 Hz, 2H), 6.91 (d, *J* = 8.8 Hz, 2H), 5.70 (q, *J* = 6.7 Hz, 1H), 3.84 (s, 3H), 1.59 (d, *J* = 6.7 Hz, 3H), 0.18 (s, 9H). ^{13}C NMR (126 MHz, $CDCl_3$) δ 165.2, 163.6, 131.9, 122.5, 113.7, 104.0, 89.5, 60.9, 55.5, 21.8, -0.1. IR (neat, cm^{-1}): 3251, 2926, 1655, 1494, 1208, 1001. ESI HRMS *m/z* $(M+Na)^+$ calcd 441.0191, obsd 441.0201. IR (neat, cm^{-1}): 3251, 2926, 1655, 1494, 1208, 1001. ESI HRMS *m/z* $(M+Na)^+$ calcd 299.1074, obsd 299.1075.

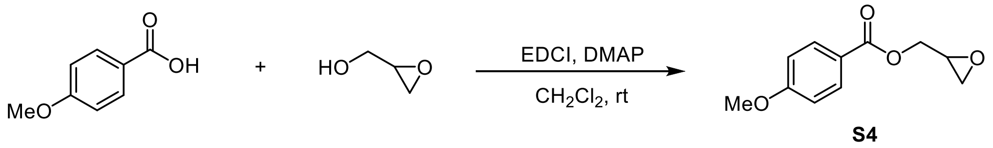

Oxiran-2-ylmethyl 4-methoxybenzoate (S4). To an oven-dried round bottom flask was added 4-methoxybenzoic acid (1.4 g, 9.0 mmol, 1.5 equiv), EDCI (2.1 g, 12 mmol, 2.0 equiv), DMAP (0.28 g, 1.2 mmol, 20 mol%), oxiran-2-ylmethanol (0.55 g, 6.0 mmol, 1.0 equiv) and CH_2Cl_2 (20 mL). The mixture was stirred at RT for 12 h. The resulting mixture was stirred until complete consumption of oxiran-2-ylmethanol and then concentrated under reduced pressure. The residue was purified by column chromatography to afford **S4** (0.85 g, 68% yield) as colorless oil. ^{1}H NMR (500 MHz, $CDCl_3$) δ 8.02 (d, *J* = 8.9 Hz, 2H), 6.92 (d, *J* = 9.0 Hz, 2H), 4.62 (dd, *J* = 12.3, 3.1 Hz, 1H), 4.14 (dd, *J* = 12.3, 6.2 Hz, 1H), 3.86 (s, 3H), 3.44 – 3.27 (m, 1H), 2.89 (t, *J* = 4.5 Hz, 1H), 2.72 (dd, *J* = 4.9, 2.6 Hz, 1H). ^{13}C NMR (126 MHz, $CDCl_3$) δ 166.1, 163.7, 131.9, 122.2, 113.8, 65.3, 55.6, 49.7, 44.8. IR (neat, cm^{-1}): 3404, 2925, 2852, 1719, 1583, 1418, 1009. ESI HRMS *m/z* $(M+Na)^+$ calcd 231.0628, obsd 231.0626.

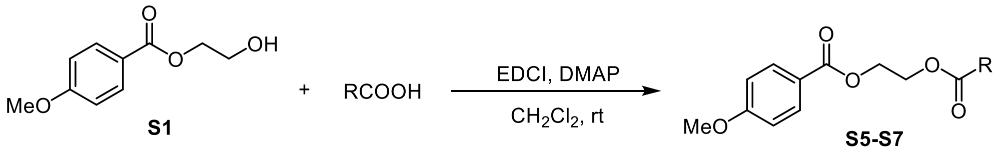

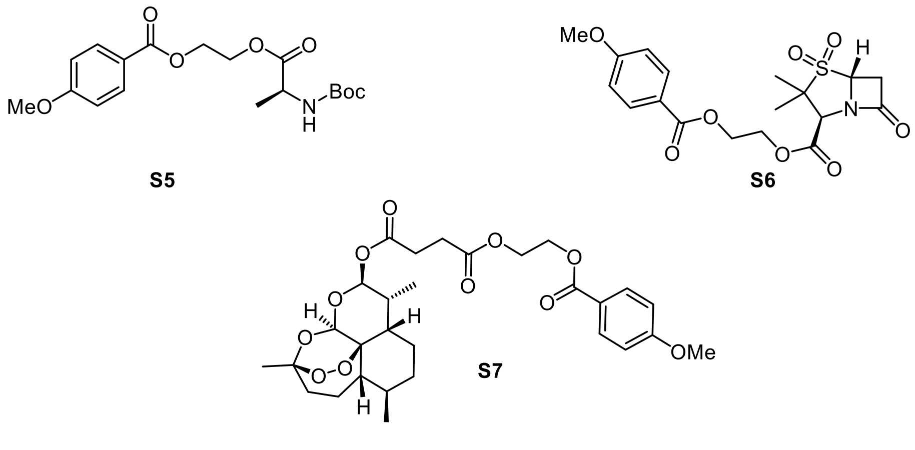

2-(((*tert*-Butoxycarbonyl)-*L*-alanyl)oxy)ethyl 4-methoxybenzoate (S5). To an oven-dried round bottom flask was added (*tert*-butoxycarbonyl)-*L*-alanine (1.7 g, 9.0 mmol, 1.5 equiv), EDCI (2.1 g, 12 mmol, 2.0 equiv), DMAP (0.15 g, 1.2 mmol, 20 mol%), **S1** (1.2 g, 6.0 mmol, 1.0 equiv) and CH_2Cl_2 (20 mL). The mixture was stirred at rt for 12 h. The resulting mixture was stirred until complete consumption of **S1** and then concentrated under reduced pressure. The residue was purified by column chromatography to afford **S5** (1.7 g, 77% yield) as colorless oil. ^{1}H NMR (500 MHz, $CDCl_3$) δ 7.95 (d, *J* = 8.9 Hz, 2H), 6.88 (d, *J* = 8.9 Hz, 2H), 5.14 (d, *J* = 8.0 Hz, 1H), 4.54 – 4.44 (m, 3H), 4.42 – 4.36 (m, 1H), 4.35 – 4.14 (m, 1H), 3.82 (s, 3H), 1.39 (s, 9H), 1.35 (d, *J* = 7.2 Hz, 3H). ^{13}C NMR (126 MHz, $CDCl_3$) δ 173.3, 166.0, 163.6, 155.2, 131.8, 122.1, 113.7, 79.8, 63.0, 62.3, 55.5, 49.3, 28.4, 18.6. IR (neat, cm^{-1}): 3395, 2073, 1645, 1217, 689. ESI HRMS *m/z* $(M+Na)^+$ calcd 390.1523, obsd 390.1527.

2-((4-Methoxybenzoyl)oxy)ethyl (2*S*,5*R*)-3,3-dimethyl-7-oxo-4-thia-1-azabicyclo[3.2.0]heptane-2-carboxylate 4,4-dioxide (S6). To an oven-dried round bottom flask was added sulbactam (2.1 g, 9.0 mmol, 1.5 equiv), EDCI (2.1 g, 12 mmol, 2.0 equiv), DMAP (0.15 g, 1.2 mmol, 20 mol%), **S1** (1.2 g, 6.0 mmol, 1.0 equiv) and CH_2Cl_2 (20 mL). The mixture was stirred at rt for 12 h. The resulting mixture was stirred until complete consumption of **S1** and then concentrated under reduced pressure. The residue was purified by column chromatography to afford **S6** (2.0 g, 81% yield) as colorless oil. ^{1}H NMR (500 MHz, $CDCl_3$) δ 7.96 (d, *J* = 8.6 Hz, 2H), 6.92 (d, *J* = 8.7 Hz, 2H), 4.64 – 4.57 (m, 2H), 4.57 – 4.50 (m, 2H), 4.50 – 4.43 (m, 1H), 4.42 (s, 1H), 3.86 (s, 3H), 3.52 – 3.38 (m, 2H), 1.57 (s, 3H), 1.38 (s, 3H). ^{13}C NMR (126 MHz, $CDCl_3$) δ 170.8, 167.0, 165.9, 163.9, 131.8, 121.8, 114.0, 64.2, 63.3, 62.8, 61.9, 61.2, 55.6, 38.4, 20.4, 18.6. IR (neat, cm^{-1}): 3399, 2130, 1703, 1512, 1094, 757. ESI HRMS *m/z* $(M+Na)^+$ calcd 434.0880, obsd 434.0885.

2-((4-Methoxybenzoyl)oxy)ethyl ((3*R*,5a*S*,6*R*,8a*S*,9*R*,10*S*,12*R*,12a*R*)-3,6,9-trimethyldecahydro-12*H*-3,12-epoxy[1,2]dioxepino[4,3-*i*]isochromen-10-yl) succinate (S7). To an oven-dried round bottom flask was added artesunate (3.5 g, 9.0 mmol, 1.5 equiv), EDCI (2.1 g, 12 mmol, 2.0 equiv), DMAP (0.15 g, 1.2 mmol, 20 mol%), **S1** (1.2 g, 6.0 mmol, 1.0 equiv) and CH_2Cl_2 (20 mL). The mixture was stirred at rt for 12 h. The resulting mixture was stirred until complete consumption of **S1** and then concentrated under reduced pressure. The residue was purified by column chromatography to afford **S7** (2.2 g, 66% yield) as white solid. ^{1}H NMR (500 MHz, $CDCl_3$) δ 7.95 (d, *J* = 8.9 Hz, 2H), 6.88 (d, *J* = 8.9 Hz, 2H), 5.72 (d, *J* = 9.8 Hz, 1H), 5.36 (s, 1H), 4.45 – 4.40 (m, 2H), 4.40 – 4.34 (m, 2H), 3.81 (s, 3H), 2.76 – 2.61 (m, 4H), 2.53 – 2.48 (m, 1H), 2.34 – 2.28 (m, 1H), 1.99 – 1.95 (m, 1H), 1.86 – 1.81 (m, 1H), 1.70 – 1.61 (m, 2H), 1.57 – 1.51 (m, 1H), 1.47 – 1.40 (m, 1H), 1.37 (s, 3H), 1.29 – 1.20 (m, 3H), 0.99 – 0.93 (m, 1H), 0.91 (d, *J* = 6.0 Hz, 3H), 0.78 (d, *J* = 7.1 Hz, 3H). ^{13}C NMR (126 MHz, $CDCl_3$) δ 171.9, 171.0, 166.0, 163.5, 131.8, 122.2, 113.7, 104.4, 92.2, 91.5, 80.1, 62.6, 62.3, 55.4, 51.5, 45.2, 37.2, 36.2, 34.1, 31.8, 29.2, 28.8, 25.9, 24.6, 21.9, 20.2, 12.0. IR (neat,

cm^{-1}): 3431, 2958, 1713, 1512, 1282, 1128, 763. ESI HRMS *m/z* $(M+Na)^+$ calcd 585.2306, obsd 585.2307.

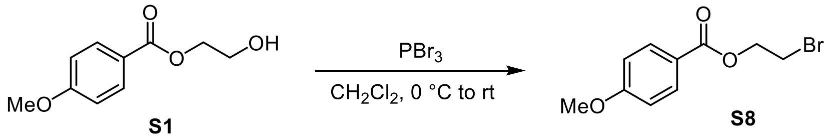

2-Bromoethyl 4-methoxybenzoate (S8). To an oven-dried round bottom flask was added **S1** (0.60 g, 3.0 mmol, 1.0 equiv) and CH_2Cl_2 (10 mL). The mixture was cooled to 0 °C and PBr_3 (0.4 mL, 3.3 mmol, 1.1 equiv) was added slowly. The resulting mixture was stirred at rt until complete consumption of **S1** and then quenched with H_2O. The mixture was diluted with H_2O and CH_2Cl_2. The combined organic phase was concentrated under reduced pressure. The residue was purified by column chromatography to afford **S8** (0.58 g, 75% yield) as pale yellow oil. ^{1}H NMR (500 MHz, $CDCl_3$) δ 8.03 (d, *J* = 9.0 Hz, 2H), 6.94 (d, *J* = 9.0 Hz, 2H), 4.60 (t, *J* = 6.2 Hz, 2H), 3.87 (s, 3H), 3.64 (t, *J* = 6.1 Hz, 2H). ^{13}C NMR (126 MHz, $CDCl_3$) δ 165.9, 163.8, 132.0, 122.2, 113.9, 64.1, 55.6, 29.1.

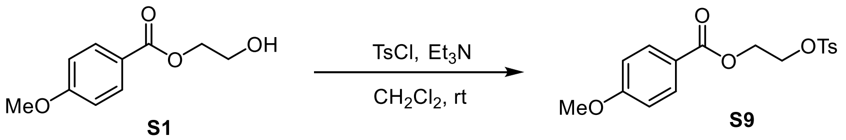

2-(Tosyloxy)ethyl 4-methoxybenzoate (S9). To an oven-dried round bottom flask was added **S1** (0.80 g, 4.0 mmol, 1.0 equiv), Et_3N (1.1 mL, 8.0 mmol, 2.0 equiv) and CH_2Cl_2 (10 mL). TsCl (1.1 g, 6.0 mmol, 1.5 equiv) was added slowly. The mixture was stirred at rt until complete consumption of **S1** and then diluted with H_2O and CH_2Cl_2. The combined organic phase was concentrated under reduced pressure. The residue was purified by column chromatography to afford **S1** (1.2 g, 88% yield) as white solid. ^{1}H NMR (500 MHz, $CDCl_3$) δ 7.89 (d, *J* = 8.9 Hz, 2H), 7.78 (d, *J* = 8.4 Hz, 2H), 7.27 (d, *J* = 7.5 Hz, 2H), 6.90 (d, *J* = 8.9 Hz, 2H), 4.49 – 4.41 (m, 2H), 4.39 – 4.31 (m, 2H), 3.86 (s, 3H), 2.39 (s, 3H). ^{13}C NMR (126 MHz, $CDCl_3$) δ 165.9, 163.8, 145.1, 133.0, 131.9, 130.0, 128.0, 121.9, 113.8, 68.0, 61.9, 55.6, 21.8. IR (neat, cm^{-1}): 3416, 2841, 2036, 1616, 1018, 764. ESI HRMS *m/z* $(M+Na)^+$ calcd 373.0716, obsd 373.0718.

6. Limitations

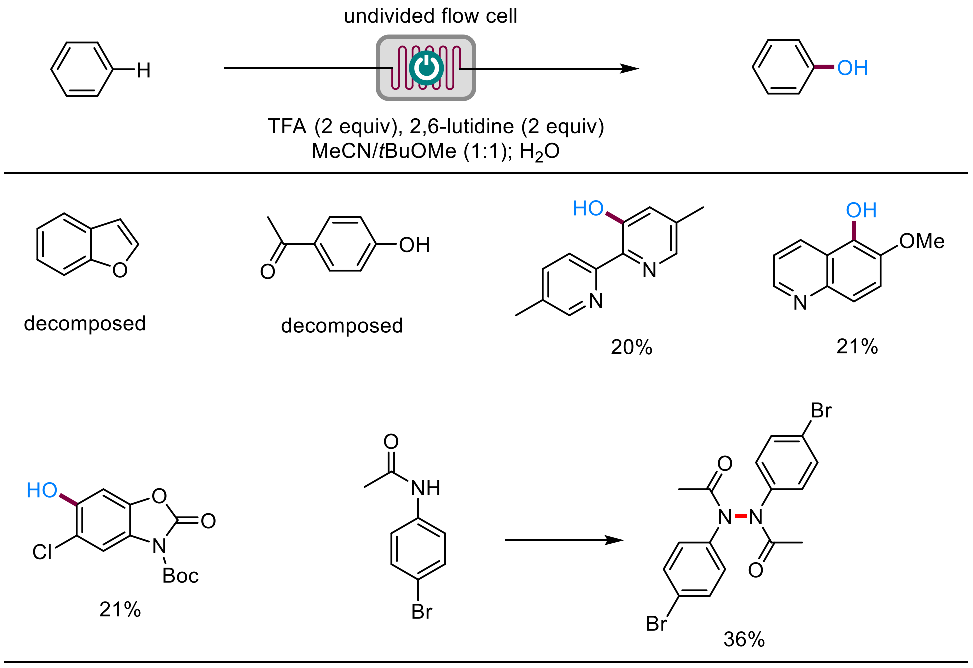

7. Mechanistic studies

7.1 Synthesis of 71

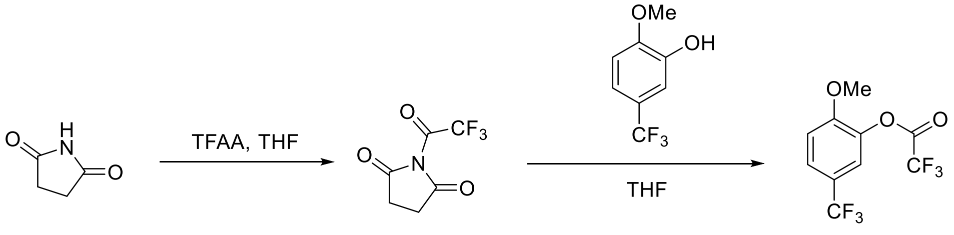

S10 71

1-(2,2,2-Trifluoroacetyl)pyrrolidine-2,5-dione (S10). To a solution of succinimide (0.98 g, 10 mmol) in anhydrous THF (10 mL) was added slowly trifluoroacetic anhydride (3.1 g, 15 mmol) at rt under Ar. The mixture was stirred until no solid was in the solution. After removal of the solvent, excess of anhydride and byproduct TFA, a white solid was obtained, which contained 10% succinimide as determined by ^{1}H NMR.

2-Methoxy-5-(trifluoromethyl)phenyl 2,2,2-trifluoroacetate (71). To an oven-dried round bottom flask was added above mixture and 2-methoxy-5-(trifluoromethyl)phenol. The mixture was stirred for 12 h under Ar. After reaction, the solid precipitated and was filtered off. Then, the solvent was removed to give trifluoroacetate ester **71**, which was not purified due to its sensatitity to hydrolysis. ^{1}H NMR (500 MHz, $CDCl_3$) δ 7.47 (dd, J = 8.7, 2.2 Hz, 1H), 7.32 (d, J = 2.2 Hz, 1H), 7.01 (d, J = 8.7 Hz, 1H), 3.79 (s, 3H).

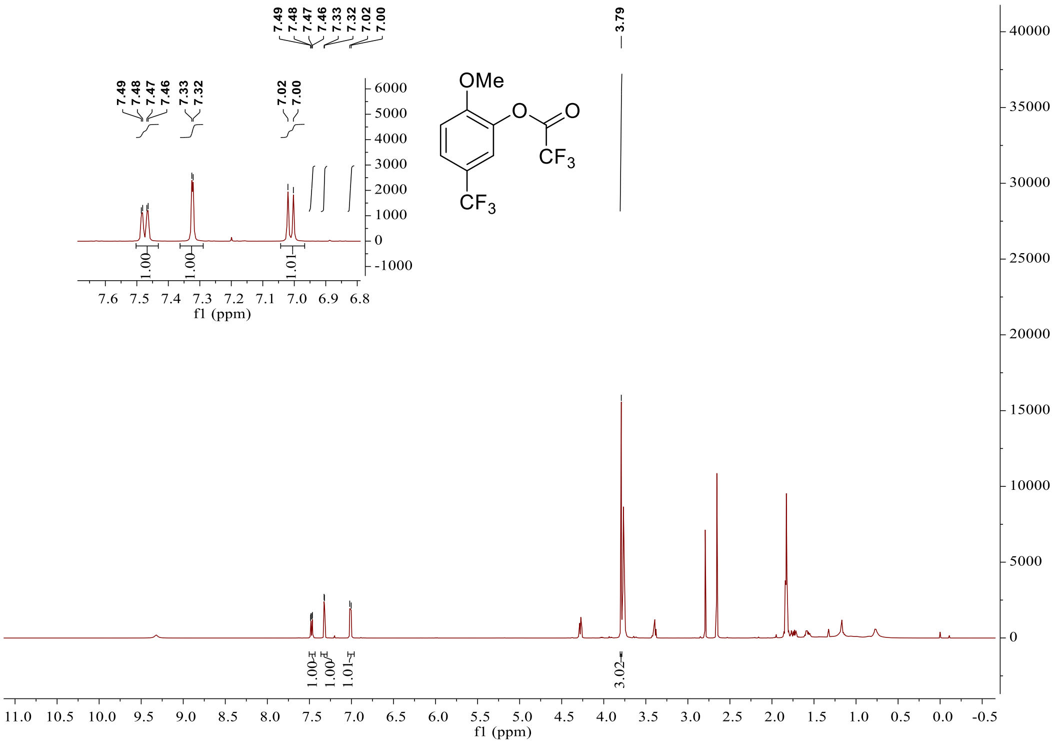

7.2 Investigation of aryl trifluoroacetate intermediate

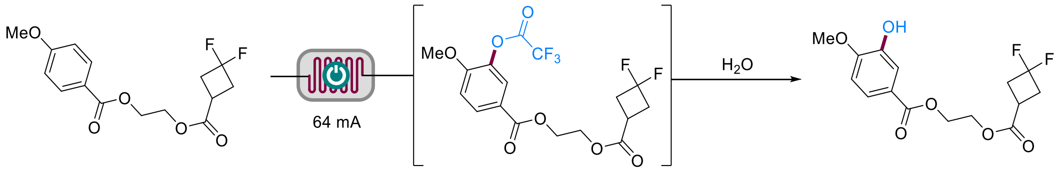

S11 **S12** **S13**

The outlet solution from the electrolytic cell was concentrated under reduced pressure without quenching with aqueous solution. The residue was chromatographed through silica gel eluting with ethyl acetate/hexanes to give a mixture of **S12** and **S13** in a ratio of 2.1:1 (Supplementary Figure 4). In a seperate reaction, the outlet solution was quenched with aqueous $NaNHCO_3$ to give **S13** in 61% yield. **S12**: ^{1}H NMR (500 MHz, $CDCl_3$) δ 8.02 (dd, J = 8.7, 2.1 Hz, 1H), 7.82 (d, J = 2.0 Hz, 1H), 7.06 (d, J = 8.7 Hz, 1H), 4.55 – 4.51 (m, 2H), 4.47 – 4.45 (m, 2H), 3.93 (s, 3H), 3.01 – 2.97 (m, 1H), 2.85 – 2.76 (m, 4H). ^{19}F NMR (471 MHz, $CDCl_3$) δ –74.5, –83.4 (d, J = 194.1 Hz), –96.7 (d, J = 194.1 Hz). **S13:** ^{1}H NMR (500 MHz, $CDCl_3$) δ 7.59 (dd, J = 8.4, 2.1 Hz, 1H), 7.56 (d, J = 2.1 Hz, 1H), 6.87 (d, J = 8.4 Hz, 1H), 5.92 (s, 1H), 4.52 – 4.47 (m, 2H), 4.46 – 4.41 (m, 2H), 3.92 (s, 3H), 3.05 – 2.92 (m, 1H), 2.91 – 2.75 (m, 4H). ^{13}C NMR (126 MHz, $CDCl_3$) δ 173.3 (t, $J_{C\text{-}F}$ = 2.7 Hz), 166.1, 150.9, 145.5, 123.0, 122.9, 118.8 (dd, $J_{C\text{-}F}$ = 283.9, 270.8 Hz), 115.8, 110.1, 63.1, 62.4, 56.1, 38.8 (t, $J_{C\text{-}F}$ = 24.5 Hz), 26.6 (d, $J_{C\text{-}F}$ = 5.5 Hz), 26.5 (d, $J_{C\text{-}F}$ = 5.5 Hz). ^{19}F NMR (471 MHz, $CDCl_3$) δ –83.2 (d, J = 193.9 Hz), –96.9 (d, J = 193.5 Hz). IR (neat, cm^{-1}): 3358, 2983, 2849, 1724, 1655, 1504, 1287, 1101, 757. ESI HRMS m/z $(M+Na)^+$ calcd 353.0807, obsd 353.0812.

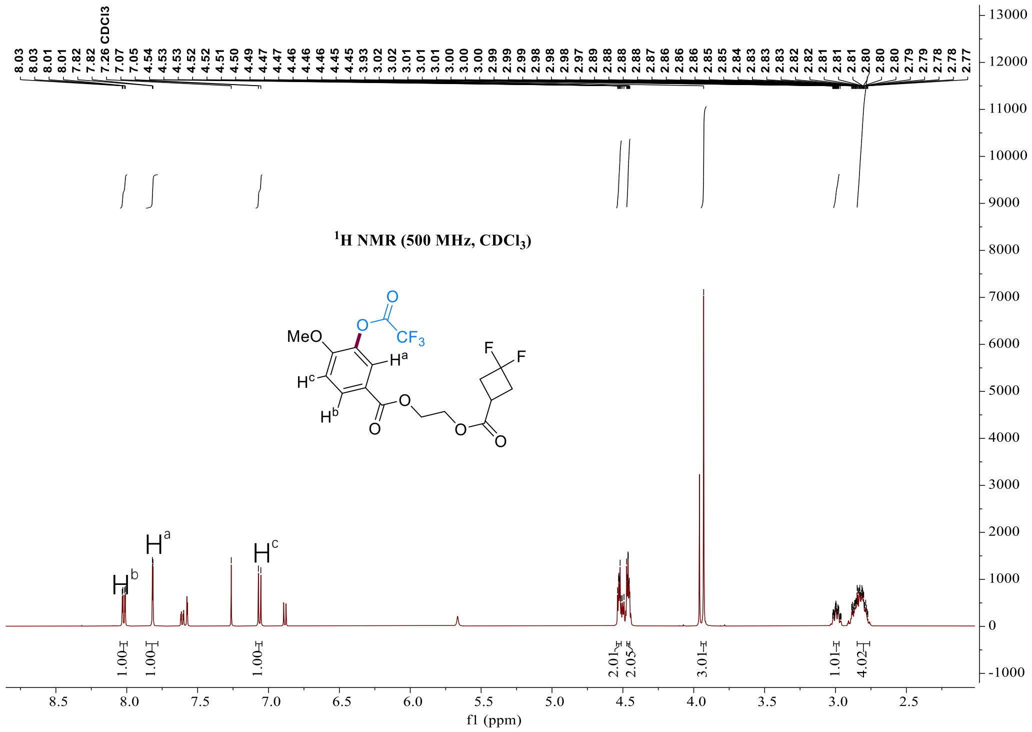

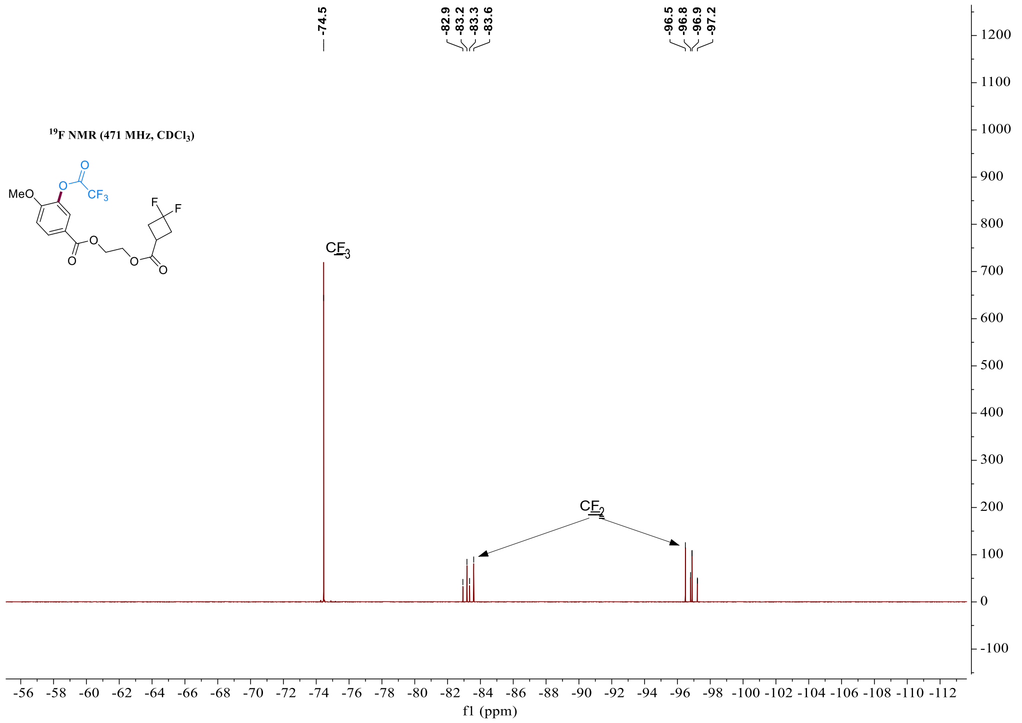

Supplementary Fig. 4. ^{1}H NMR and ^{19}F NMR of a mixture of **S12** and **S13**.

7.3 Cyclic voltammetry studies

Cyclic voltammograms were obtained on a CHI 660E potentiostat. The cyclic voltammograms were recorded in a solution of $n\text{Bu}_4\text{NPF}_6$ (0.1 M) in MeCN using a glassy carbon disk working electrode (diameter, 1 mm), a Pt wire auxiliary electrode and a SCE reference electrode. The scan rate was 100 mV/s.

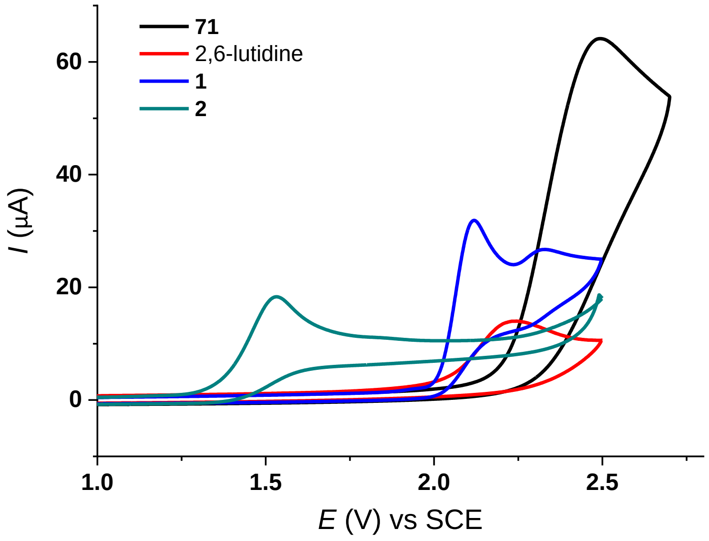

Supplementary Fig. 5. Cyclic voltammograms. Black, compound **71** (6 mM). Red, 2,6-lutidine (6 mM). Blue, compound **1** (3 mM). Green, compound **2** (3 mM).

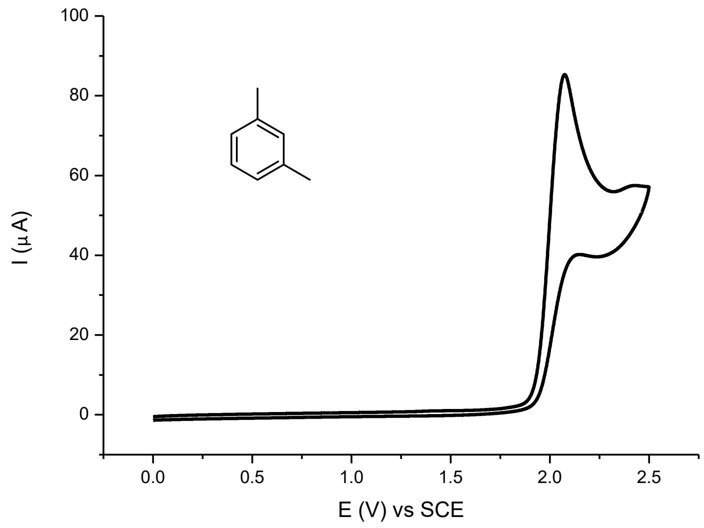

Supplementary Fig. 6. Cyclic voltammogram of *m*-xylene (6 mM). $E_{p/2}$ = 2.07 V.

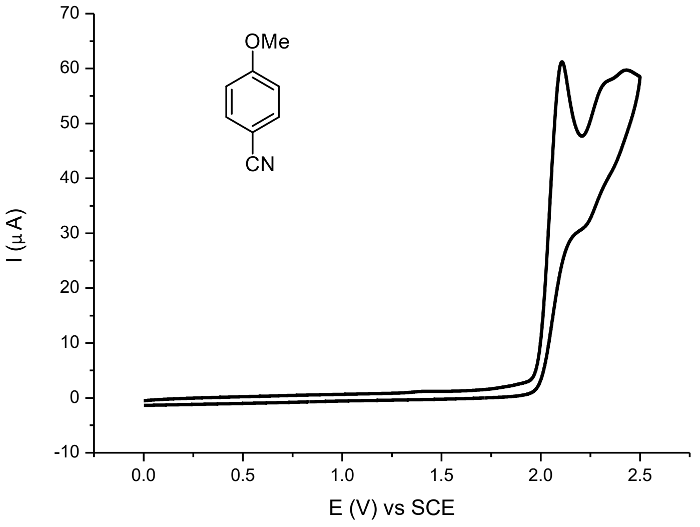

Supplementary Fig. 7. Cyclic voltammogram of 4-methoxybenzonitrile (6 mM). $E_{p/2}$ = 2.10 V.

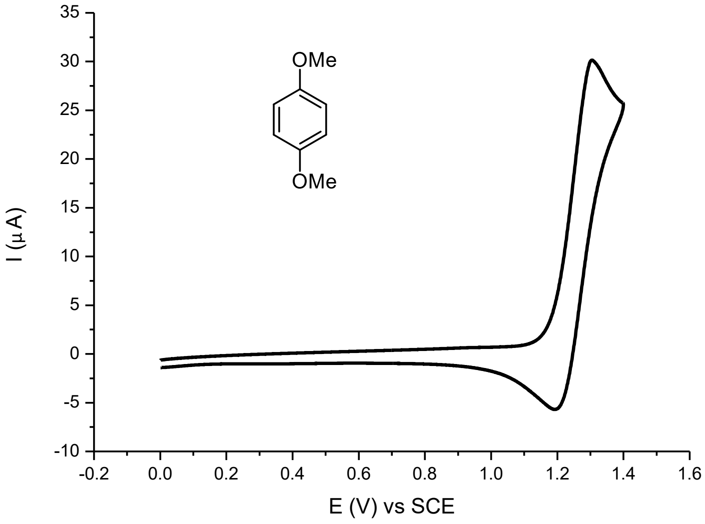

Supplementary Fig. 8. Cyclic voltammogram of 1,4-dimethoxybenzene (6 mM). $E_{p/2}$ = 1.25 V.

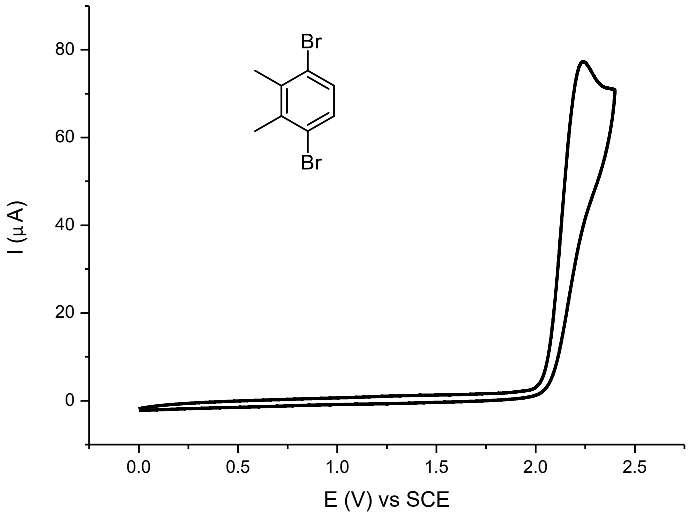

Supplementary Fig. 9. Cyclic voltammogram of 1,4-dibromo-2,3-dimethylbenzene (6 mM). $E_{p/2}$ = 2.13 V.

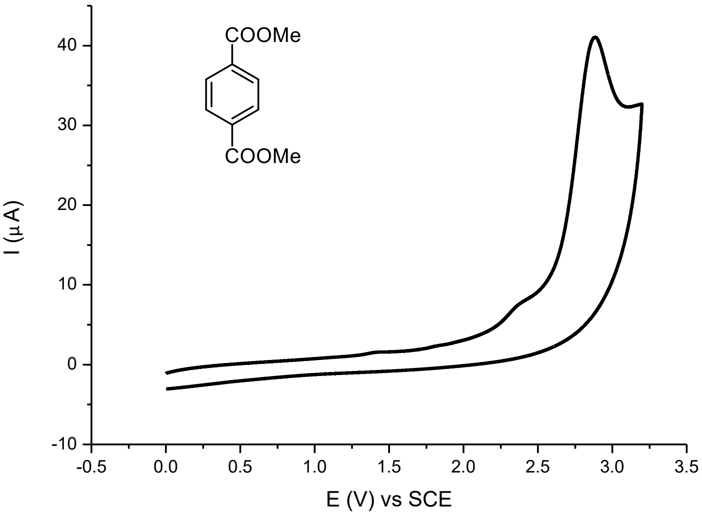

Supplementary Fig. 10. Cyclic voltammogram of dimethyl terephthalate (6 mM). $E_{p/2}$ = 2.70 V.

8. Computational studies

The geometries were optimized by restricted open-shell M062X functional with 6-31G* basis set for all atoms using Gaussian 16 package in MeCN. Nature population analysis was performed to obtain the charge distribution.

Molecular Geometries

B3LYP/6-31G* cartesian coordinates in Å

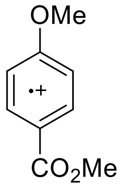

C	-1.399102	-1.036477	0.000156
C	-0.032022	-1.007495	0.000316
C	0.657821	0.236604	0.000252
C	-0.038801	1.466070	0.000032
C	-1.404618	1.463990	-0.000132
C	-2.109752	0.207863	-0.000072
O	-3.401885	0.309811	-0.000239
C	-4.262145	-0.850853	-0.000198
C	2.162965	0.302578	0.000460
O	2.747740	1.354831	-0.000049
O	2.709914	-0.902374	0.000119
C	4.152582	-0.920575	-0.000335
H	-1.935917	-1.977698	0.000196
H	0.545761	-1.924885	0.000494
H	0.536401	2.386027	0.000001
H	-1.996331	2.373341	-0.000308
H	-5.272140	-0.450398	-0.000347
H	-4.080516	-1.437470	-0.902753
H	-4.080703	-1.437271	0.902524
H	4.428899	-1.971646	-0.000521
H	4.523143	-0.414101	-0.892552
H	4.523694	-0.414267	0.891748

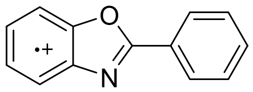

C	4.091731	-0.733649	-0.000001
C	4.139604	0.693586	-0.000013
C	2.997170	1.450091	-0.000018
C	1.765317	0.739181	-0.000011
C	1.761594	-0.691948	0.000002
C	2.898385	-1.462835	0.000008
N	0.502090	1.183565	-0.000015
C	-0.250055	0.078905	-0.000002
O	0.463780	-1.079665	0.000010
C	-1.667320	0.024521	0.000002
C	-2.387735	1.244898	-0.000017
C	-3.767575	1.212399	-0.000013
C	-4.432433	-0.020360	0.000010
C	-3.723692	-1.231643	0.000030
C	-2.344581	-1.218743	0.000025
H	5.030455	-1.278319	0.000003
H	5.110843	1.175061	-0.000019
H	3.004743	2.533822	-0.000028
H	2.877123	-2.545635	0.000018

H	-1.840173	2.181280	-0.000035
H	-4.335481	2.135719	-0.000028
H	-5.517841	-0.040322	0.000014
H	-4.260973	-2.173182	0.000048
H	-1.776020	-2.142465	0.000040

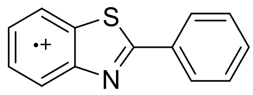

C	-4.303011	-0.216308	0.000000
C	-4.051929	1.184652	0.000001
C	-2.770972	1.662507	0.000000
C	-1.696903	0.721468	-0.000000
C	-1.978612	-0.694932	-0.000000
C	-3.280623	-1.164825	-0.000000
N	-0.405445	1.051061	0.000000
C	0.372448	-0.034831	0.000000
S	-0.489584	-1.574870	-0.000001
C	1.797132	0.050760	0.000000
C	2.611058	-1.109606	-0.000001
C	3.983339	-0.983501	-0.000000
C	4.565461	0.294301	0.000000
C	3.774385	1.448838	0.000001
C	2.398389	1.337225	0.000001
H	-5.332364	-0.560955	0.000001
H	-4.892806	1.868705	0.000001
H	-2.537345	2.721228	0.000001
H	-3.507105	-2.224877	-0.000000
H	2.159757	-2.098050	-0.000001
H	4.612836	-1.866060	-0.000001
H	5.647131	0.386154	0.000000
H	4.241257	2.427274	0.000002
H	1.759134	2.212585	0.000002

9. NMR spectra

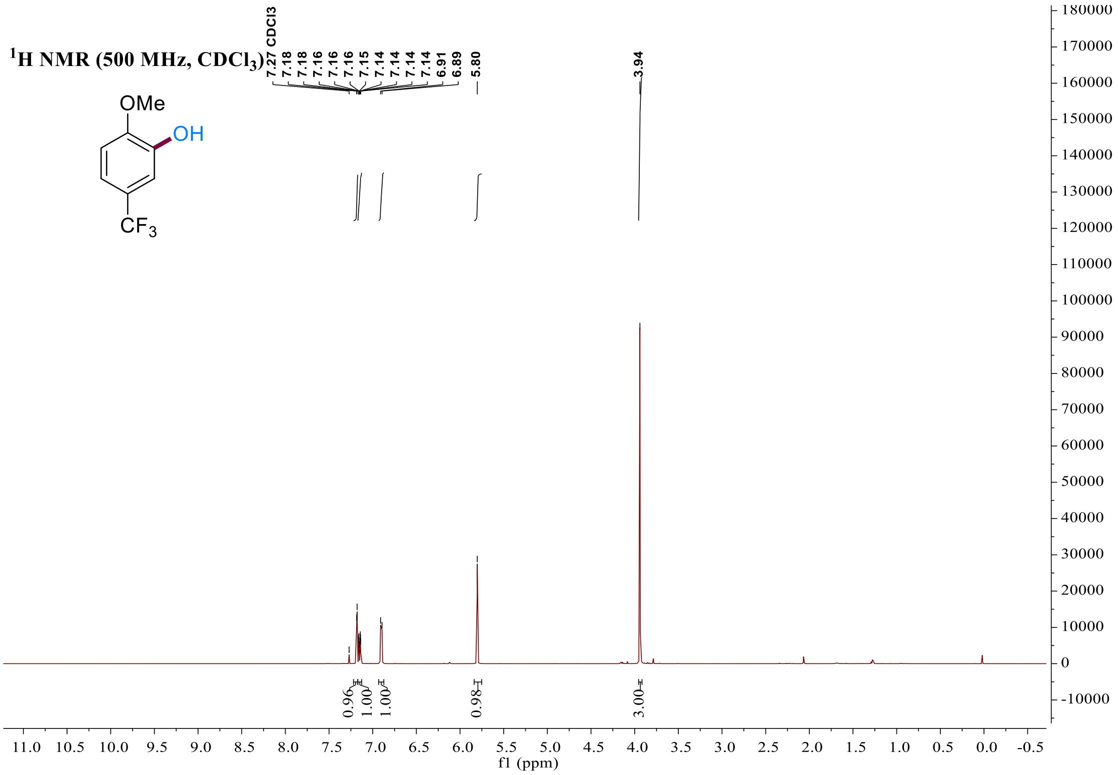

Supplementary Fig. 11. ^{1}H NMR spectra of compound **2** (500 MHz, rt, $CDCl_3$).

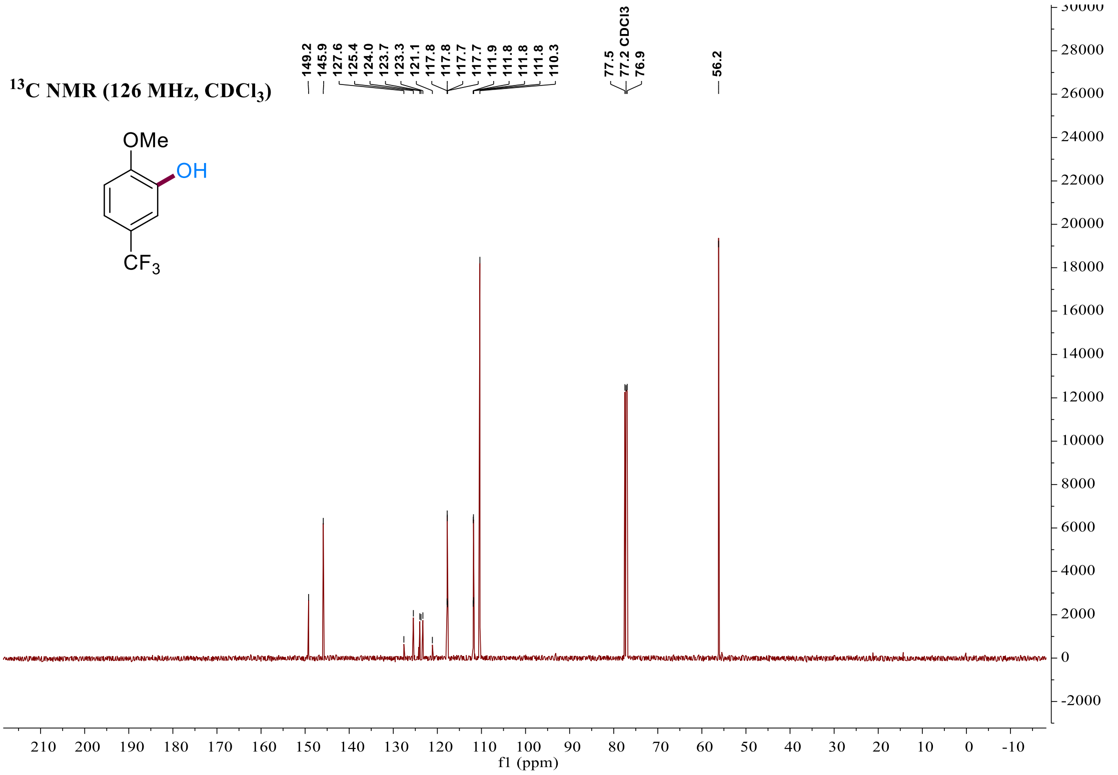

Supplementary Fig. 12. ^{13}C NMR spectra of compound **2** (126 MHz, rt, $CDCl_3$).

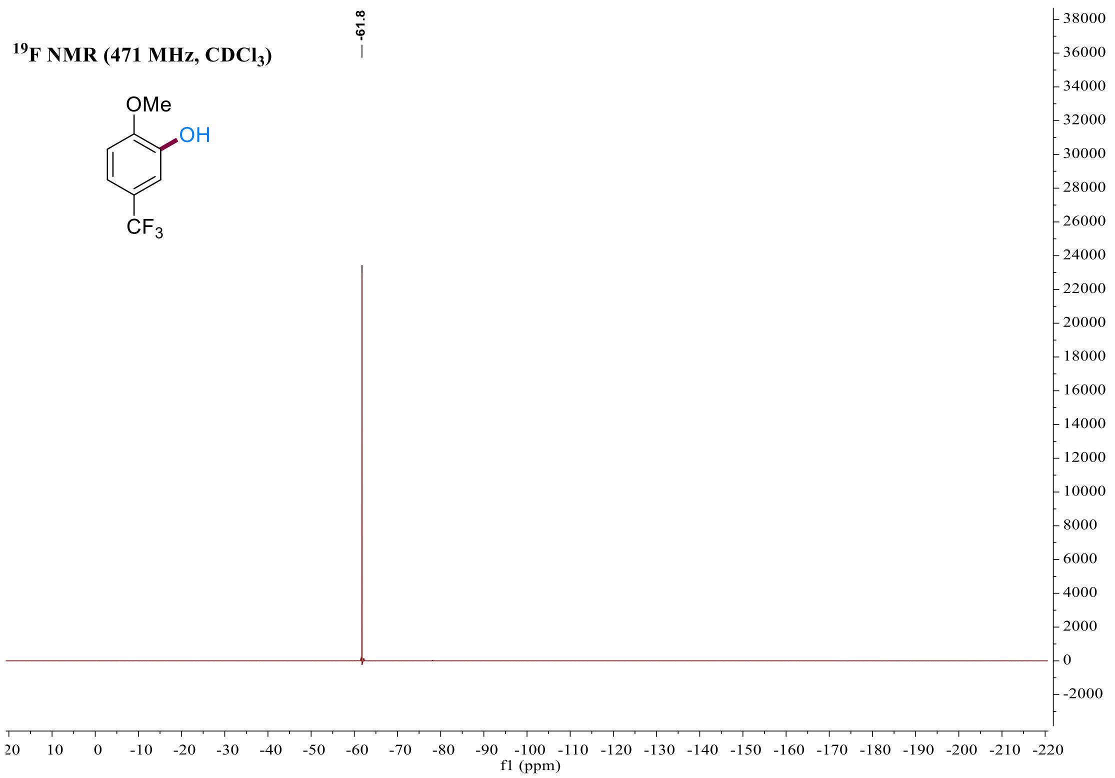

Supplementary Fig. 13. ^{19}F NMR spectra of compound **2** (471 MHz, rt, $CDCl_3$).

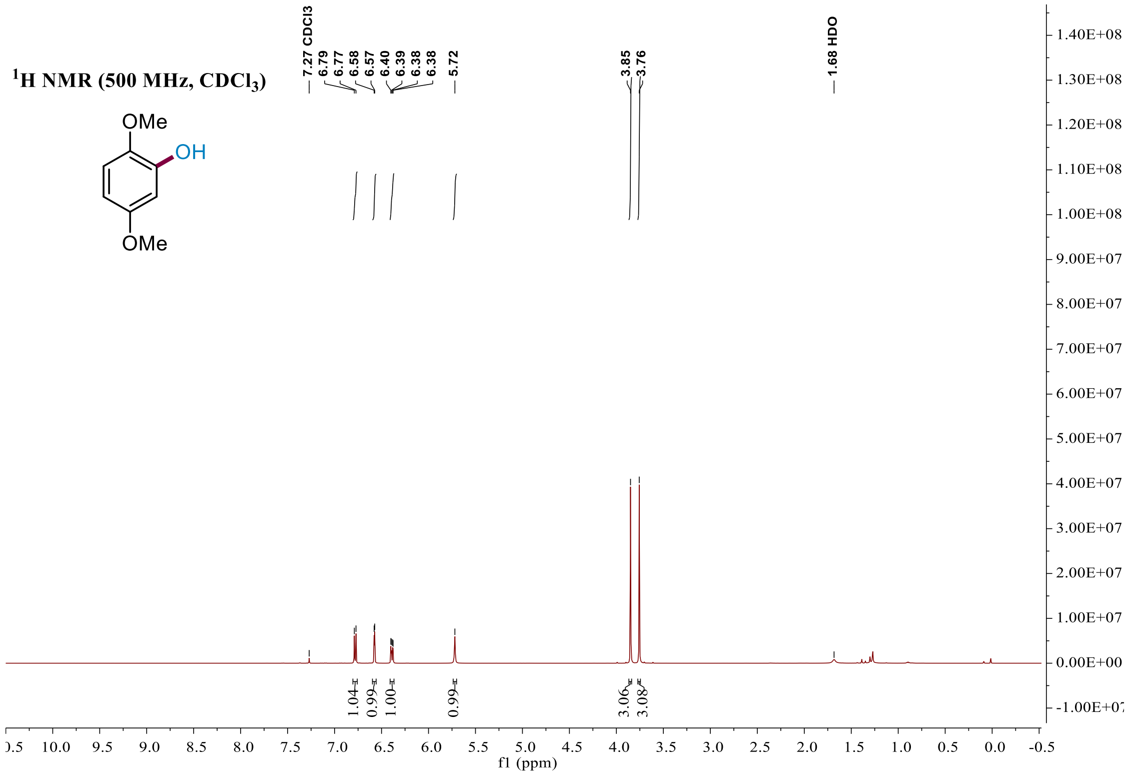

Supplementary Fig. 14. ^{1}H NMR spectra of compound **3** (500 MHz, rt, $CDCl_3$).

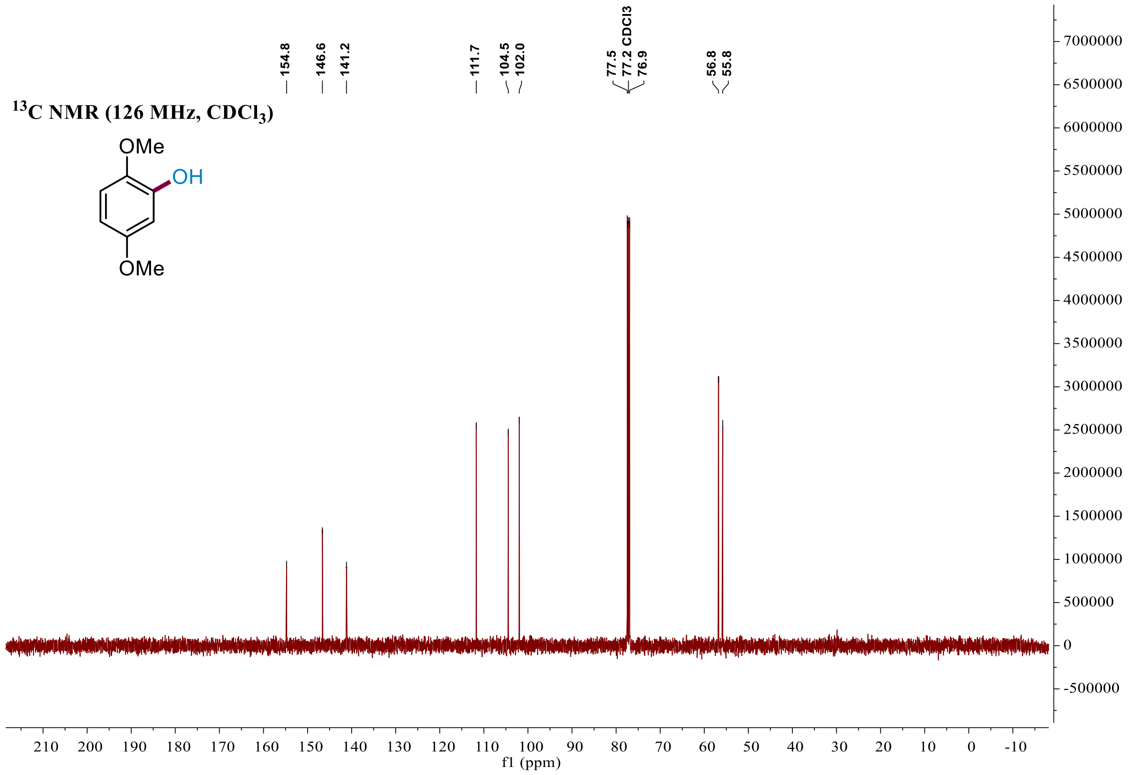

Supplementary Fig. 15. ^{13}C NMR spectra of compound **3** (126 MHz, rt, $CDCl_3$).

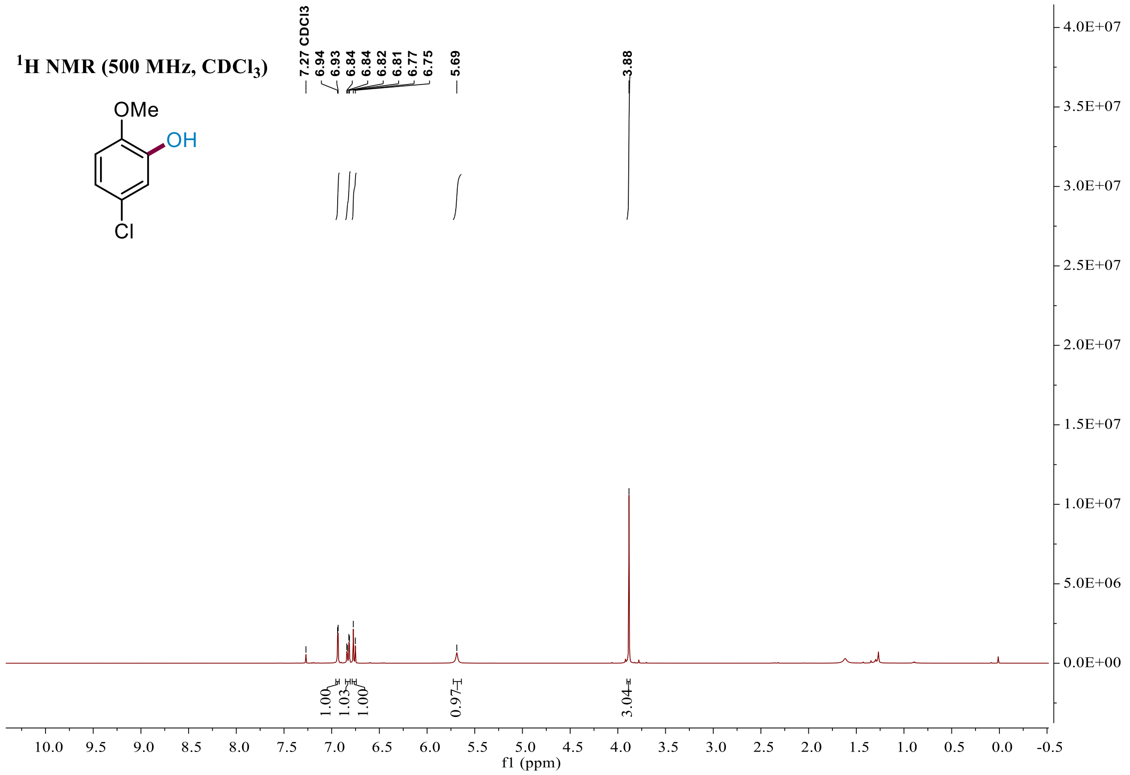

Supplementary Fig. 16. ^{1}H NMR spectra of compound **4** (500 MHz, rt, $CDCl_3$).

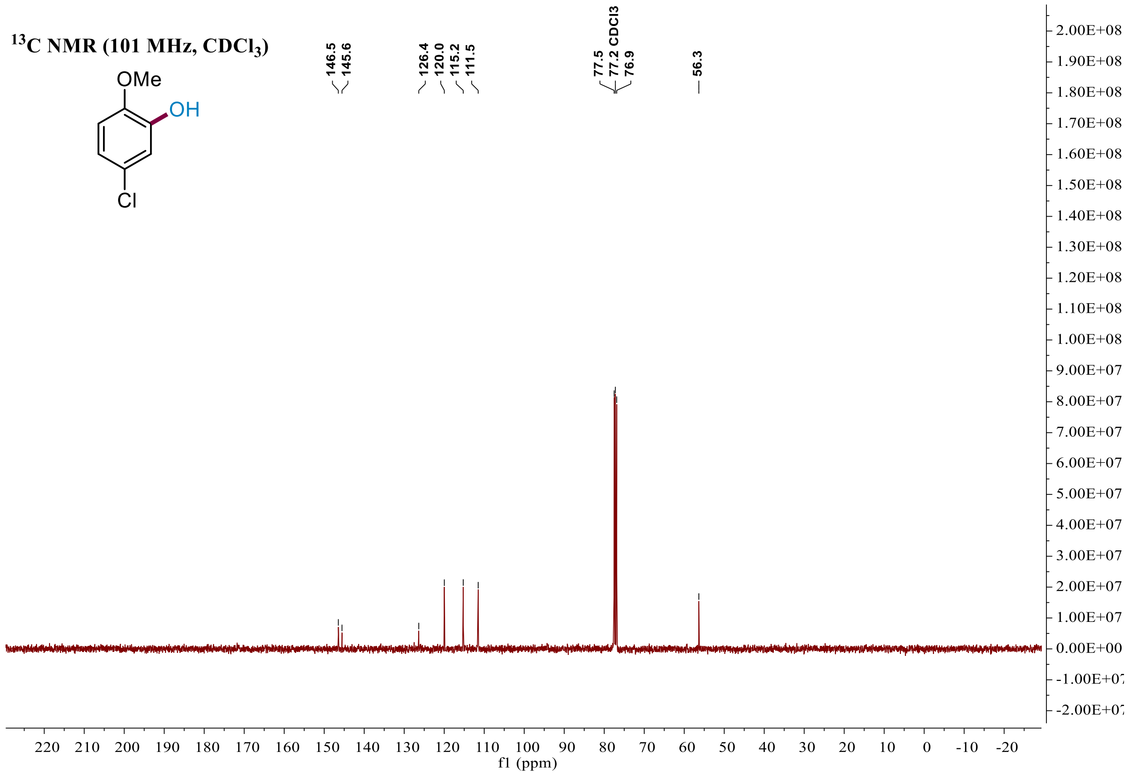

Supplementary Fig. 17. ^{13}C NMR spectra of compound **4** (101 MHz, rt, $CDCl_3$).

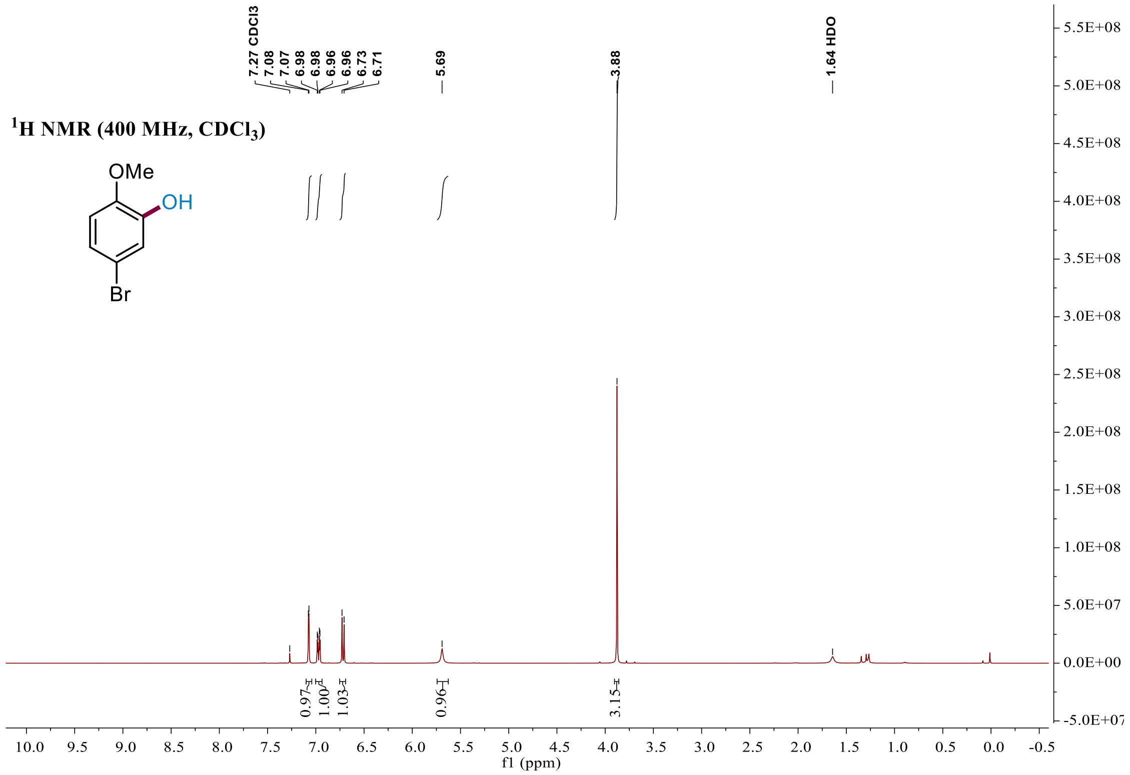

Supplementary Fig. 18. ^{1}H NMR spectra of compound **5** (400 MHz, rt, $CDCl_3$).

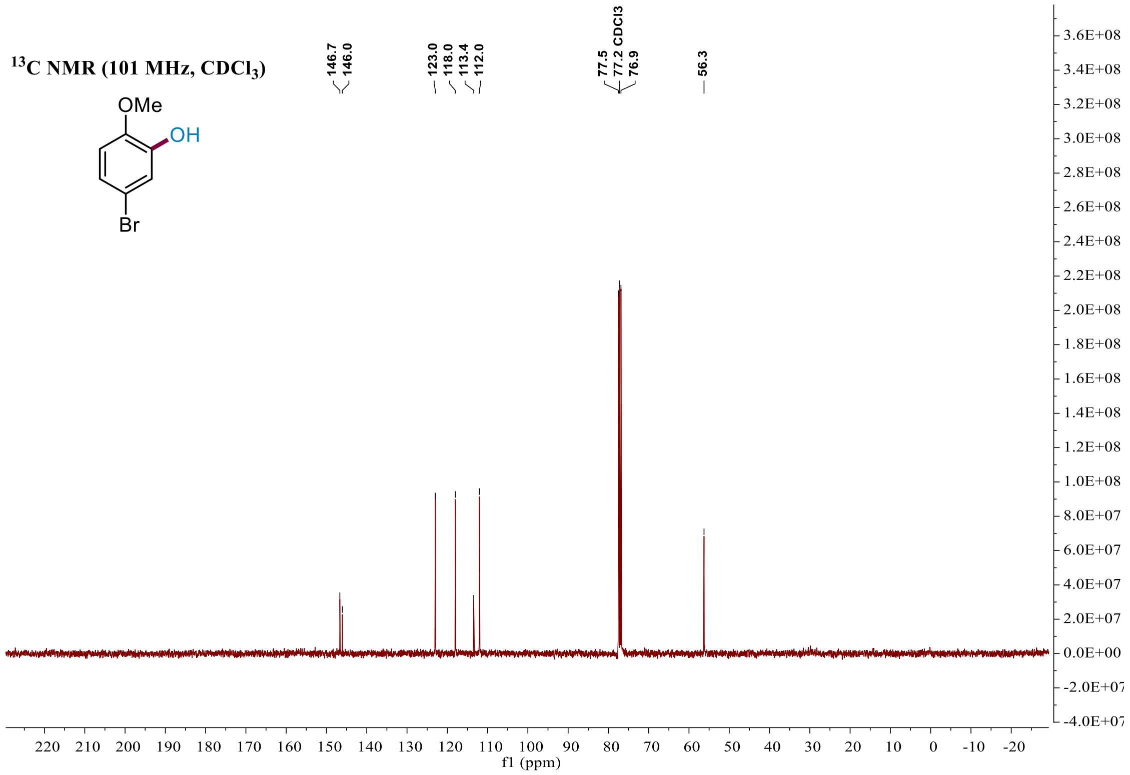

Supplementary Fig. 19. ^{13}C NMR spectra of compound **5** (101 MHz, rt, $CDCl_3$).

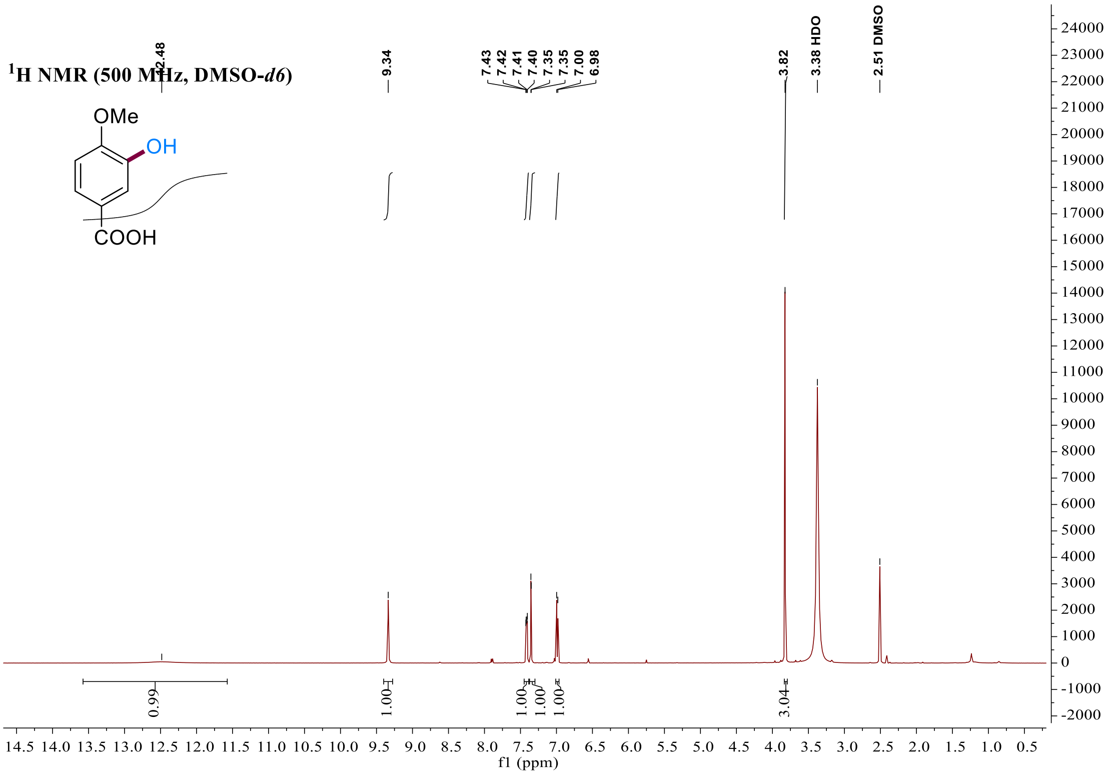

Supplementary Fig. 20. ^{1}H NMR spectra of compound **6** (500 MHz, rt, DMSO).

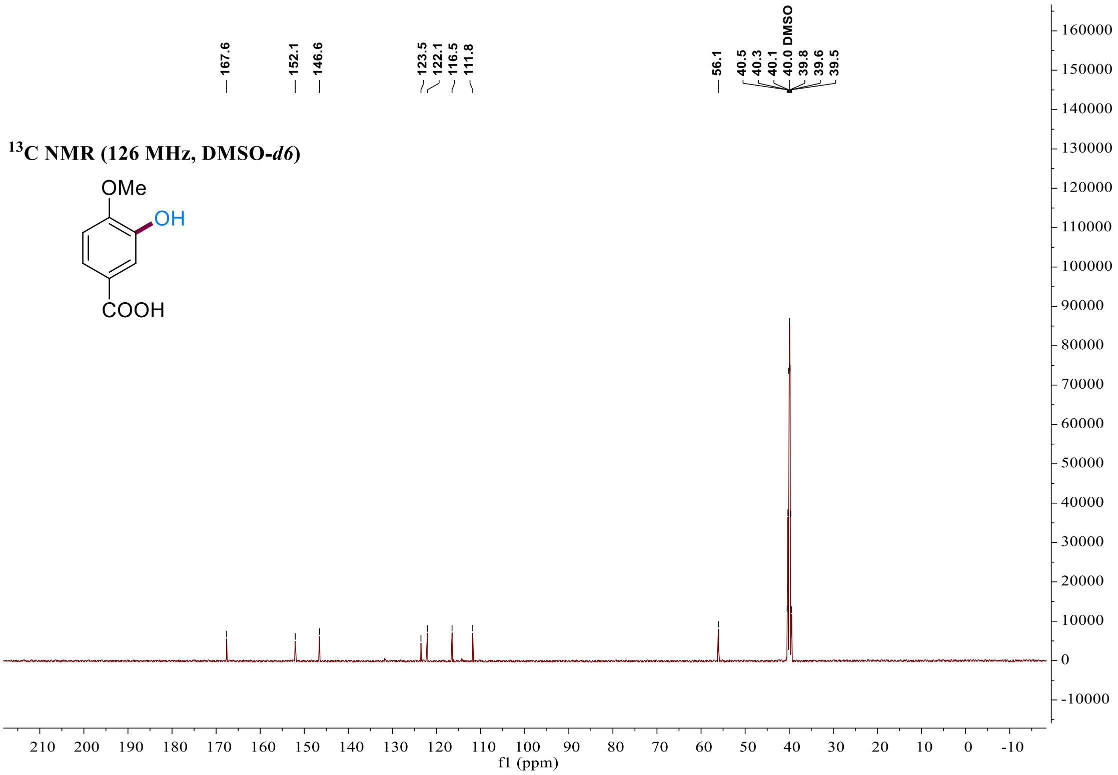

Supplementary Fig. 21. ^{13}C NMR spectra of compound **6** (126 MHz, rt, DMSO).

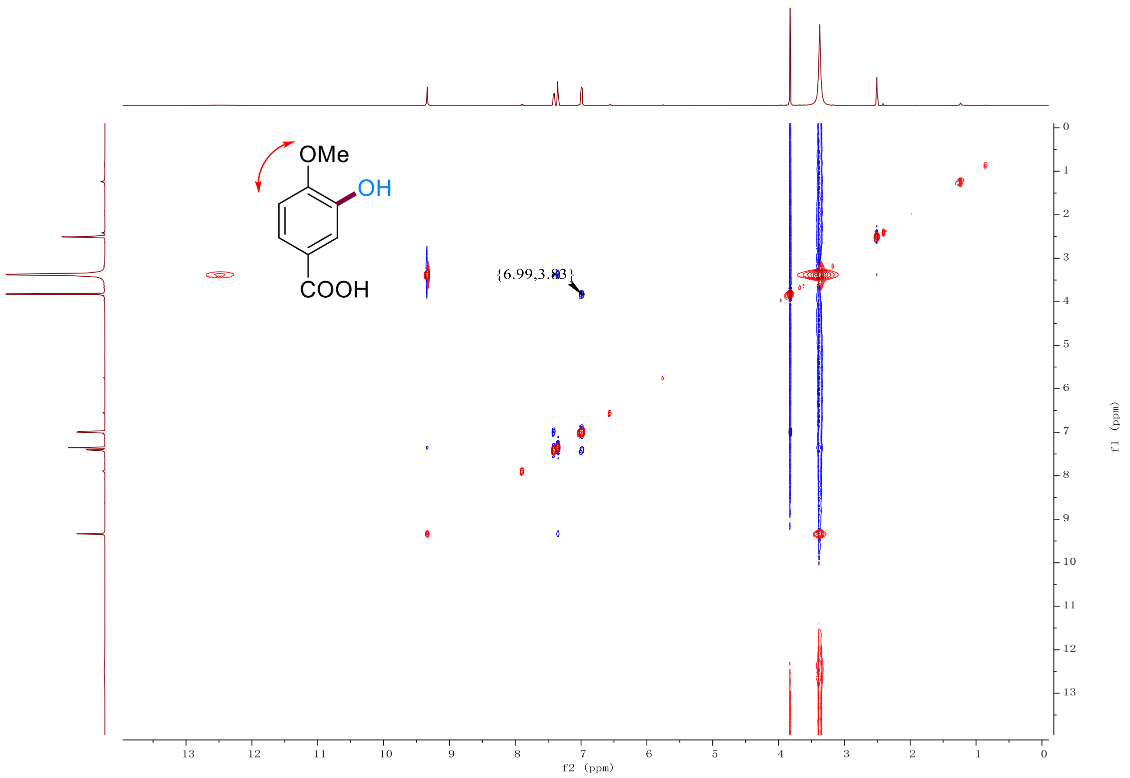

Supplementary Fig. 22. 2D NOESY spectra of compound **6** (500 MHz, rt, $CDCl_3$).

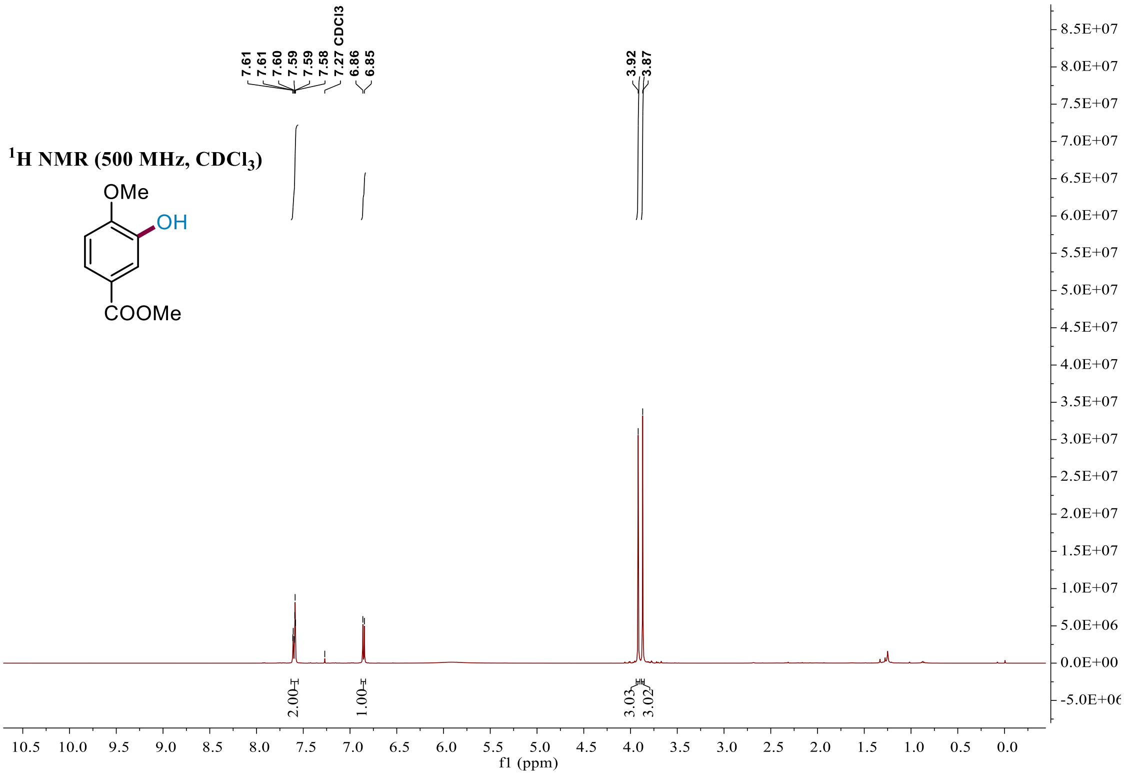

Supplementary Fig. 23. ^{1}H NMR spectra of compound **7** (500 MHz, rt, $CDCl_3$).

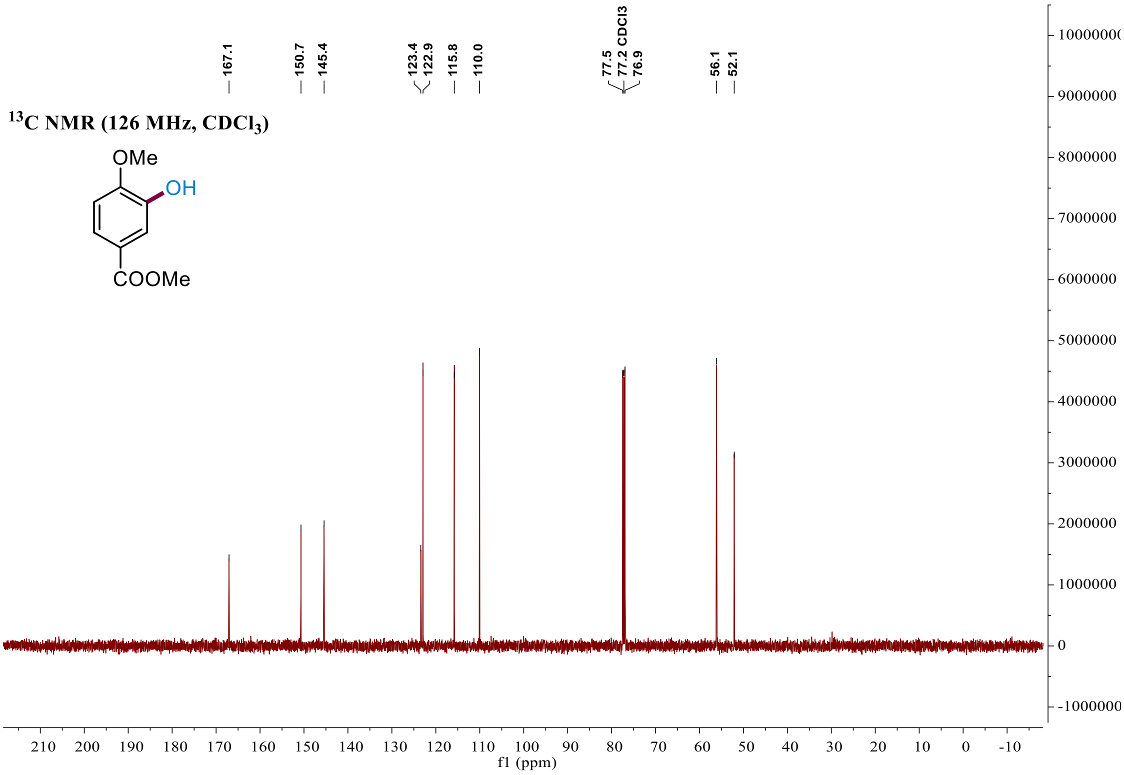

Supplementary Fig. 24. ^{13}C NMR spectra of compound **7** (126 MHz, rt, $CDCl_3$).

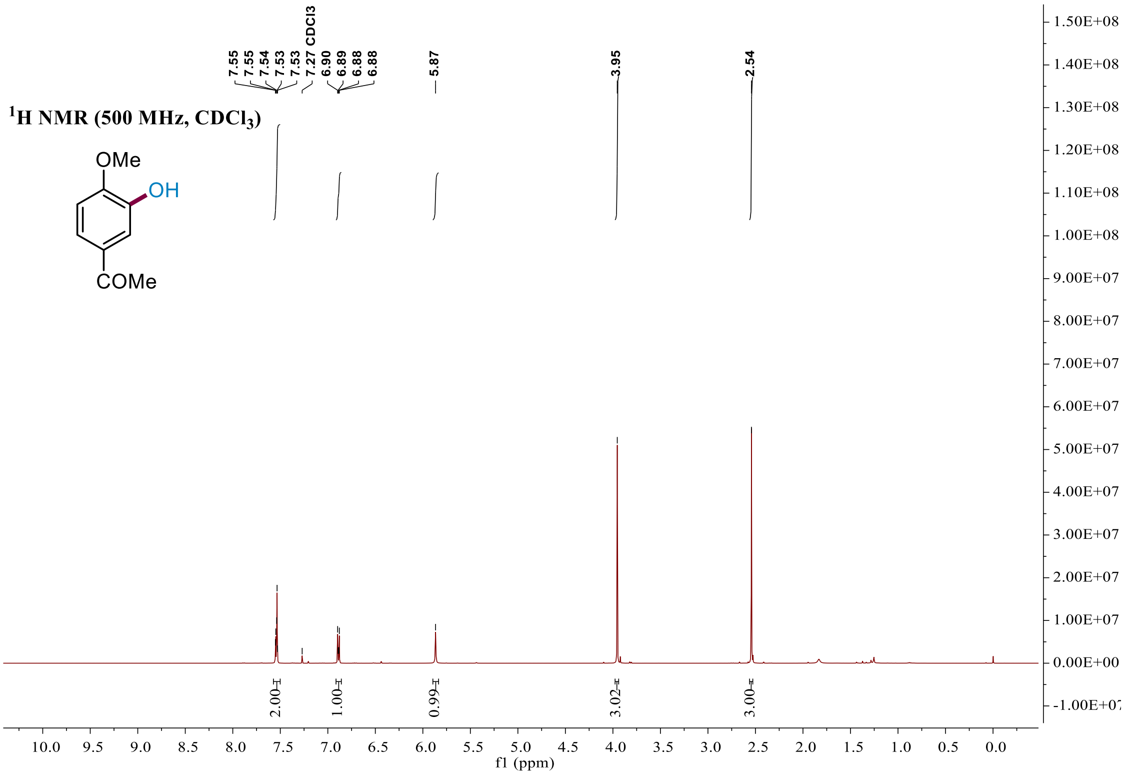

Supplementary Fig. 25. ^{1}H NMR spectra of compound **8** (500 MHz, rt, $CDCl_3$).

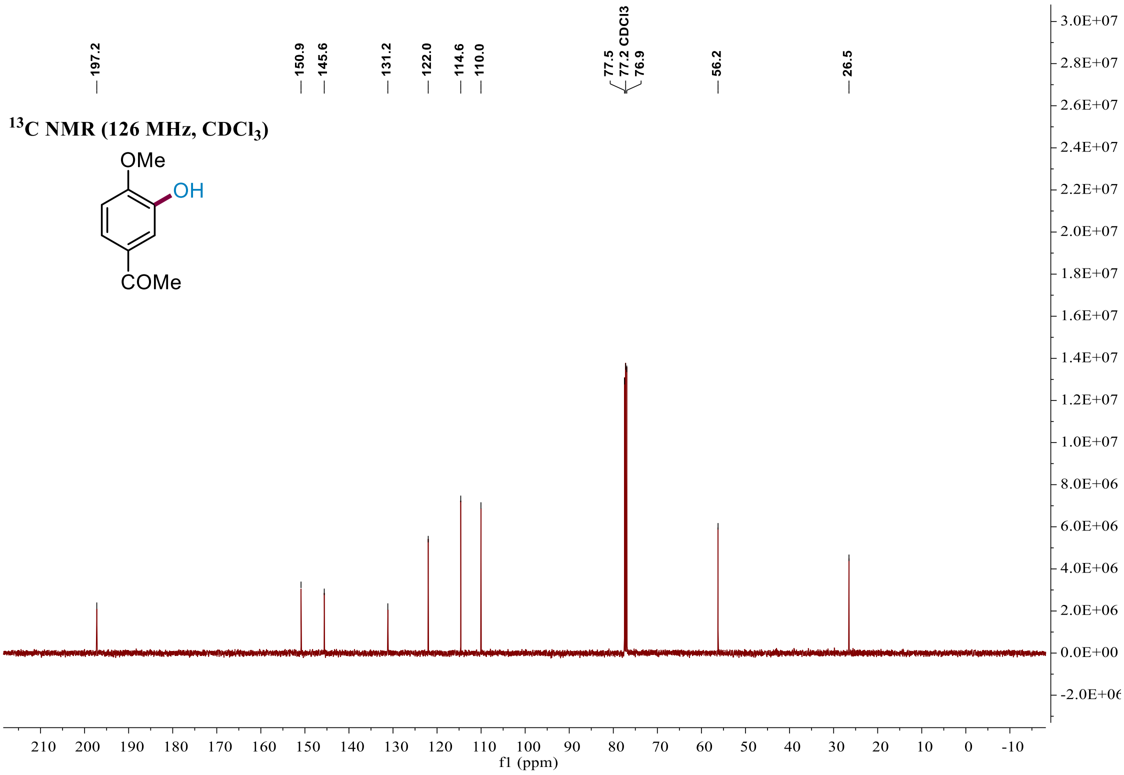

Supplementary Fig. 26. ^{13}C NMR spectra of compound **8** (126 MHz, rt, $CDCl_3$).

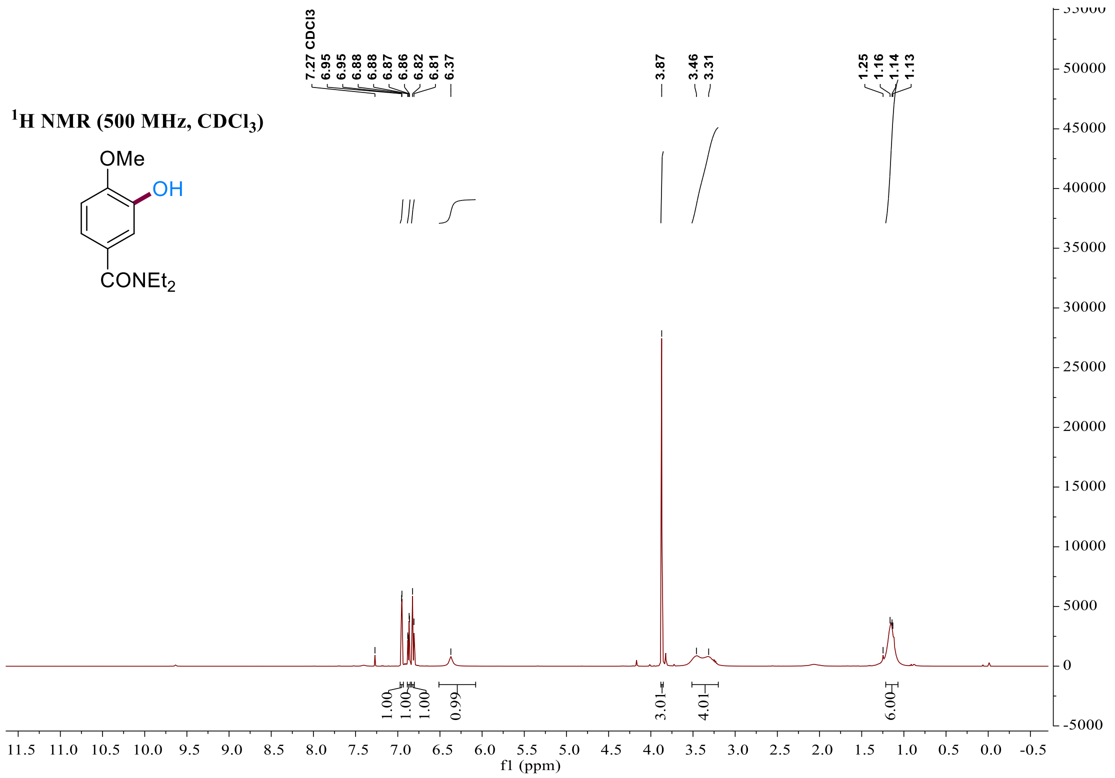

Supplementary Fig. 27. ^{1}H NMR spectra of compound **9** (500 MHz, rt, $CDCl_3$).

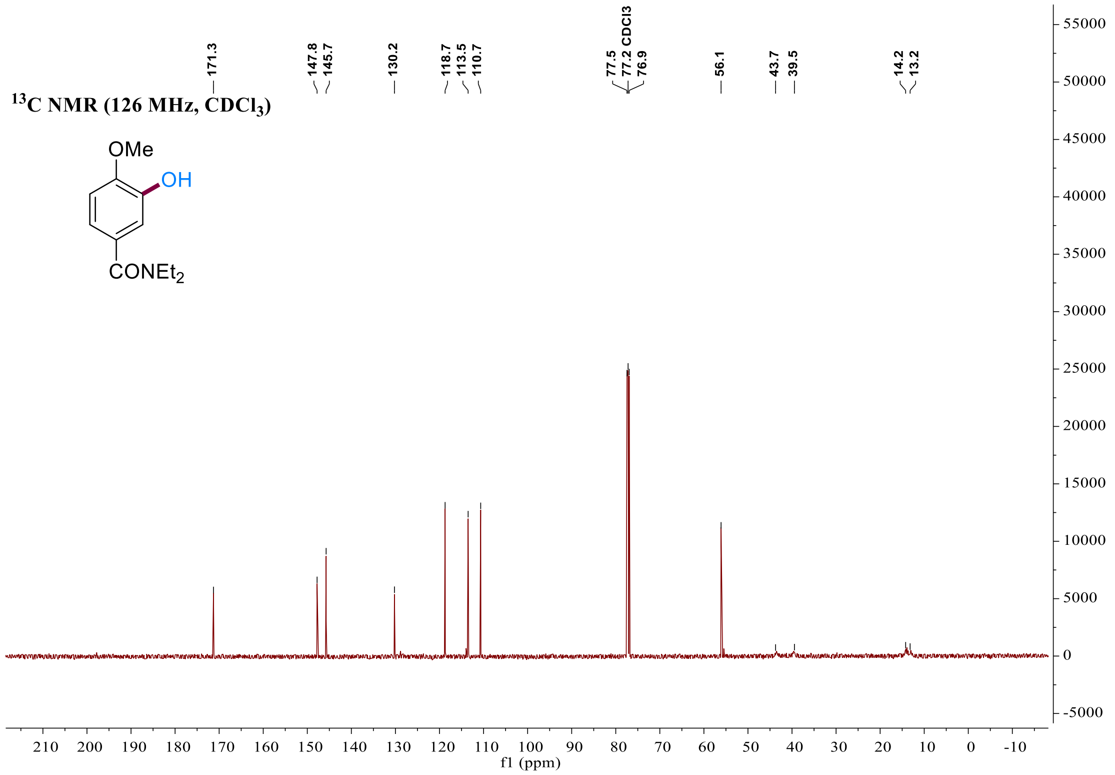

Supplementary Fig. 28. ^{13}C NMR spectra of compound **9** (126 MHz, rt, $CDCl_3$).

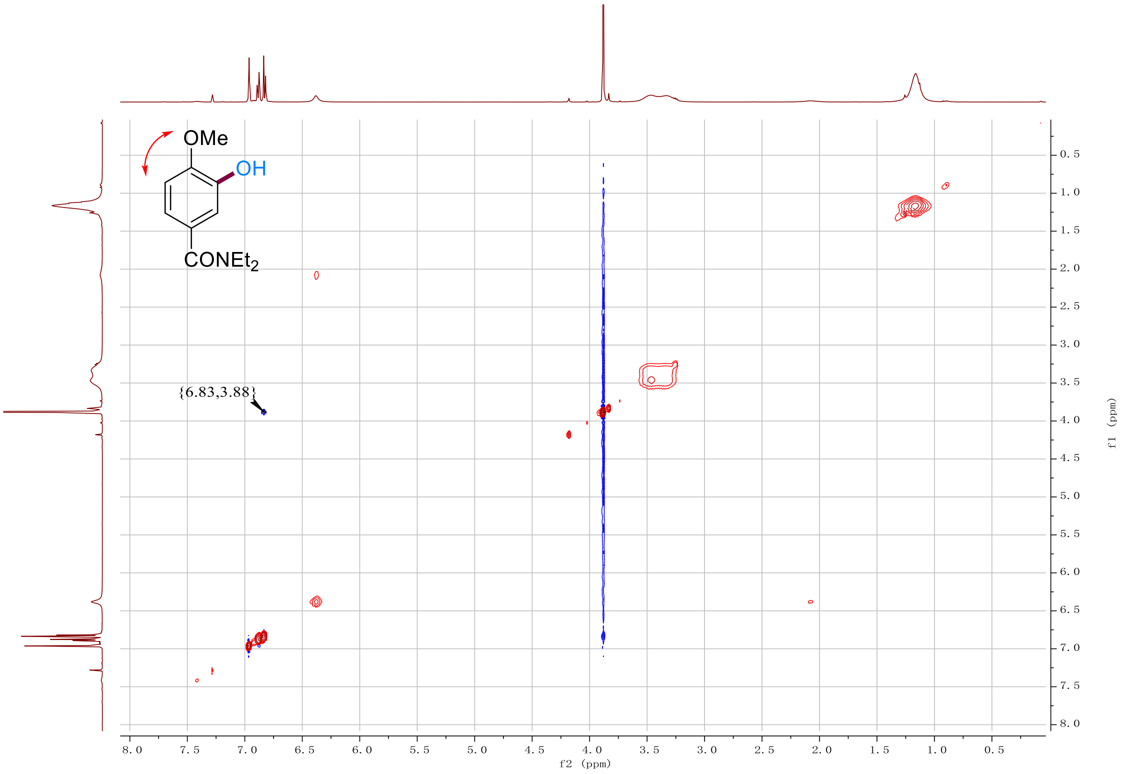

Supplementary Fig. 29. 2D NOESY spectra of compound **9** (500 MHz, rt, $CDCl_3$).

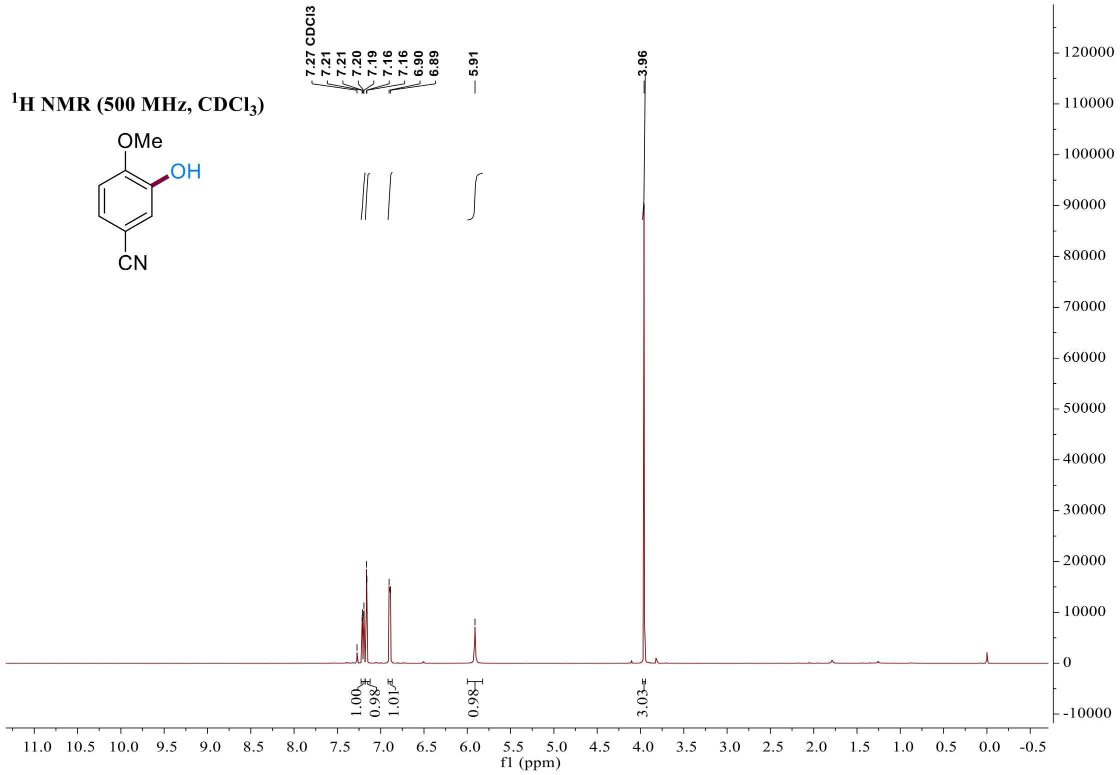

Supplementary Fig. 30. ^{1}H NMR spectra of compound **10** (500 MHz, rt, $CDCl_3$).

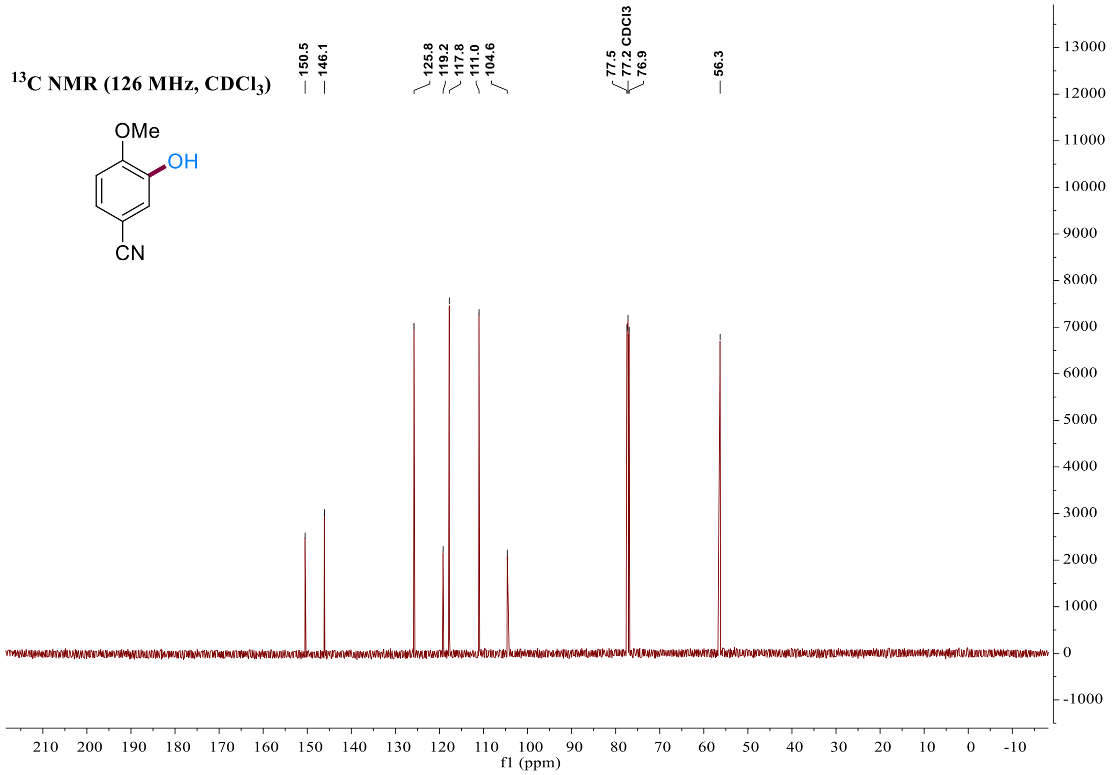

Supplementary Fig. 31. ^{13}C NMR spectra of compound **10** (126 MHz, rt, $CDCl_3$).

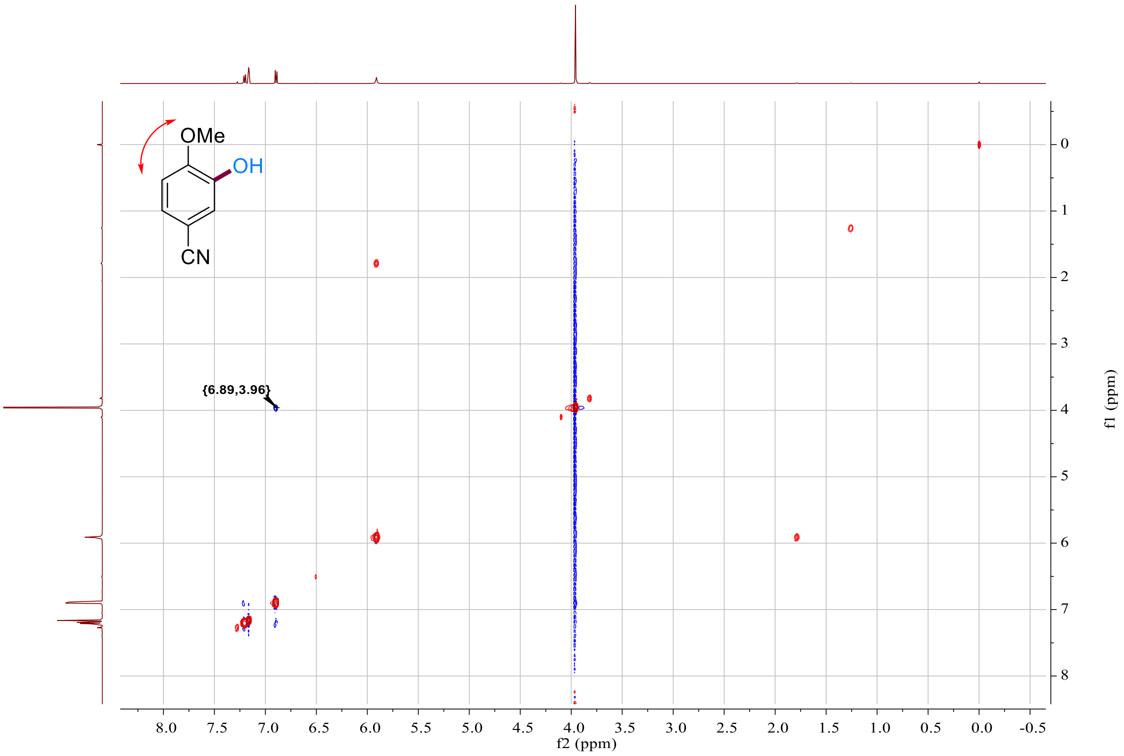

Supplementary Fig. 32. 2D NOESY spectra of compound **10** (500 MHz, rt, $CDCl_3$).

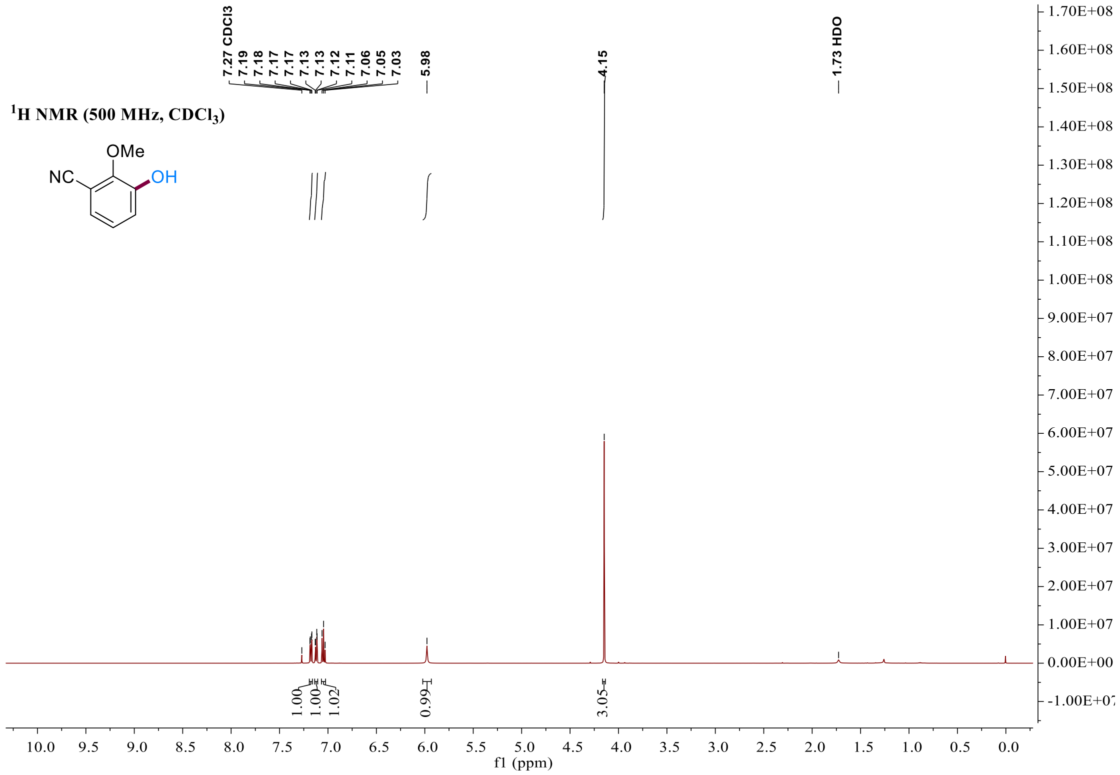

Supplementary Fig. 33. ^{1}H NMR spectra of compound **11** (500 MHz, rt, $CDCl_3$).

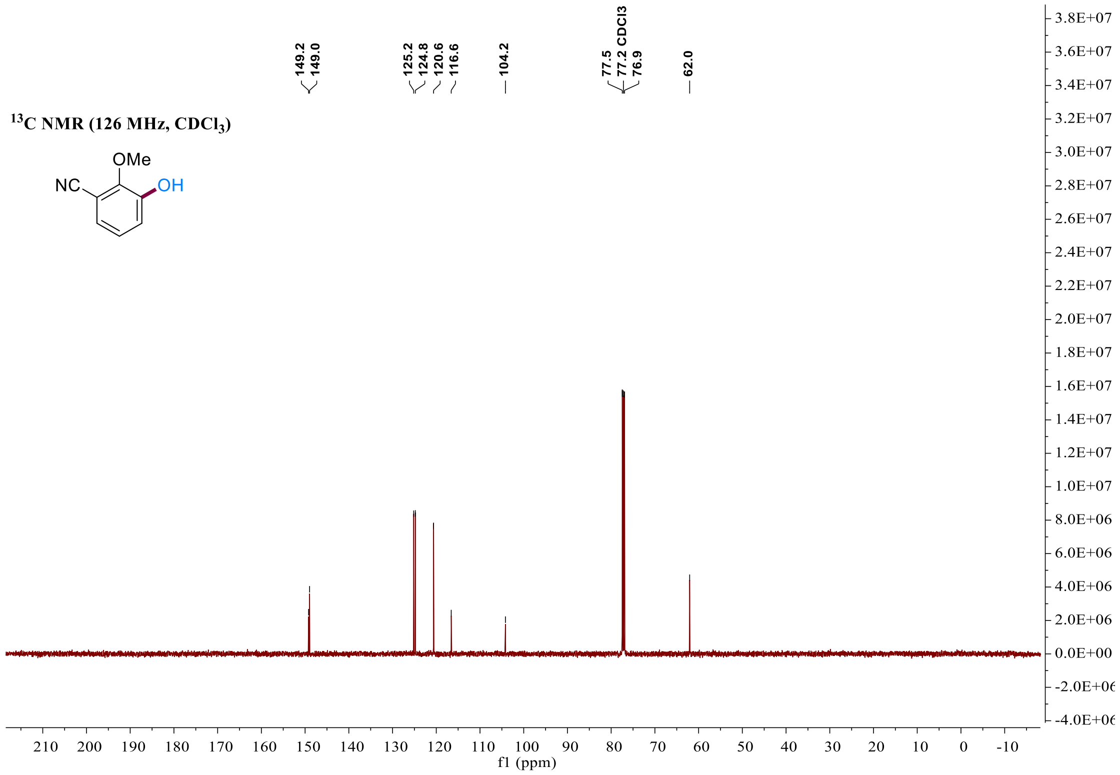

Supplementary Fig. 34. ^{13}C NMR spectra of compound **11** (126 MHz, rt, $CDCl_3$).

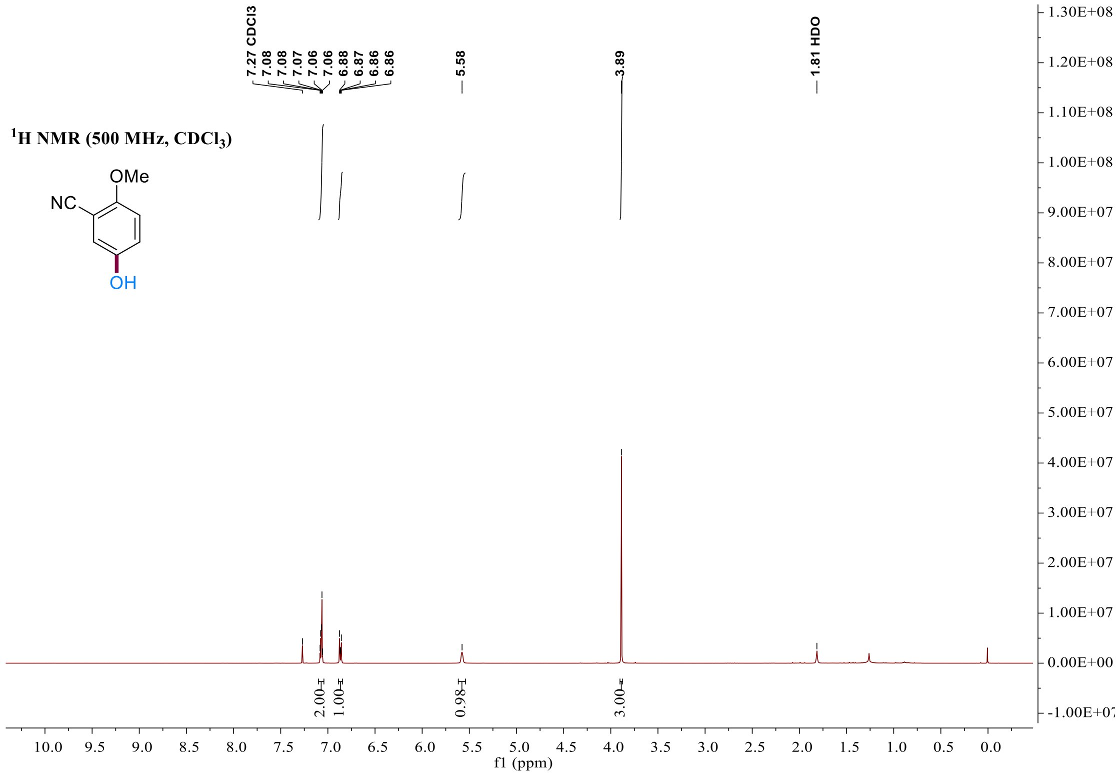

Supplementary Fig. 35. ^{1}H NMR spectra of compound **11** (500 MHz, rt, CDCl$_3$).

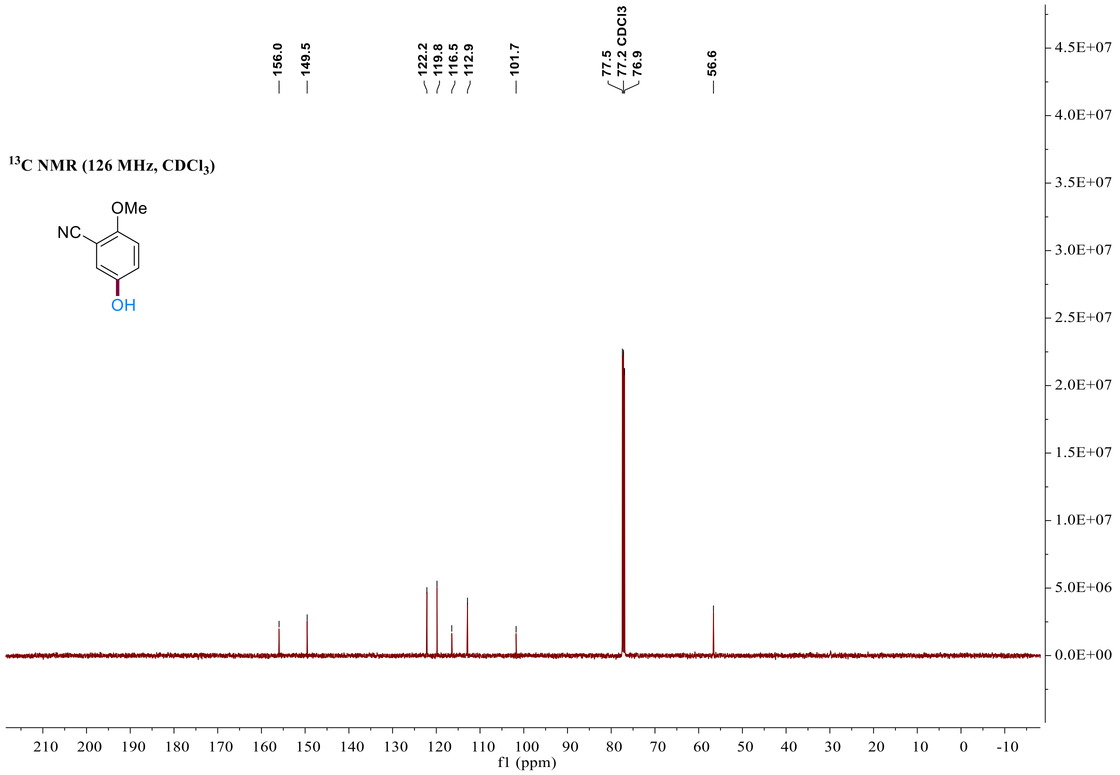

Supplementary Fig. 36. ^{13}C NMR spectra of compound **11** (126 MHz, rt, CDCl$_3$).

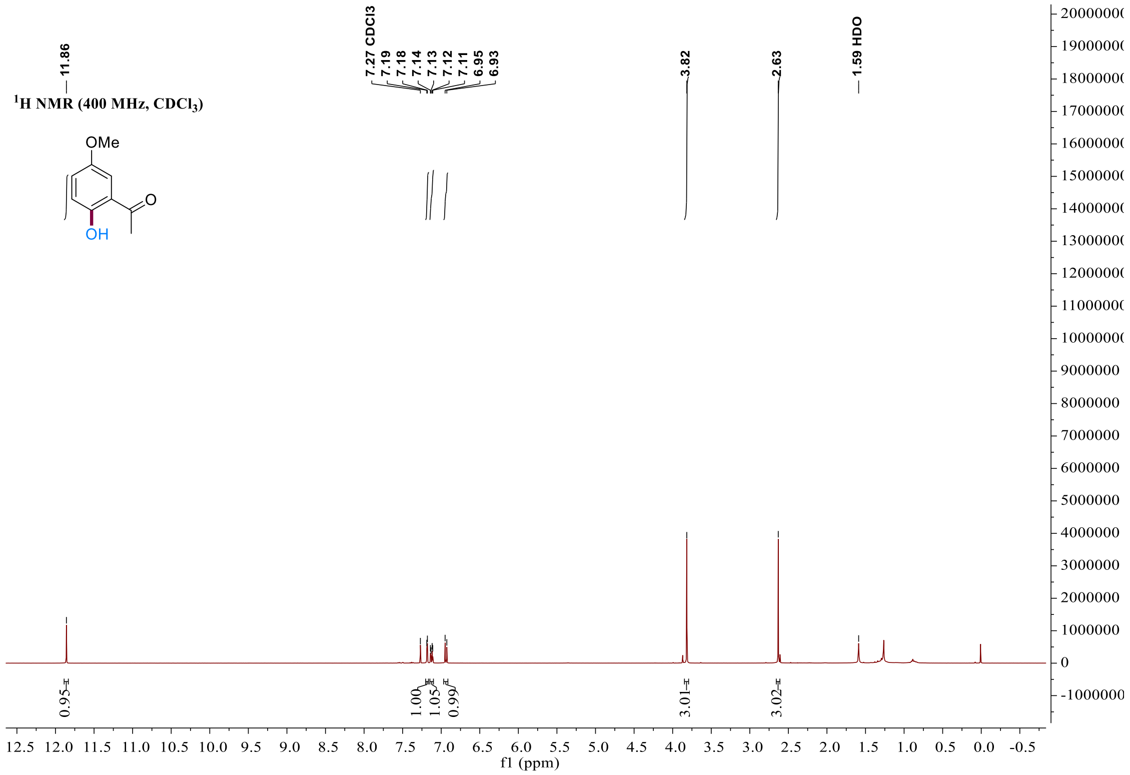

Supplementary Fig. 37. ^{1}H NMR spectra of compound **12** (400 MHz, rt, $CDCl_3$).

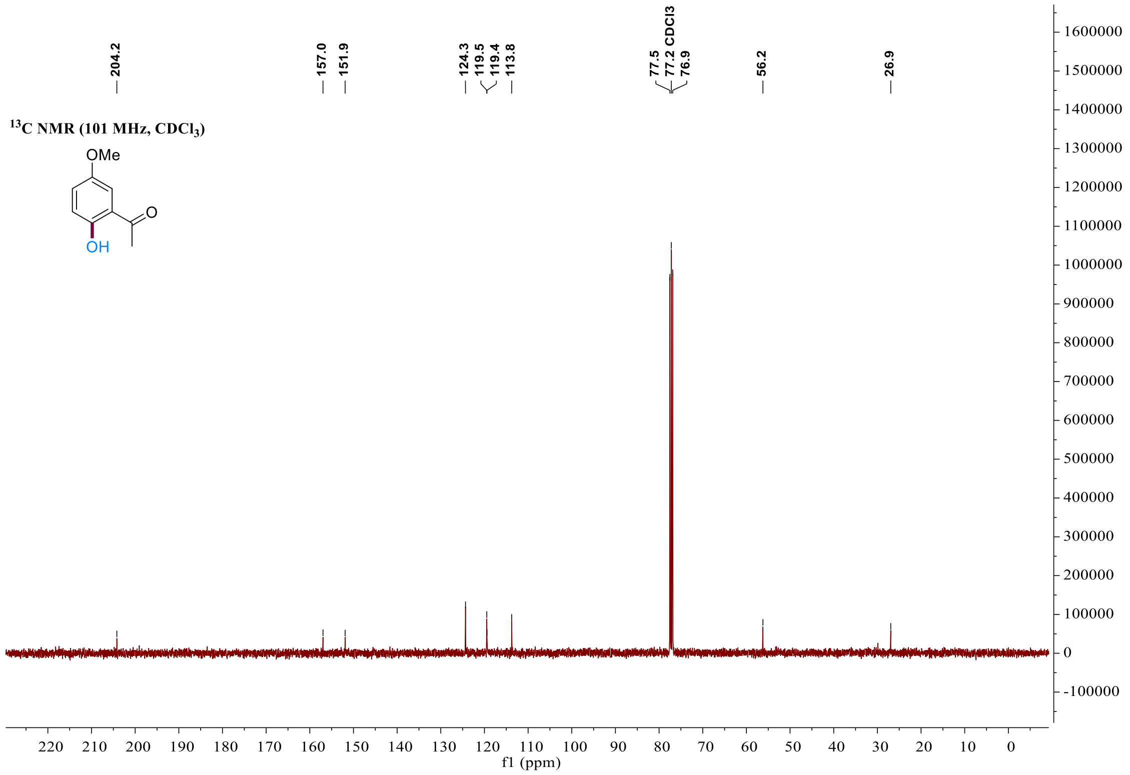

Supplementary Fig. 38. ^{13}C NMR spectra of compound **12** (101 MHz, rt, $CDCl_3$).

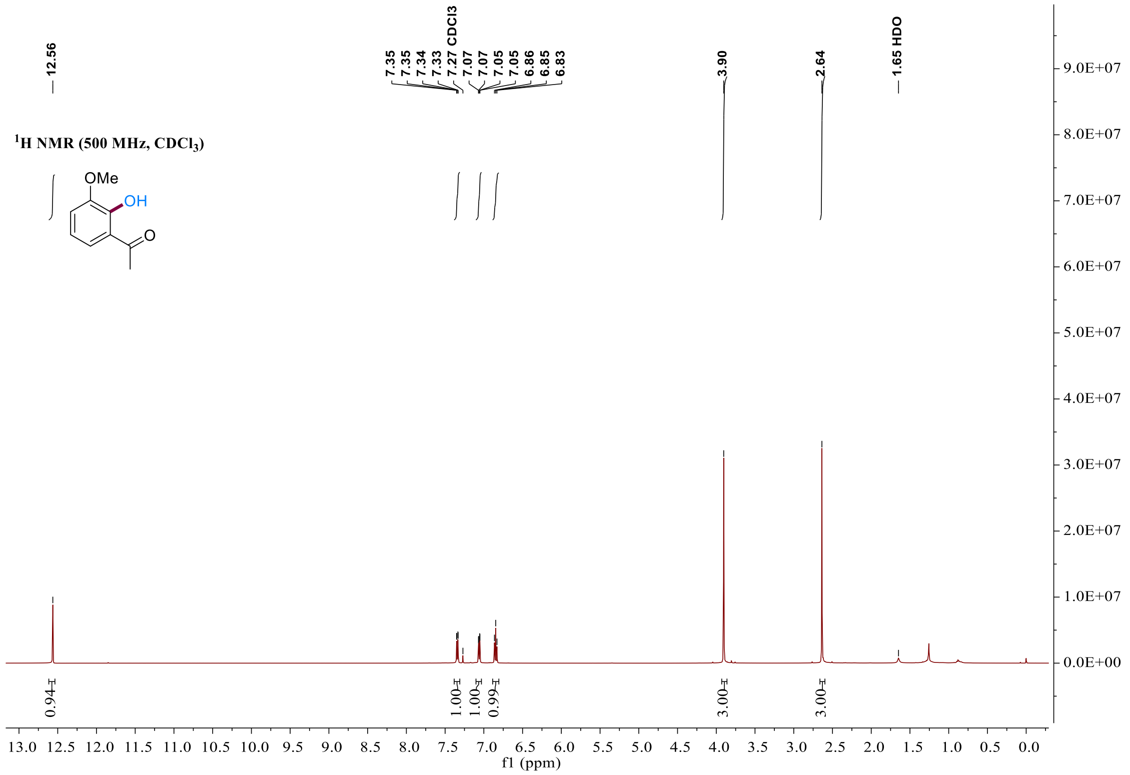

Supplementary Fig. 39. ^{1}H NMR spectra of compound **12** (500 MHz, rt, $CDCl_3$).

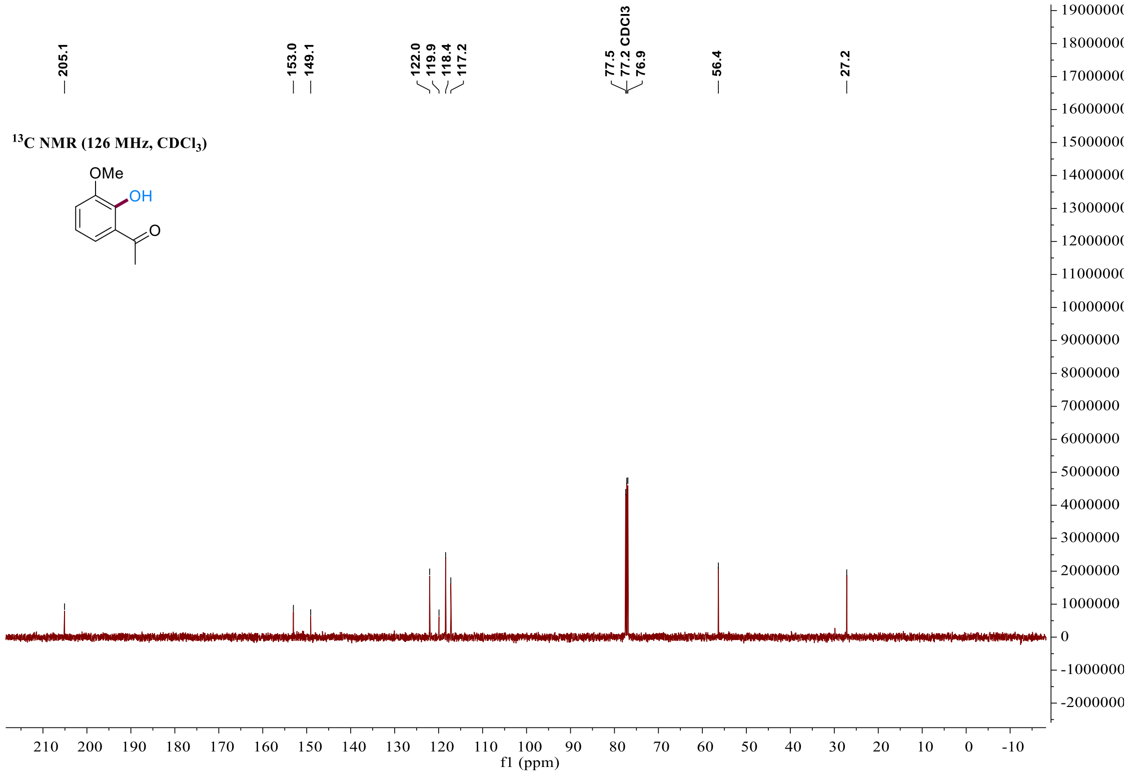

Supplementary Fig. 40. ^{13}C NMR spectra of compound **12** (126 MHz, rt, $CDCl_3$).

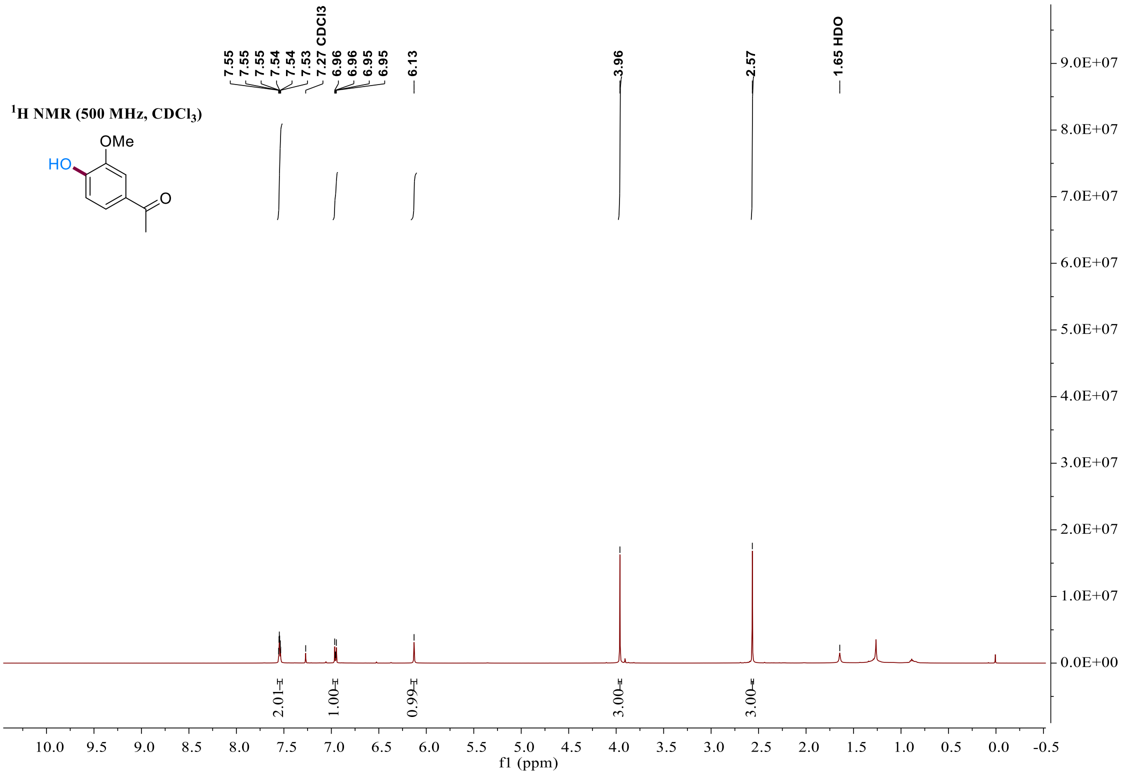

Supplementary Fig. 41. ^{1}H NMR spectra of compound **12** (500 MHz, rt, $CDCl_3$).

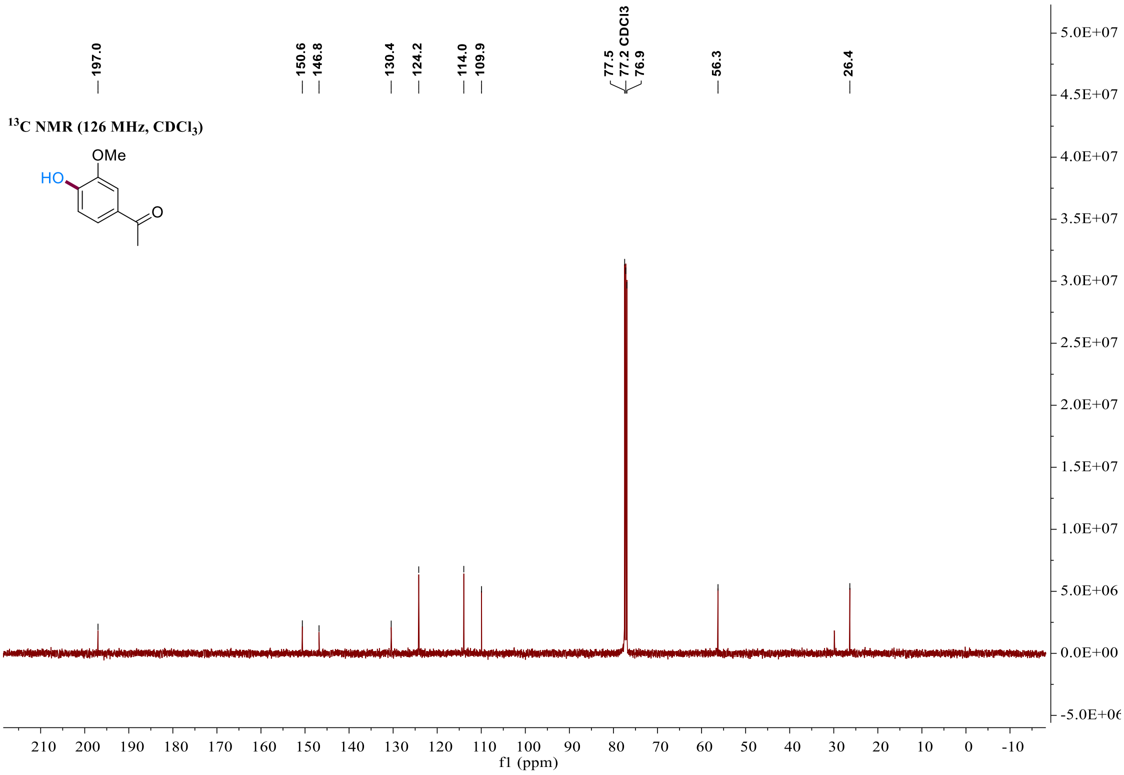

Supplementary Fig. 42. ^{13}C NMR spectra of compound **12** (126 MHz, rt, $CDCl_3$).

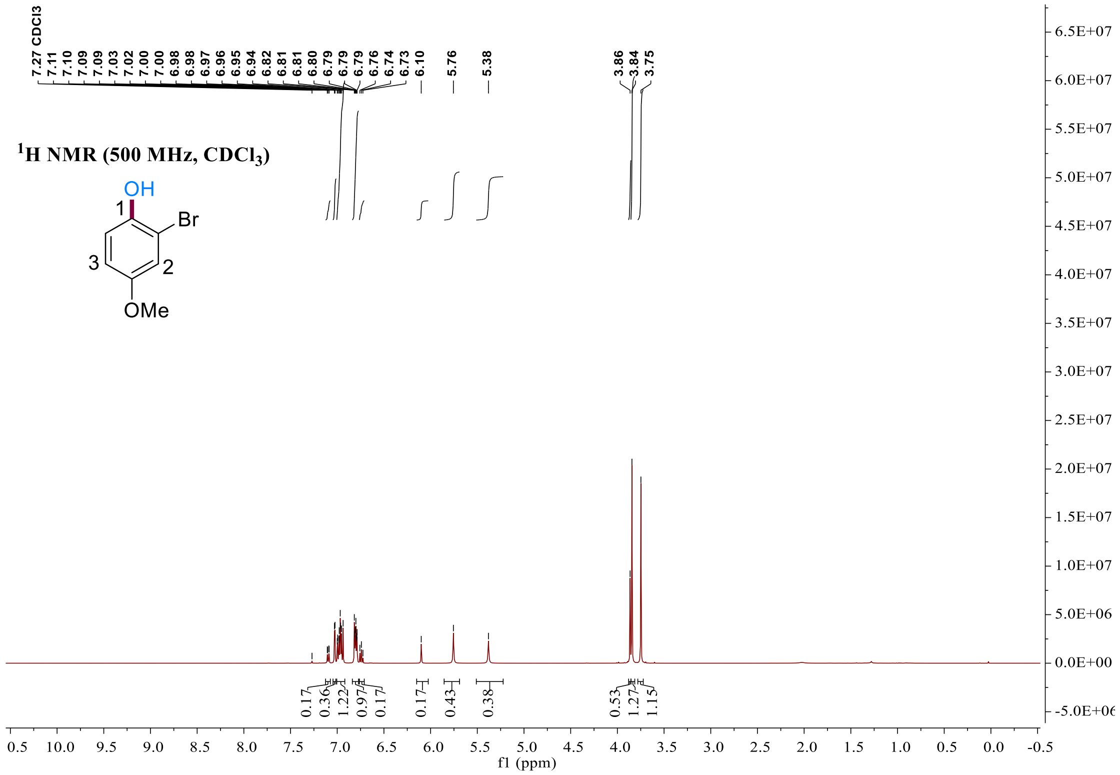

Supplementary Fig. 43. ^{1}H NMR spectra of compound **13** (500 MHz, rt, $CDCl_3$).

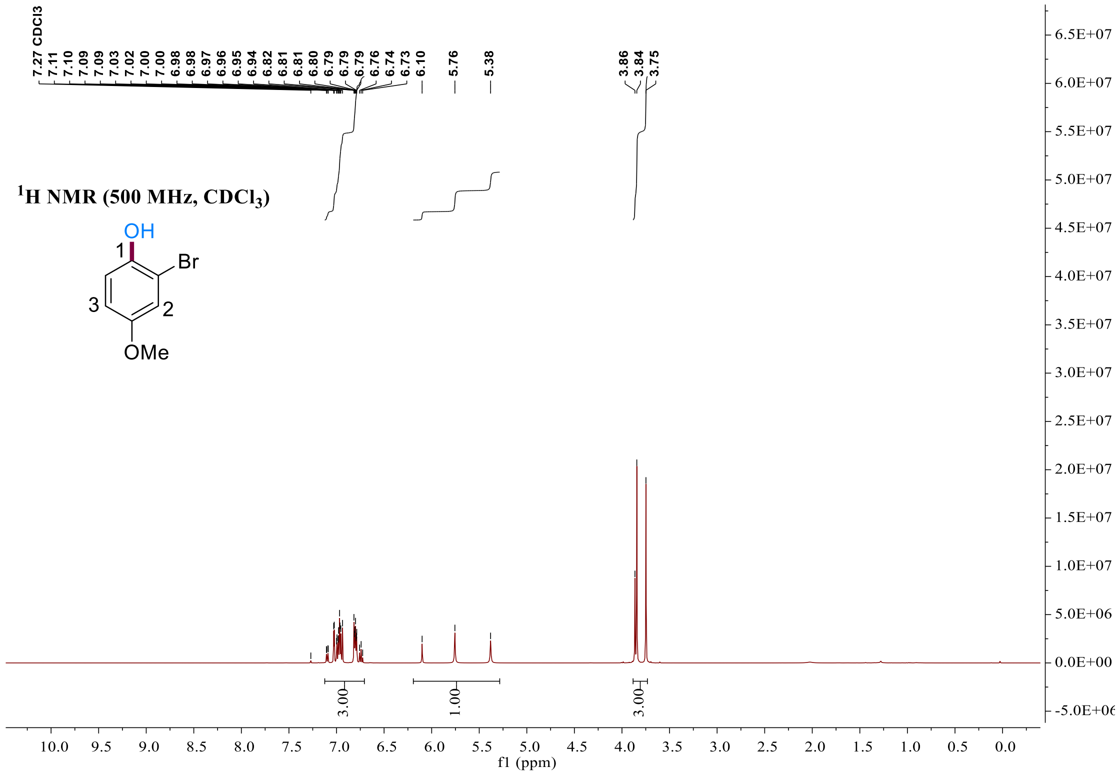

Supplementary Fig. 44. ^{1}H NMR spectra of compound **13** (500 MHz, rt, $CDCl_3$).

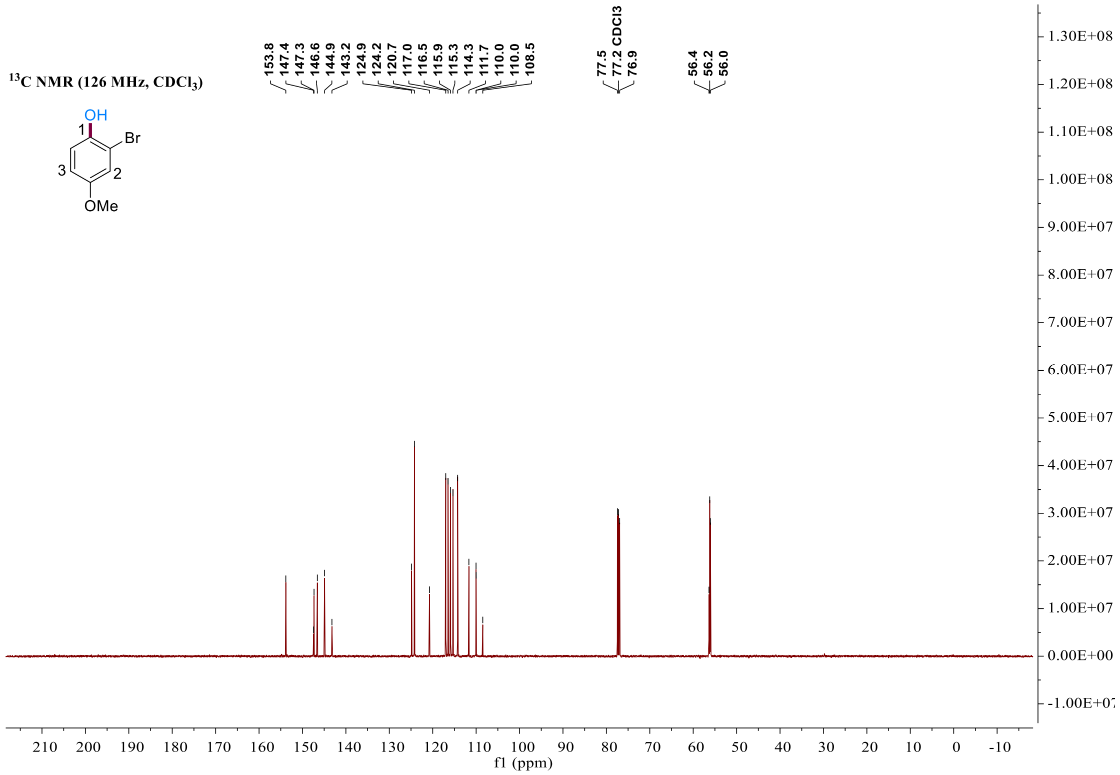

Supplementary Fig. 45. ^{13}C NMR spectra of compound **13** (126 MHz, rt, CDCl$_3$).

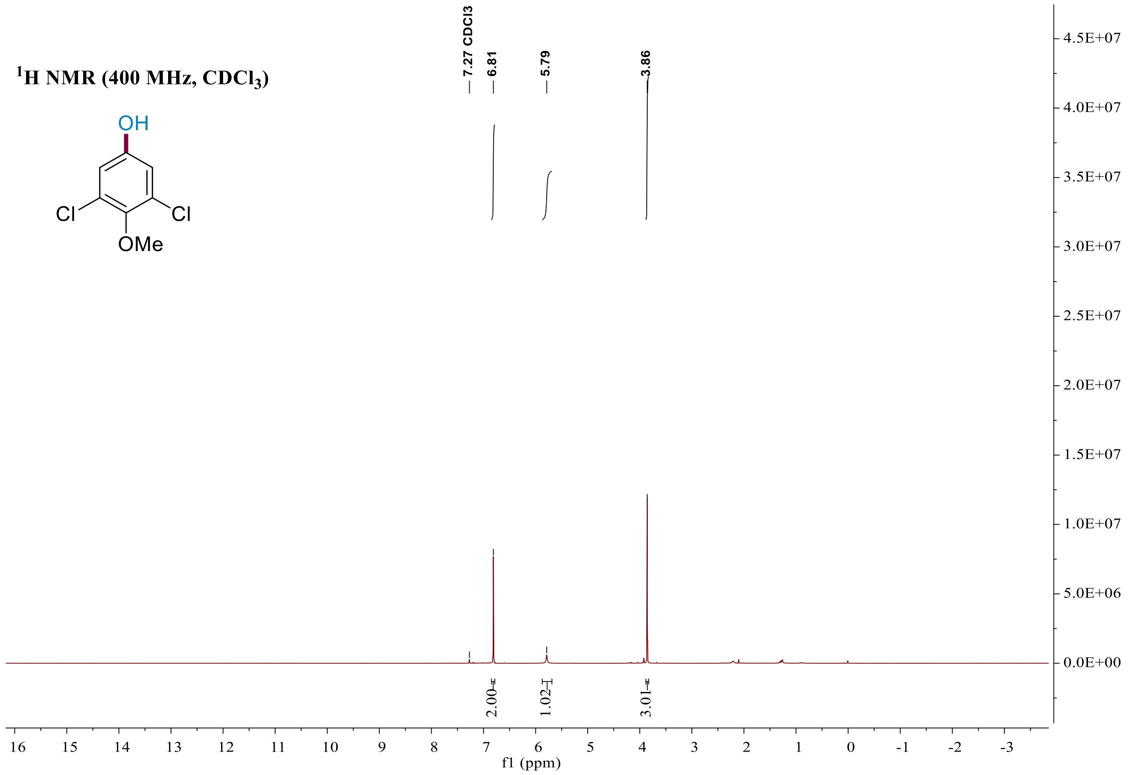

Supplementary Fig. 46. ^{1}H NMR spectra of compound **14** (400 MHz, rt, $CDCl_3$).

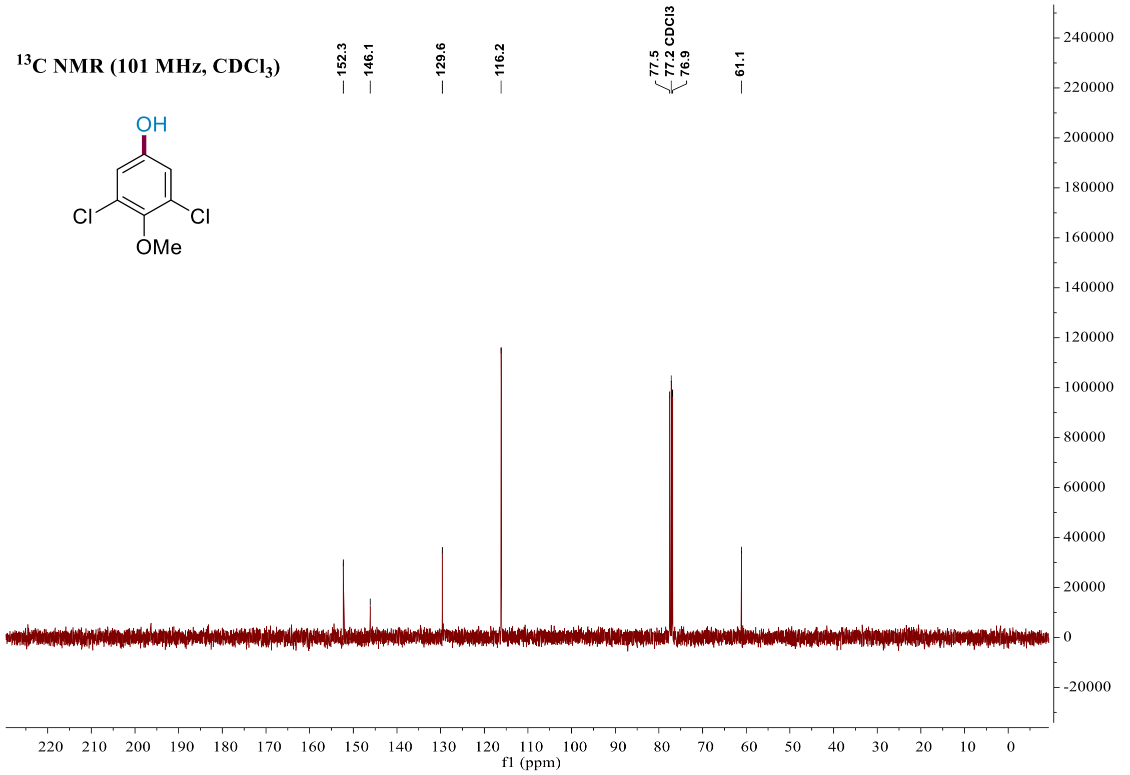

Supplementary Fig. 47. ^{13}C NMR spectra of compound **14** (101 MHz, rt, $CDCl_3$).

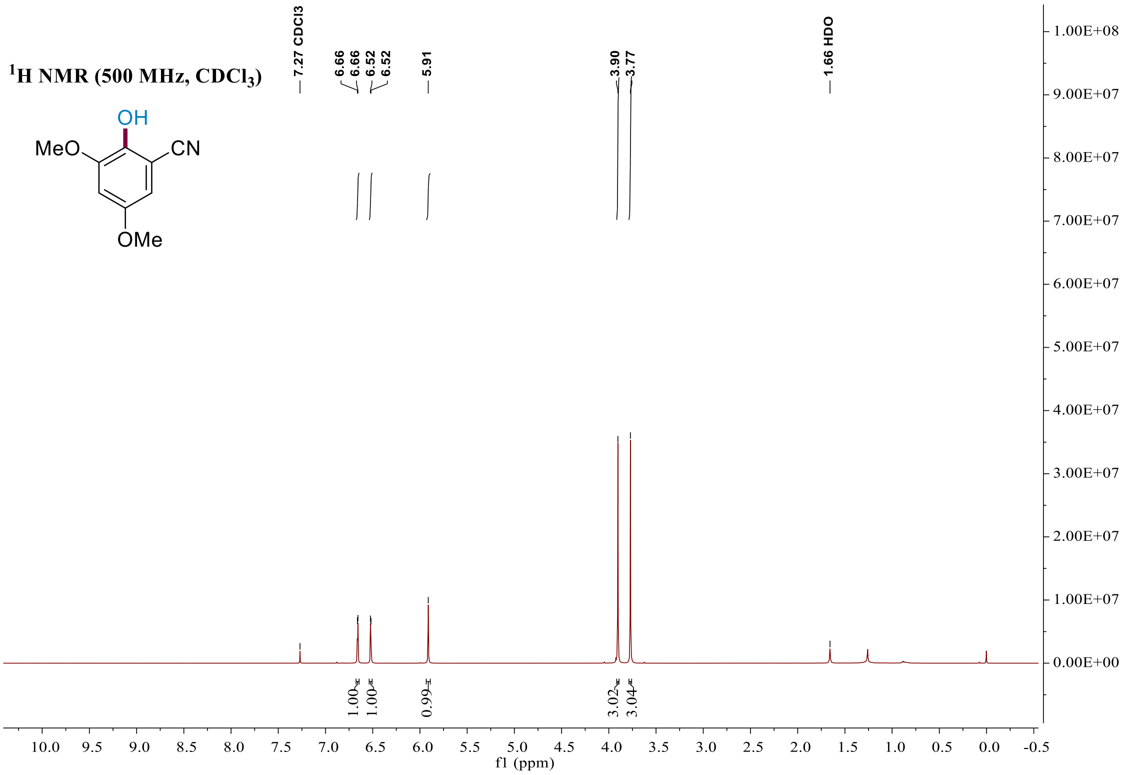

Supplementary Fig. 48. ^{1}H NMR spectra of compound **15** (500 MHz, rt, $CDCl_3$).

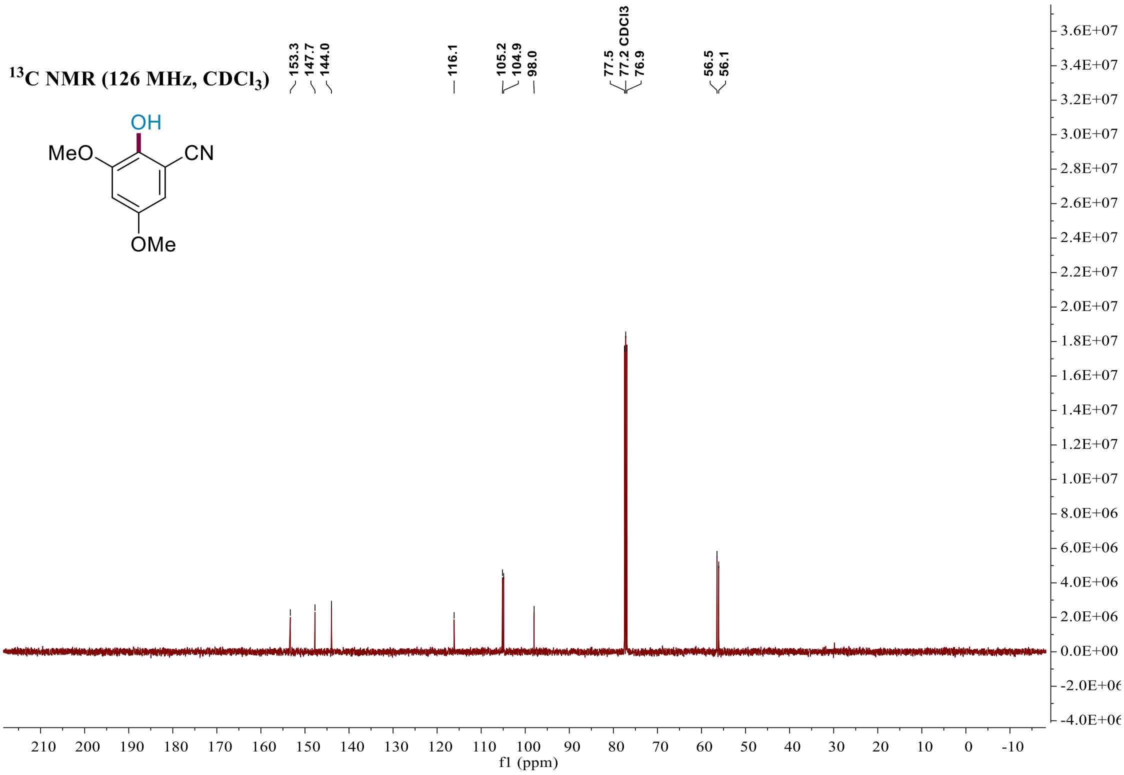

Supplementary Fig. 49. ^{13}C NMR spectra of compound **15** (126 MHz, rt, $CDCl_3$).

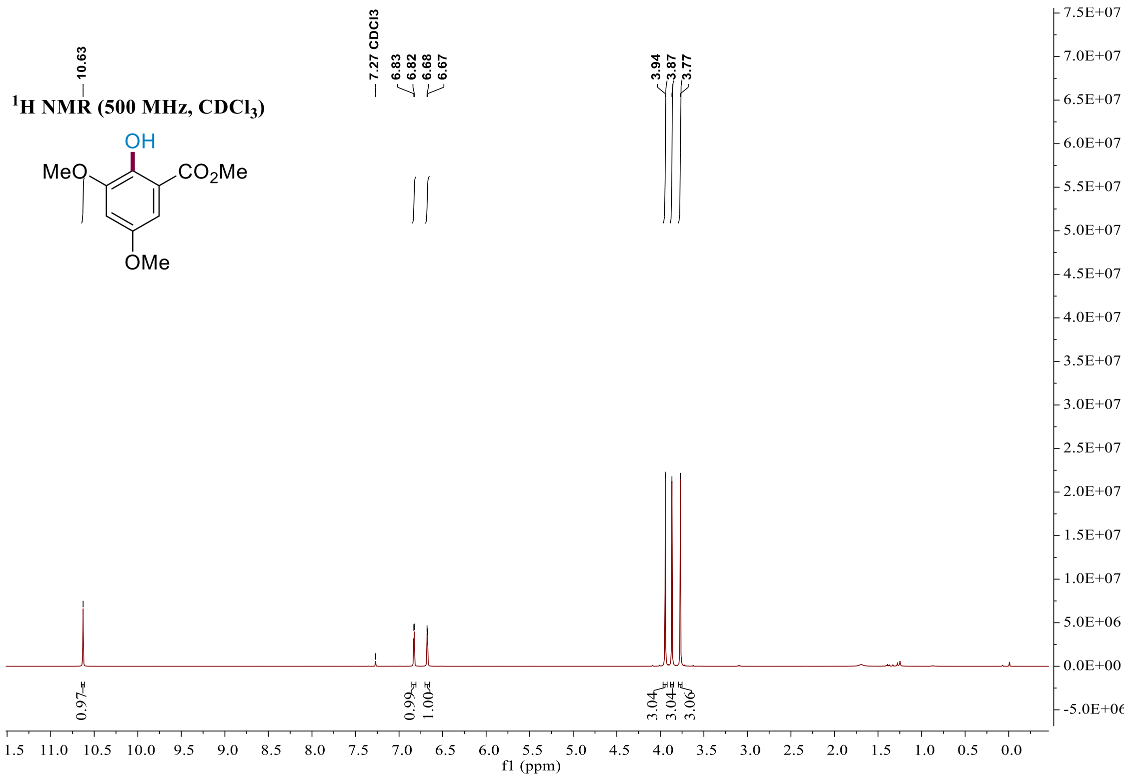

Supplementary Fig. 50. ^{1}H NMR spectra of compound **16** (500 MHz, rt, $CDCl_3$).

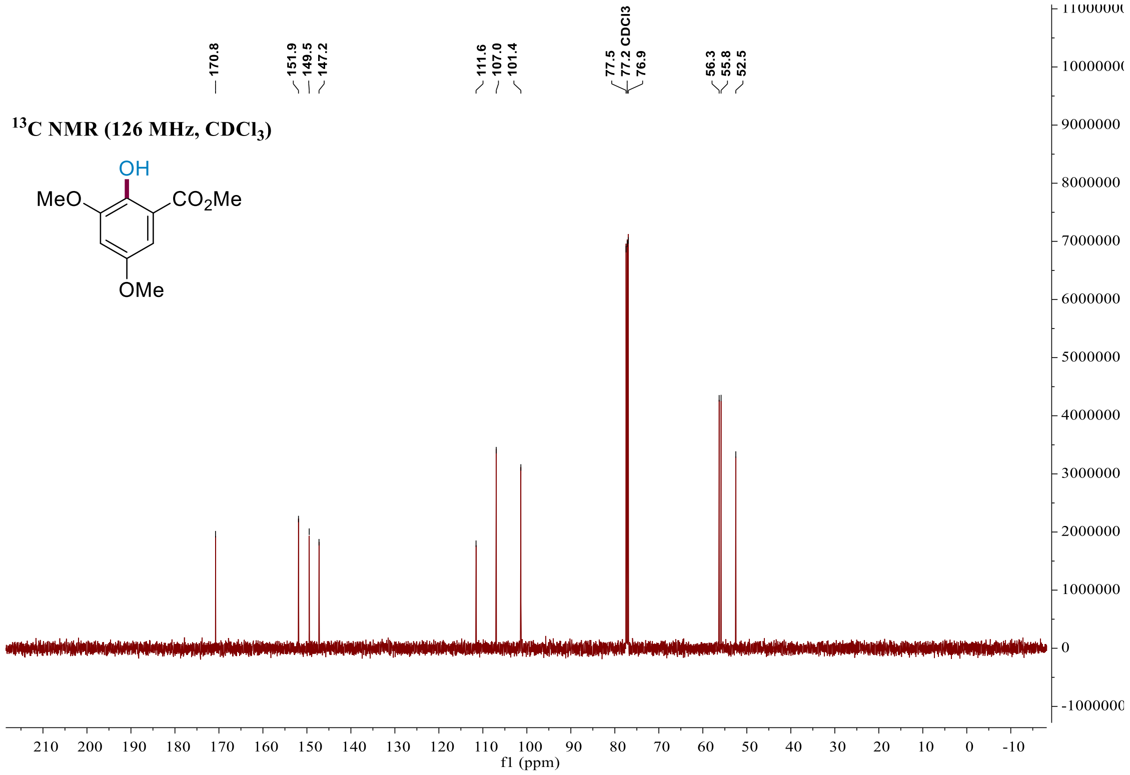

Supplementary Fig. 51. ^{13}C NMR spectra of compound **16** (126 MHz, rt, $CDCl_3$).

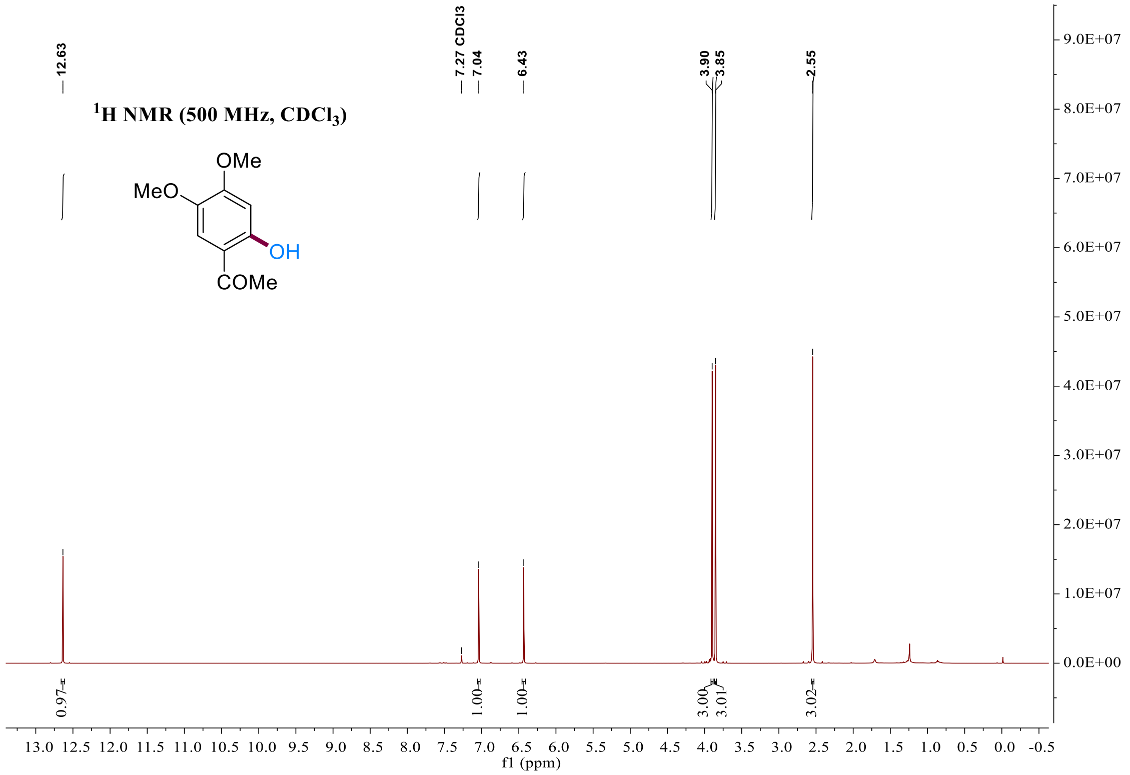

Supplementary Fig. 52. ^{1}H NMR spectra of compound **17** (500 MHz, rt, CDCl$_3$).

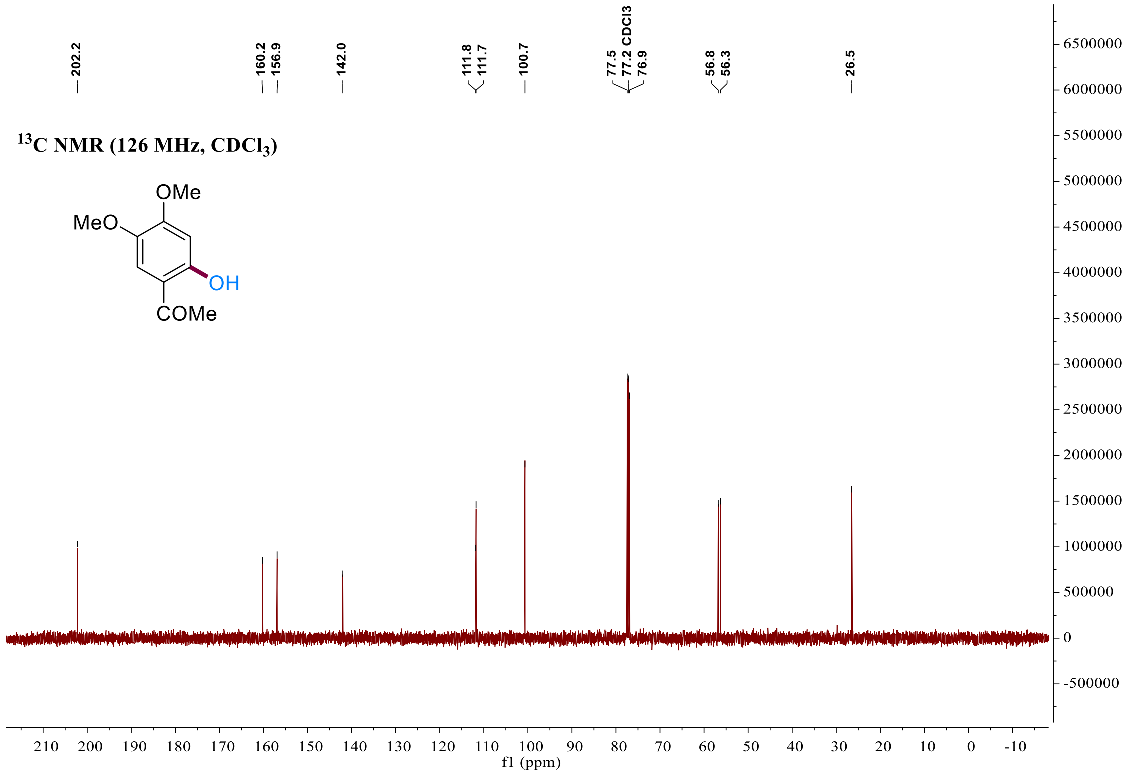

Supplementary Fig. 53. ^{13}C NMR spectra of compound **17** (126 MHz, rt, CDCl$_3$).

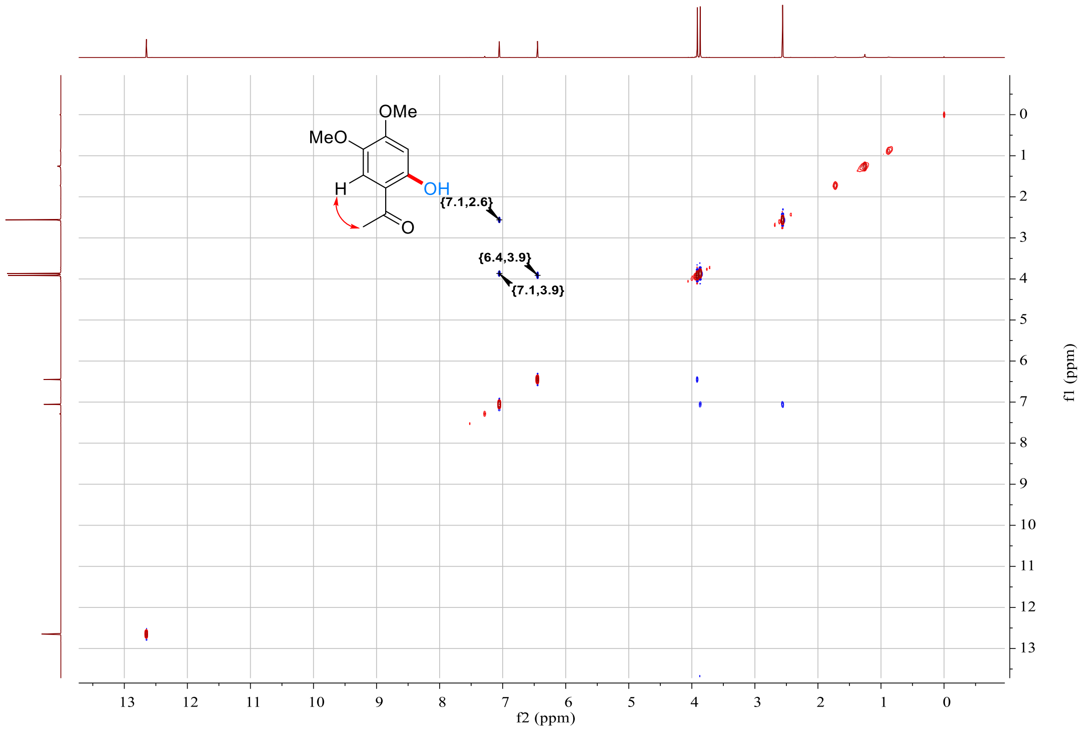

Supplementary Fig. 54. 2D NOESY spectra of compound **17** (500 MHz, rt, $CDCl_3$).

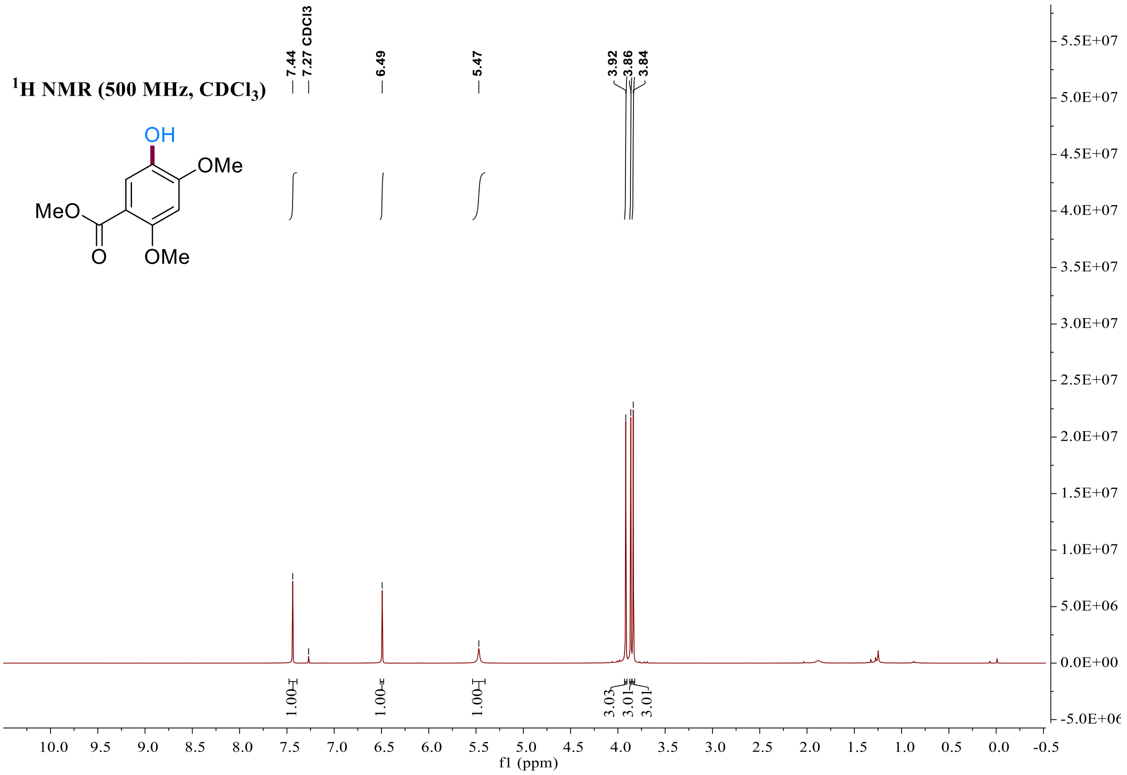

Supplementary Fig. 55. ^{1}H NMR spectra of compound **18** (500 MHz, rt, $CDCl_3$).

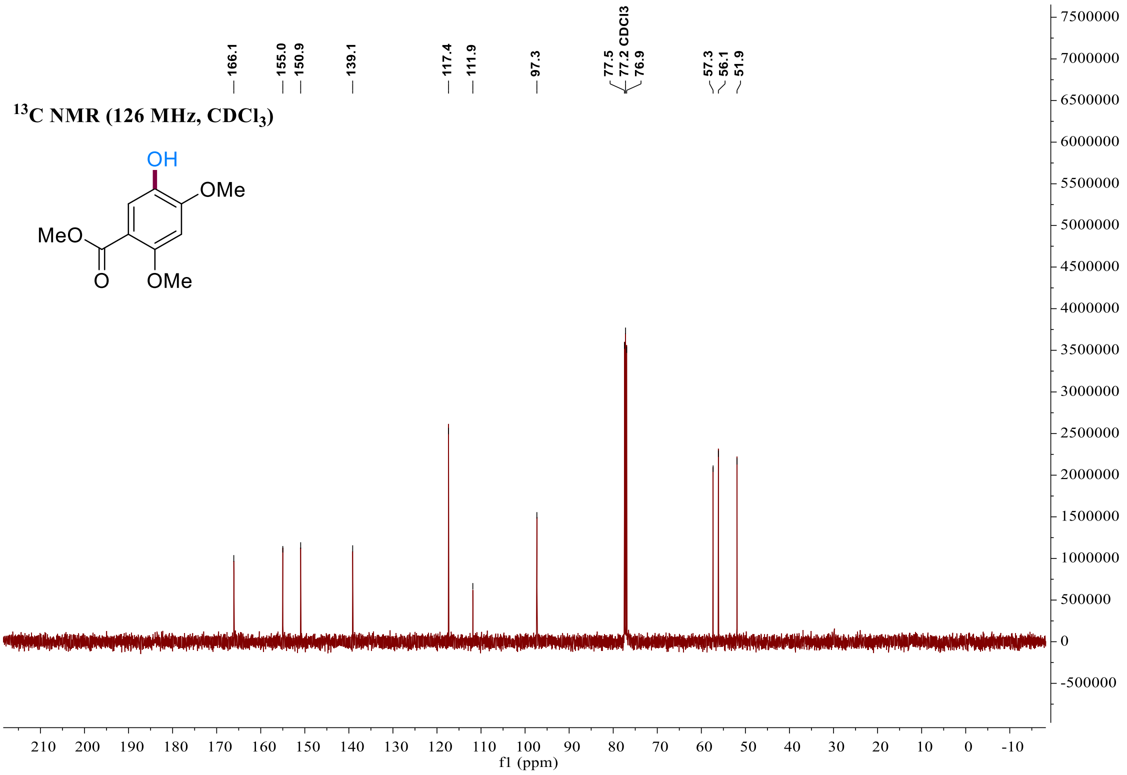

Supplementary Fig. 56. ^{13}C NMR spectra of compound **18** (126 MHz, rt, $CDCl_3$).

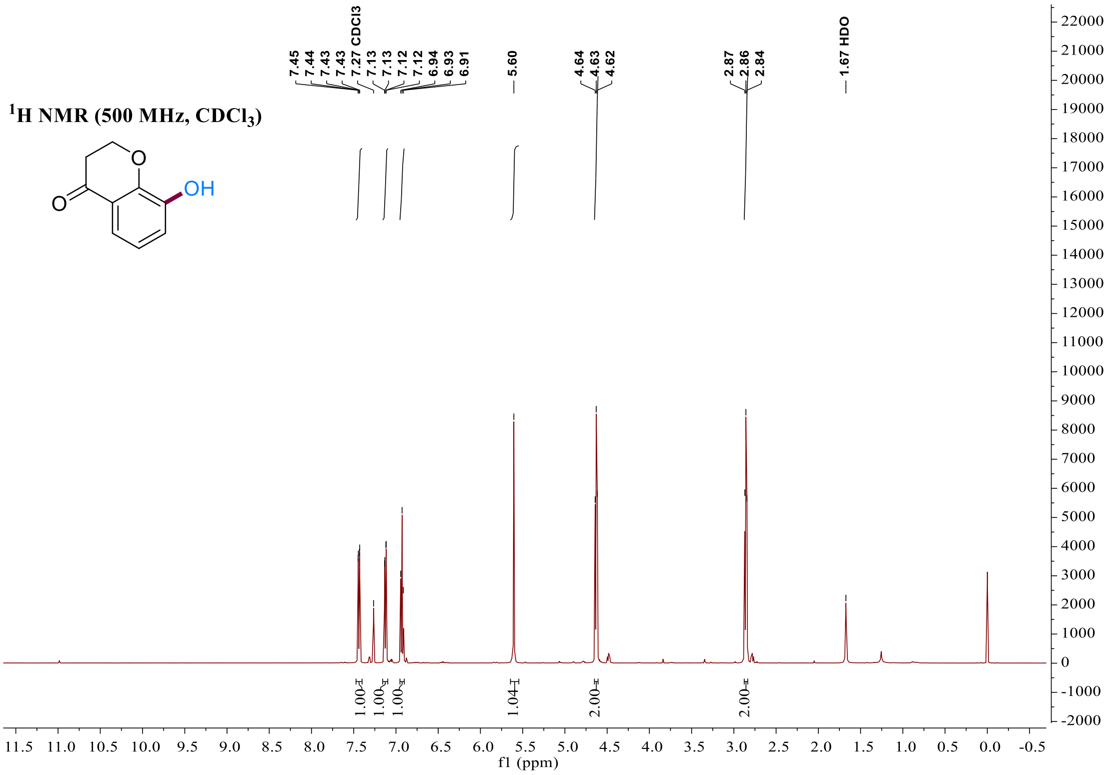

Supplementary Fig. 57. ^{1}H NMR spectra of compound **19** (500 MHz, rt, $CDCl_3$).

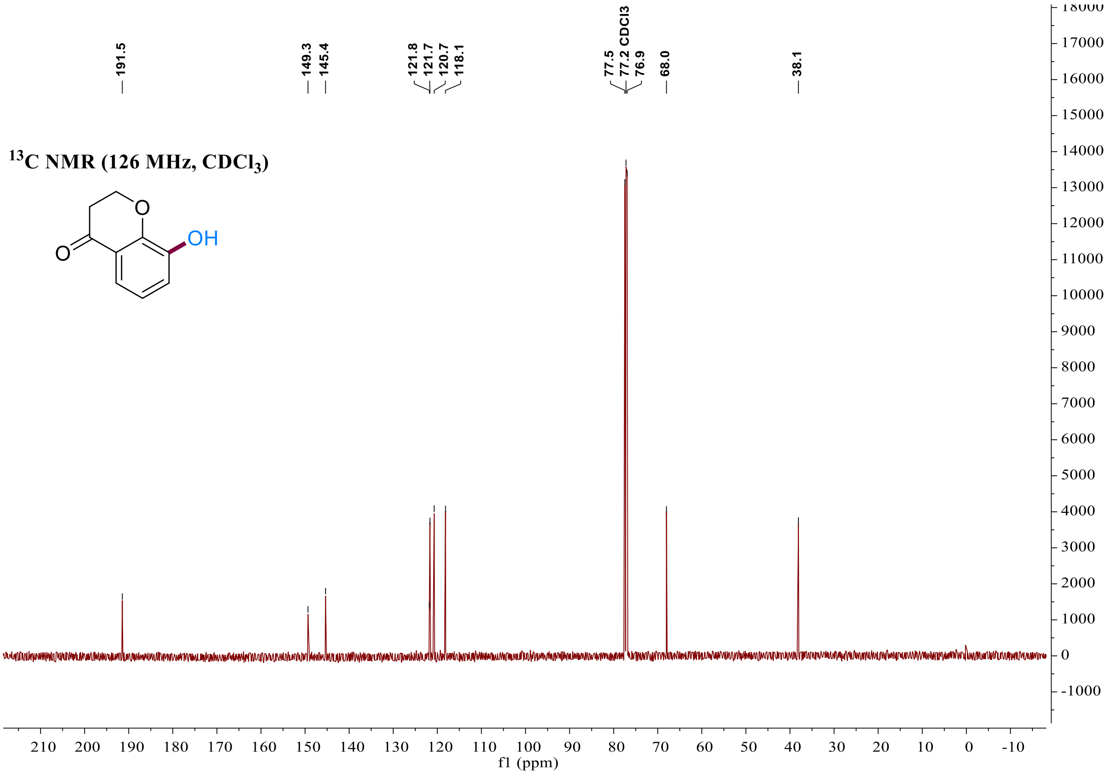

Supplementary Fig. 58. ^{13}C NMR spectra of compound **19** (126 MHz, rt, $CDCl_3$).

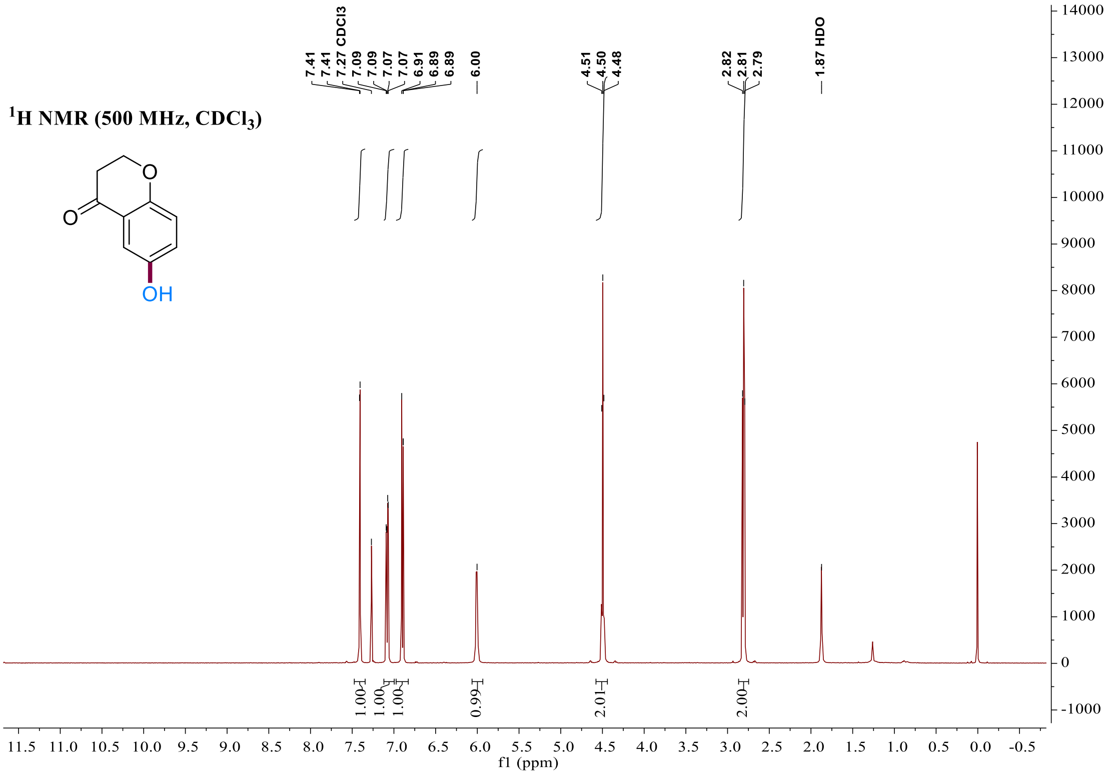

Supplementary Fig. 59. ^{1}H NMR spectra of compound **19** (500 MHz, rt, $CDCl_3$).

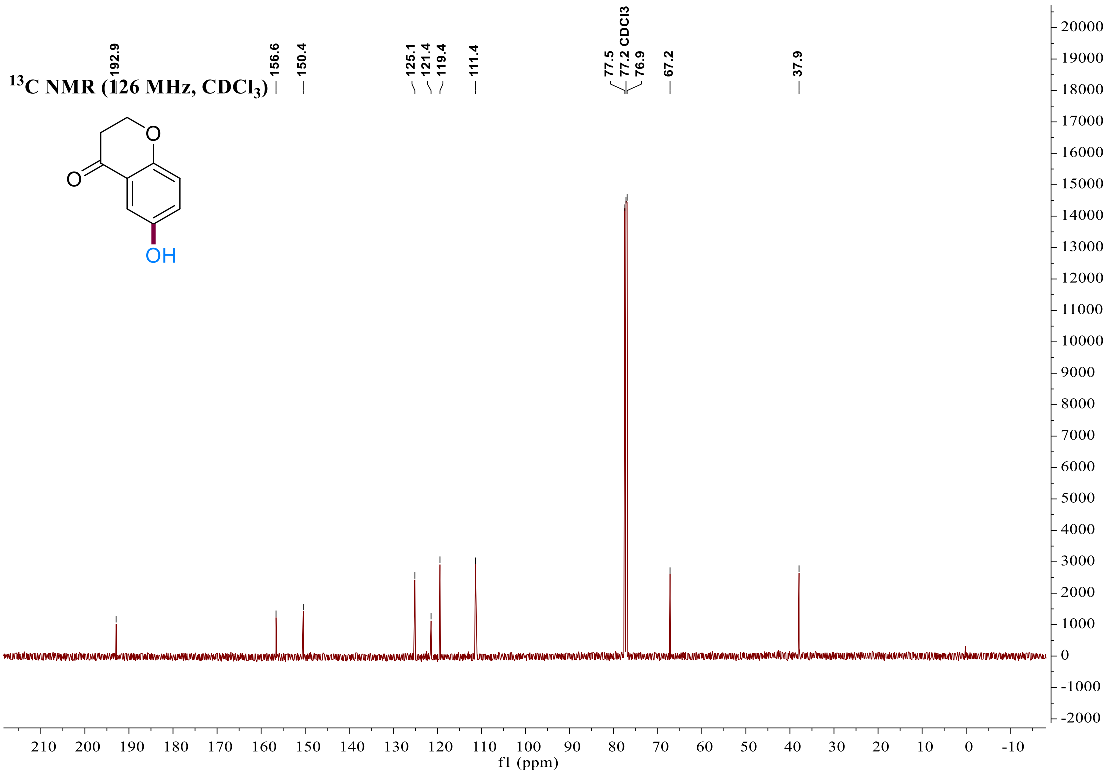

Supplementary Fig. 60. ^{13}C NMR spectra of compound **19** (126 MHz, rt, $CDCl_3$).

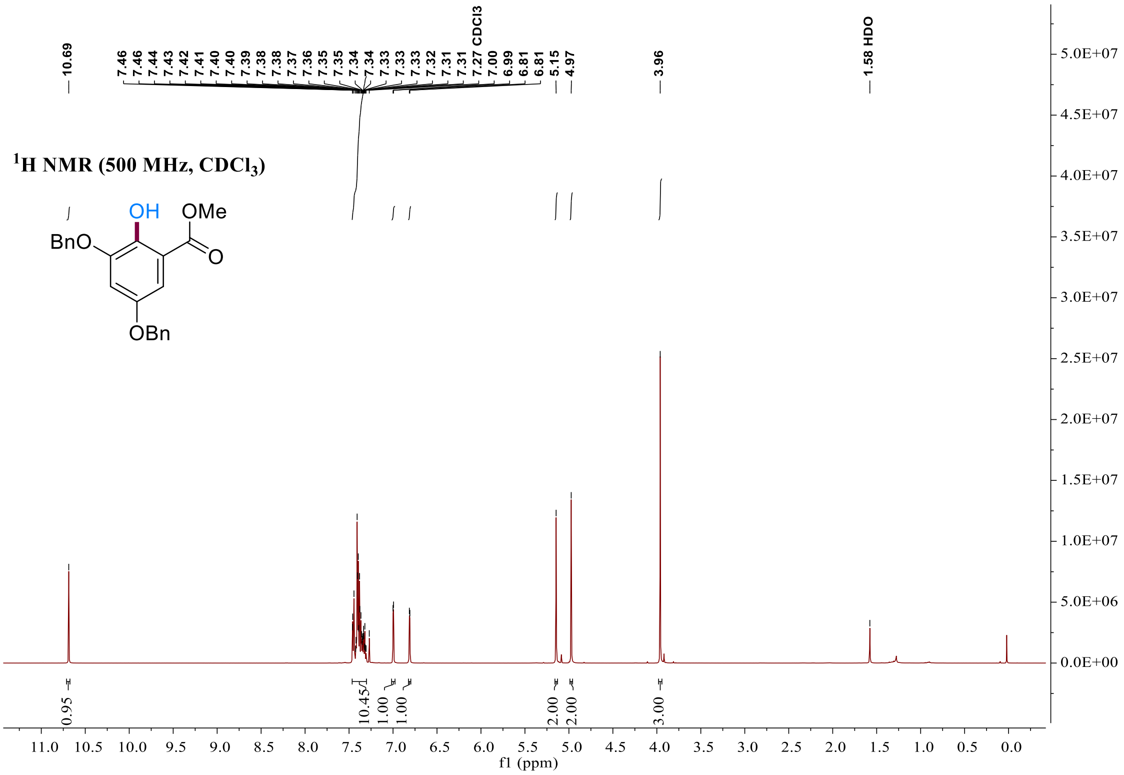

Supplementary Fig. 61. ^{1}H NMR spectra of compound **20** (500 MHz, rt, $CDCl_3$).

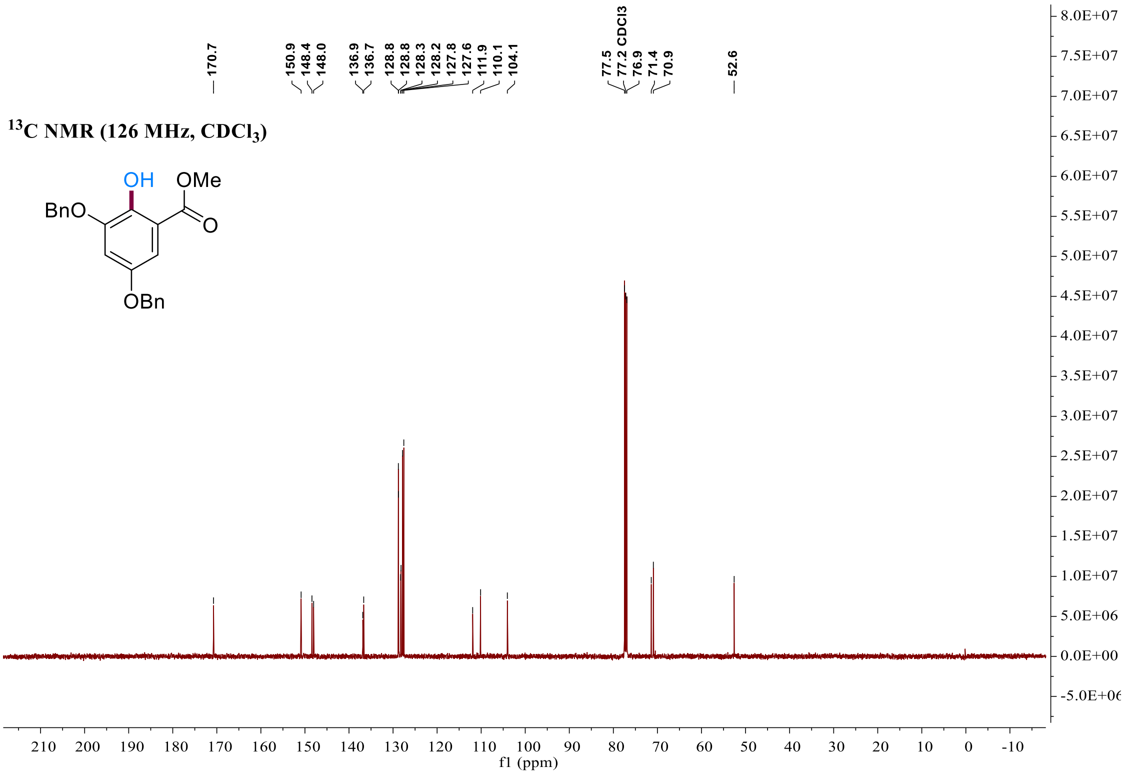

Supplementary Fig. 62. ^{13}C NMR spectra of compound **20** (126 MHz, rt, $CDCl_3$).

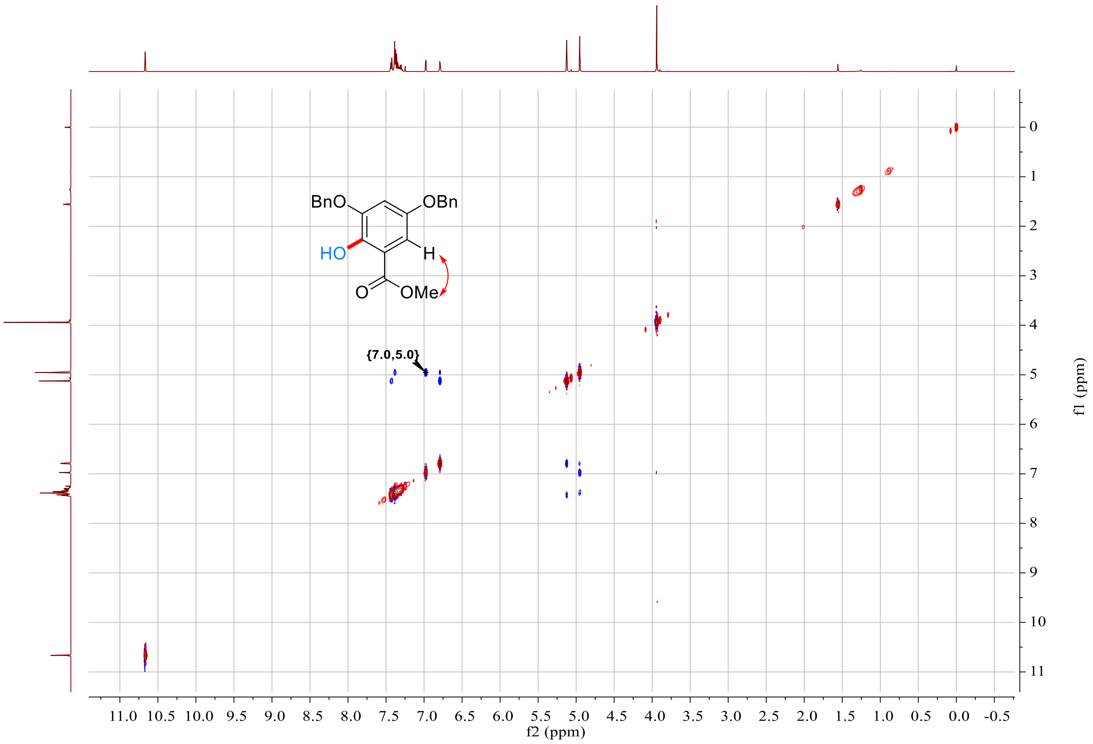

Supplementary Fig. 63. 2D NOESY spectra of compound **20** (500 MHz, rt, $CDCl_3$).

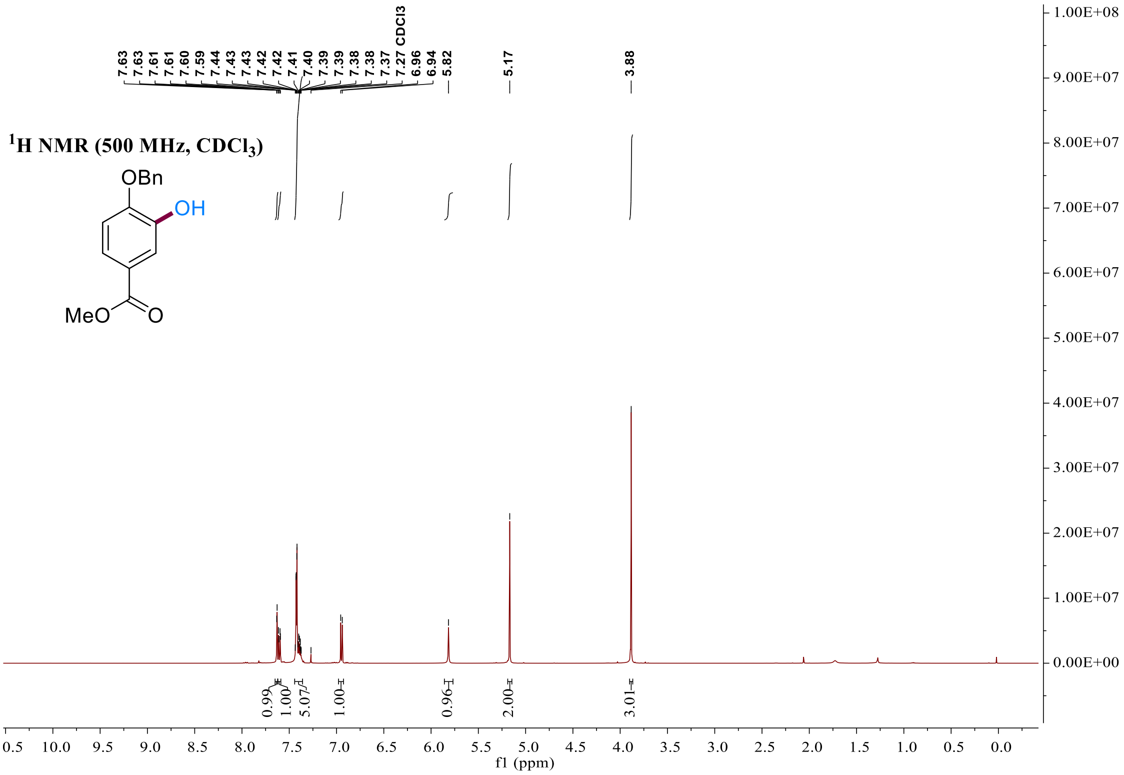

Supplementary Fig. 64. ^{1}H NMR spectra of compound **21** (500 MHz, rt, $CDCl_3$).

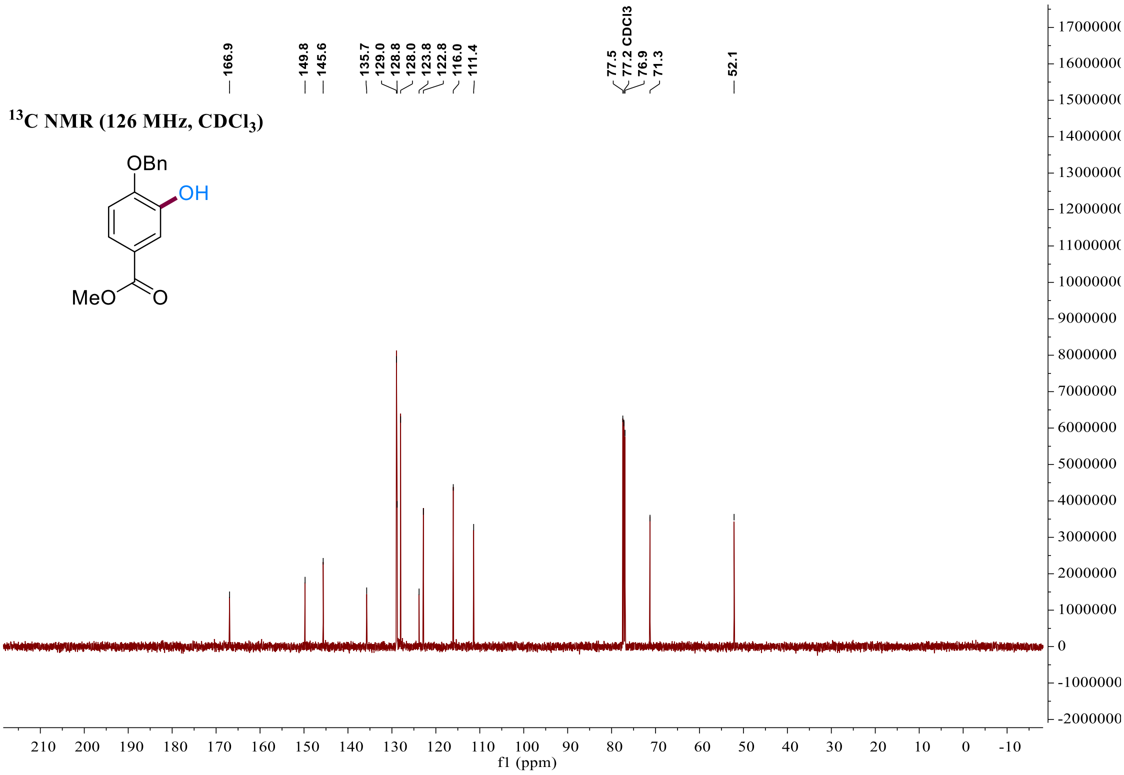

Supplementary Fig. 65. ^{13}C NMR spectra of compound **21** (126 MHz, rt, $CDCl_3$).

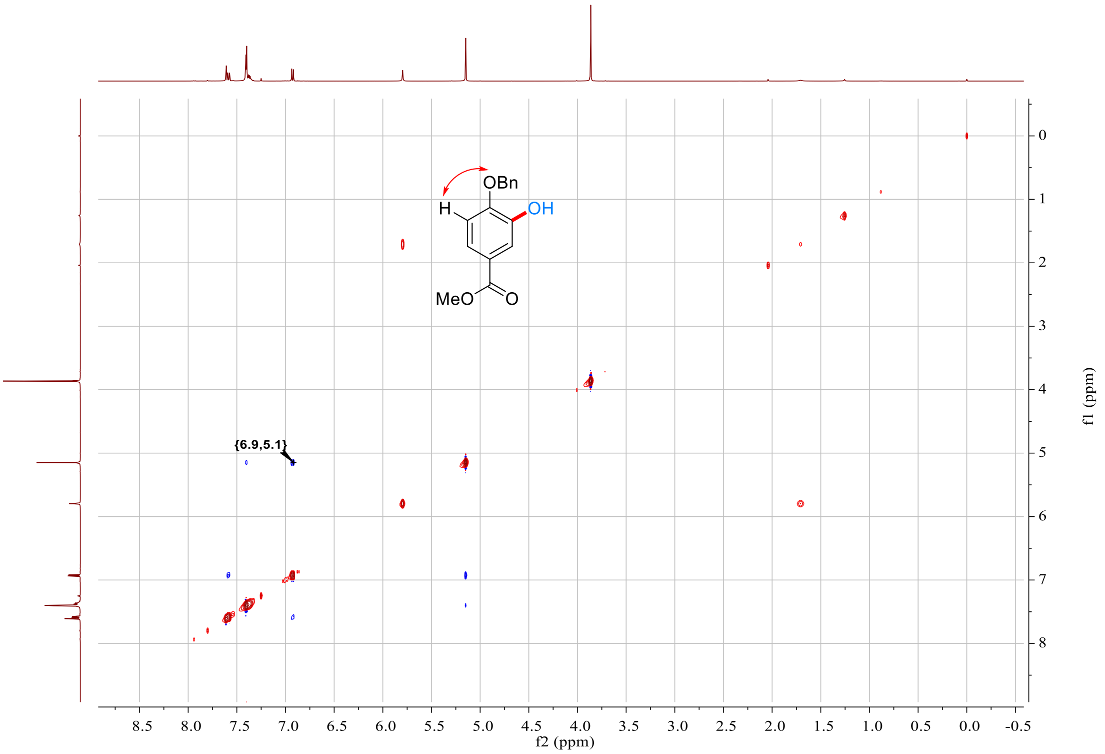

Supplementary Fig. 66. 2D NOESY spectra of compound **21** (500 MHz, rt, $CDCl_3$).

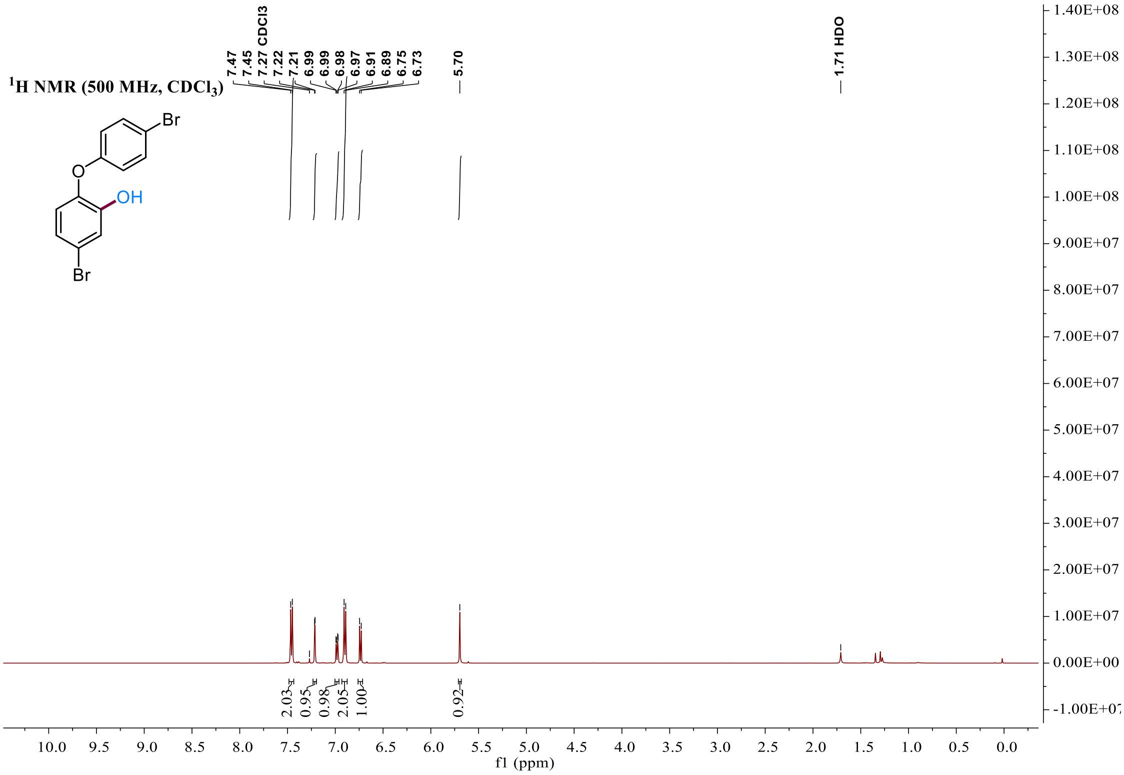

Supplementary Fig. 67. ^{1}H NMR spectra of compound **22** (500 MHz, rt, CDCl$_3$).

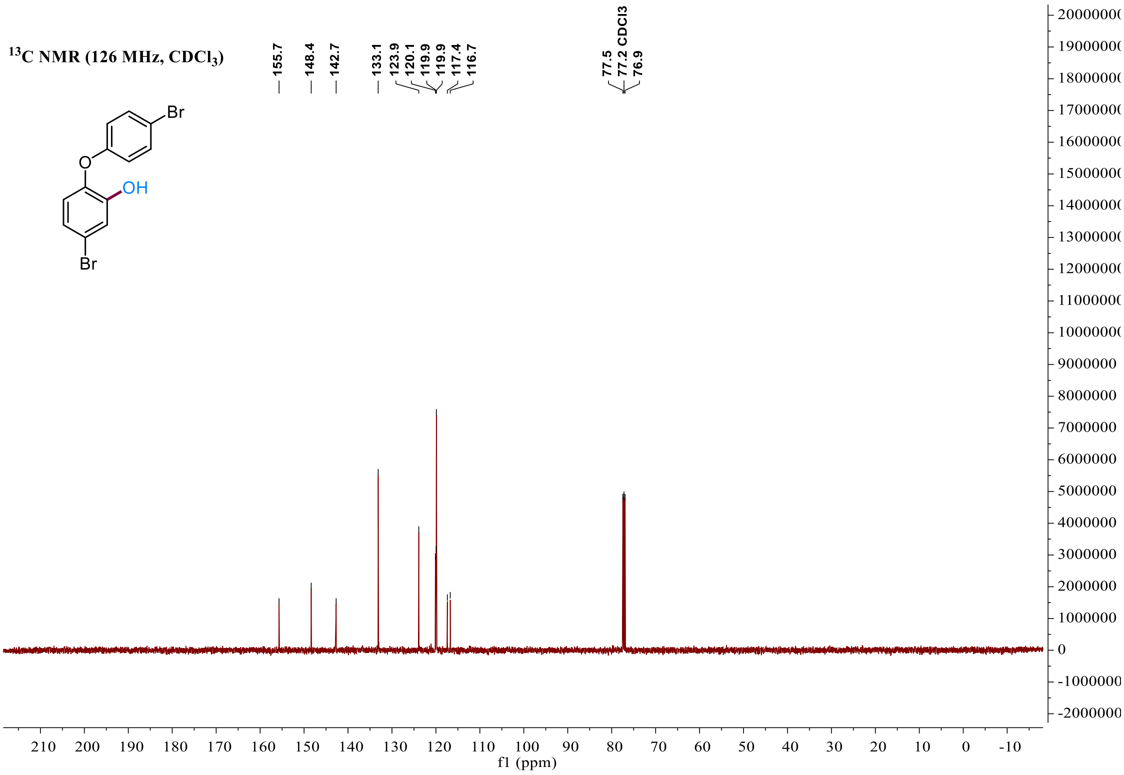

Supplementary Fig. 68. ^{13}C NMR spectra of compound **22** (126 MHz, rt, CDCl$_3$).

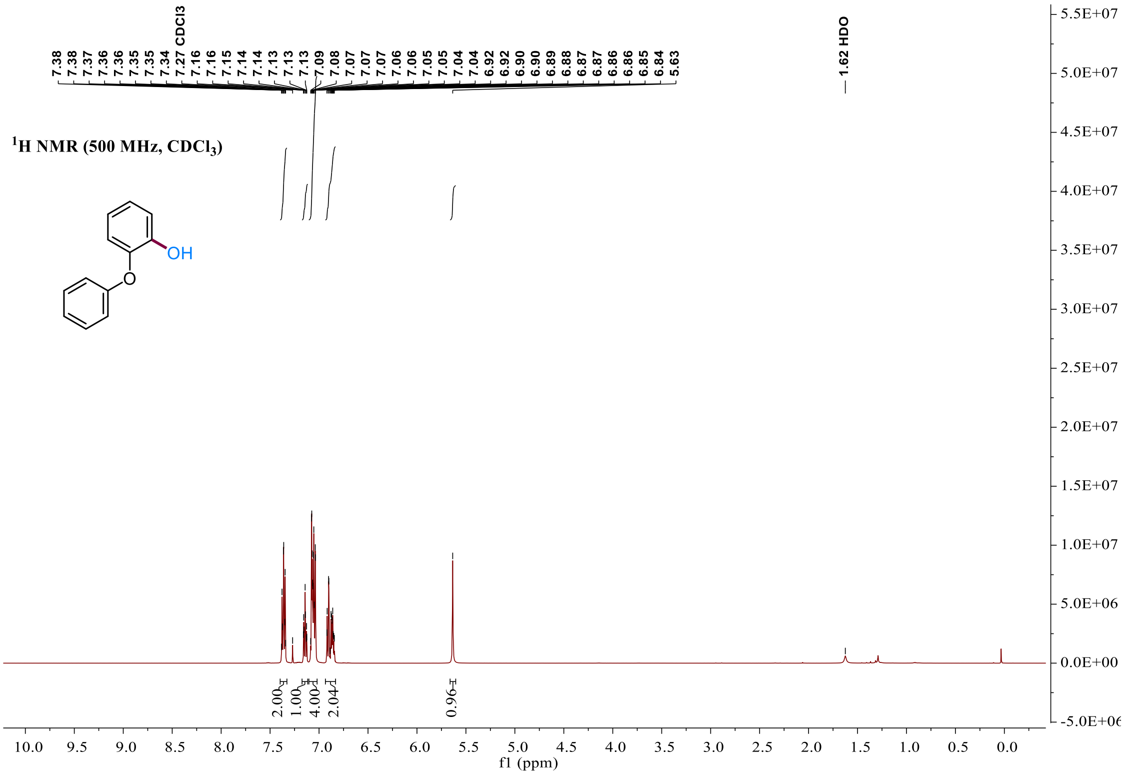

Supplementary Fig. 69. ^{1}H NMR spectra of compound **23** (500 MHz, rt, $CDCl_3$).

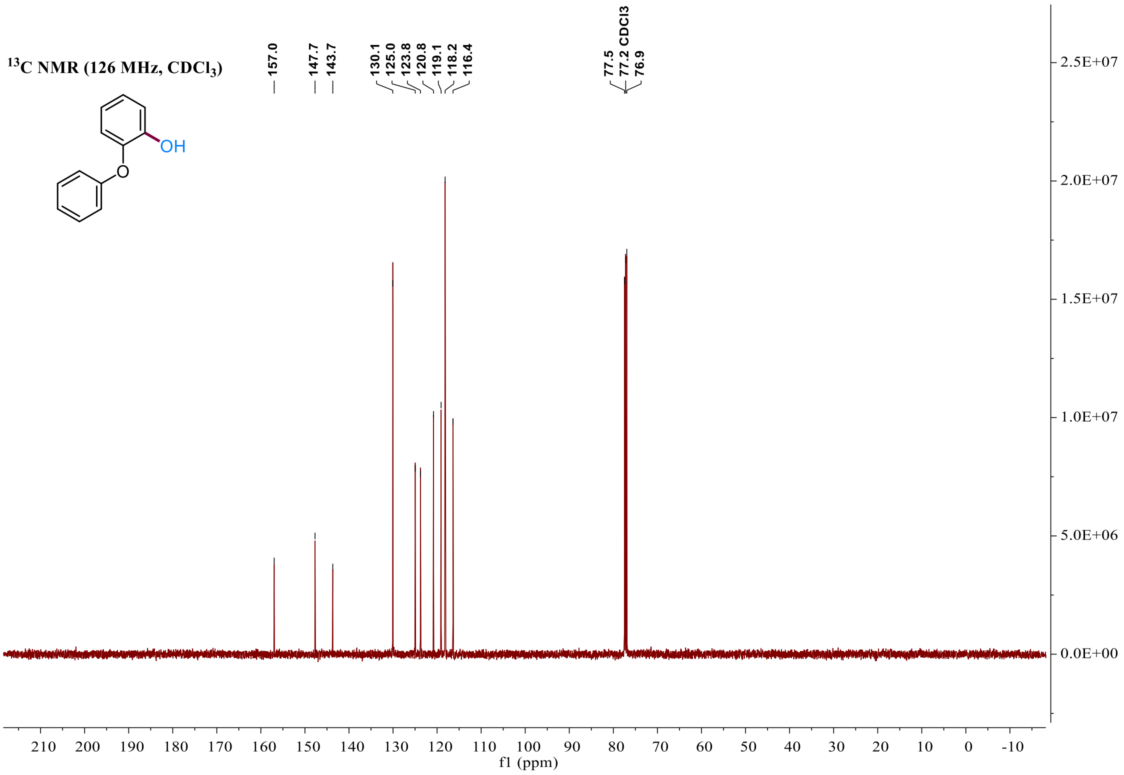

Supplementary Fig. 70. ^{13}C NMR spectra of compound **23** (126 MHz, rt, $CDCl_3$).

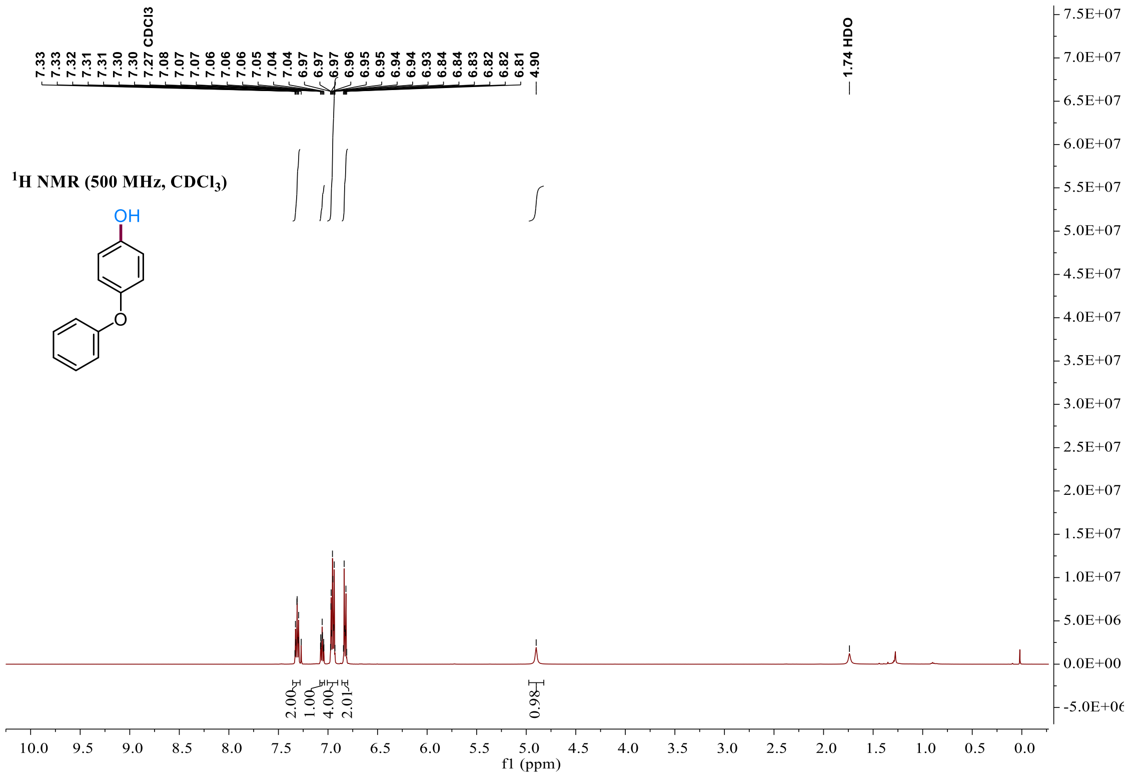

Supplementary Fig. 71. ^{1}H NMR spectra of compound **23** (500 MHz, rt, $CDCl_3$).

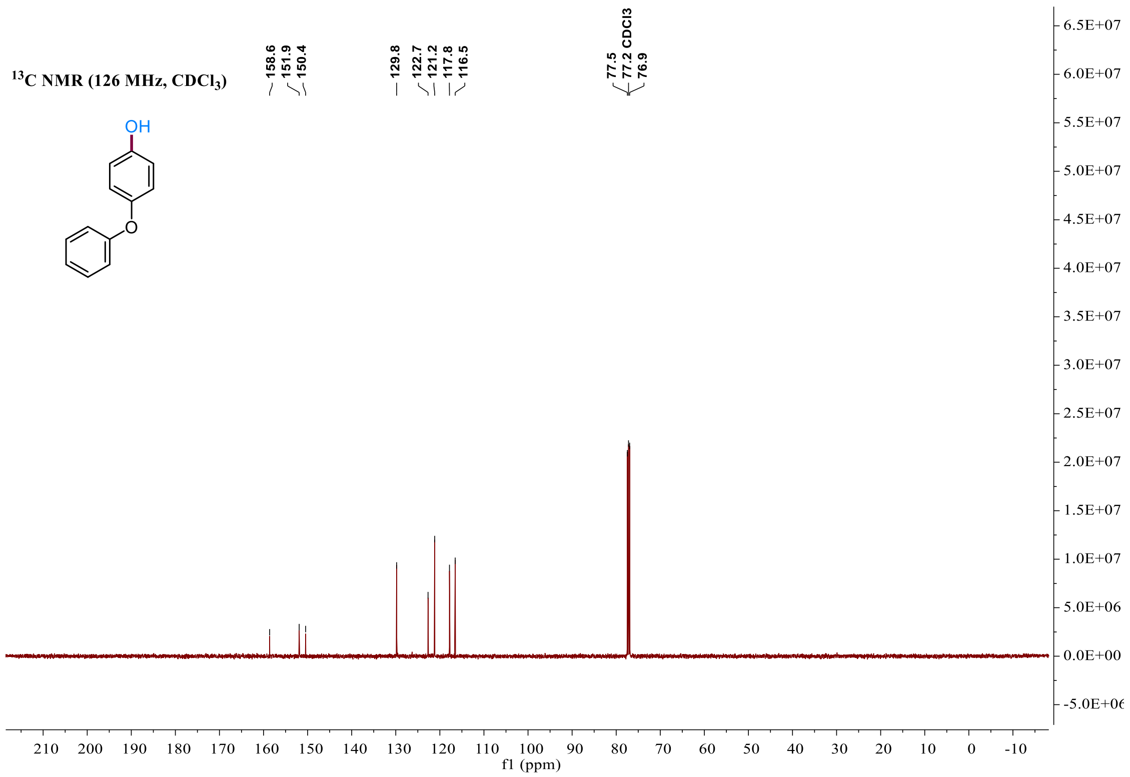

Supplementary Fig. 72. ^{13}C NMR spectra of compound **23** (126 MHz, rt, $CDCl_3$).

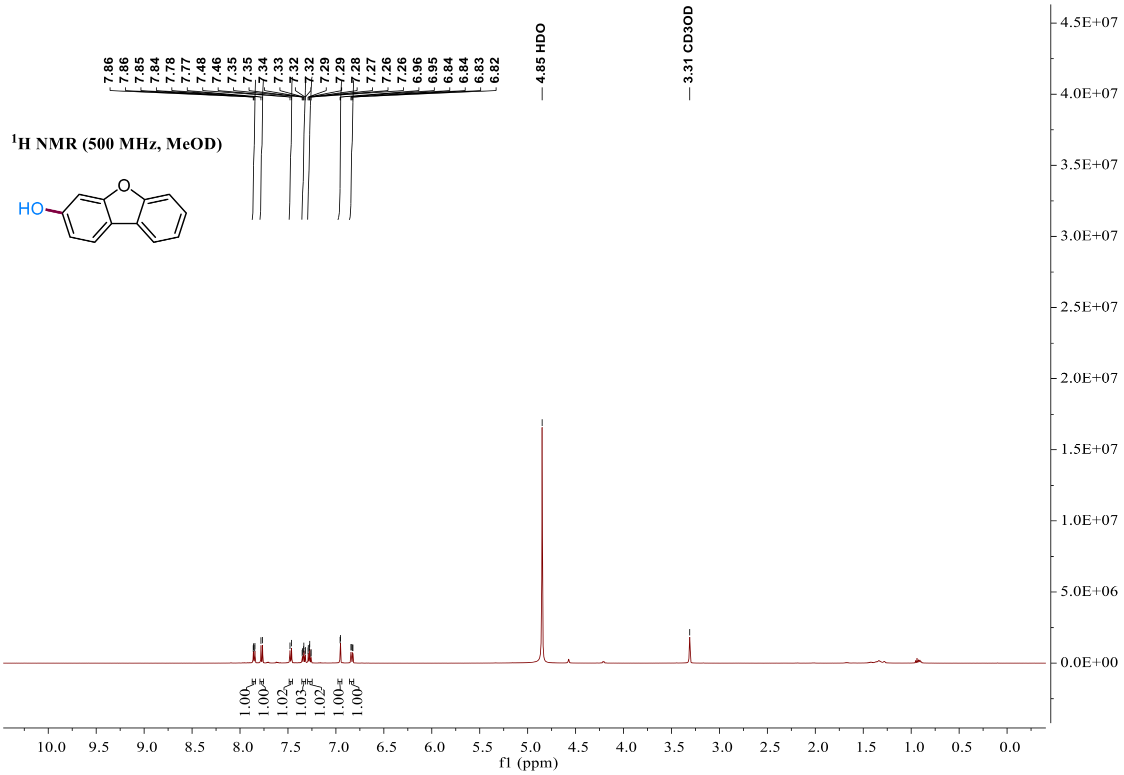

Supplementary Fig. 73. ^{1}H NMR spectra of compound **24** (500 MHz, rt, MeOD).

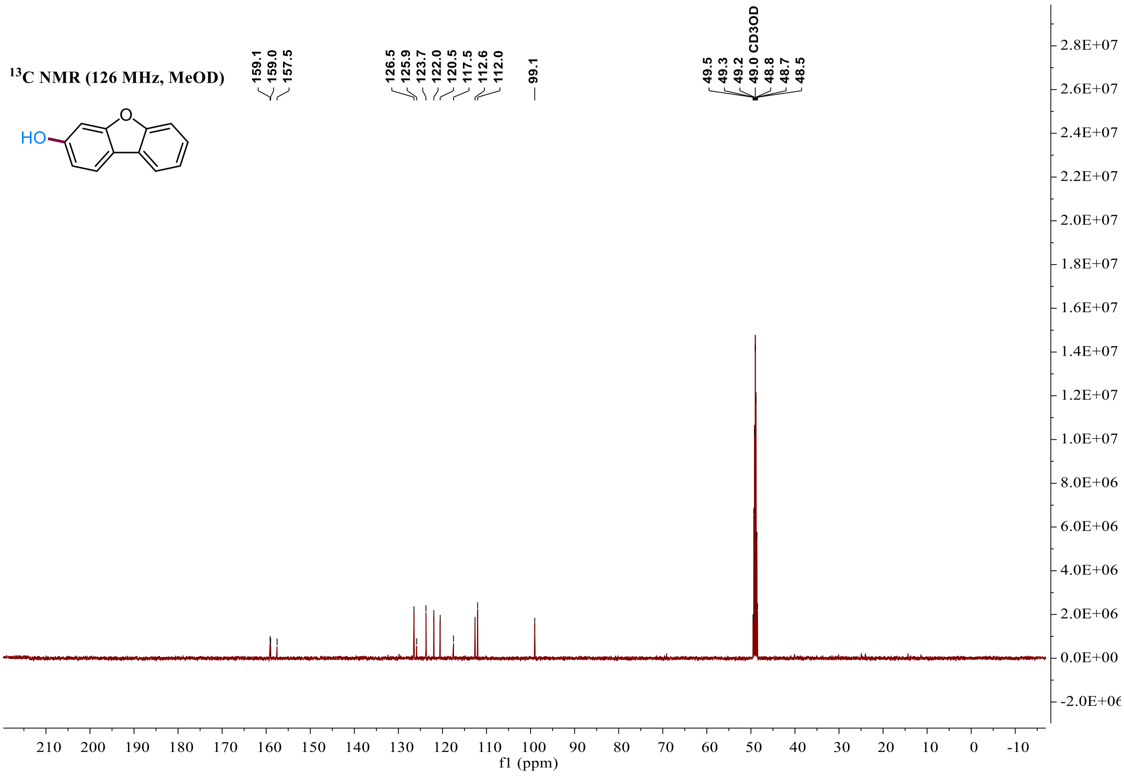

Supplementary Fig. 74. ^{13}C NMR spectra of compound **24** (126 MHz, rt, MeOD).

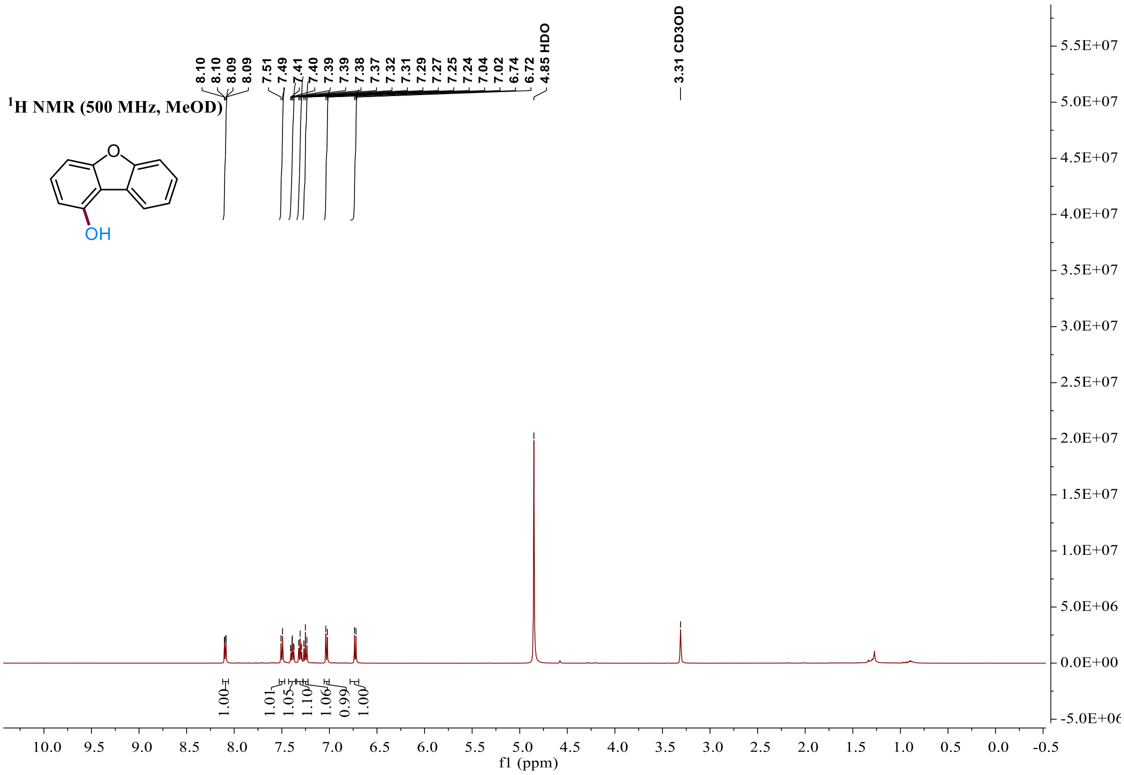

Supplementary Fig. 75. ^{1}H NMR spectra of compound **24** (500 MHz, rt, MeOD).

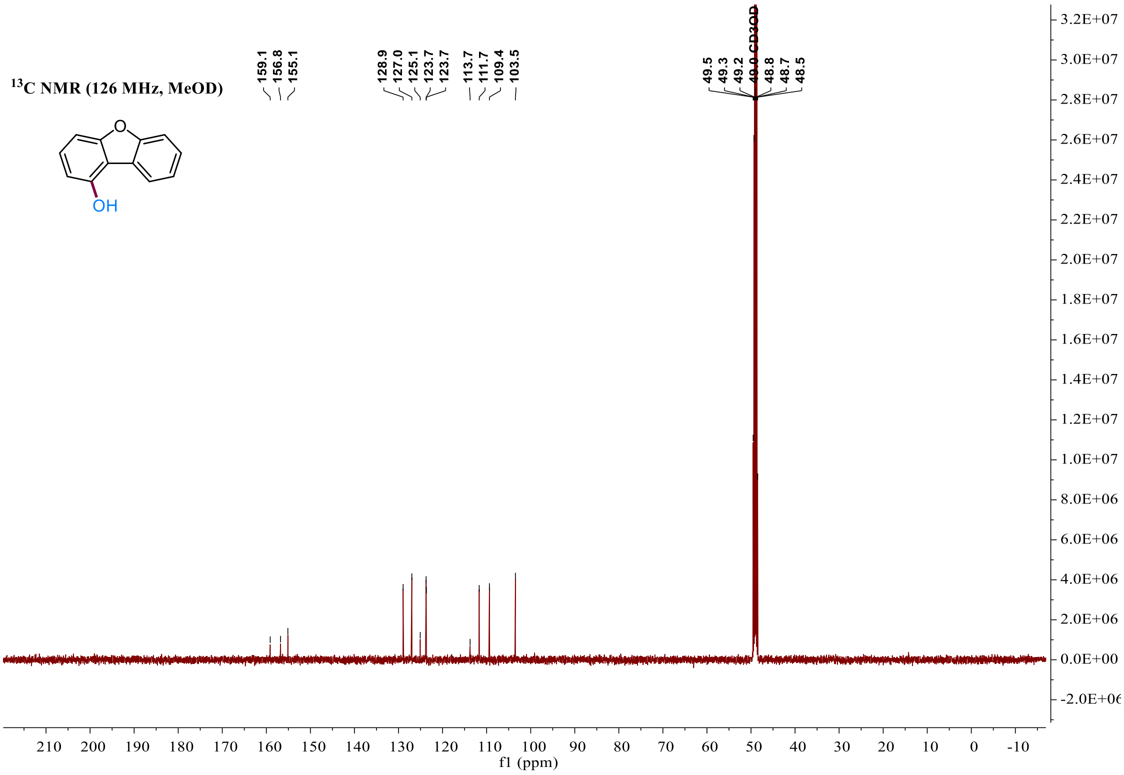

Supplementary Fig. 76. ^{13}C NMR spectra of compound **24** (126 MHz, rt, MeOD).

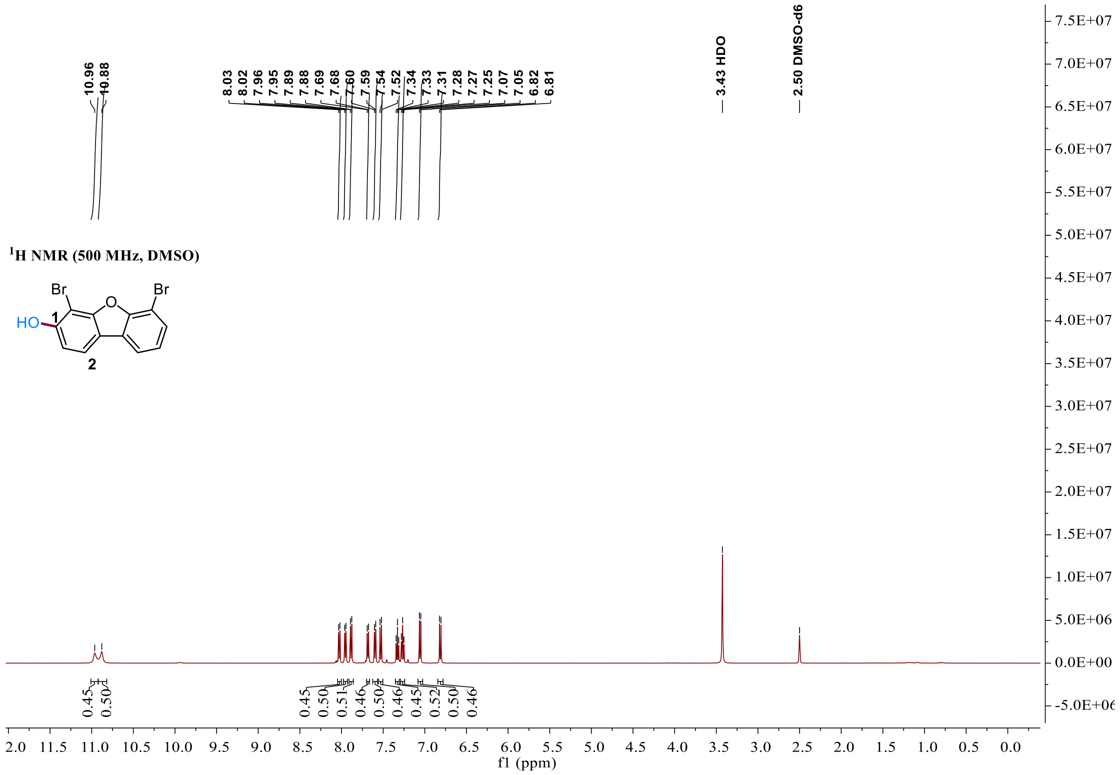

Supplementary Fig. 77. ^{1}H NMR spectra of compound **25** (500 MHz, rt, DMSO).

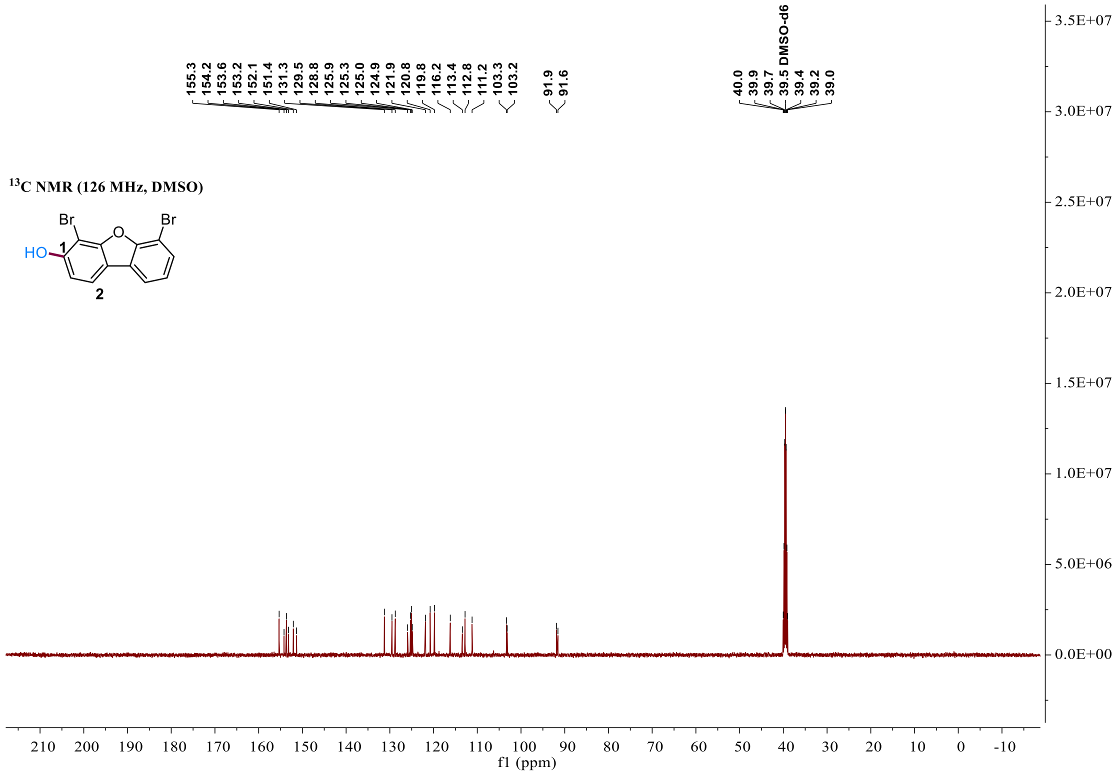

Supplementary Fig. 78. ^{13}C NMR spectra of compound **25** (126 MHz, rt, DMSO).

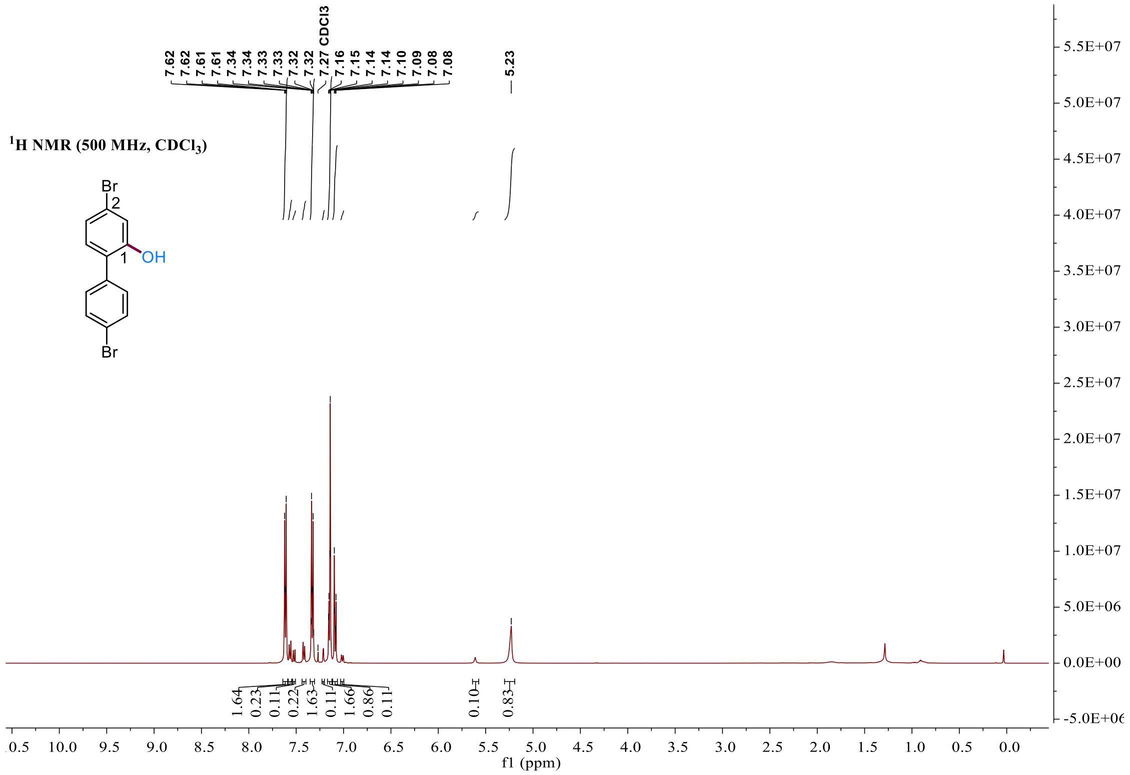

Supplementary Fig. 79. ^{1}H NMR spectra of compound **26** (500 MHz, rt, $CDCl_3$).

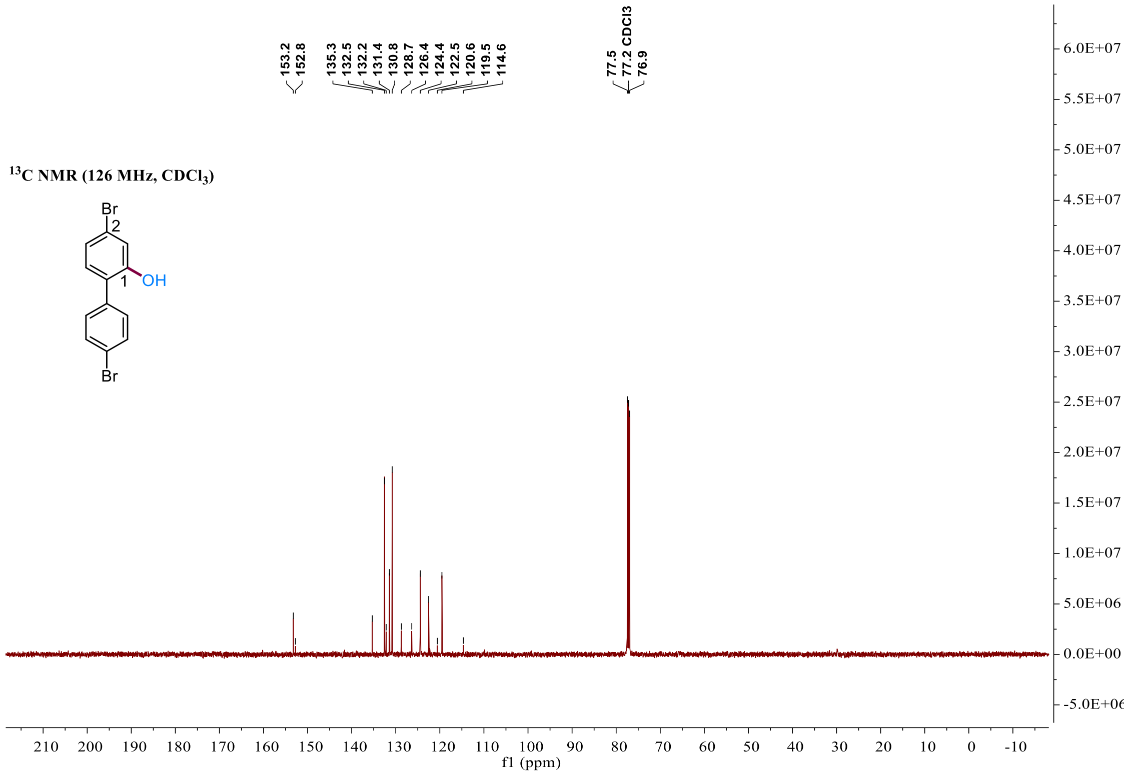

Supplementary Fig. 80. ^{13}C NMR spectra of compound **26** (126 MHz, rt, $CDCl_3$).

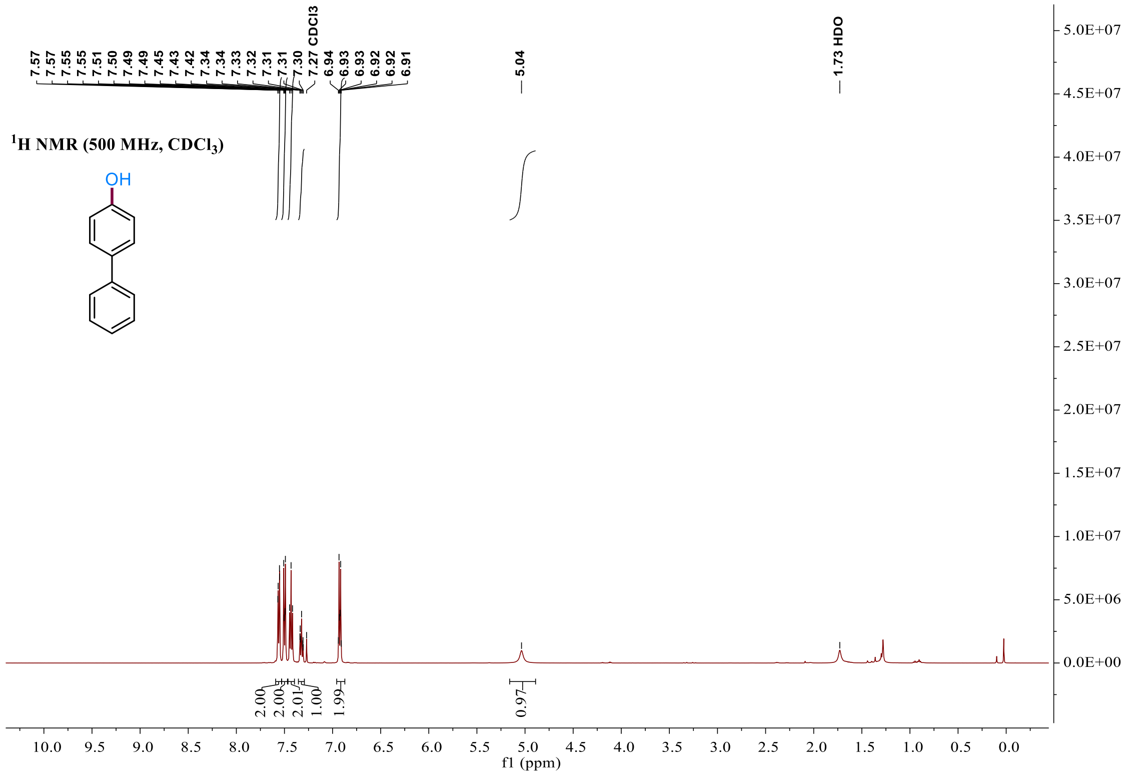

Supplementary Fig. 81. ^{1}H NMR spectra of compound **27** (500 MHz, rt, $CDCl_3$).

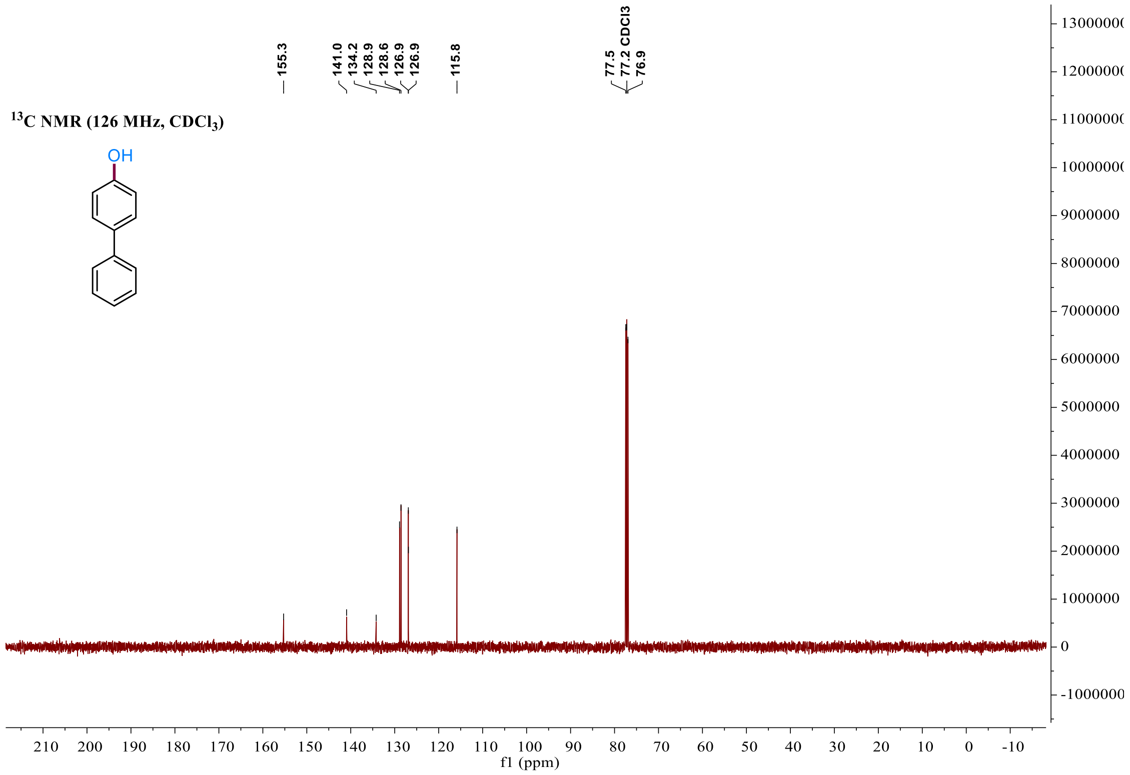

Supplementary Fig. 82. ^{13}C NMR spectra of compound **27** (126 MHz, rt, $CDCl_3$).

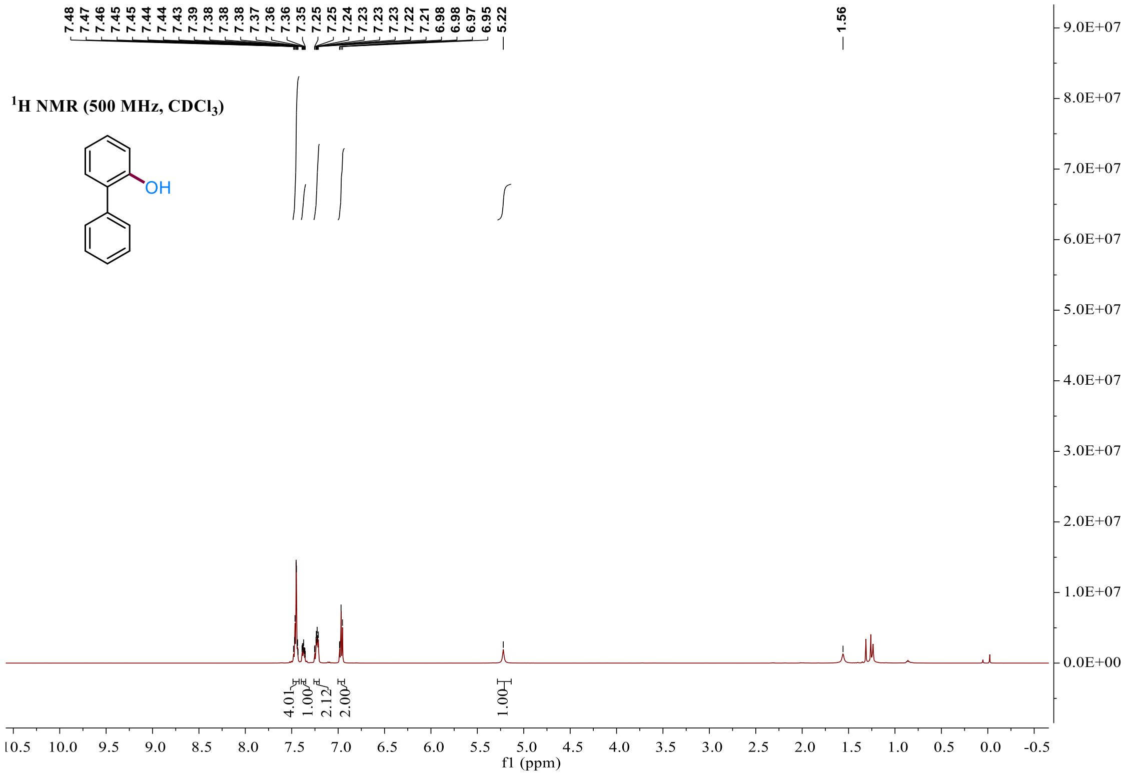

Supplementary Fig. 83. ^{1}H NMR spectra of compound **27** (500 MHz, rt, CDCl$_3$).

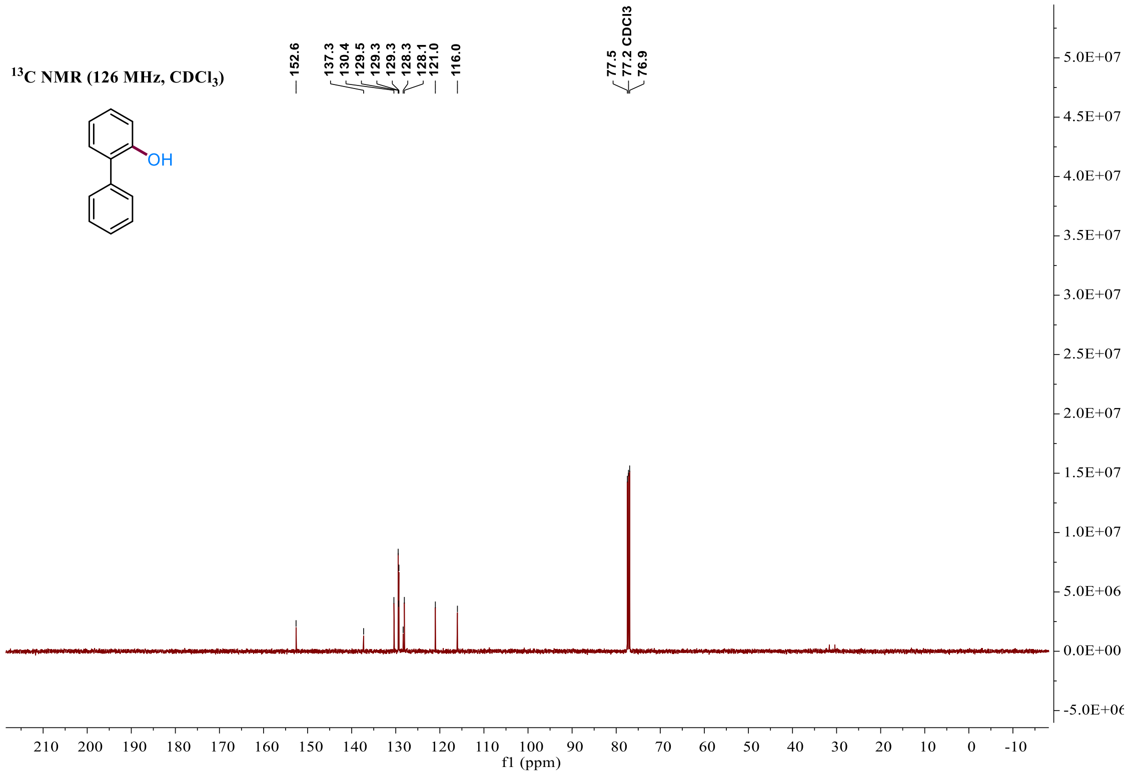

Supplementary Fig. 84. ^{13}C NMR spectra of compound **27** (126 MHz, rt, CDCl$_3$).

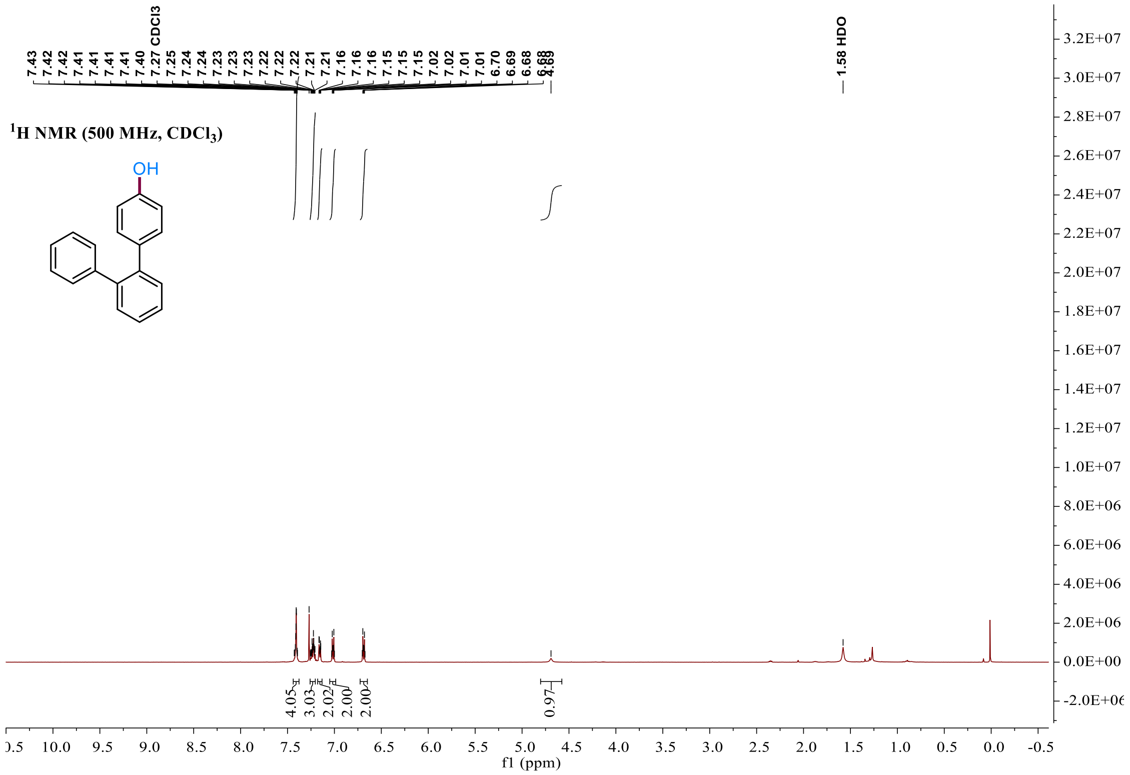

Supplementary Fig. 85. ^{1}H NMR spectra of compound **28** (500 MHz, rt, $CDCl_3$).

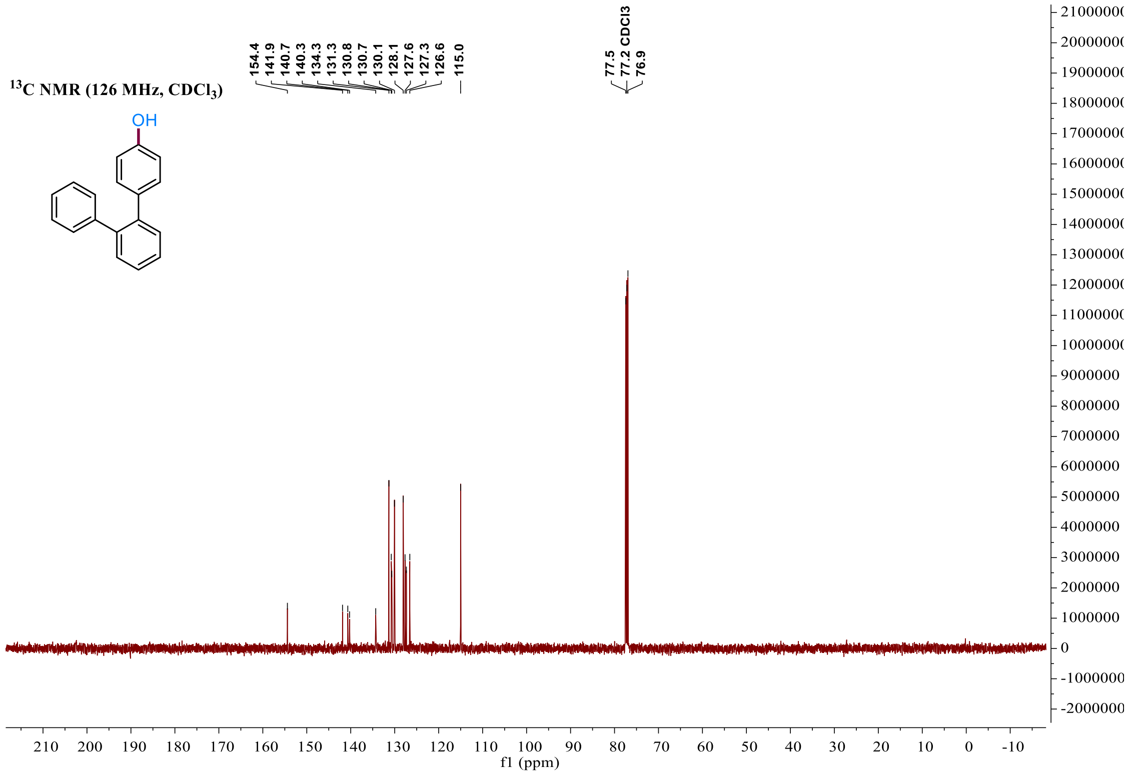

Supplementary Fig. 86. ^{13}C NMR spectra of compound **28** (126 MHz, rt, $CDCl_3$).

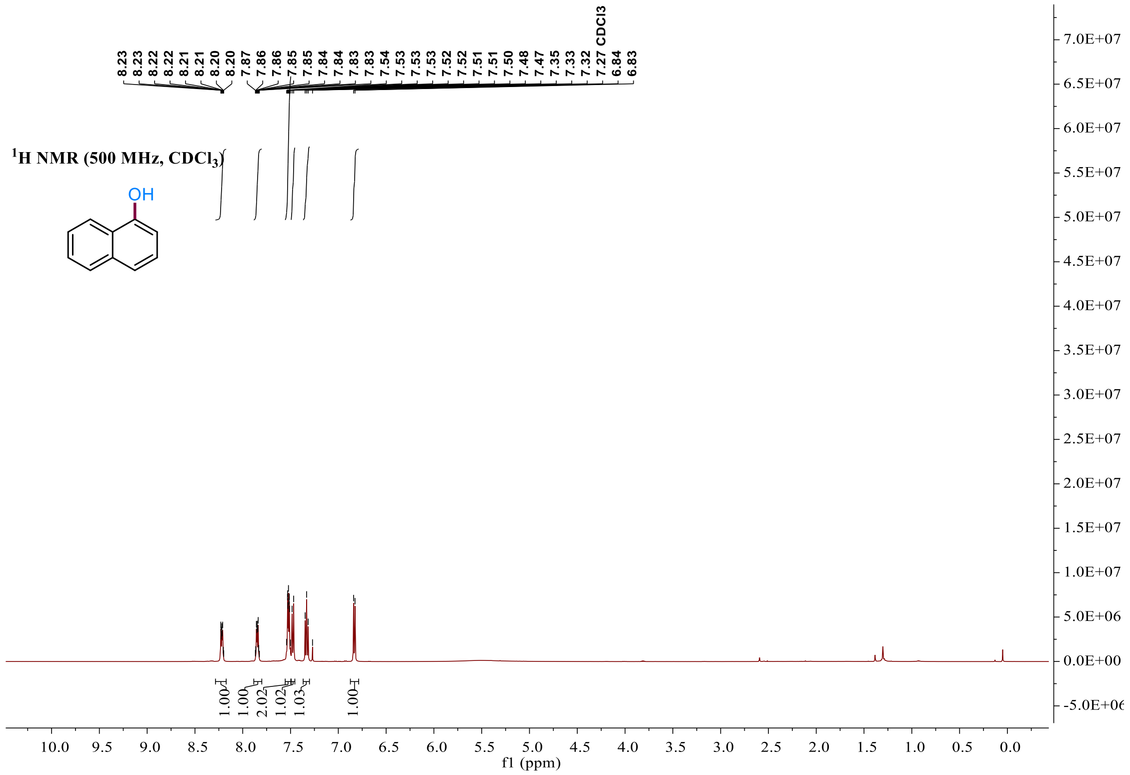

Supplementary Fig. 87. ^{1}H NMR spectra of compound **29** (500 MHz, rt, $CDCl_3$).

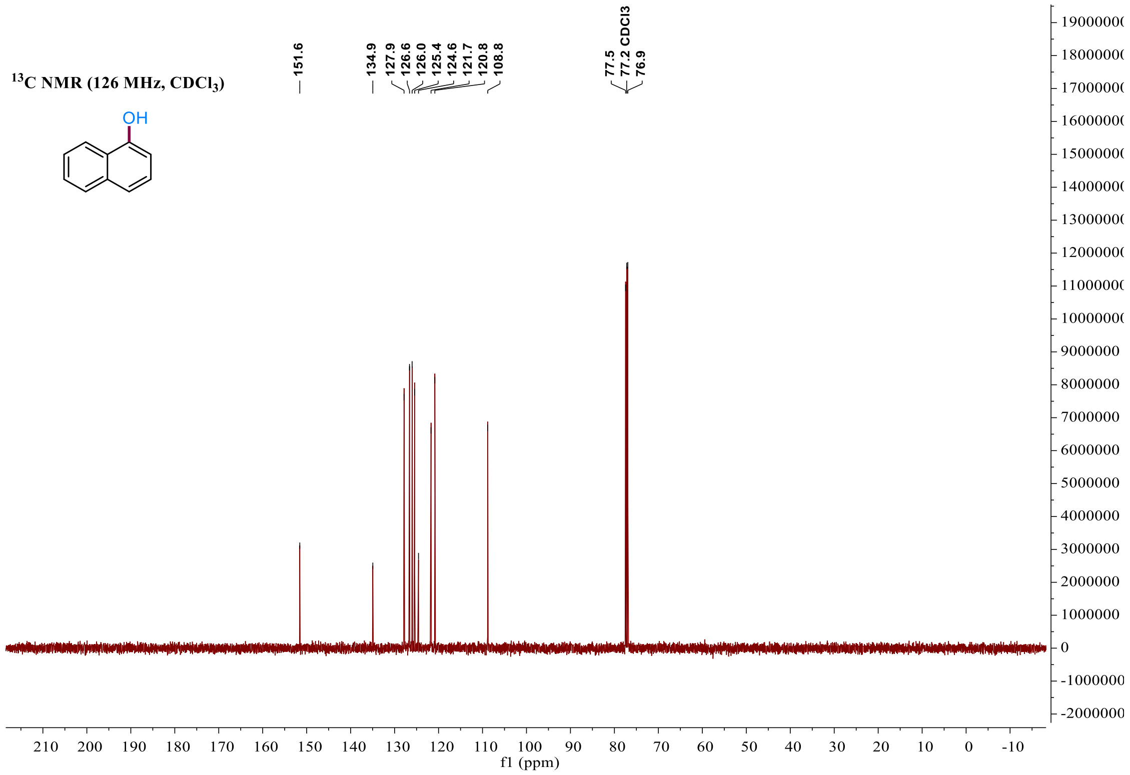

Supplementary Fig. 88. ^{13}C NMR spectra of compound **29** (126 MHz, rt, $CDCl_3$).

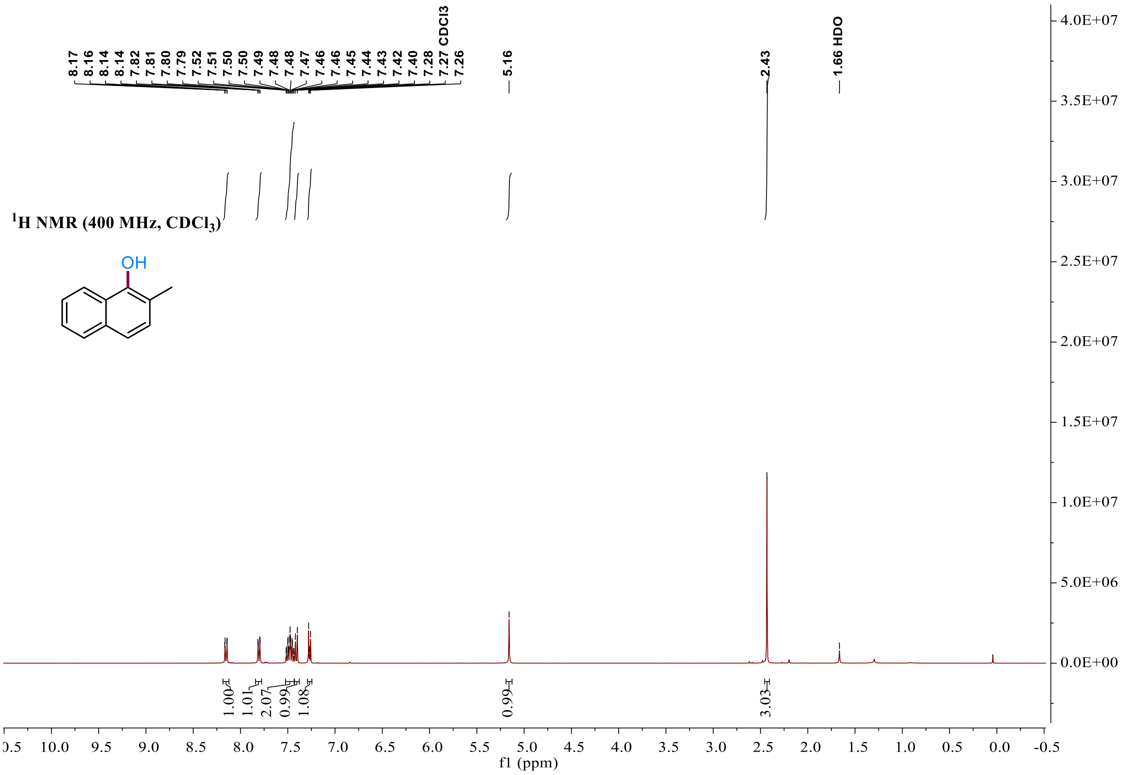

Supplementary Fig. 89. ^{1}H NMR spectra of compound **30** (400 MHz, rt, $CDCl_3$).

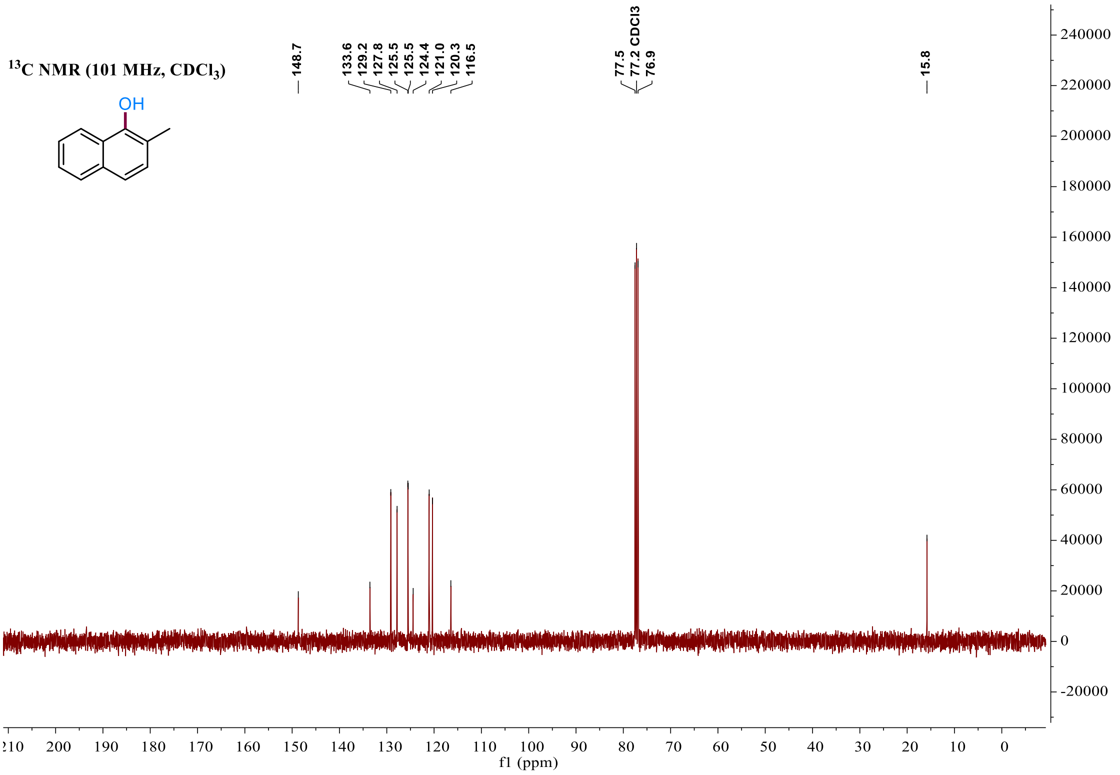

Supplementary Fig. 90. ^{13}C NMR spectra of compound **30** (101 MHz, rt, $CDCl_3$).

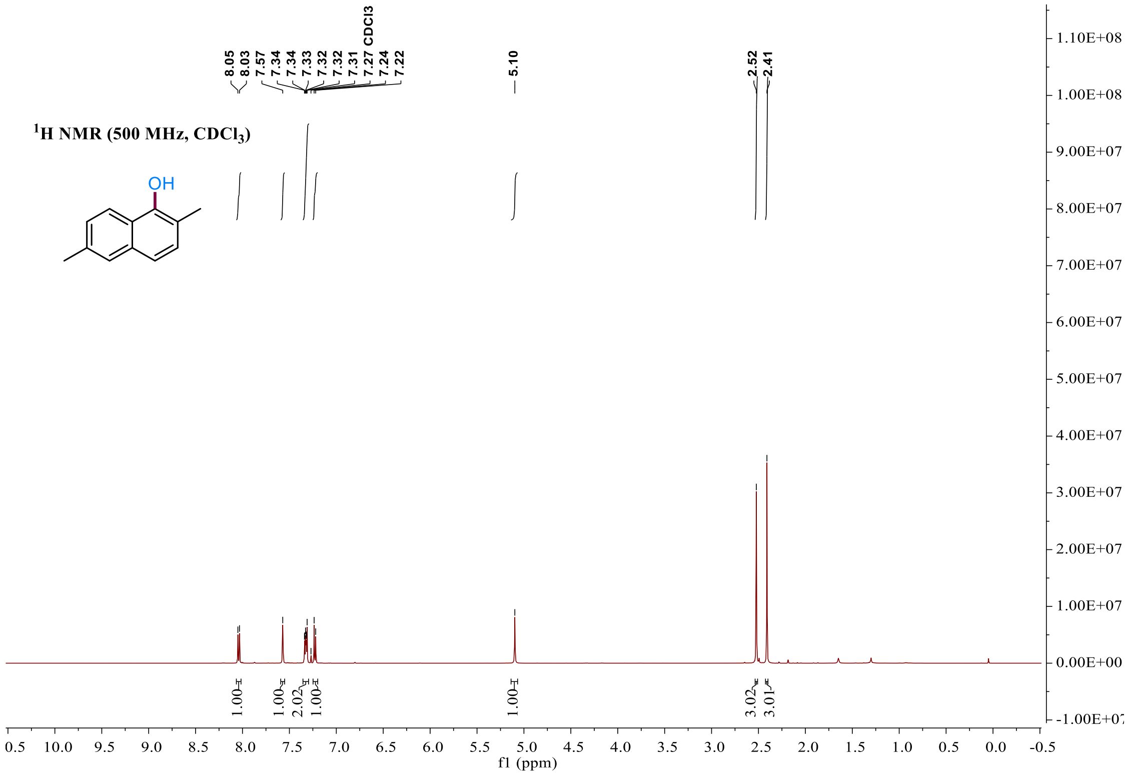

Supplementary Fig. 91. ^{1}H NMR spectra of compound **31** (500 MHz, rt, $CDCl_3$).

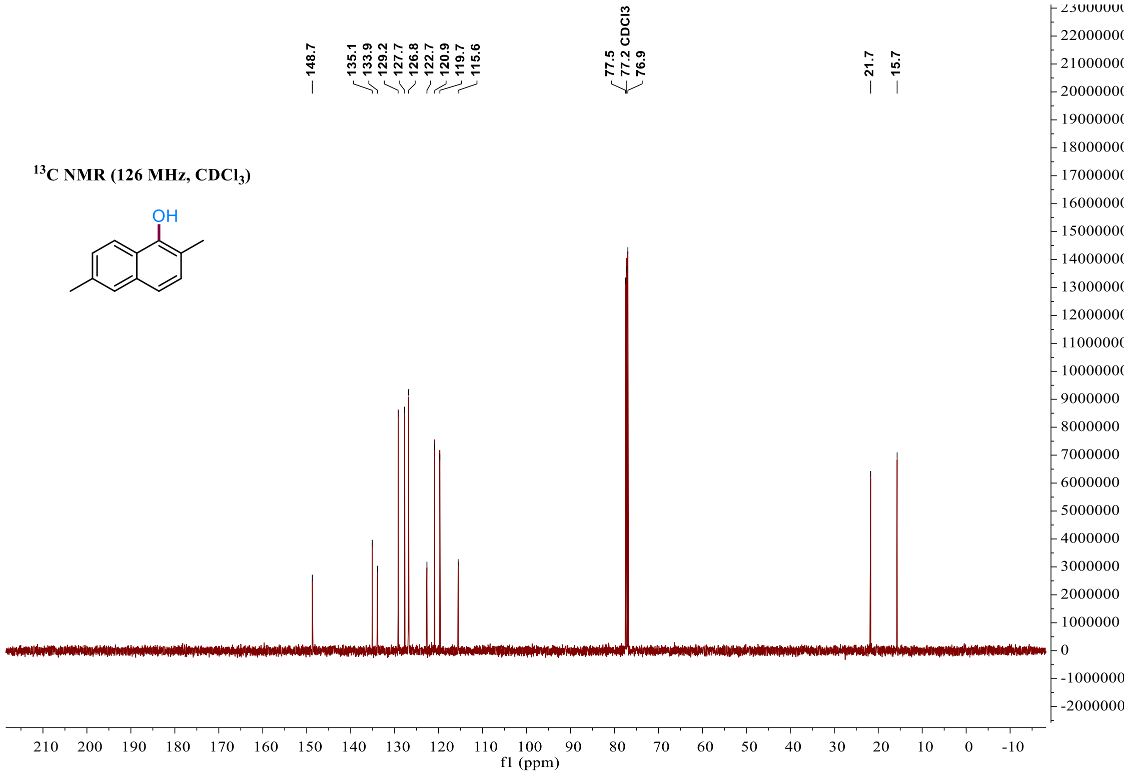

Supplementary Fig. 92. ^{13}C NMR spectra of compound **31** (126 MHz, rt, $CDCl_3$).

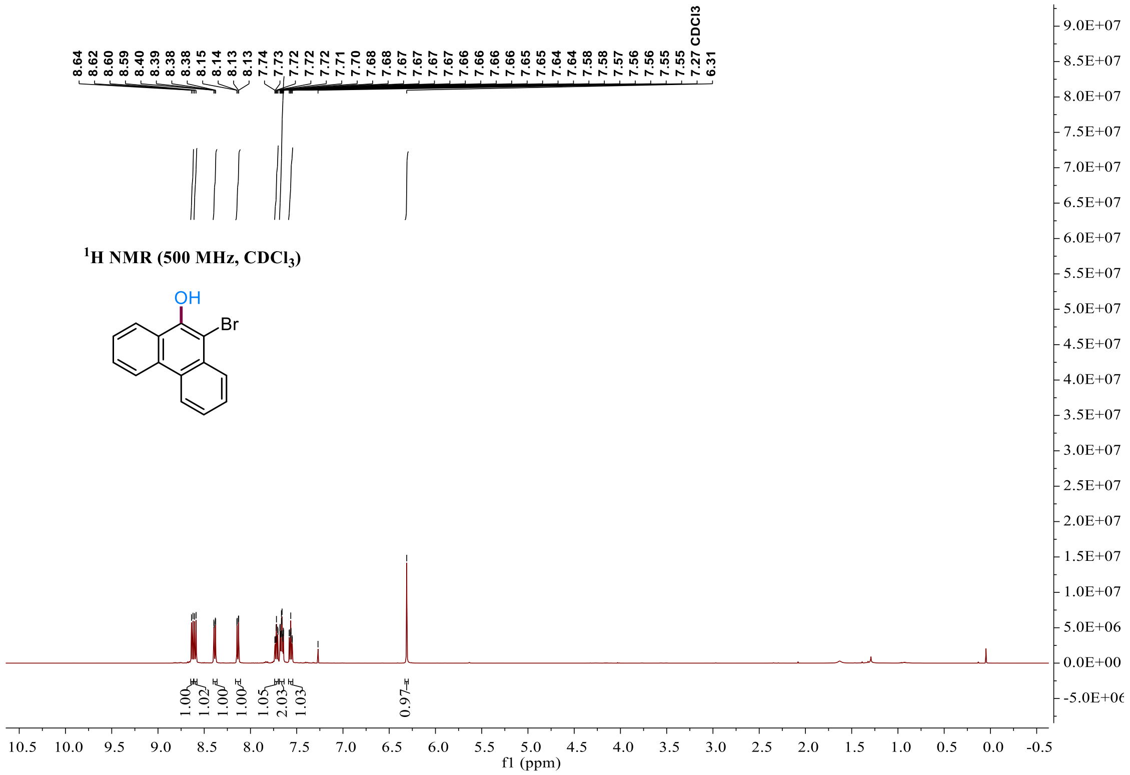

Supplementary Fig. 93. ^{1}H NMR spectra of compound **32** (500 MHz, rt, $CDCl_3$).

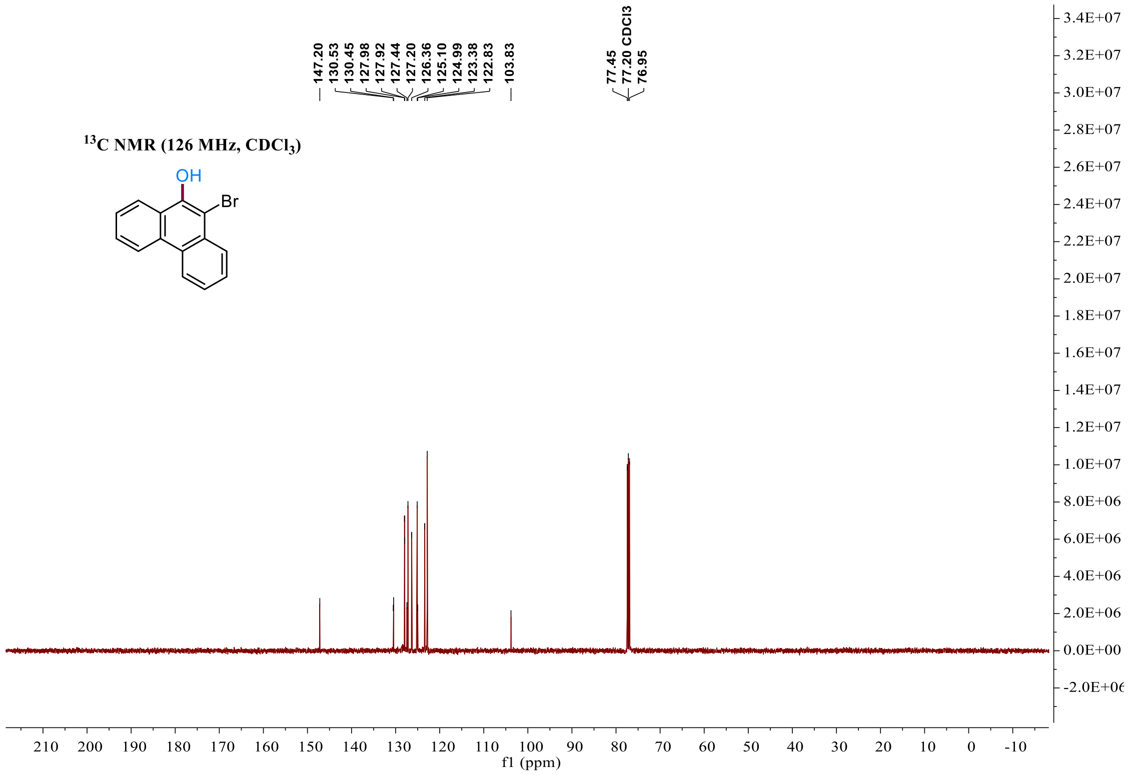

Supplementary Fig. 94. ^{13}C NMR spectra of compound **32** (126 MHz, rt, $CDCl_3$).

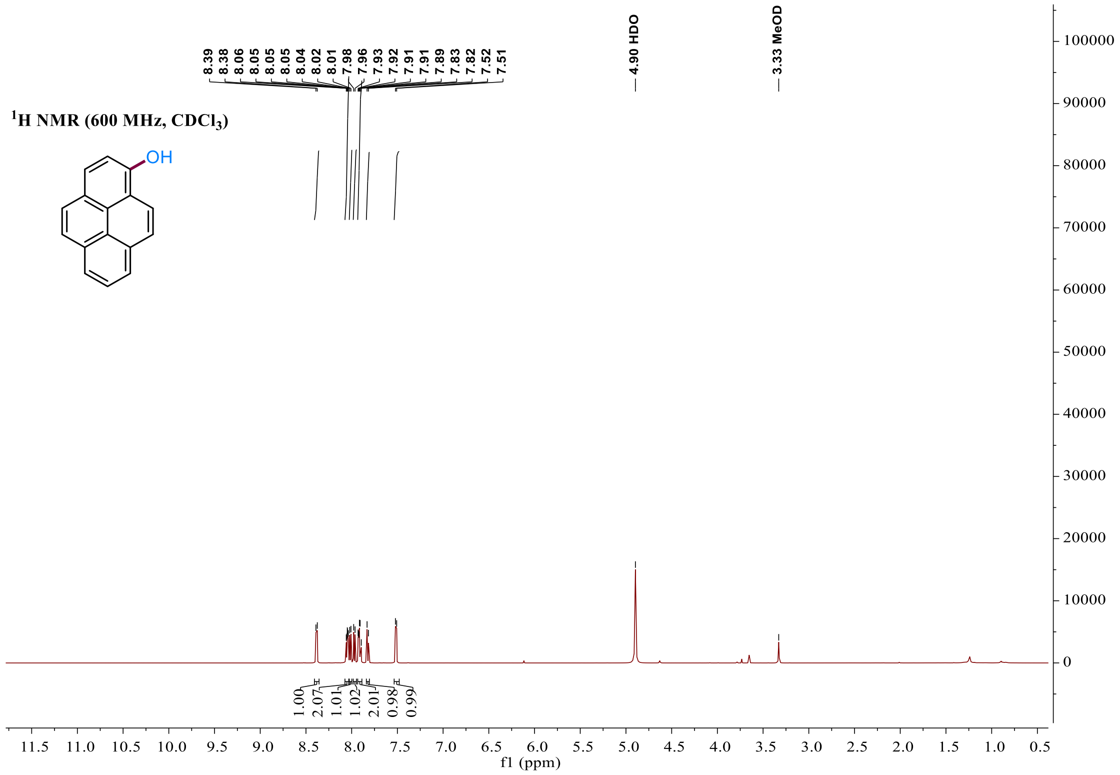

Supplementary Fig. 95. ^{1}H NMR spectra of compound **33** (600 MHz, rt, $CDCl_3$).

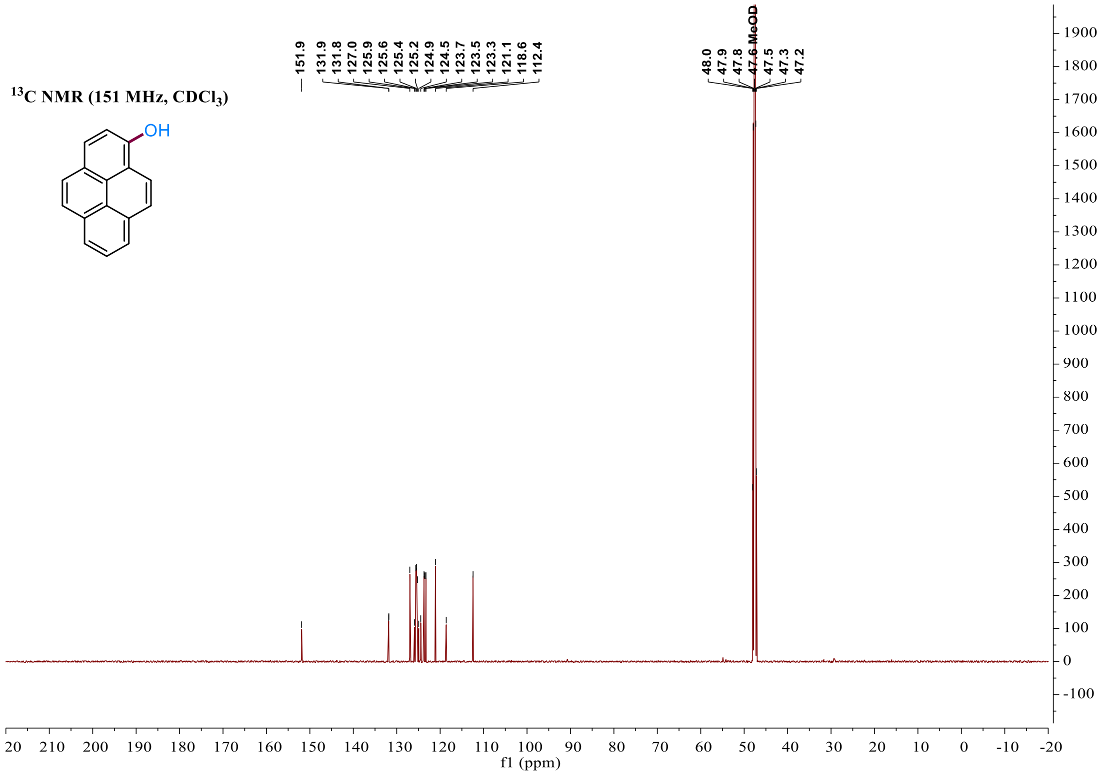

Supplementary Fig. 96. ^{13}C NMR spectra of compound **33** (126 MHz, rt, $CDCl_3$).

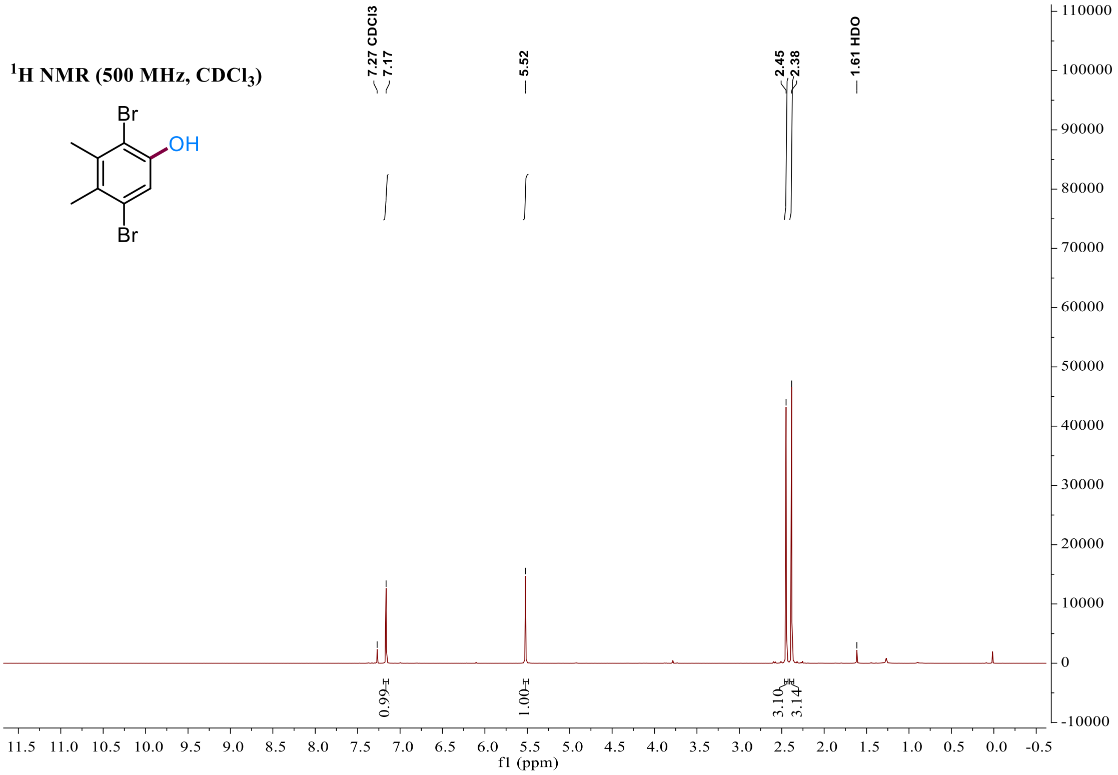

Supplementary Fig. 97. ^{1}H NMR spectra of compound **34** (500 MHz, rt, $CDCl_3$).

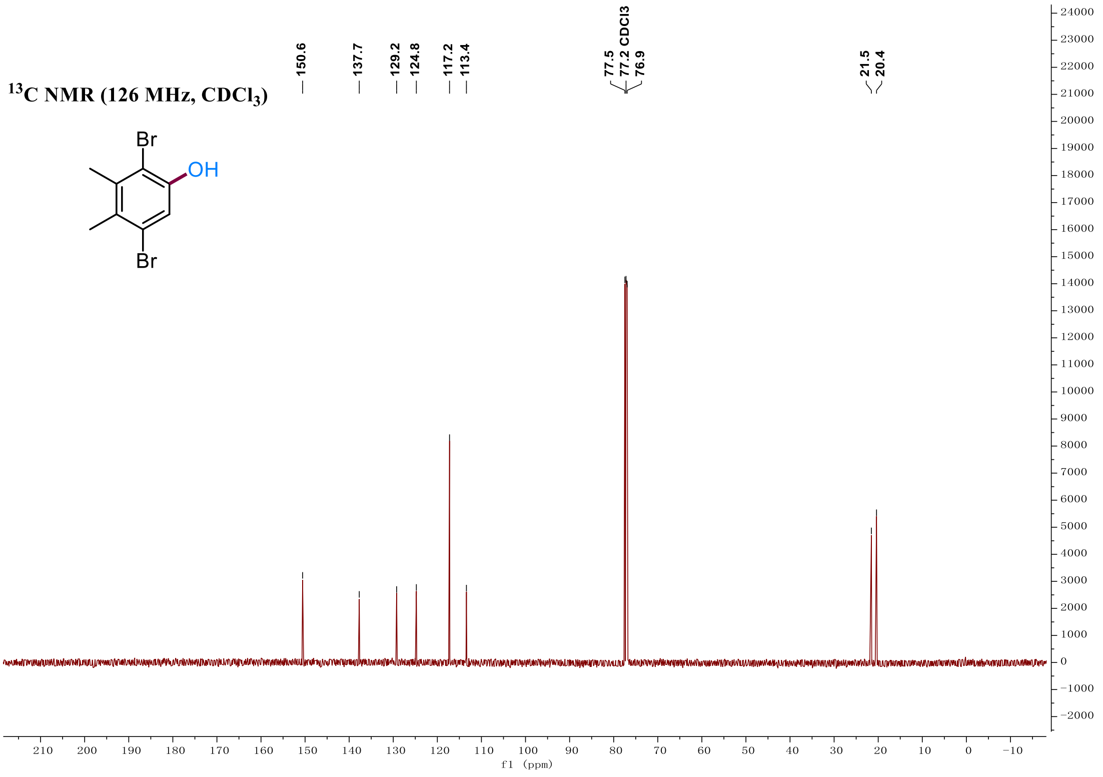

Supplementary Fig. 98. ^{13}C NMR spectra of compound **34** (126 MHz, rt, $CDCl_3$).

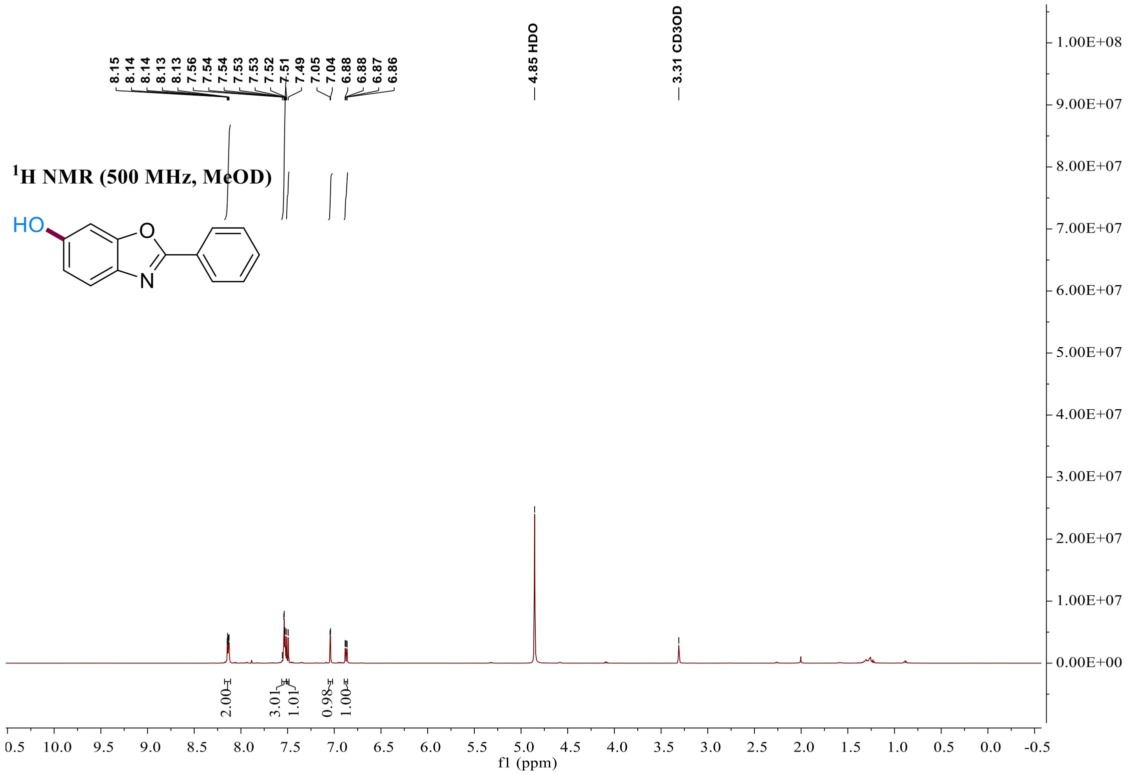

Supplementary Fig. 99. ^{1}H NMR spectra of compound **36** (500 MHz, rt, MeOD).

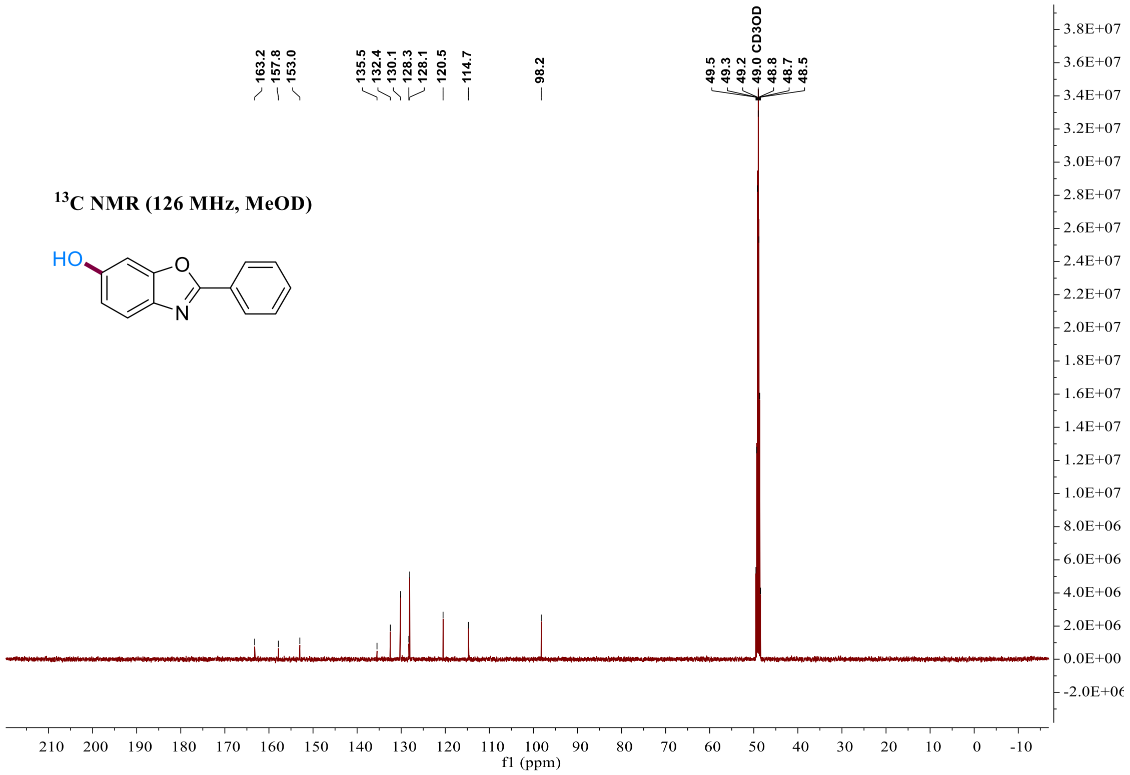

Supplementary Fig. 100. ^{13}C NMR spectra of compound **36** (126 MHz, rt, MeOD).

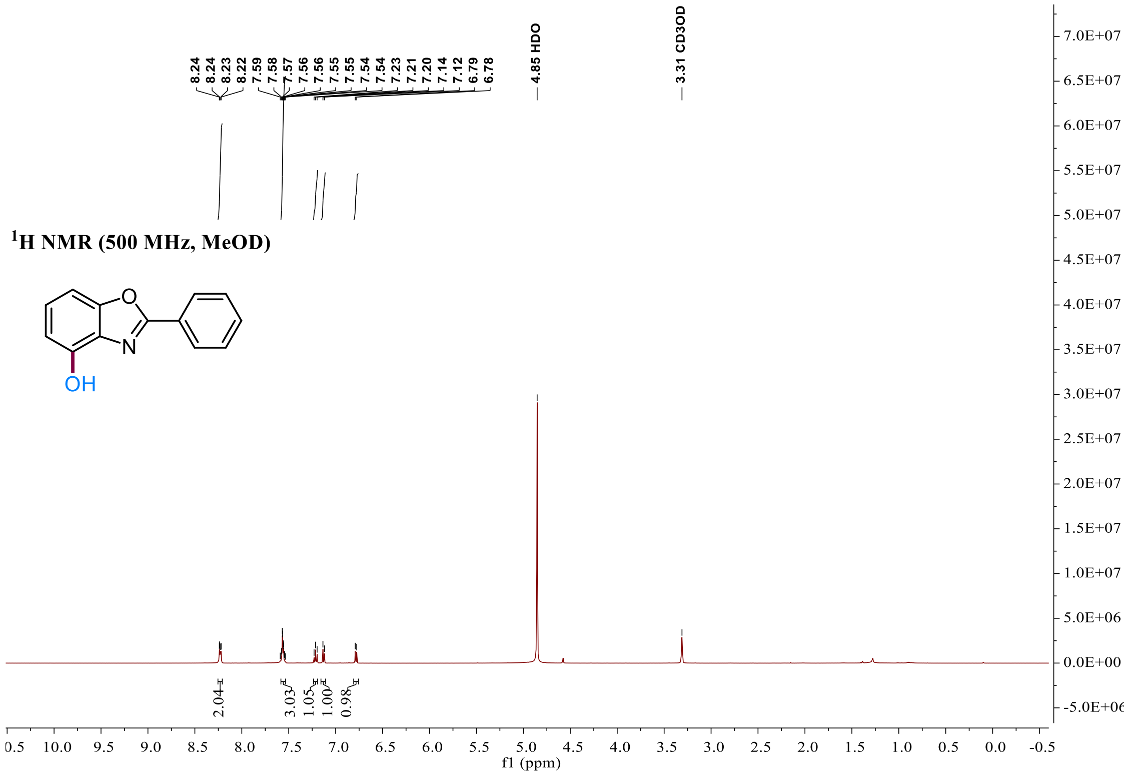

Supplementary Fig. 101. ^{1}H NMR spectra of compound **36** (500 MHz, rt, MeOD).

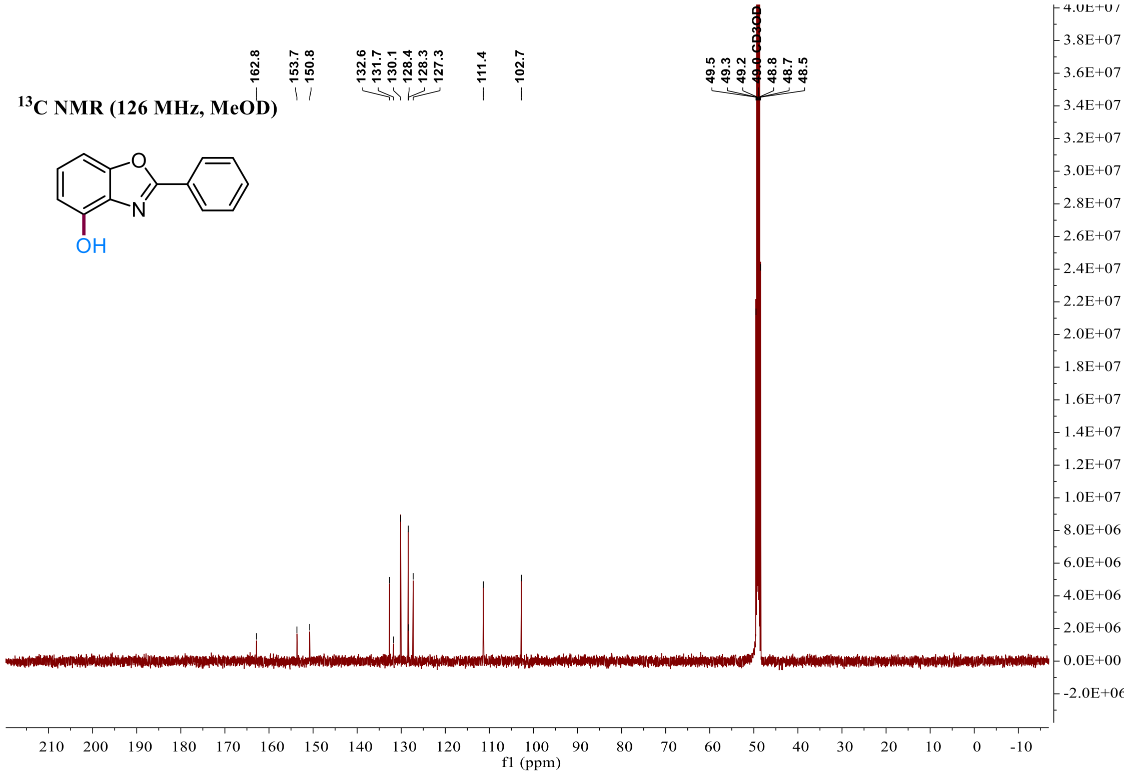

Supplementary Fig. 102. ^{13}C NMR spectra of compound **36** (126 MHz, rt, MeOD).

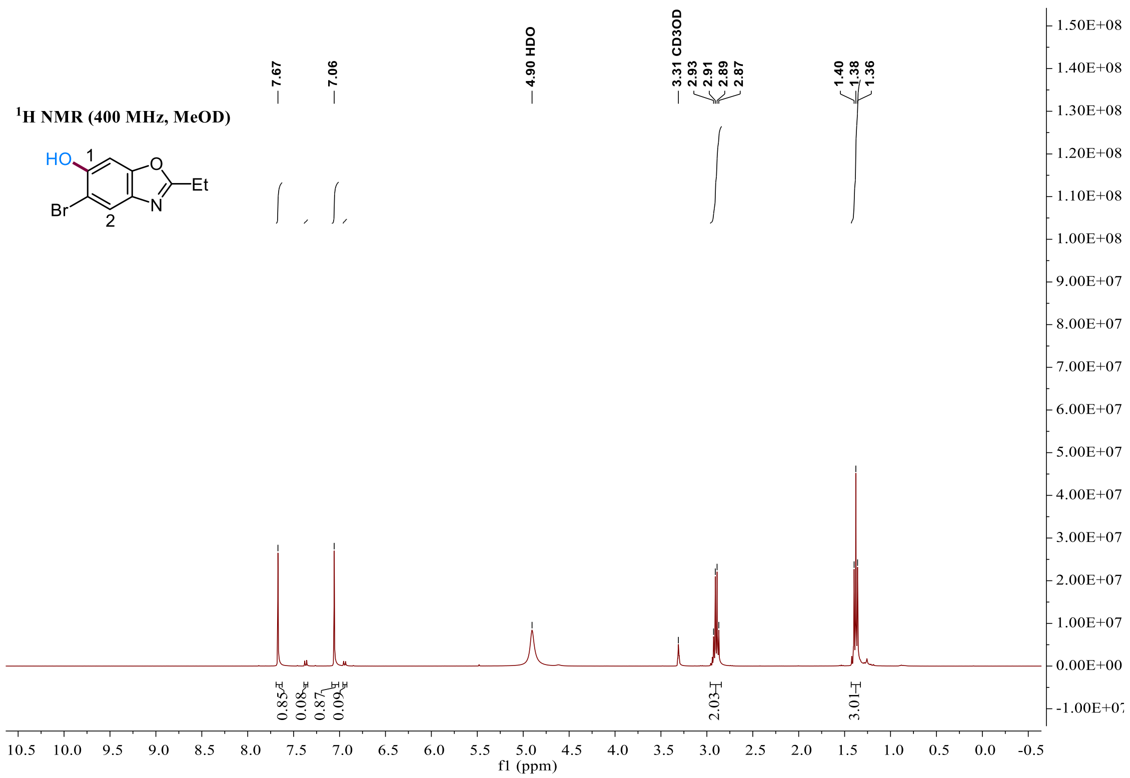

Supplementary Fig. 103. ^{1}H NMR spectra of compound **37** (400 MHz, rt, MeOD).

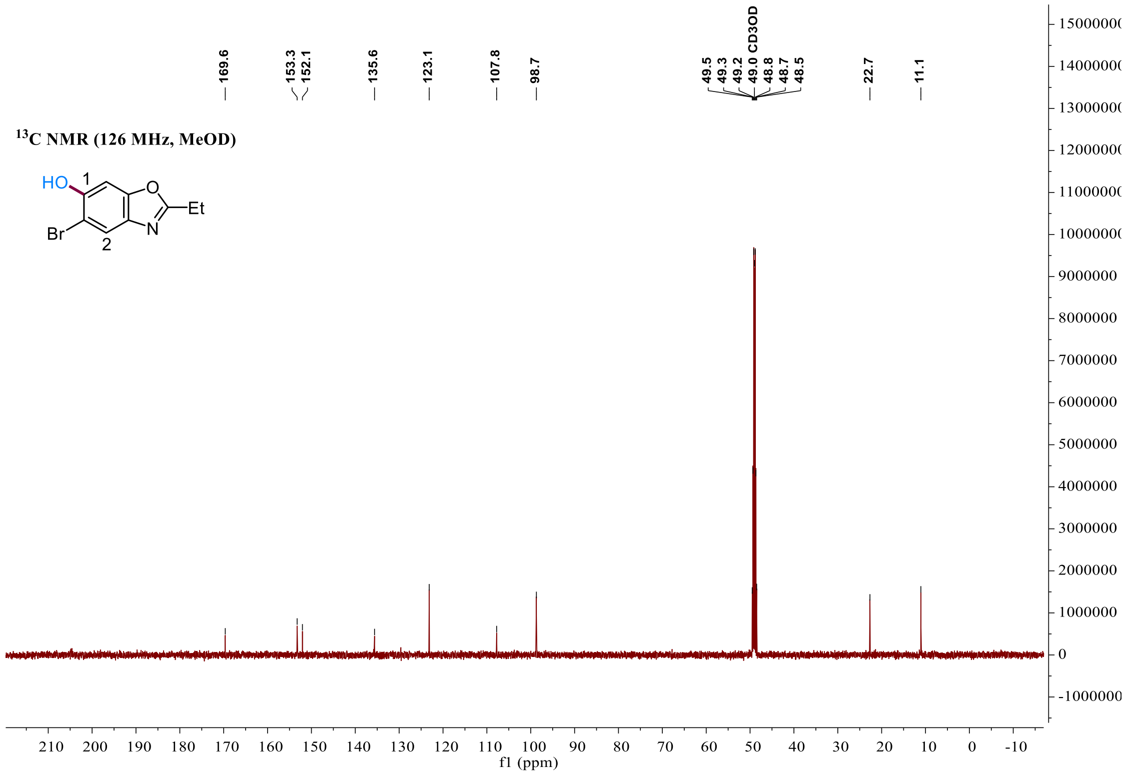

Supplementary Fig. 104. ^{13}C NMR spectra of compound **37** (126 MHz, rt, MeOD).

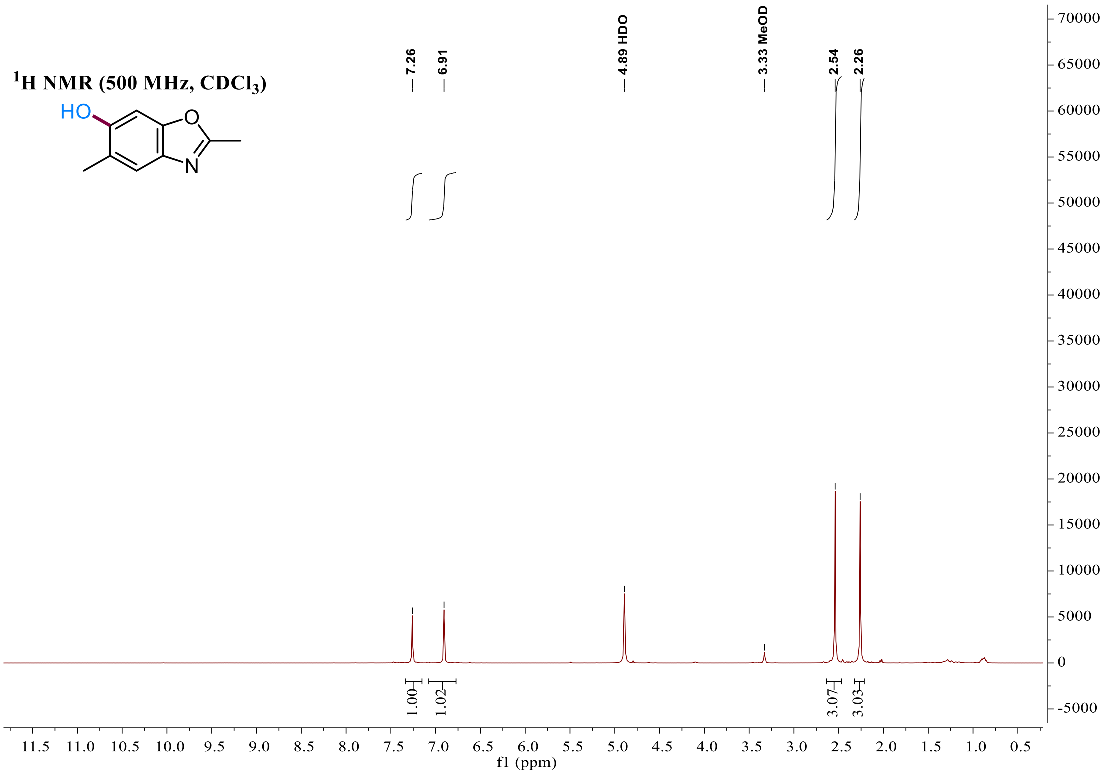

Supplementary Fig. 105. ^{1}H NMR spectra of compound **38** (500 MHz, rt, $CDCl_3$).

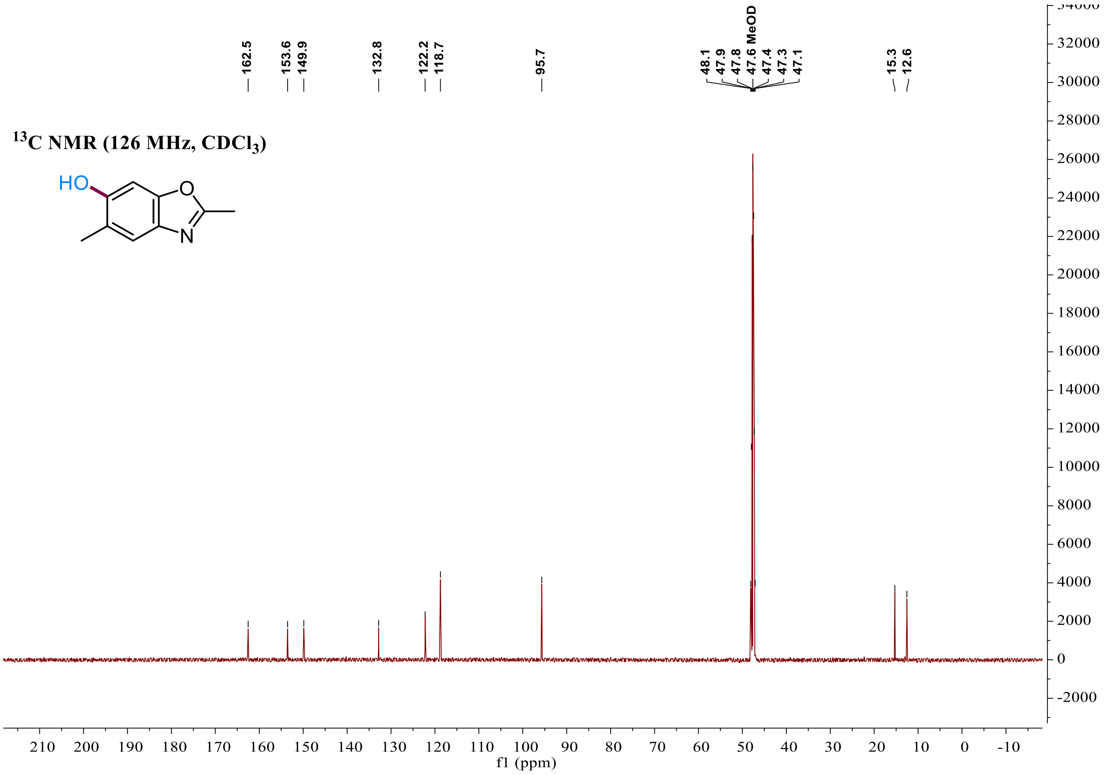

Supplementary Fig. 106. ^{13}C NMR spectra of compound **38** (126 MHz, rt, $CDCl_3$).

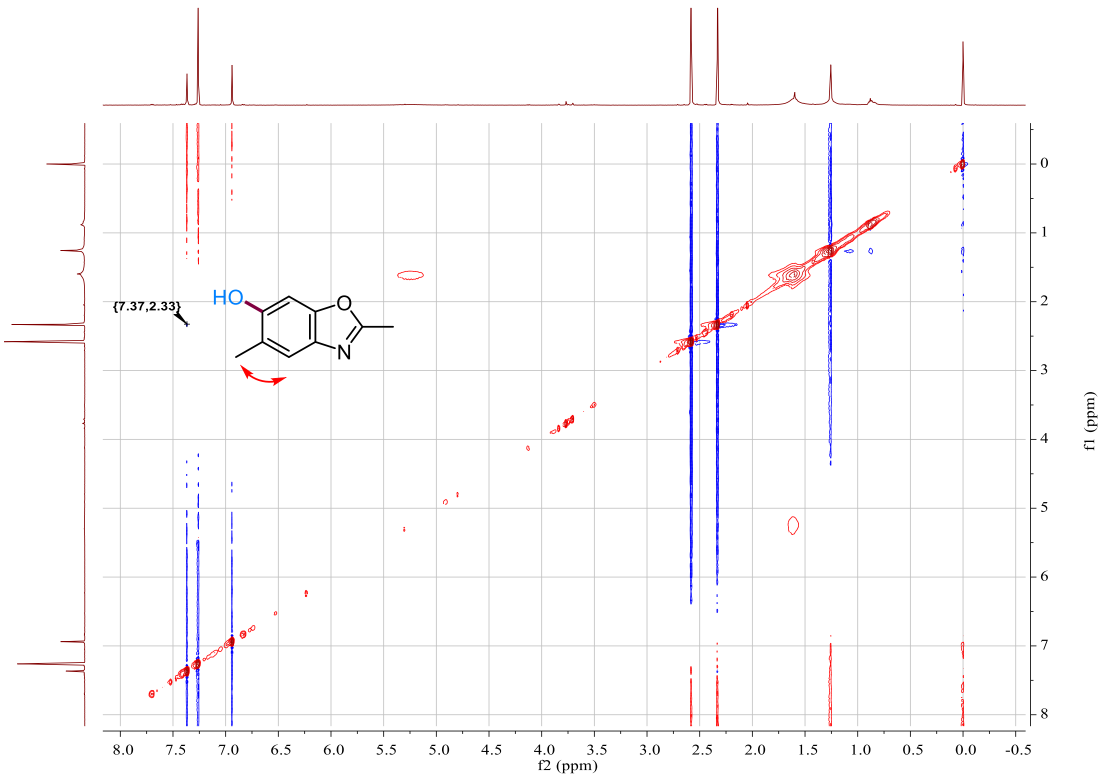

Supplementary Fig. 107. 2D NOESY spectra of compound **38** (500 MHz, rt, $CDCl_3$).

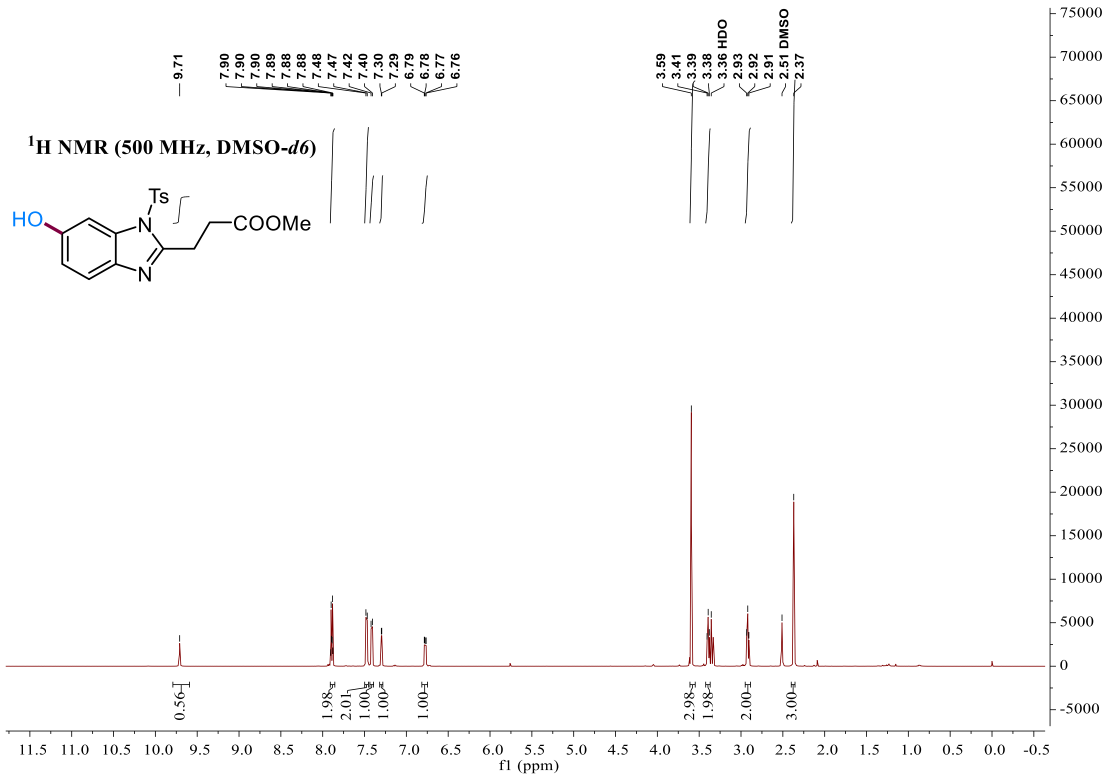

Supplementary Fig. 108. ^{1}H NMR spectra of compound **39** (500 MHz, rt, DMSO).

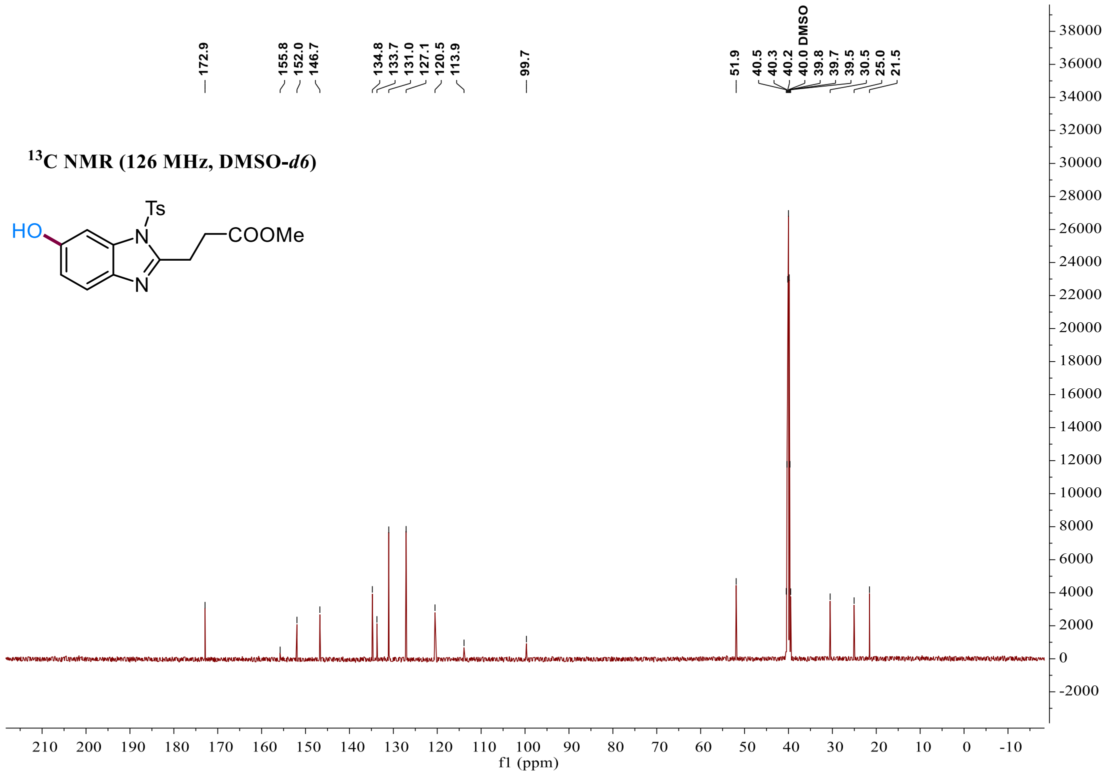

Supplementary Fig. 109. ^{13}C NMR spectra of compound **39** (126 MHz, rt, DMSO).

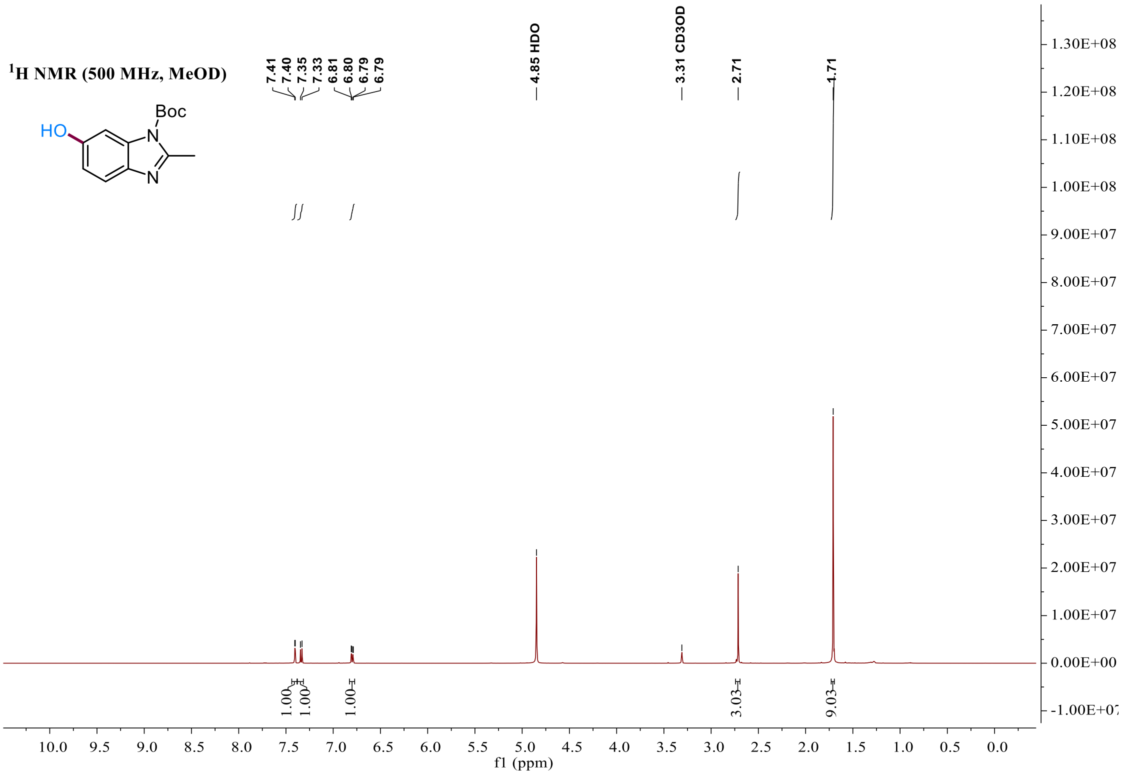

Supplementary Fig. 110. ^{1}H NMR spectra of compound **40** (500 MHz, rt, MeOD).

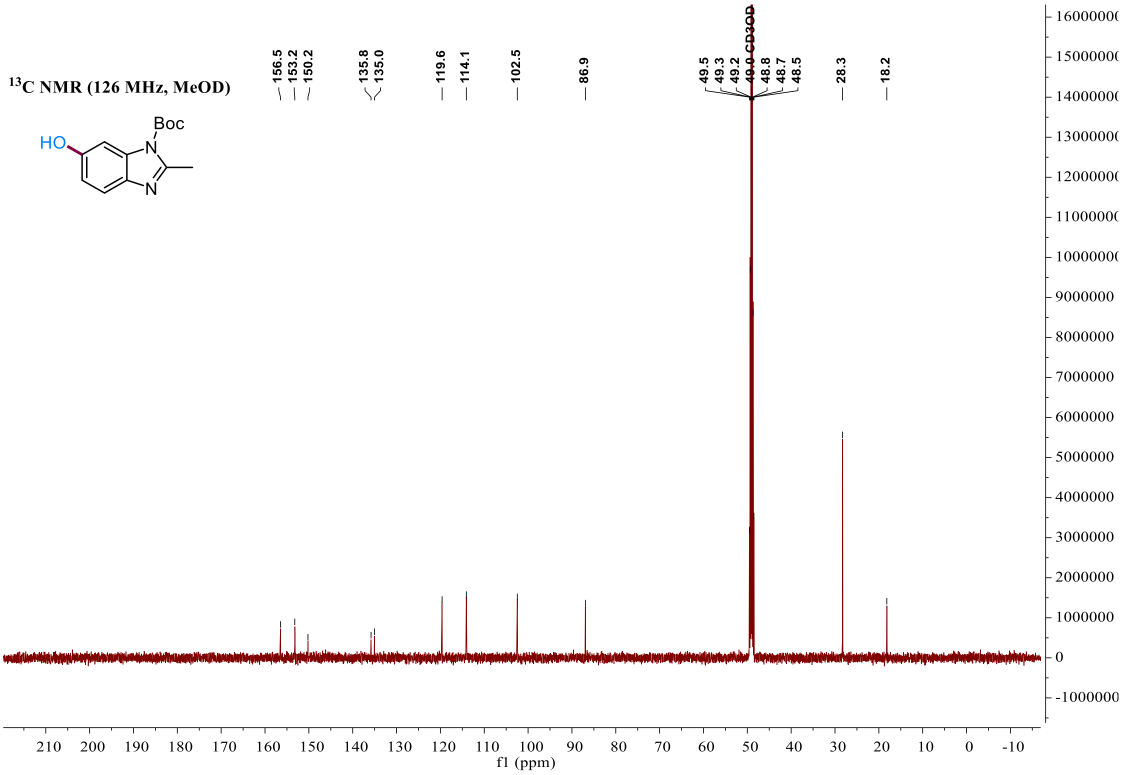

Supplementary Fig. 111. ^{13}C NMR spectra of compound **40** (126 MHz, rt, MeOD).

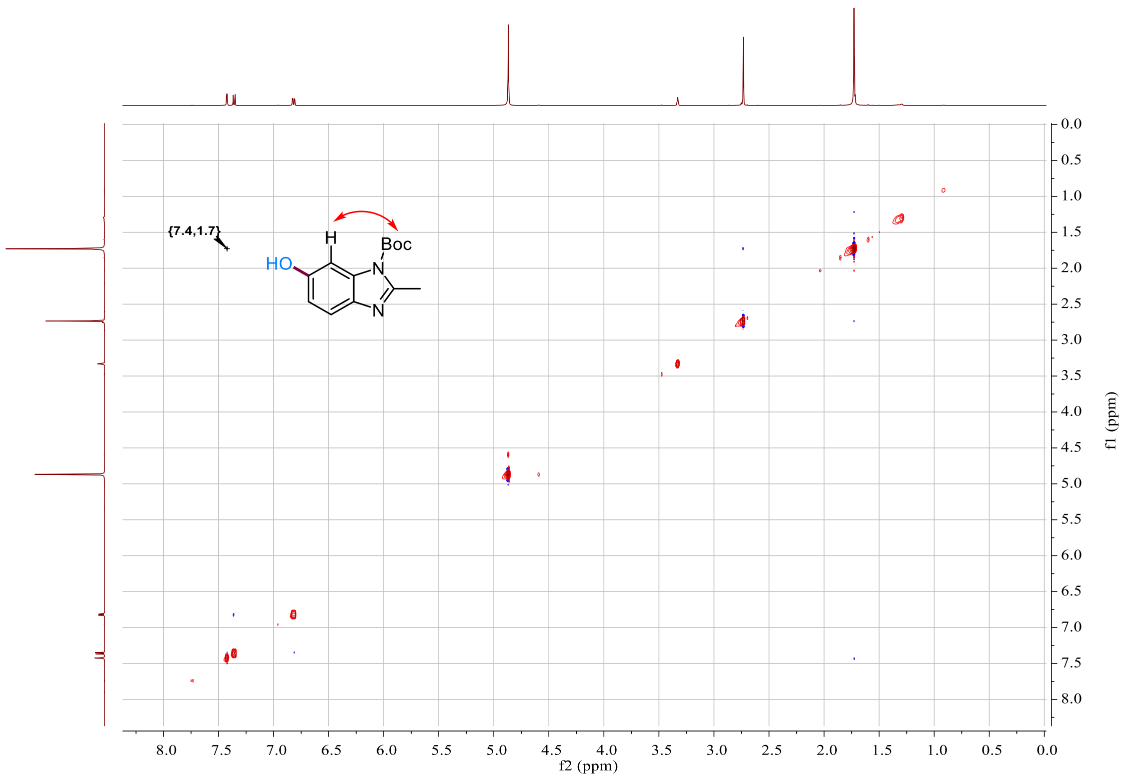

Supplementary Fig. 112. 2D NOESY spectra of compound **40** (500 MHz, rt, MeOD).

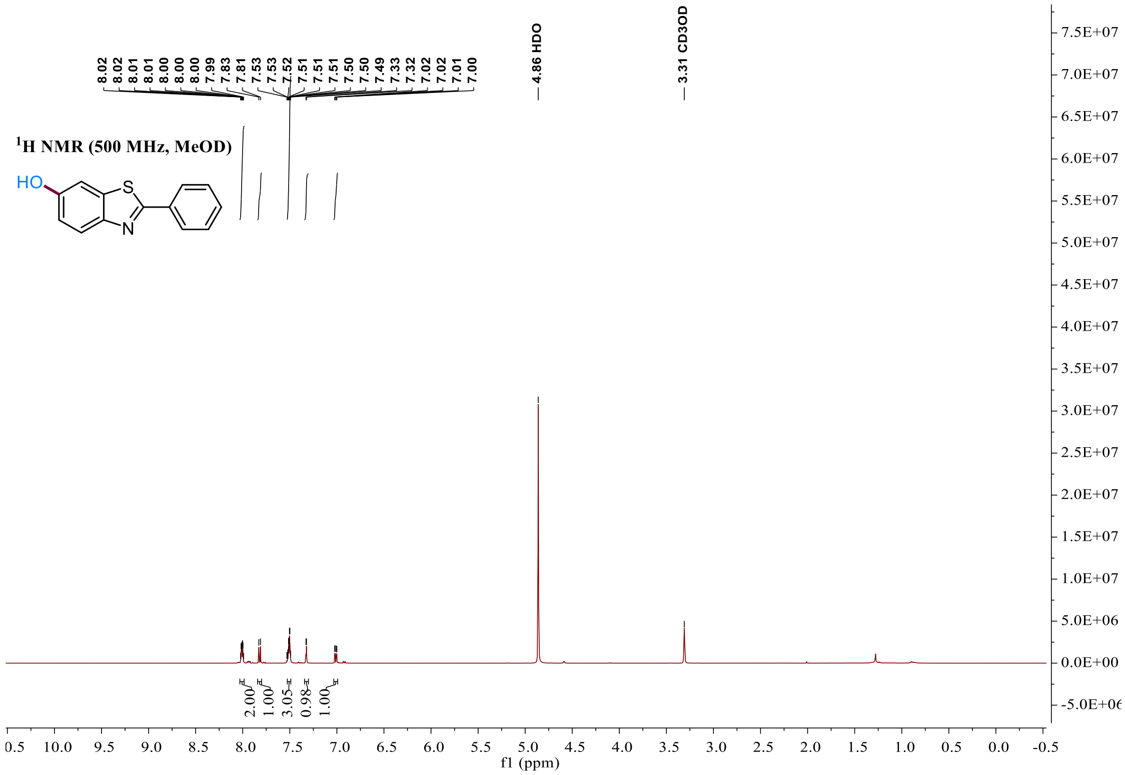

Supplementary Fig. 113. ^{1}H NMR spectra of compound **41** (500 MHz, rt, MeOD).

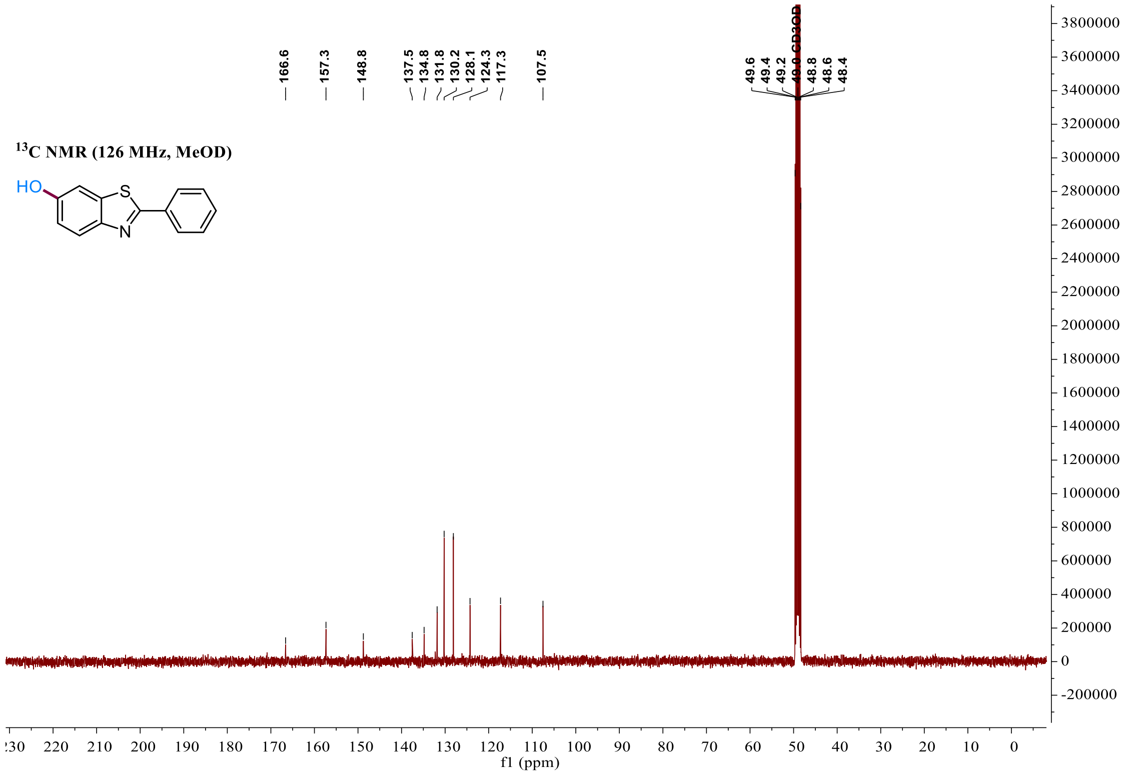

Supplementary Fig. 114. ^{13}C NMR spectra of compound **41** (126 MHz, rt, MeOD).

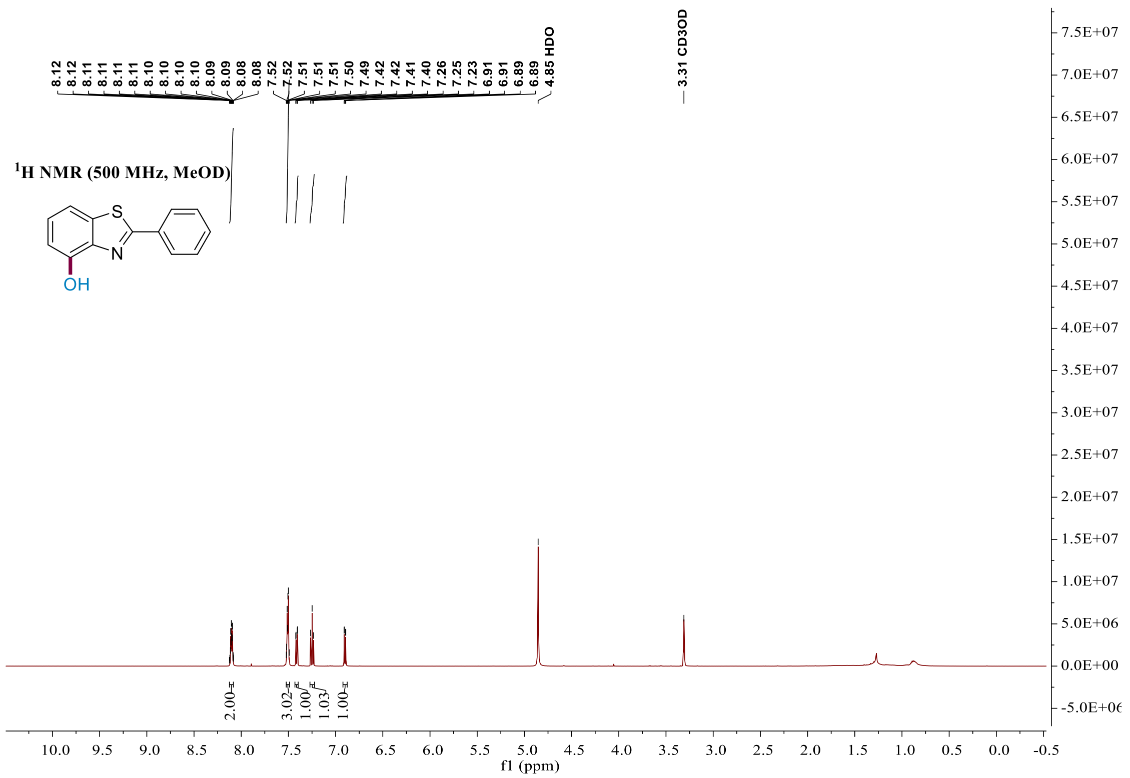

Supplementary Fig. 115. ^{1}H NMR spectra of compound **41** (500 MHz, rt, MeOD).

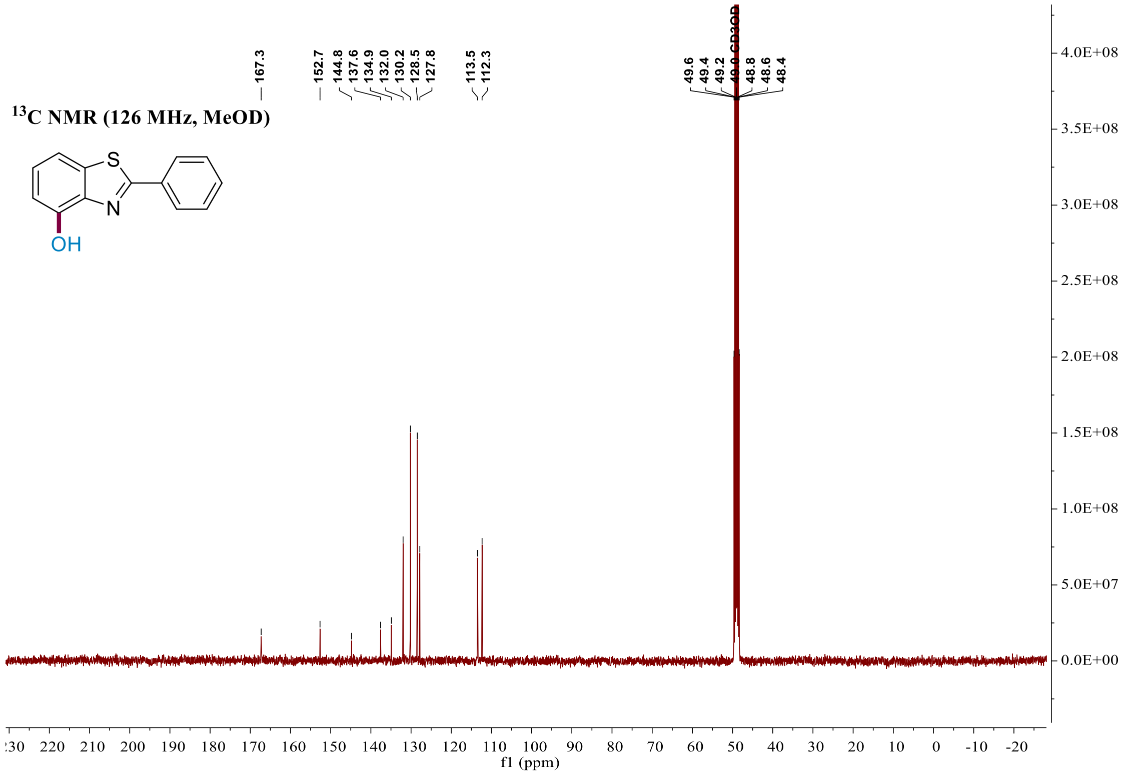

Supplementary Fig. 116. ^{13}C NMR spectra of compound **41** (126 MHz, rt, MeOD).

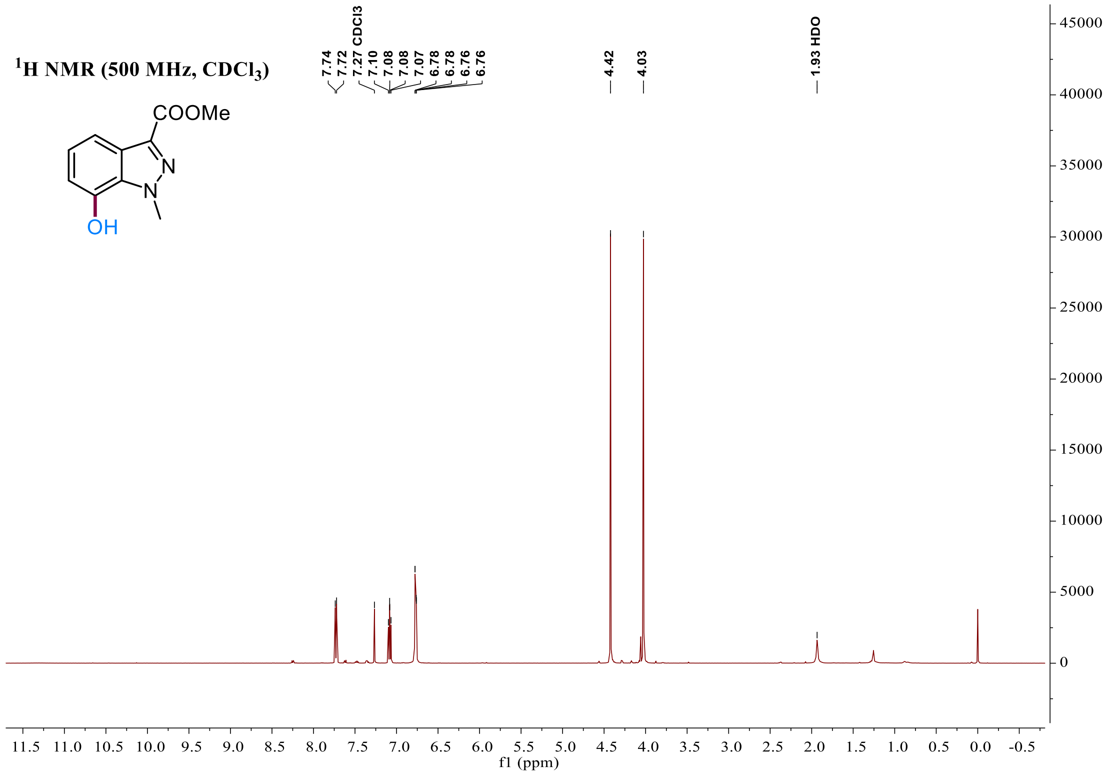

Supplementary Fig. 117. ^{1}H NMR spectra of compound **42** (500 MHz, rt, $CDCl_3$).

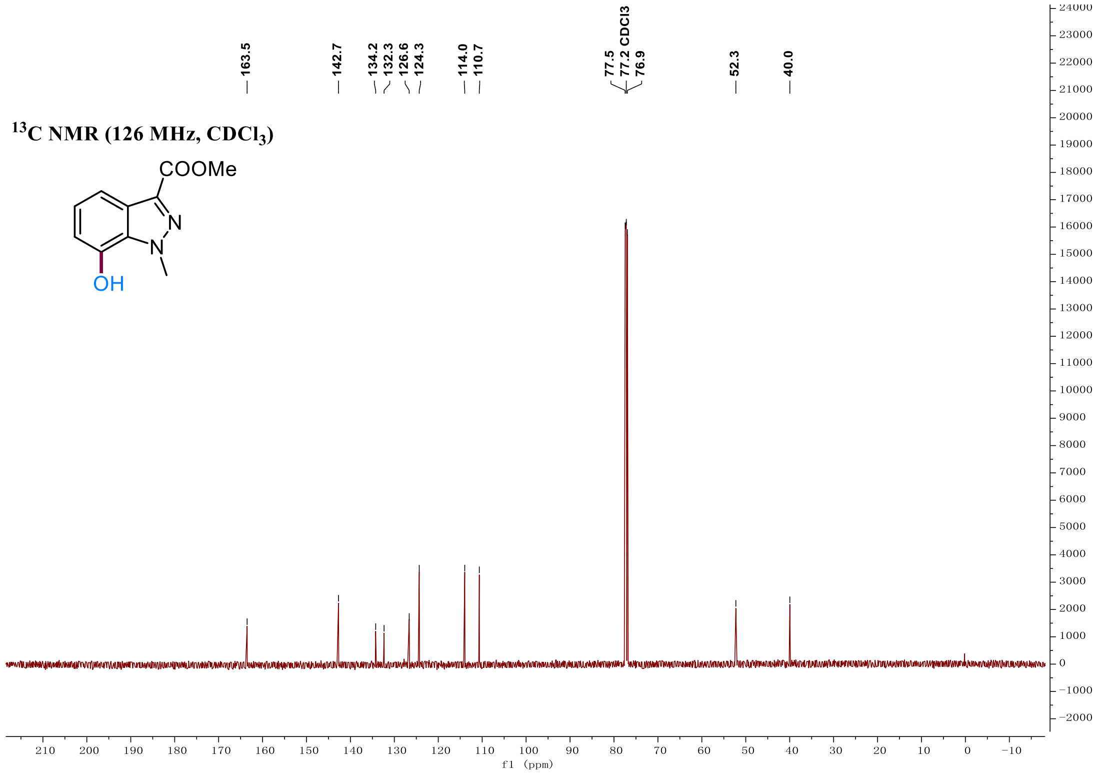

Supplementary Fig. 118. ^{13}C NMR spectra of compound **42** (126 MHz, rt, $CDCl_3$).

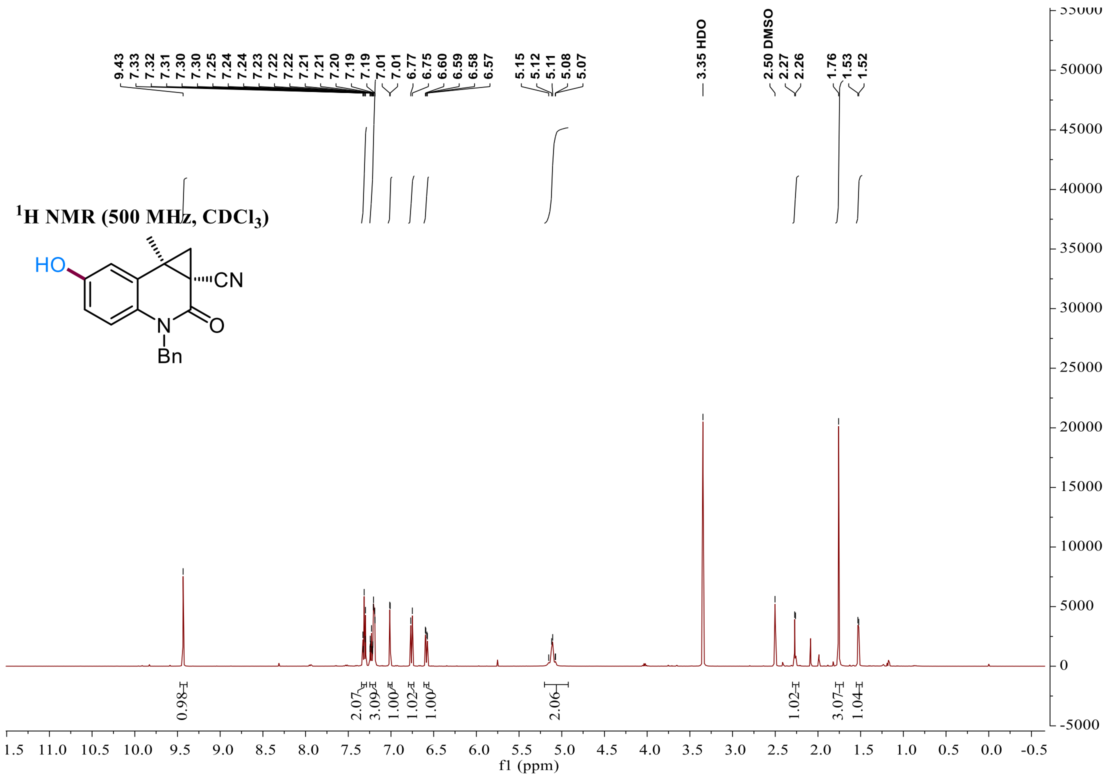

Supplementary Fig. 119. ^{1}H NMR spectra of compound **43** (500 MHz, rt, $CDCl_3$).

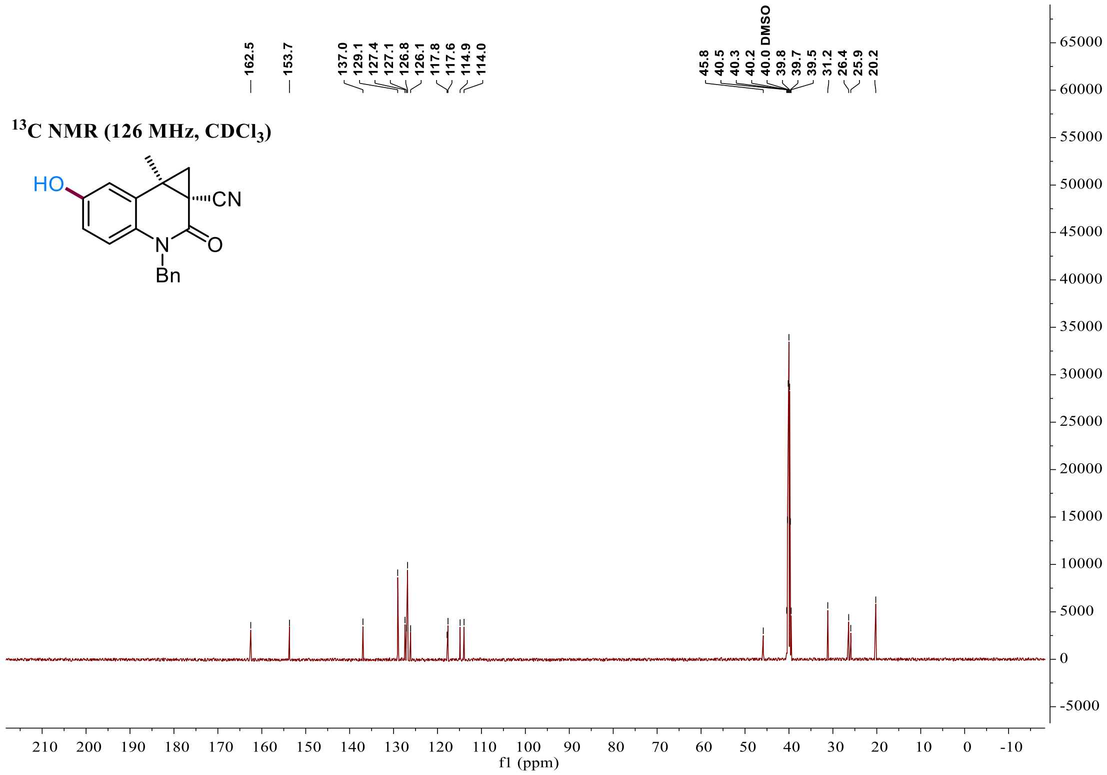

Supplementary Fig. 120. ^{13}C NMR spectra of compound **43** (126 MHz, rt, $CDCl_3$).

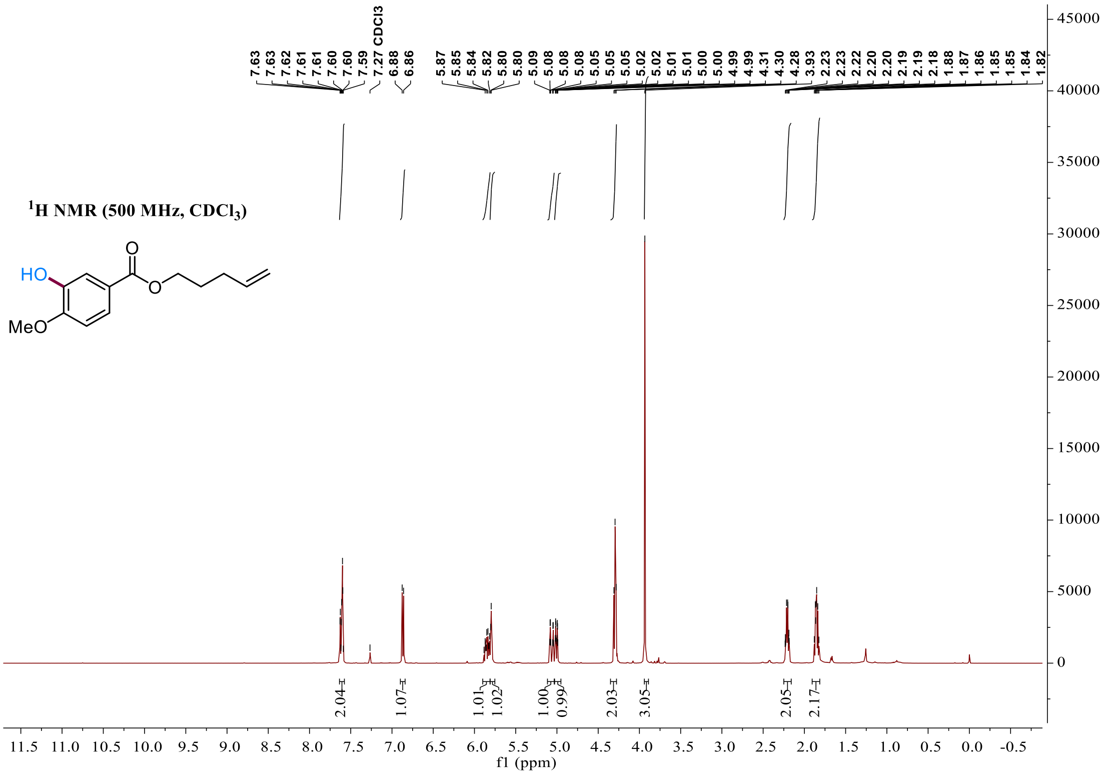

Supplementary Fig. 121. ^{1}H NMR spectra of compound **44** (500 MHz, rt, $CDCl_3$).

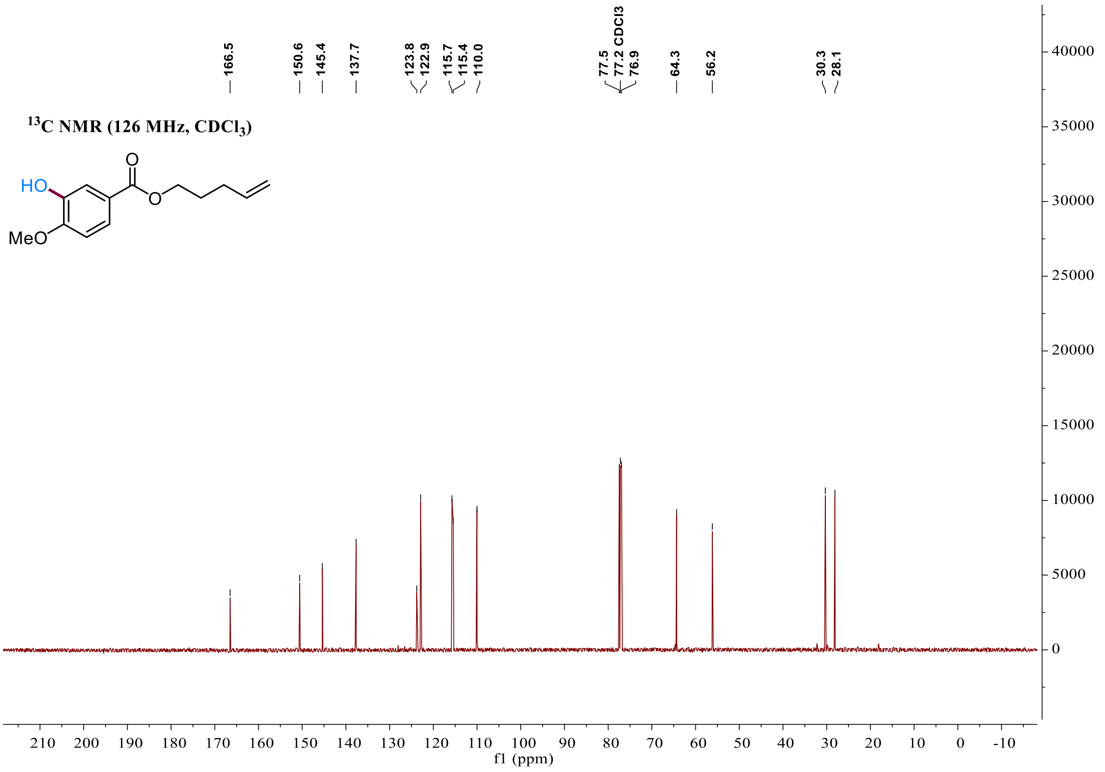

Supplementary Fig. 122. ^{13}C NMR spectra of compound **44** (126 MHz, rt, $CDCl_3$).

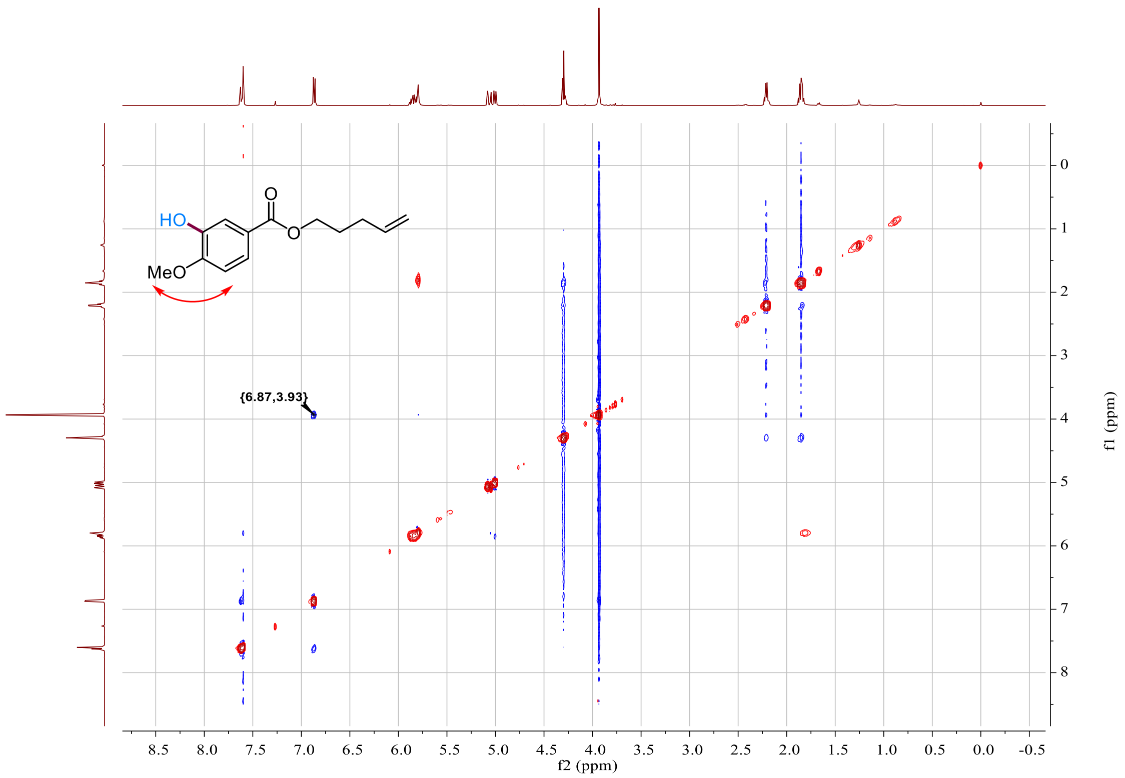

Supplementary Fig. 123. 2D NOESY spectra of compound **44** (500 MHz, rt, $CDCl_3$).

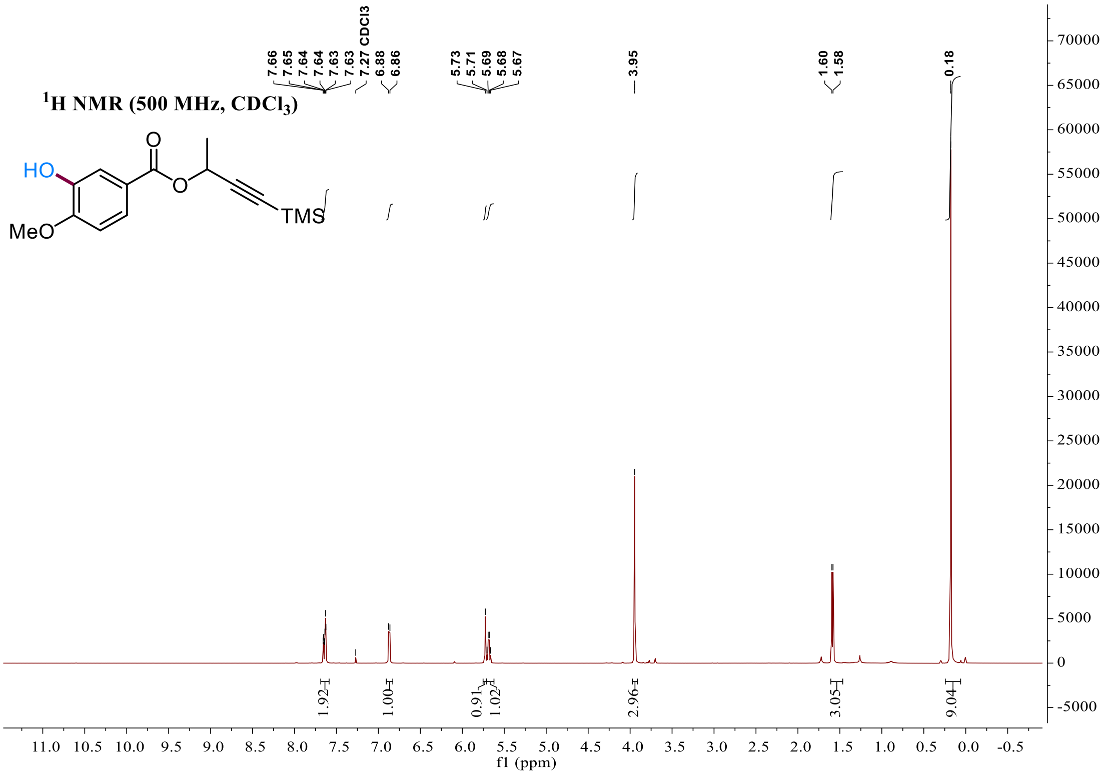

Supplementary Fig. 124. ^{1}H NMR spectra of compound **45** (500 MHz, rt, $CDCl_3$).

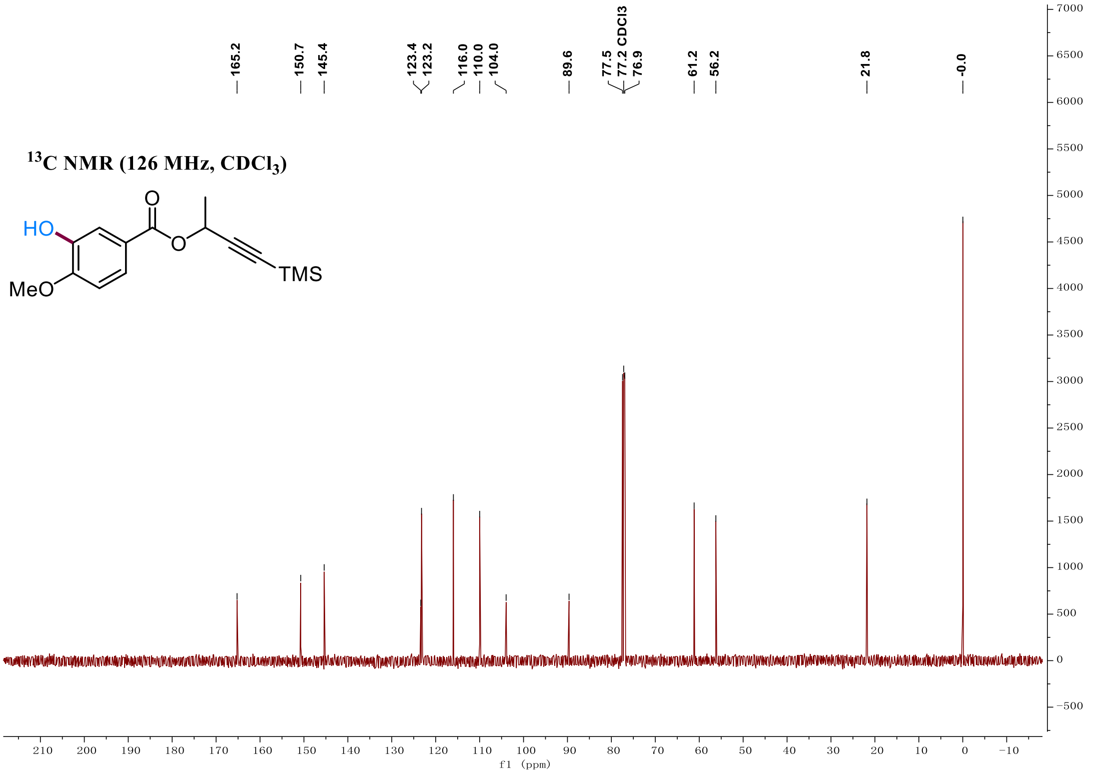

Supplementary Fig. 125. ^{13}C NMR spectra of compound **45** (126 MHz, rt, $CDCl_3$).

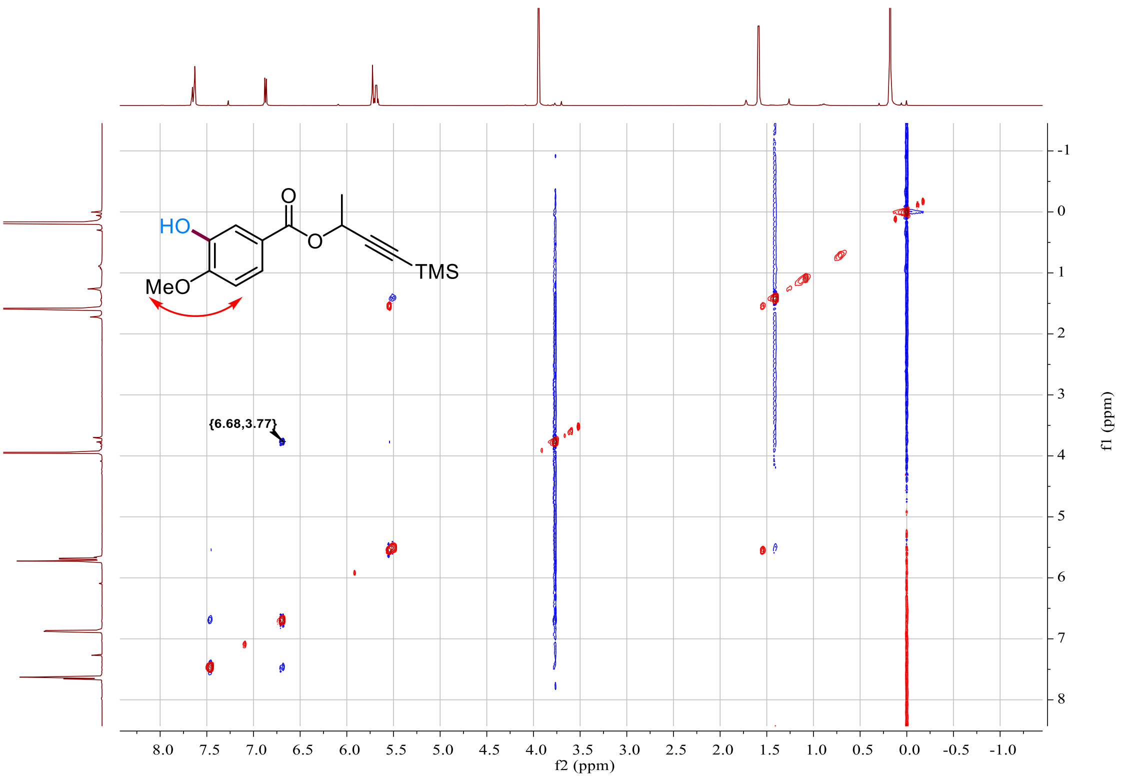

Supplementary Fig. 126. 2D NOESY spectra of compound **45** (500 MHz, rt, $CDCl_3$).

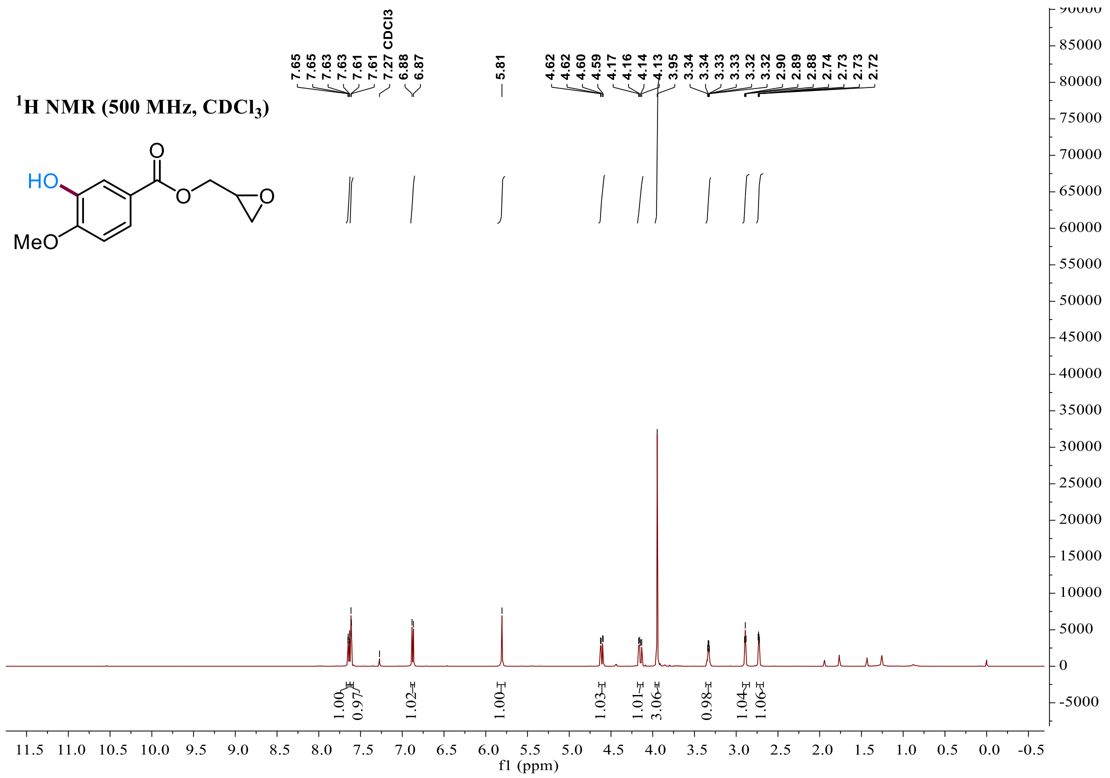

Supplementary Fig. 127. ^{1}H NMR spectra of compound **46** (500 MHz, rt, $CDCl_3$).

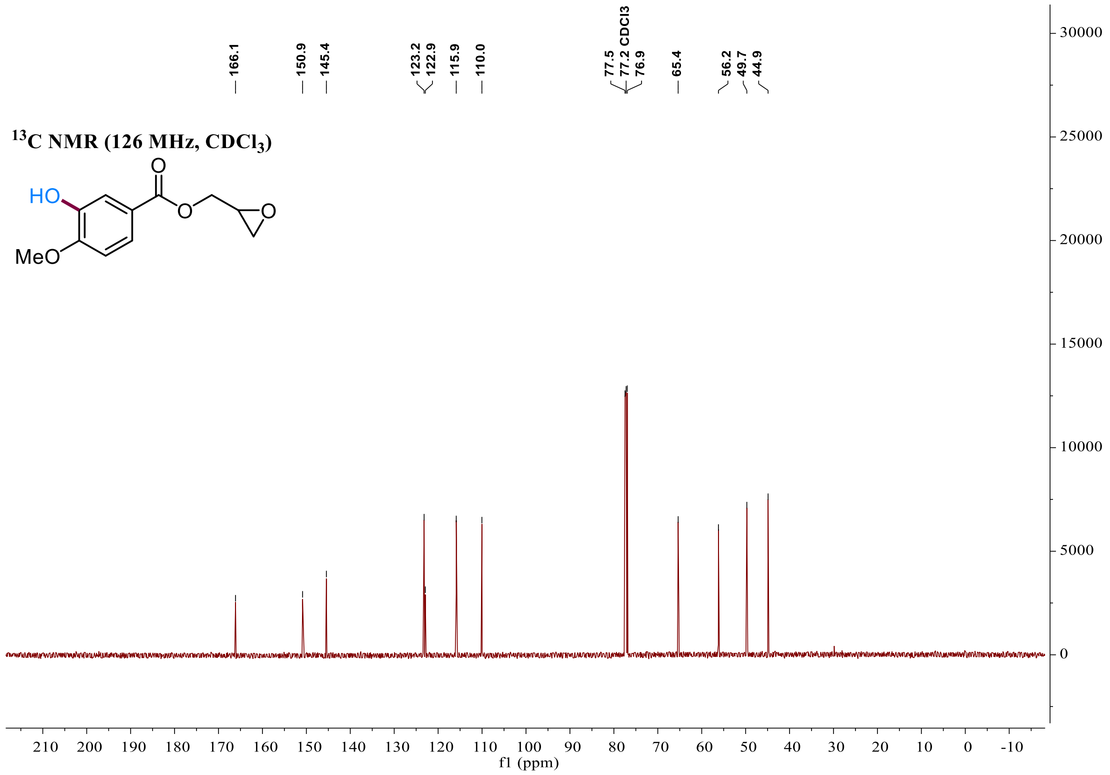

Supplementary Fig. 128. ^{13}C NMR spectra of compound **46** (126 MHz, rt, $CDCl_3$).

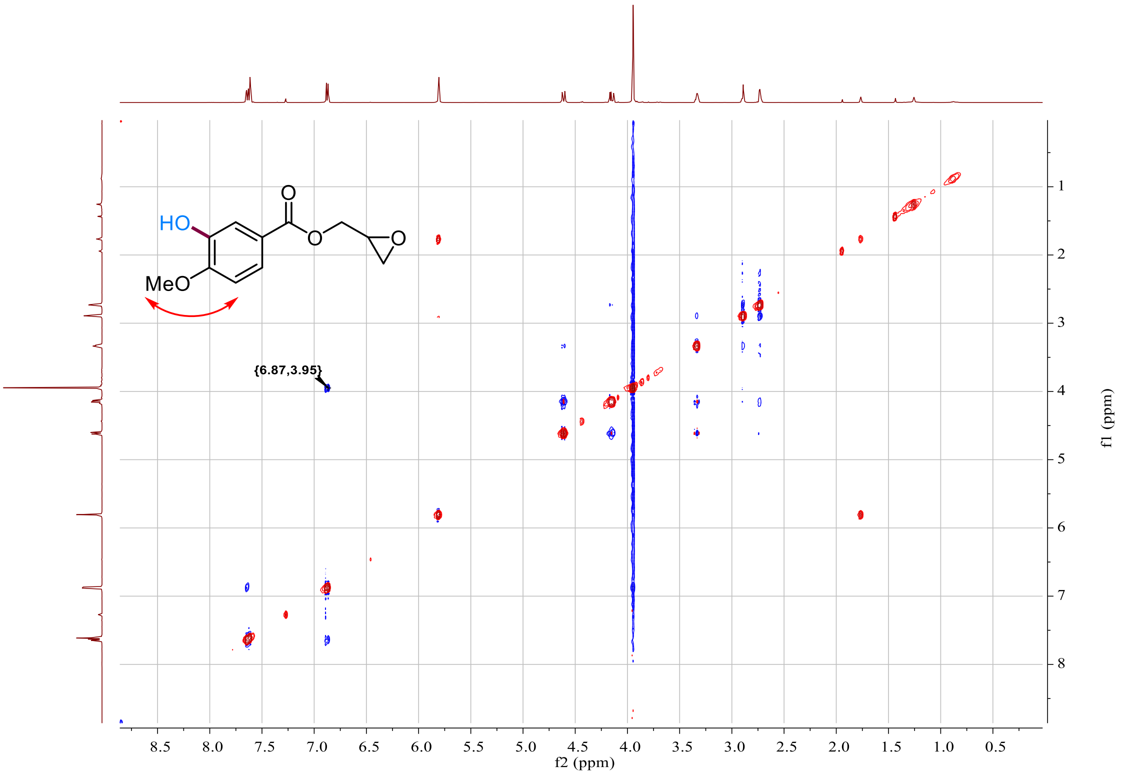

Supplementary Fig. 129. 2D NOESY spectra of compound **46** (500 MHz, rt, $CDCl_3$).

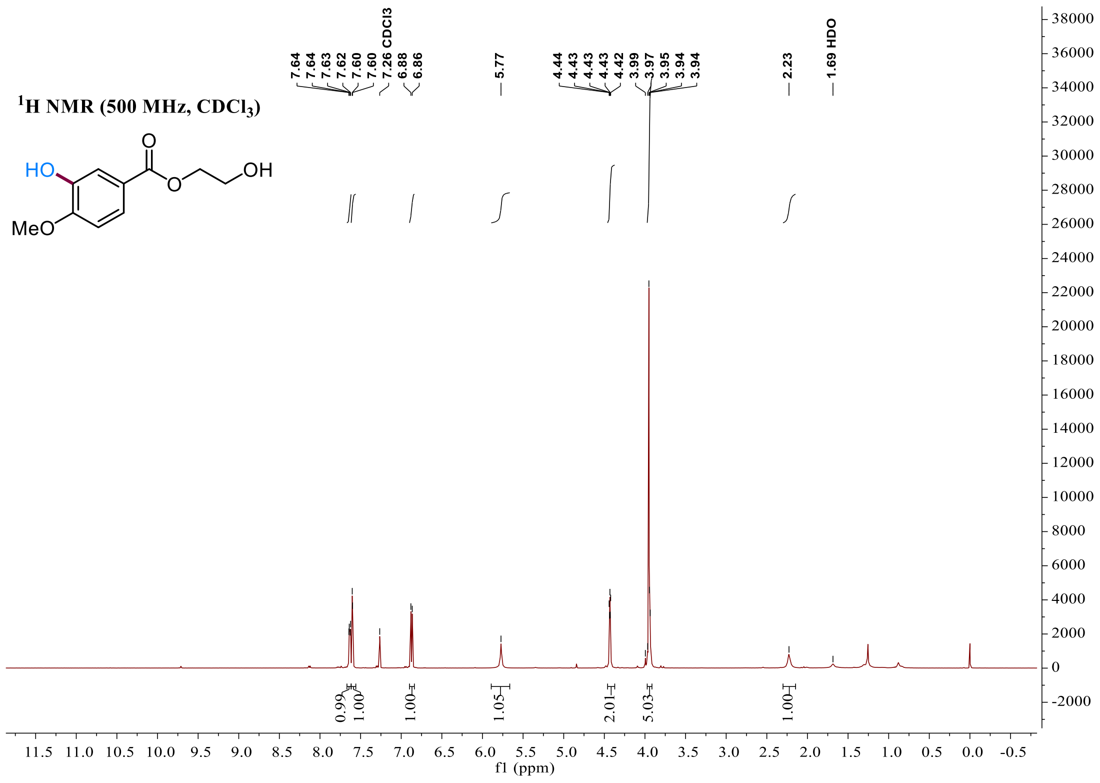

Supplementary Fig. 130. ^{1}H NMR spectra of compound **47** (500 MHz, rt, $CDCl_3$).

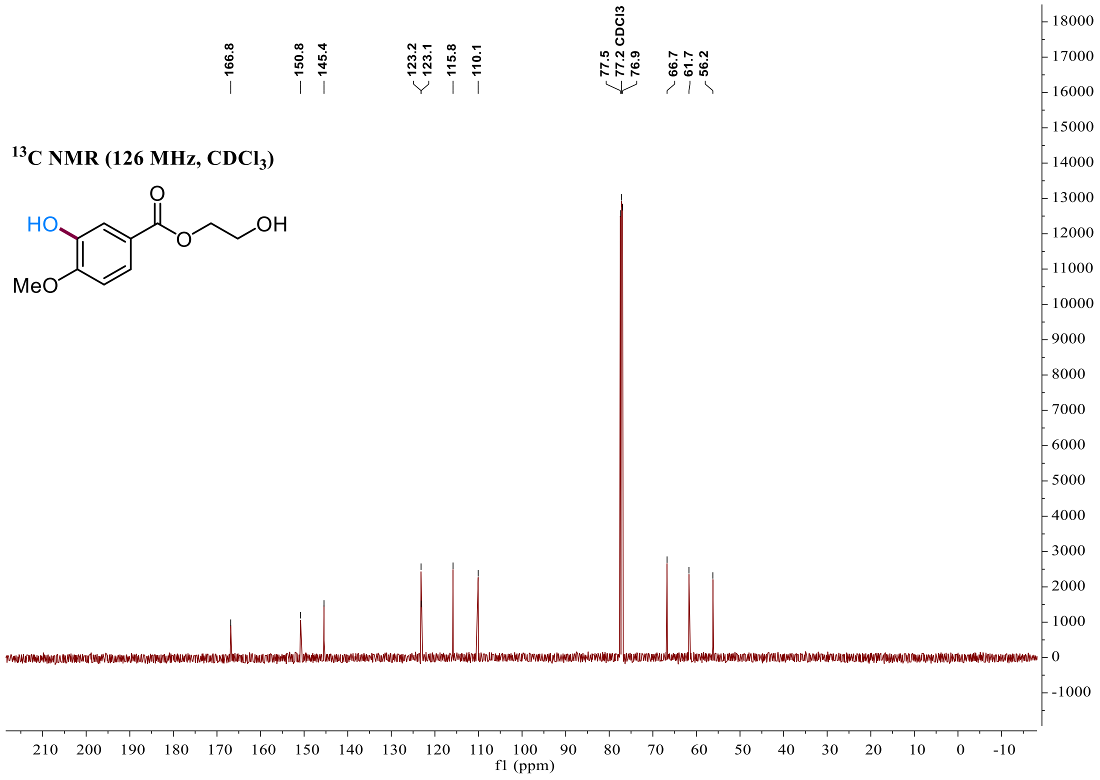

Supplementary Fig. 131. ^{13}C NMR spectra of compound **47** (126 MHz, rt, $CDCl_3$).

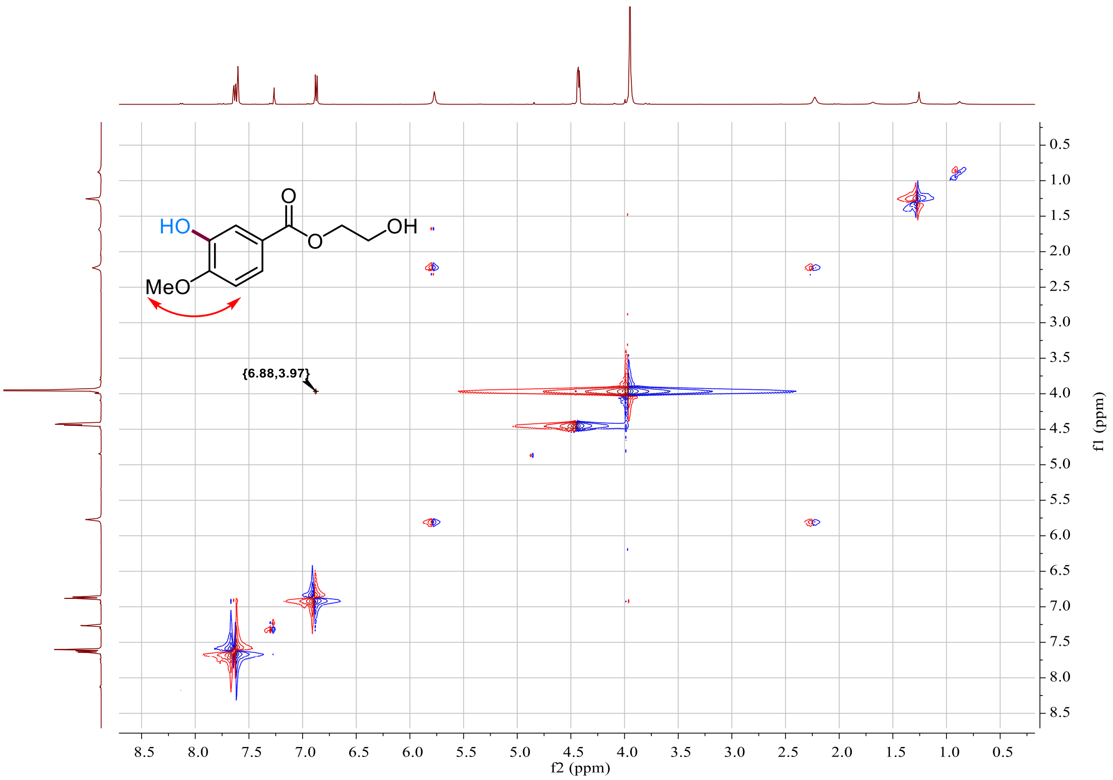

Supplementary Fig. 132. 2D NOESY spectra of compound **47** (500 MHz, rt, $CDCl_3$).

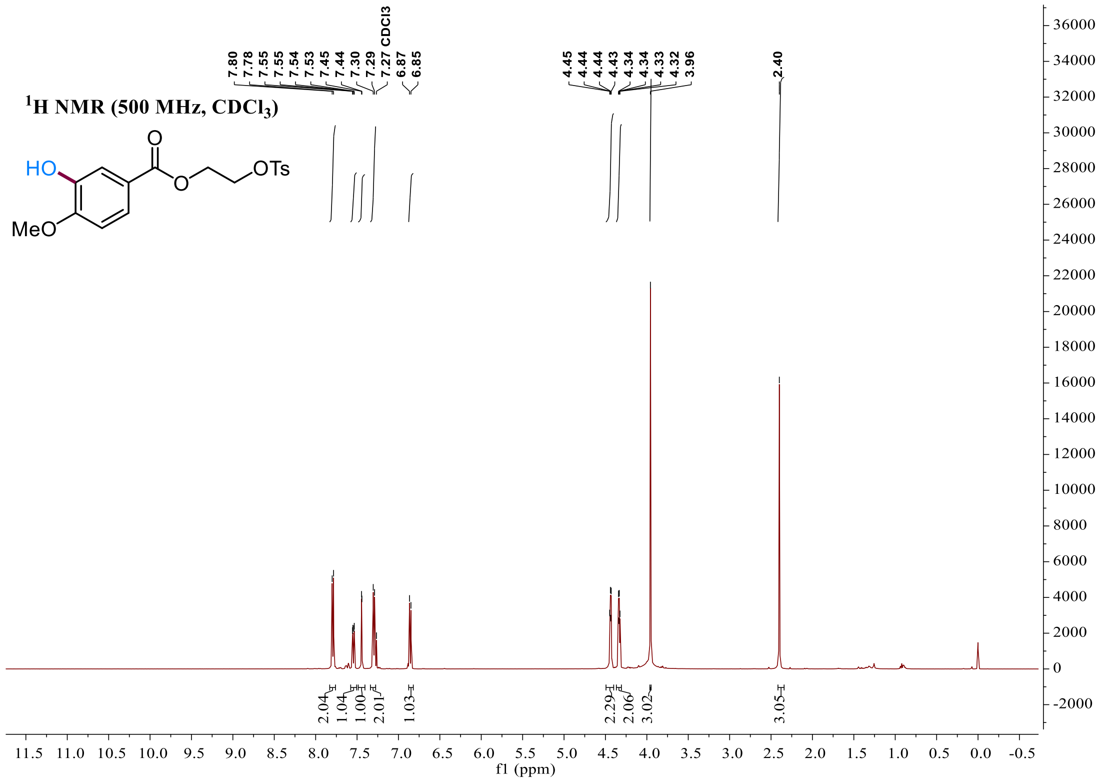

Supplementary Fig. 133. ^{1}H NMR spectra of compound **48** (500 MHz, rt, $CDCl_3$).

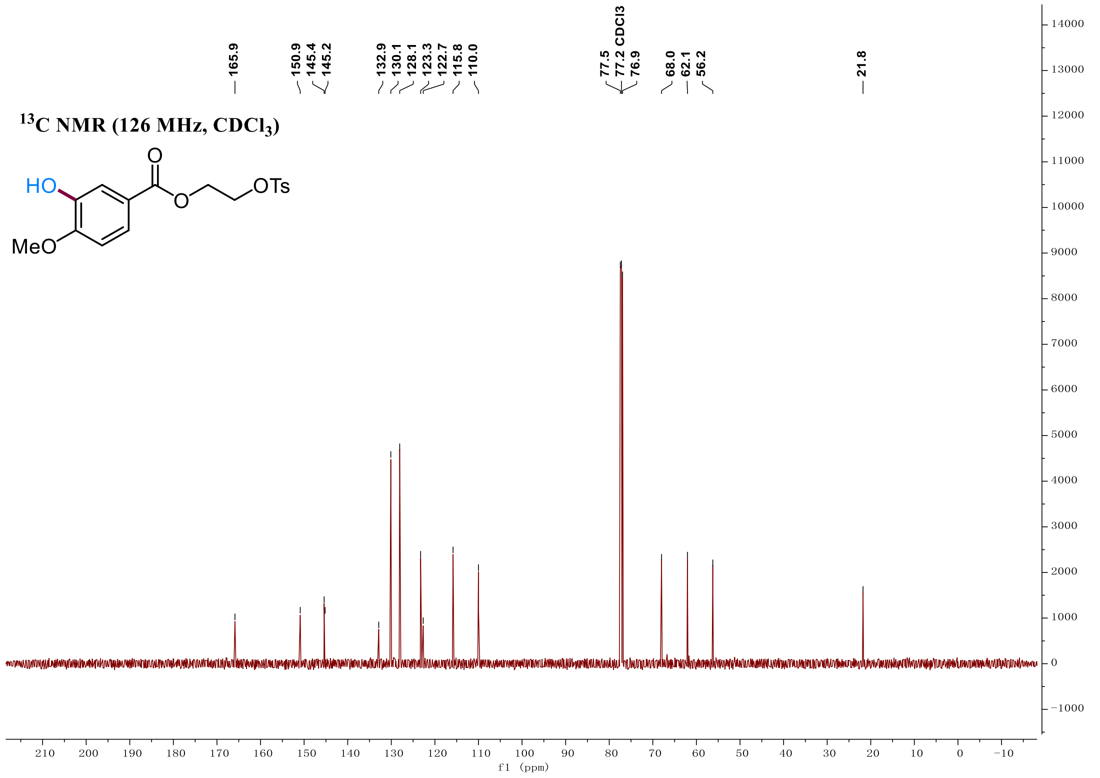

Supplementary Fig. 134. ^{13}C NMR spectra of compound **48** (126 MHz, rt, $CDCl_3$).

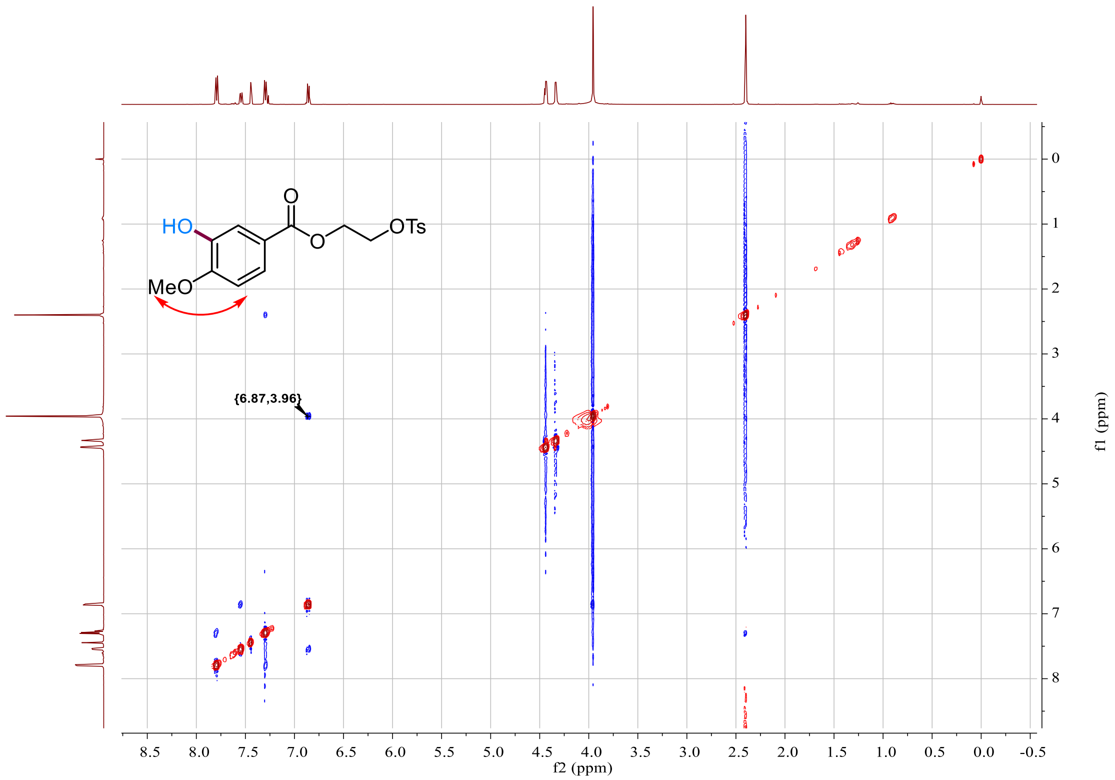

Supplementary Fig. 135. 2D NOESY spectra of compound **48** (500 MHz, rt, $CDCl_3$).

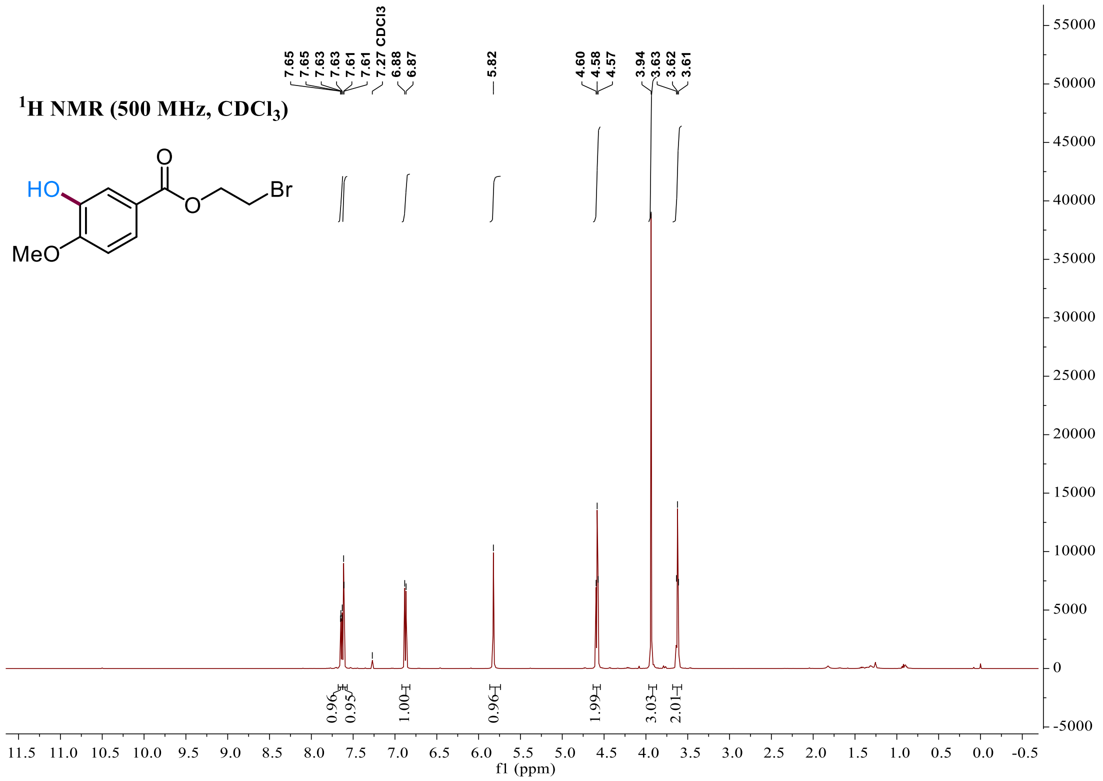

Supplementary Fig. 136. ^{1}H NMR spectra of compound **49** (500 MHz, rt, CDCl$_3$).

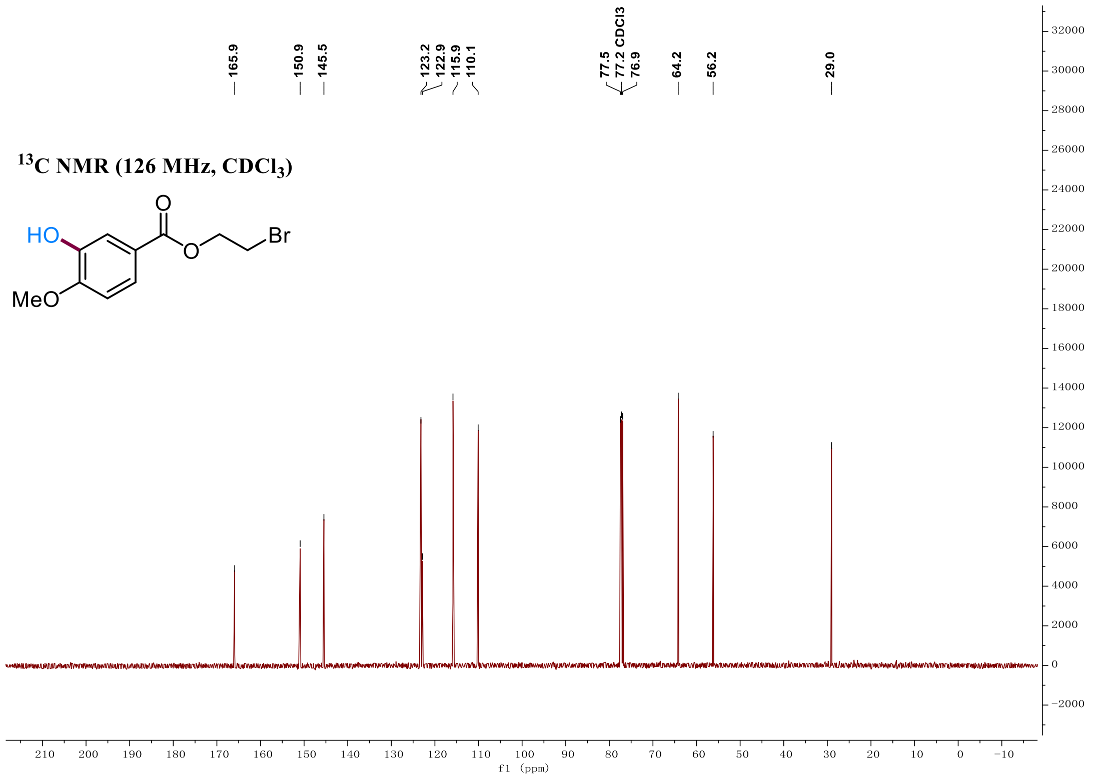

Supplementary Fig. 137. ^{13}C NMR spectra of compound **49** (126 MHz, rt, CDCl$_3$).

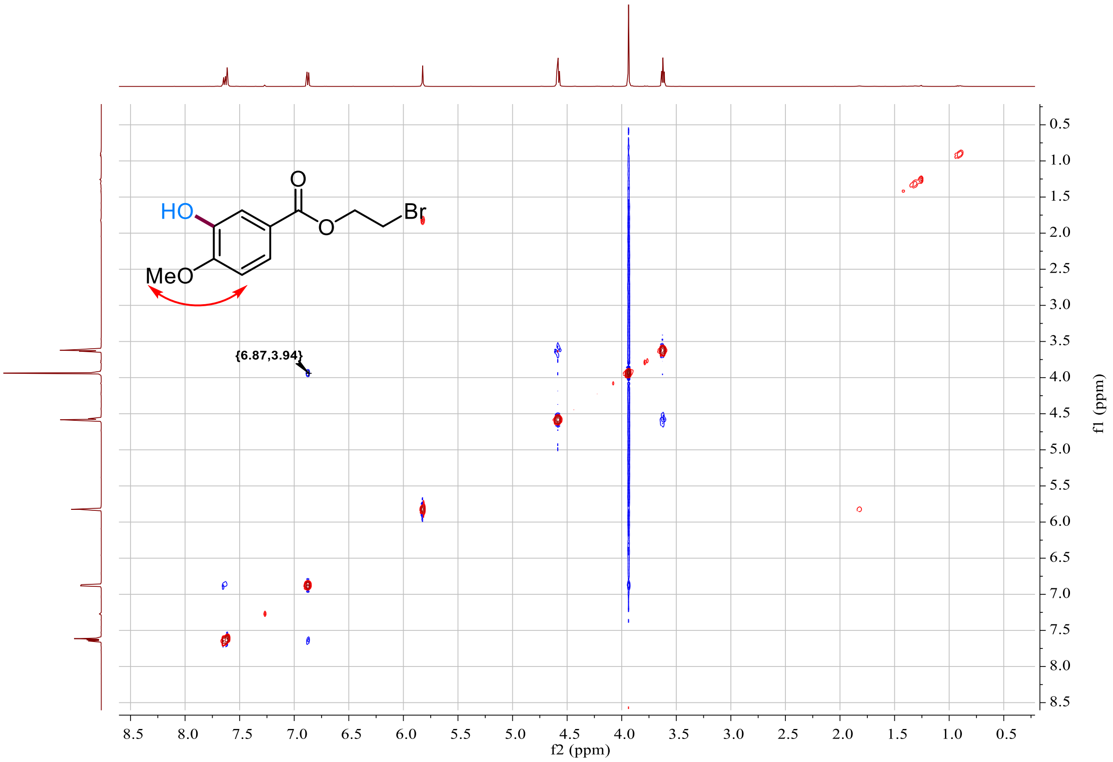

Supplementary Fig. 138. 2D NOESY spectra of compound **49** (500 MHz, rt, $CDCl_3$).

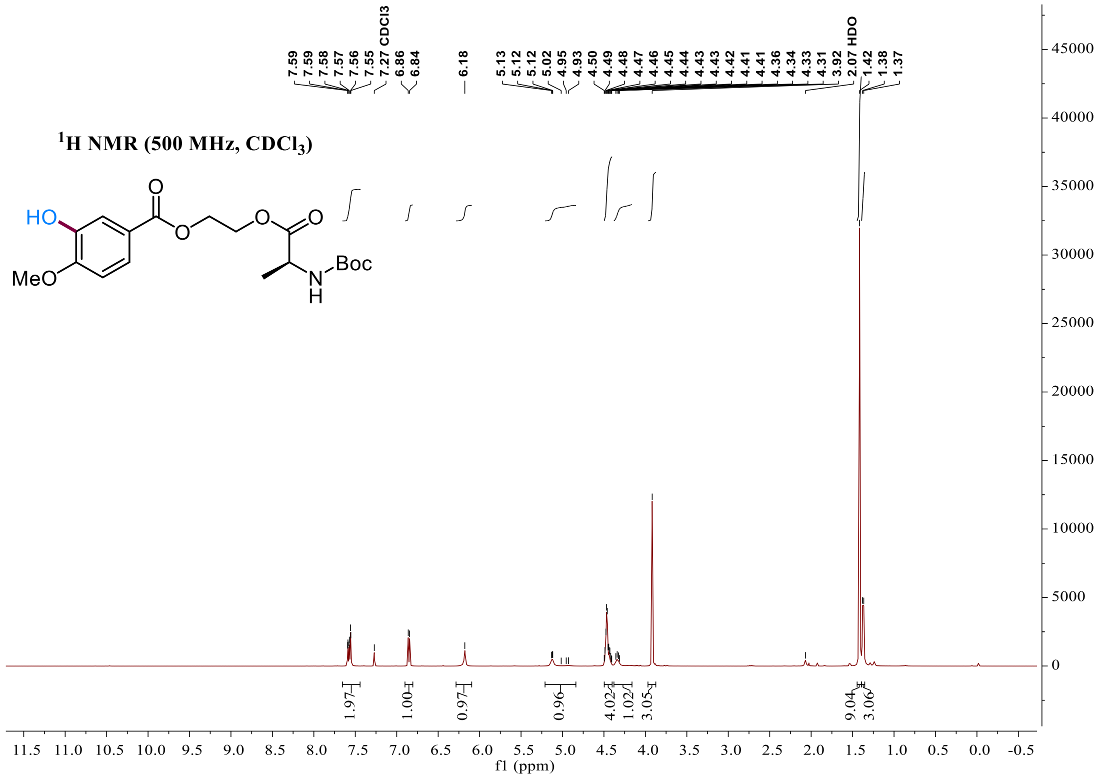

Supplementary Fig. 139. ^{1}H NMR spectra of compound **50** (500 MHz, rt, $CDCl_3$).

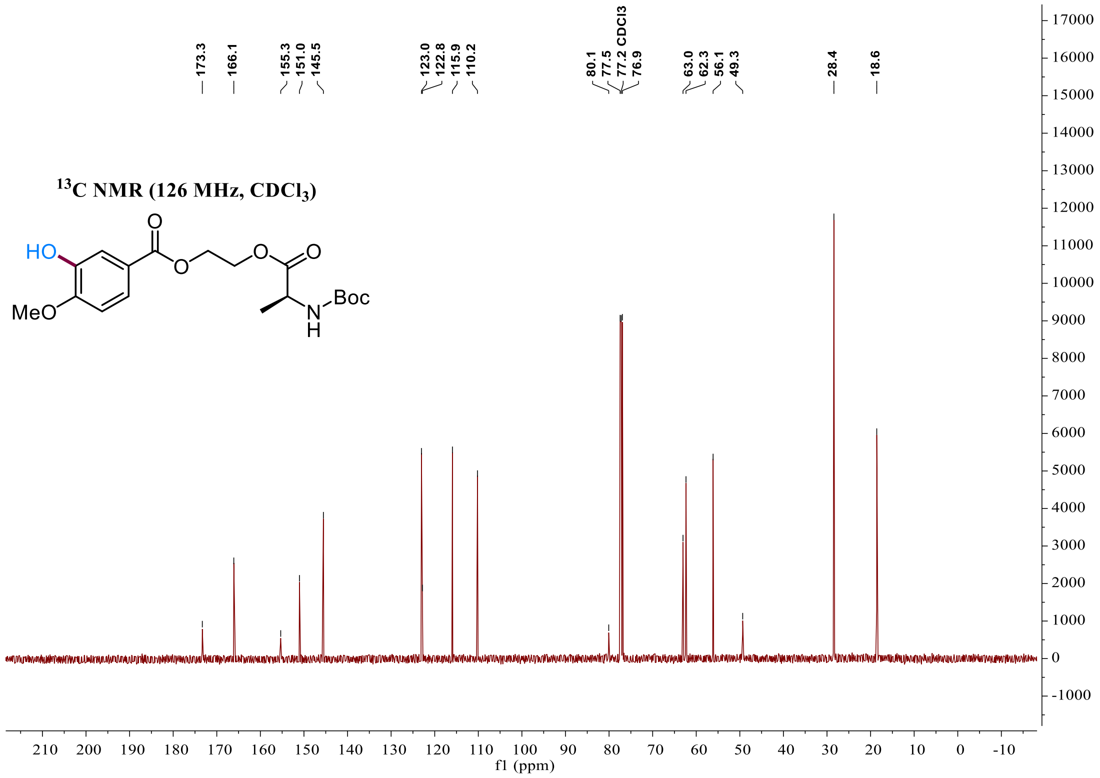

Supplementary Fig. 140. ^{13}C NMR spectra of compound **50** (126 MHz, rt, $CDCl_3$).

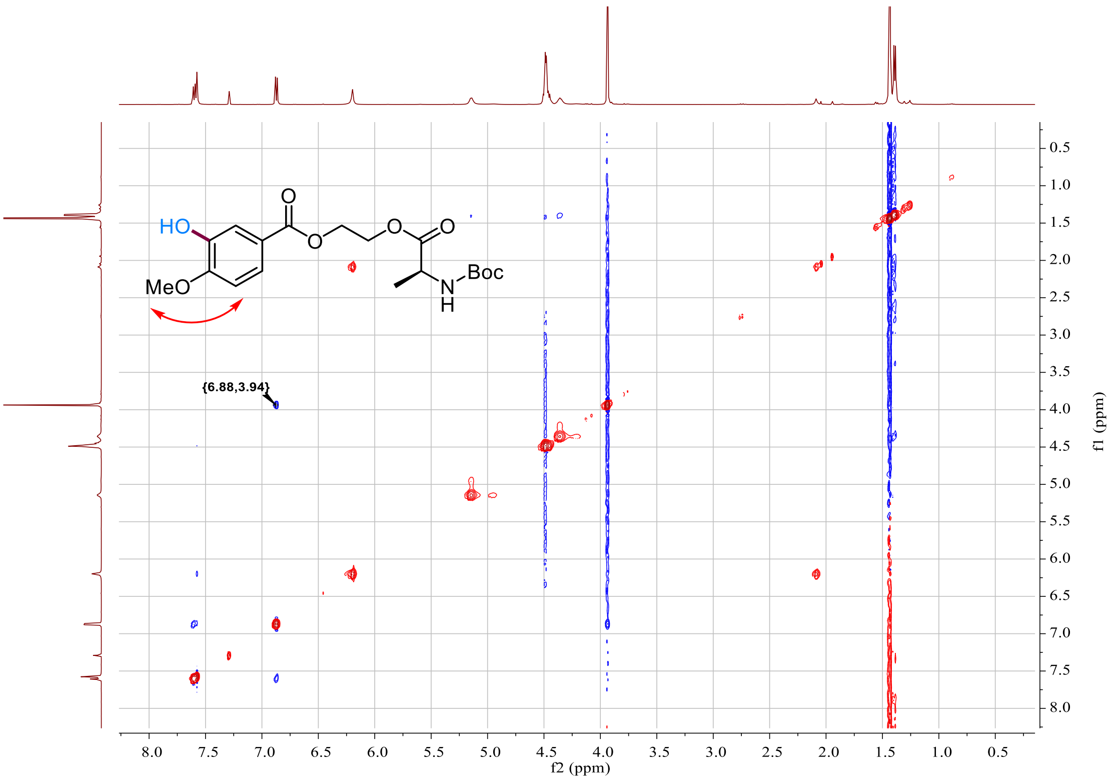

Supplementary Fig. 141. 2D NOESY spectra of compound **50** (500 MHz, rt, $CDCl_3$).

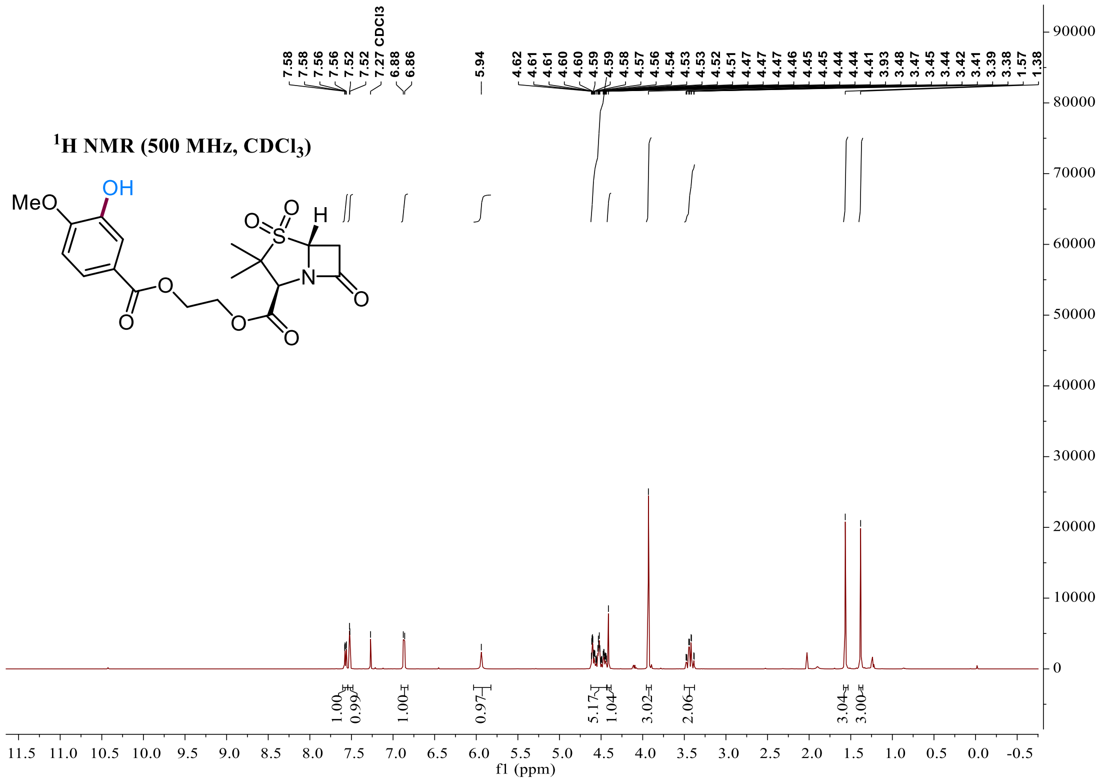

Supplementary Fig. 142. ^{1}H NMR spectra of compound **51** (500 MHz, rt, $CDCl_3$).

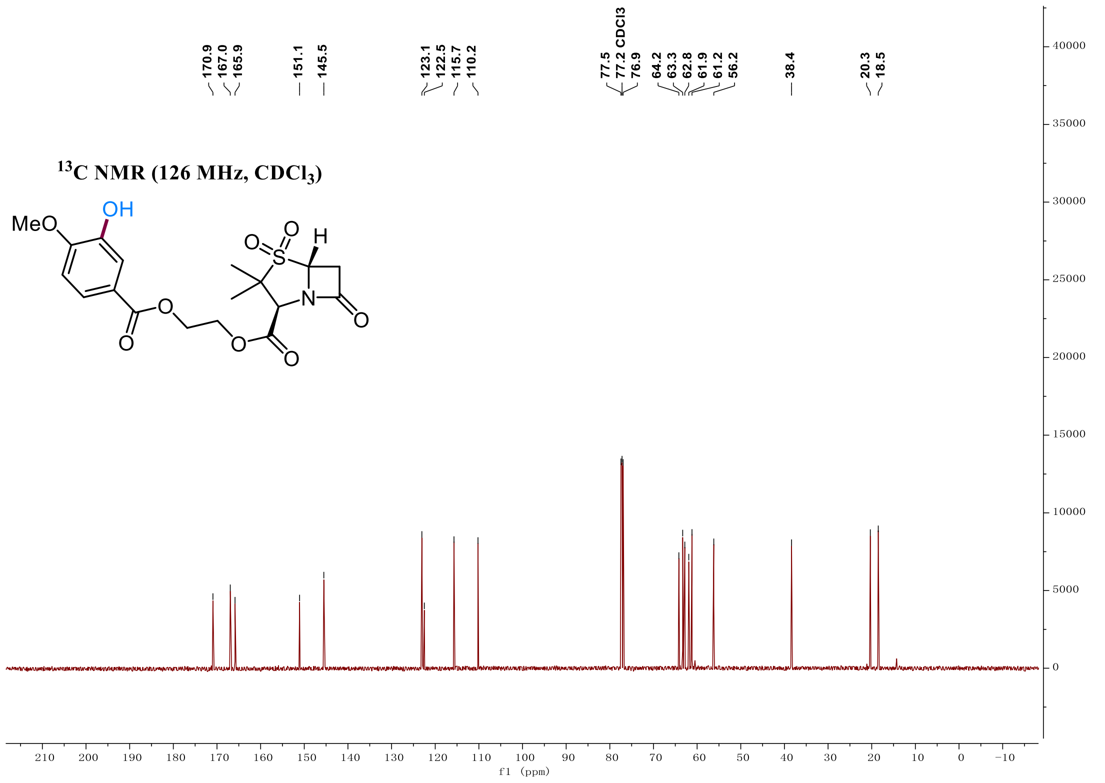

Supplementary Fig. 143. ^{13}C NMR spectra of compound **51** (126 MHz, rt, $CDCl_3$).

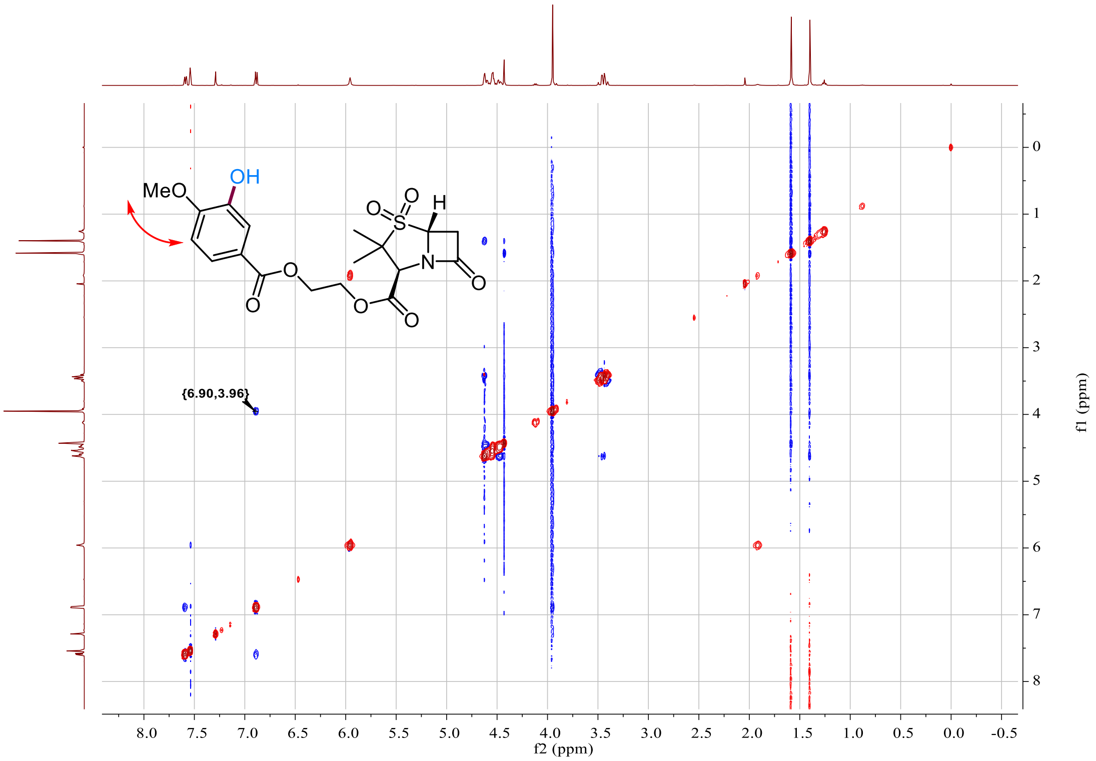

Supplementary Fig. 144. 2D NOESY spectra of compound **51** (500 MHz, rt, $CDCl_3$).

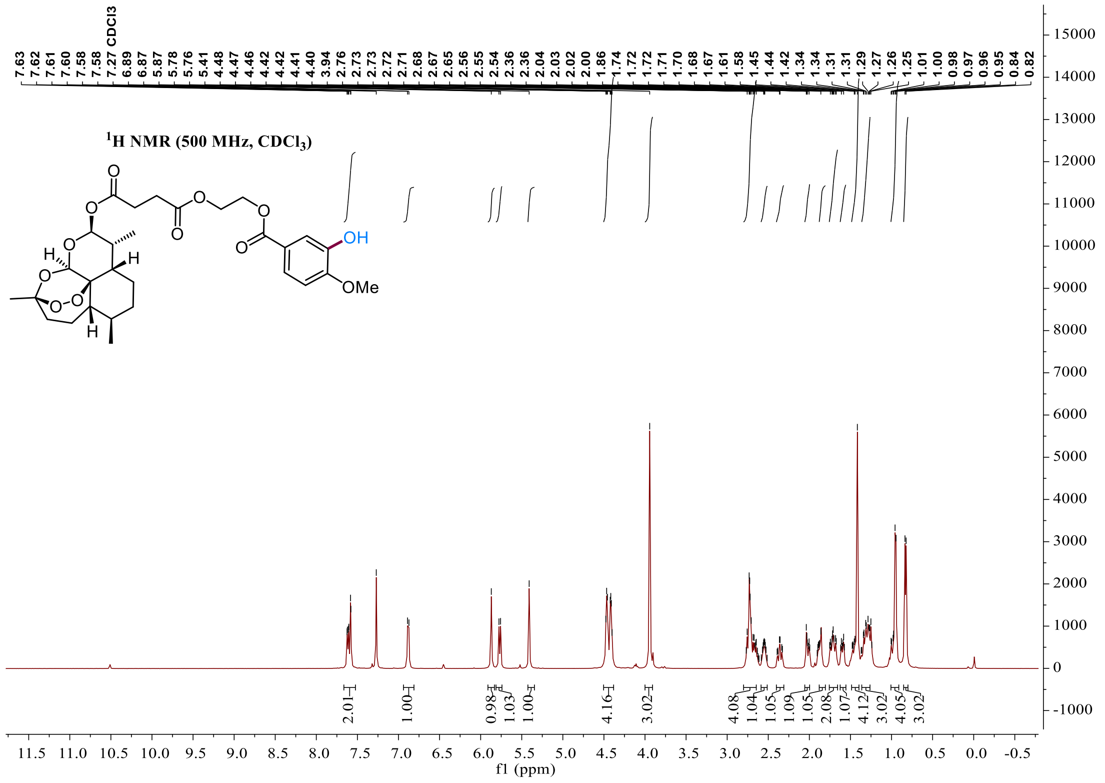

Supplementary Fig. 145. ^{1}H NMR spectra of compound **52** (500 MHz, rt, $CDCl_3$).

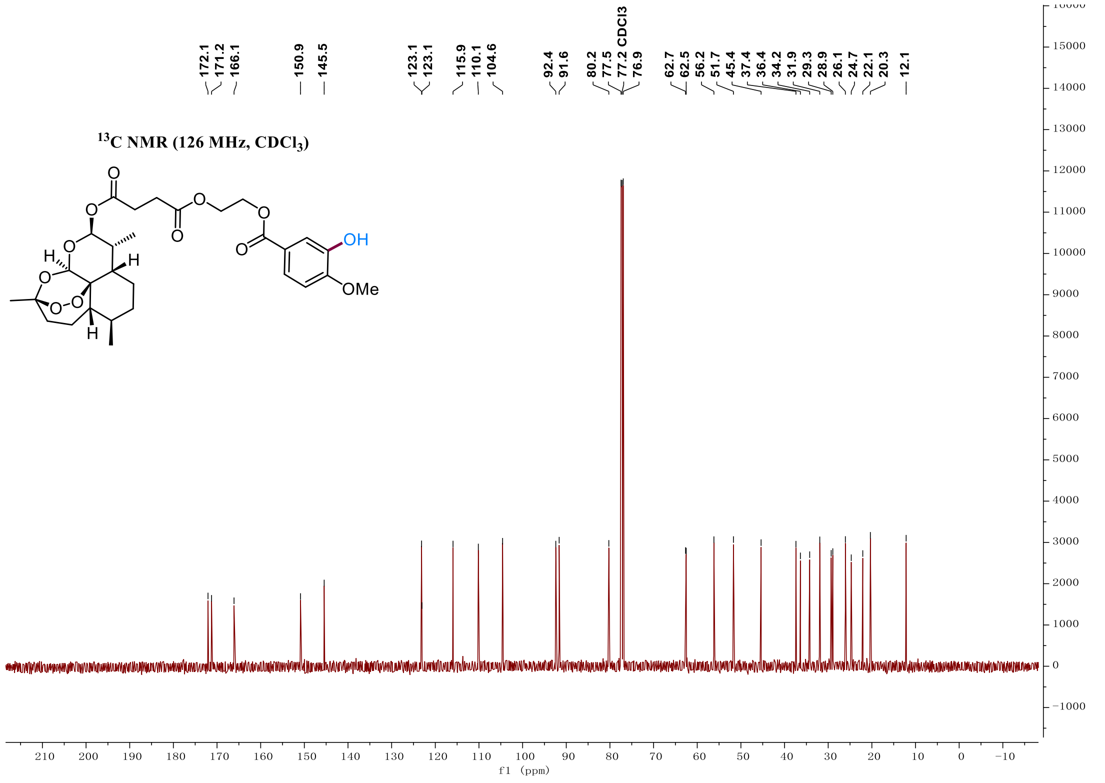

Supplementary Fig. 146. ^{13}C NMR spectra of compound **52** (126 MHz, rt, $CDCl_3$).

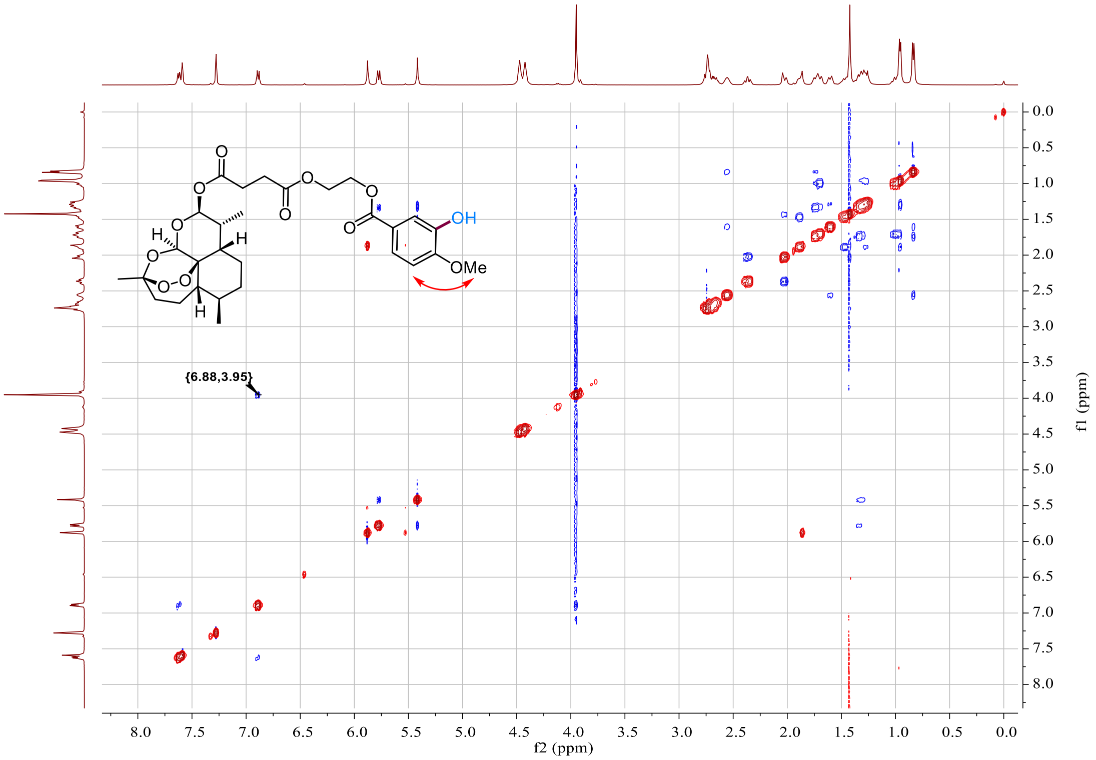

Supplementary Fig. 147. 2D NOESY spectra of compound **52** (500 MHz, rt, $CDCl_3$).

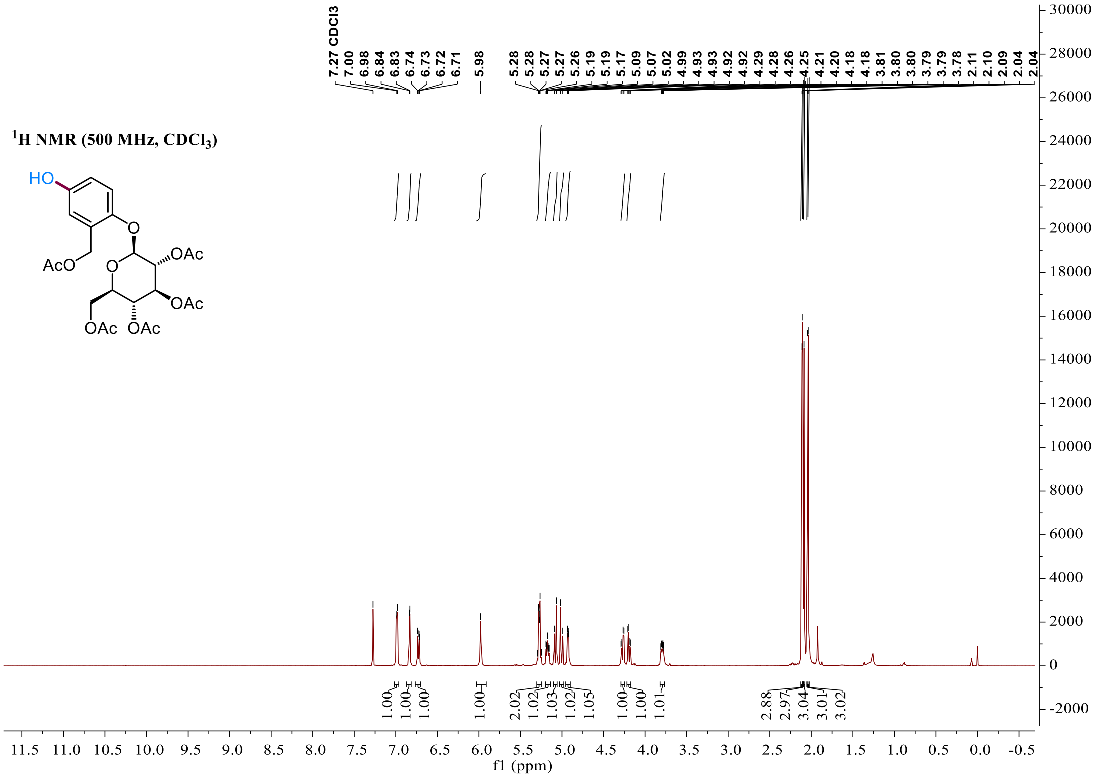

Supplementary Fig. 148. ^{1}H NMR spectra of compound **53** (500 MHz, rt, $CDCl_3$).

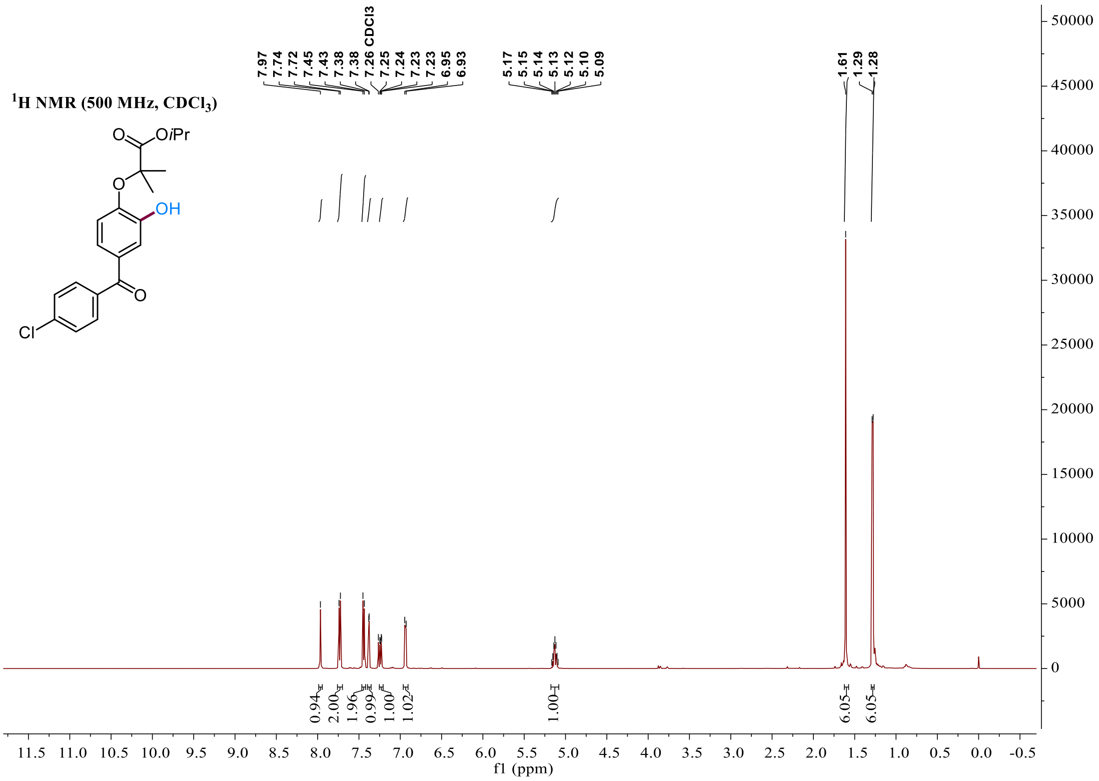

Supplementary Fig. 149. ^{1}H NMR spectra of compound **54** (500 MHz, rt, $CDCl_3$).

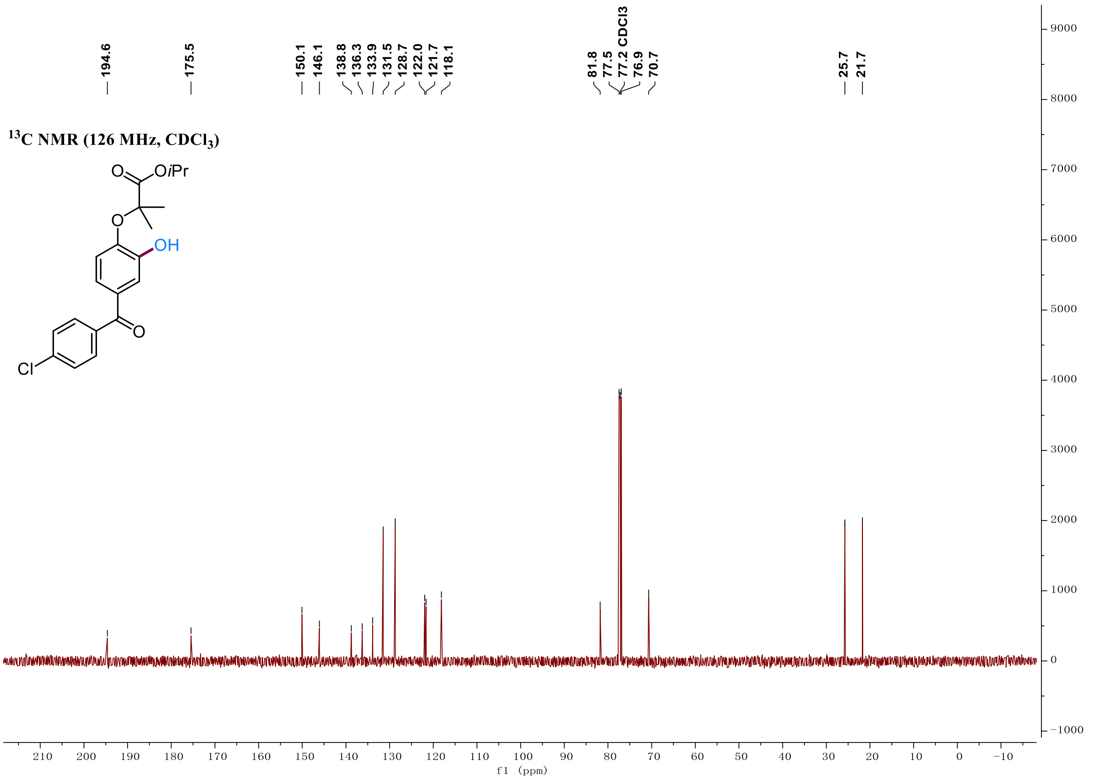

Supplementary Fig. 150. ^{13}C NMR spectra of compound **54** (126 MHz, rt, $CDCl_3$).

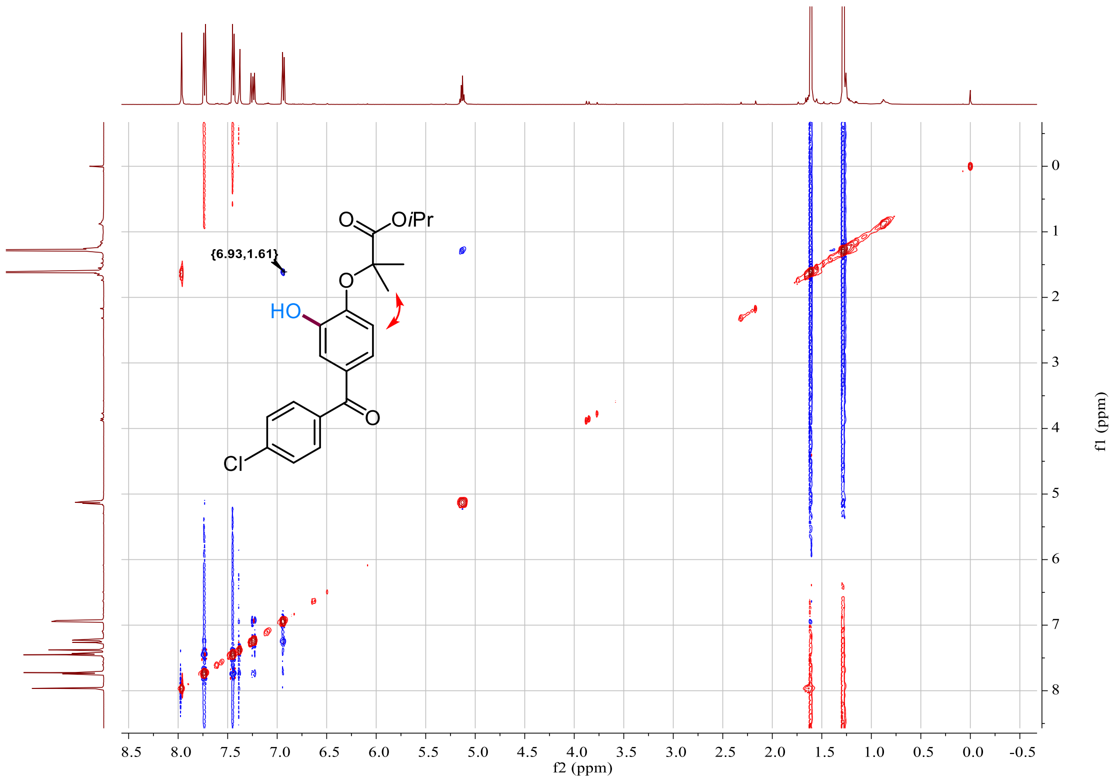

Supplementary Fig. 151. 2D NOESY spectra of compound **54** (500 MHz, rt, $CDCl_3$).

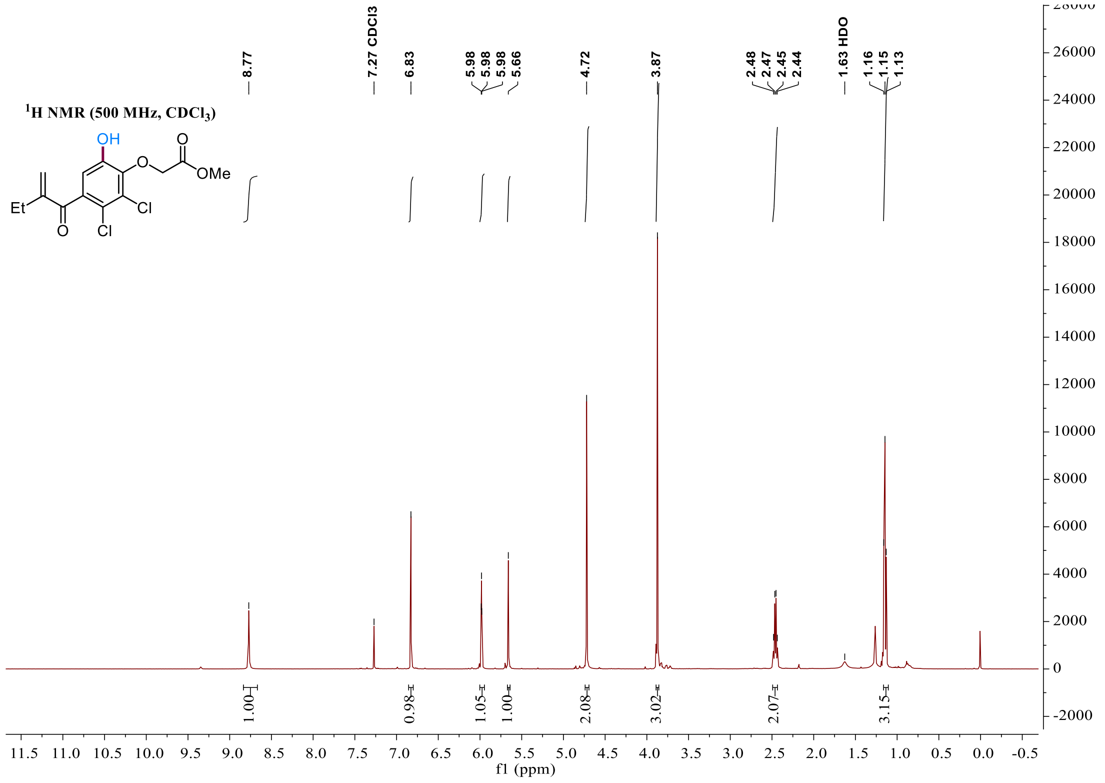

Supplementary Fig. 152. ^{1}H NMR spectra of compound **55** (500 MHz, rt, $CDCl_3$).

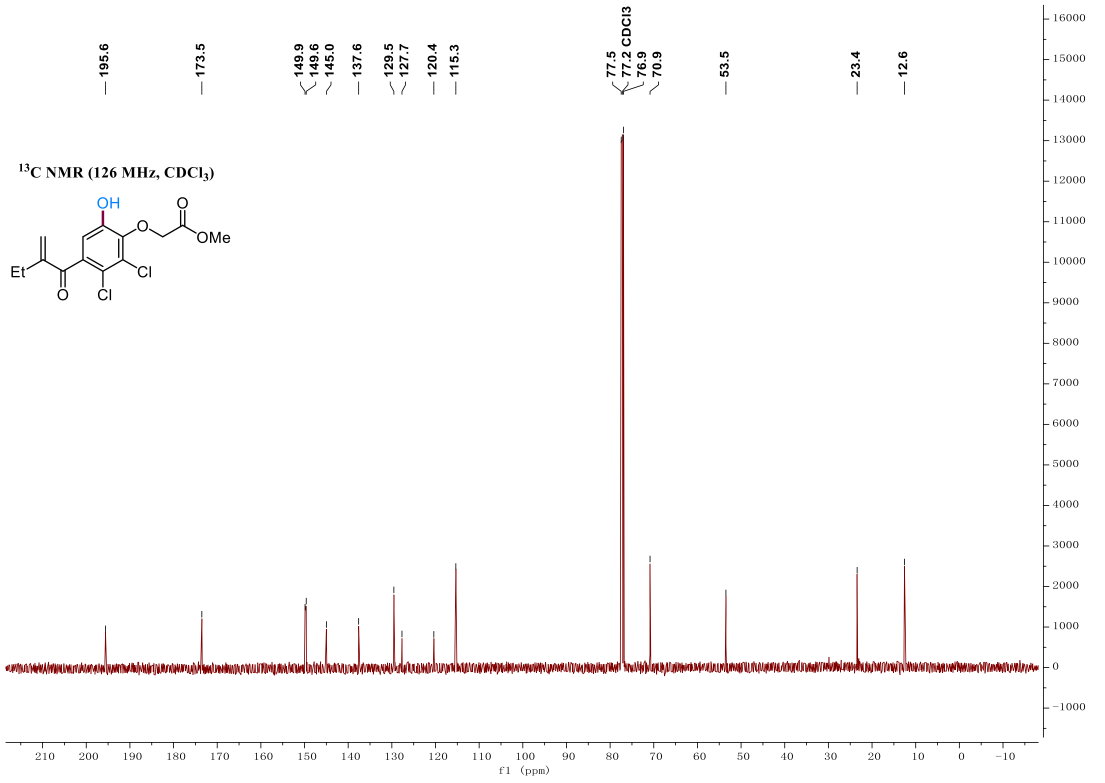

Supplementary Fig. 153. ^{13}C NMR spectra of compound **55** (126 MHz, rt, $CDCl_3$).

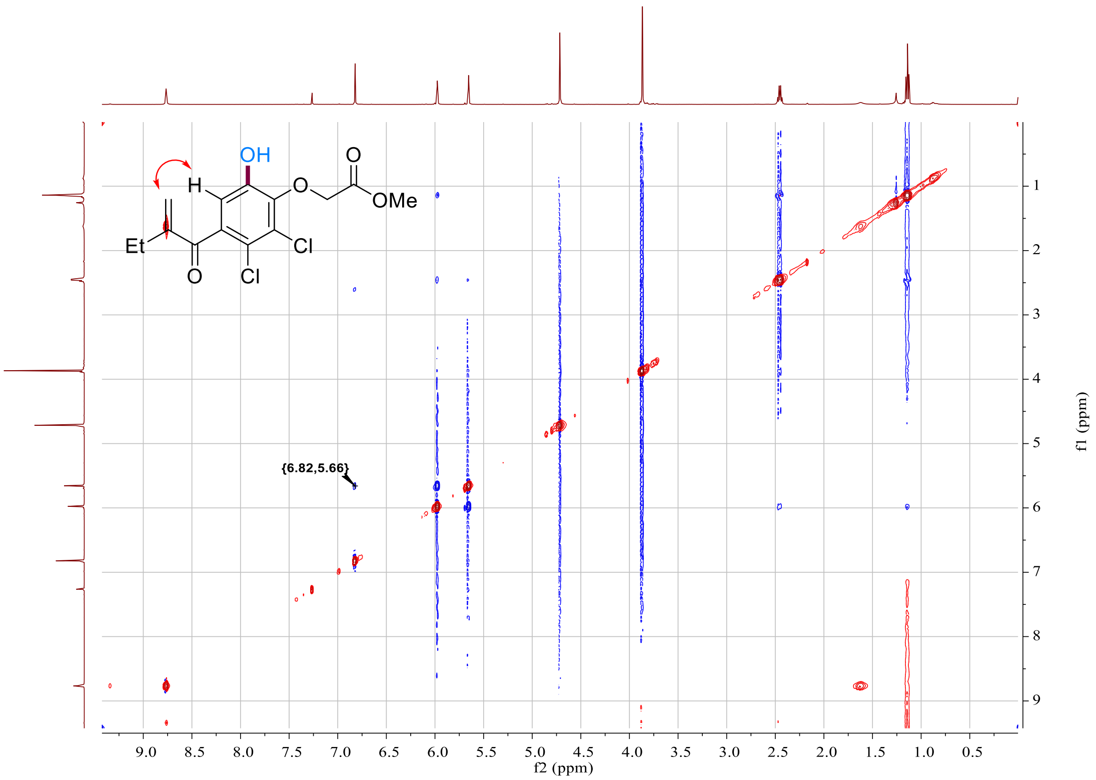

Supplementary Fig. 154. 2D NOESY spectra of compound **55** (500 MHz, rt, $CDCl_3$).

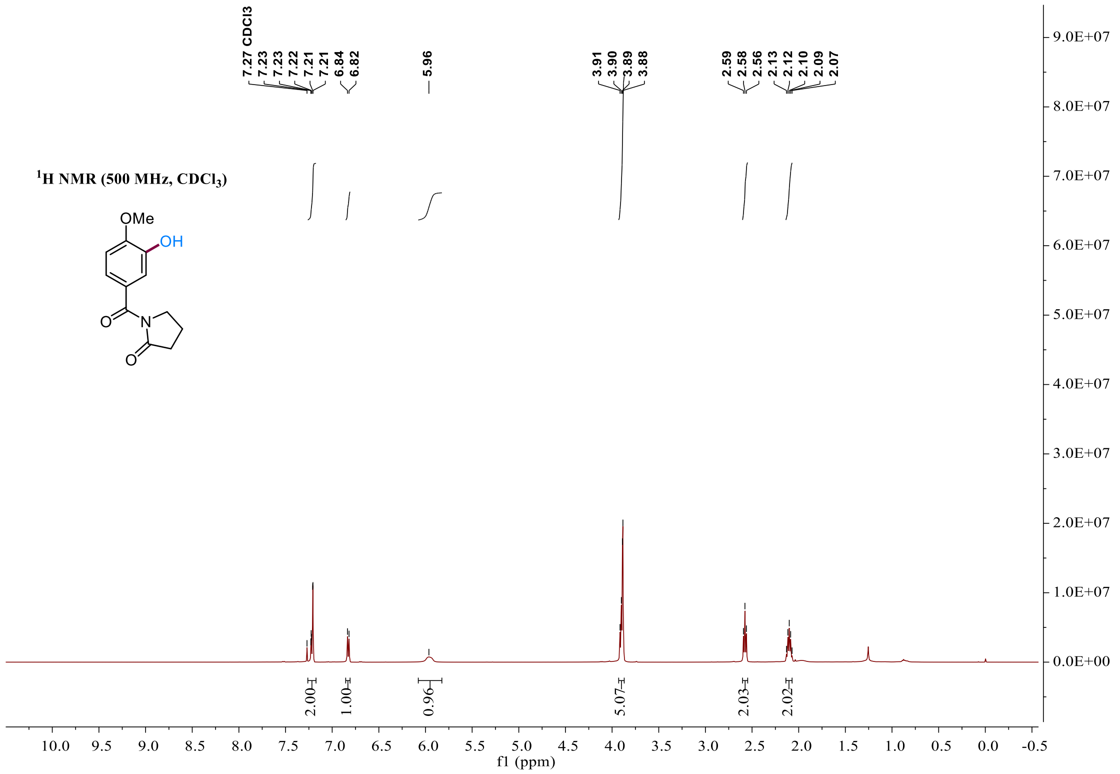

Supplementary Fig. 155. ^{1}H NMR spectra of compound **56** (500 MHz, rt, $CDCl_3$).

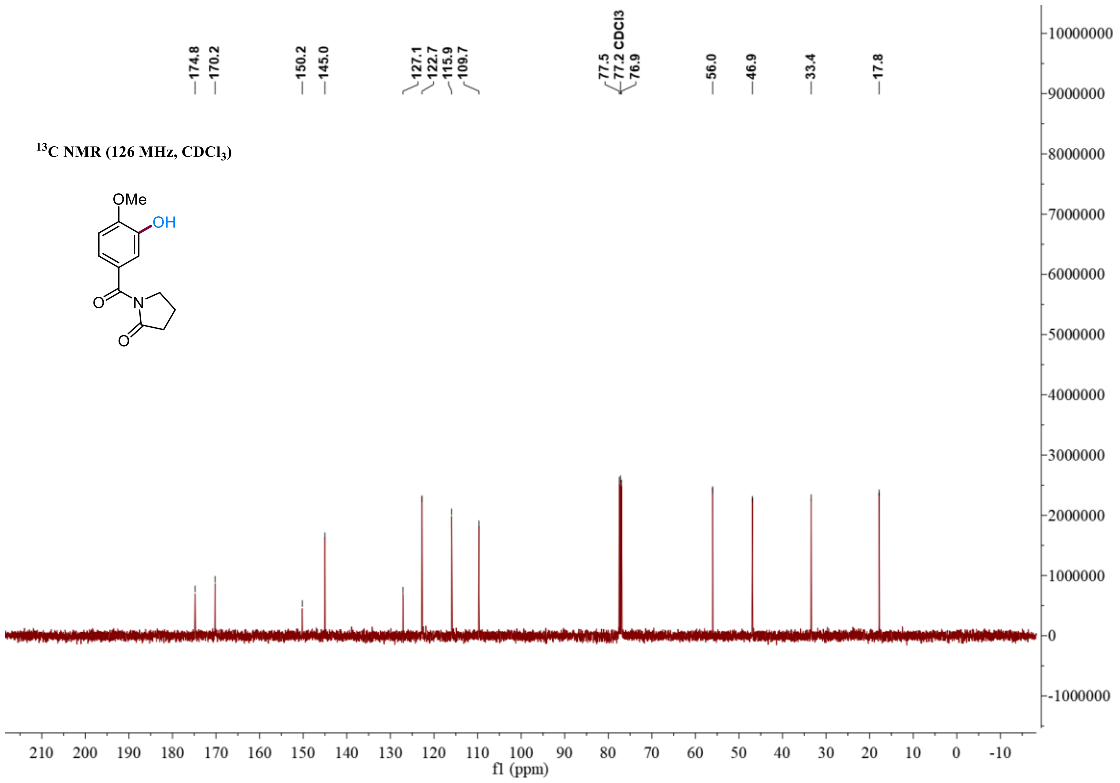

Supplementary Fig. 156. ^{13}C NMR spectra of compound **56** (126 MHz, rt, $CDCl_3$).

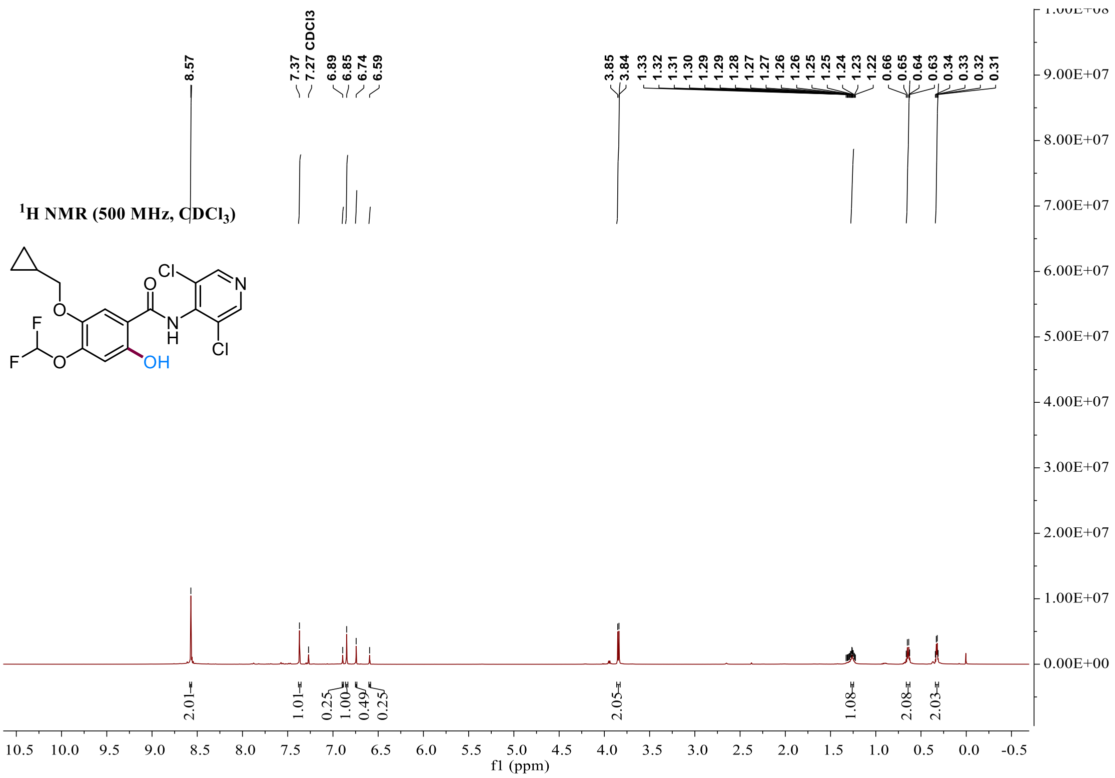

Supplementary Fig. 157. ^{1}H NMR spectra of compound **57** (500 MHz, rt, $CDCl_3$).

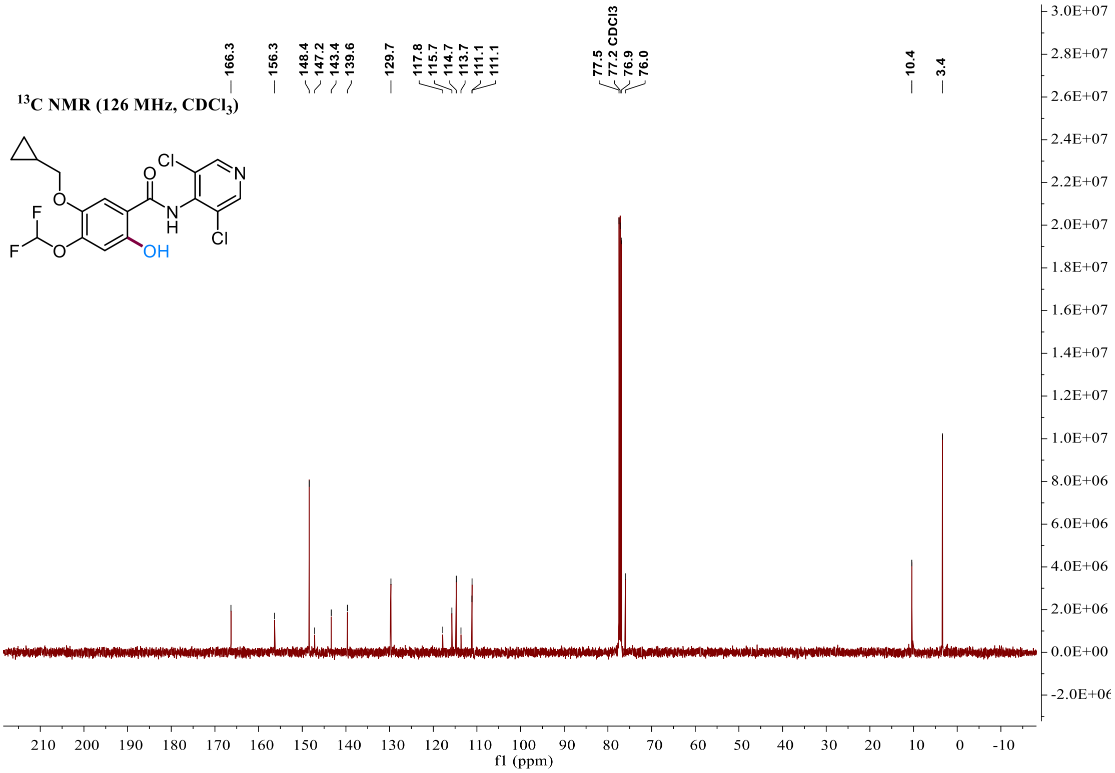

Supplementary Fig. 158. ^{13}C NMR spectra of compound **57** (126 MHz, rt, $CDCl_3$).

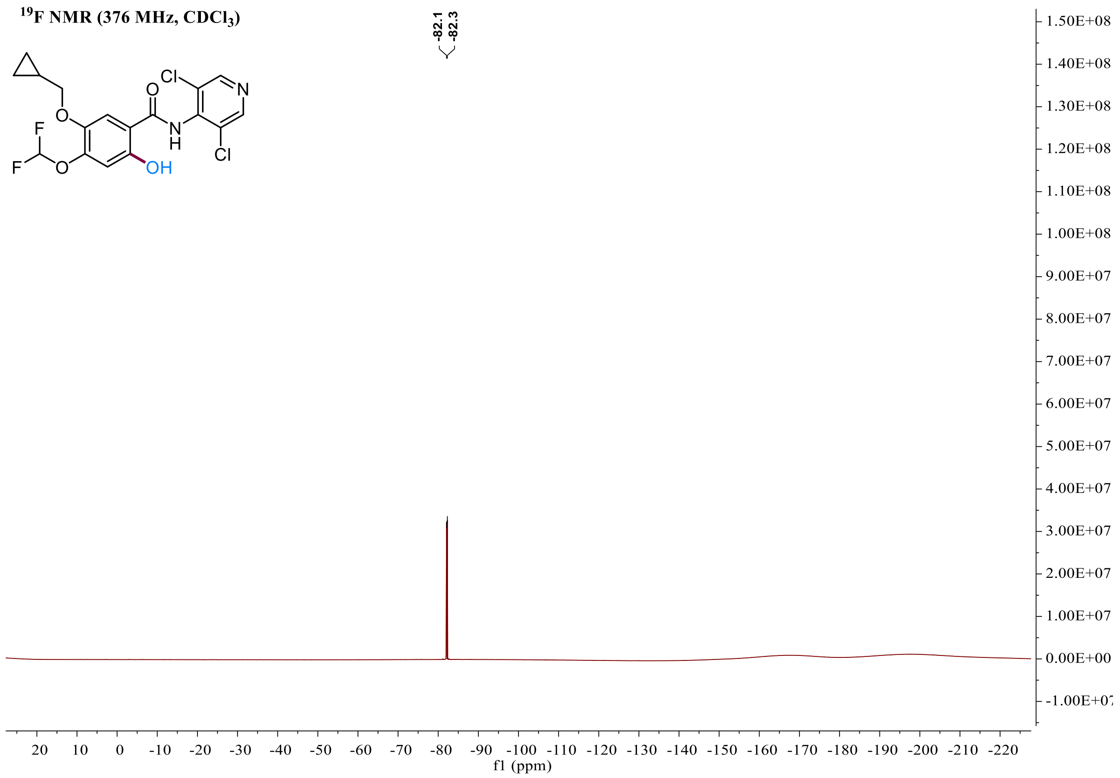

Supplementary Fig. 159. ^{19}F NMR spectra of compound **57** (376 MHz, rt, $CDCl_3$).

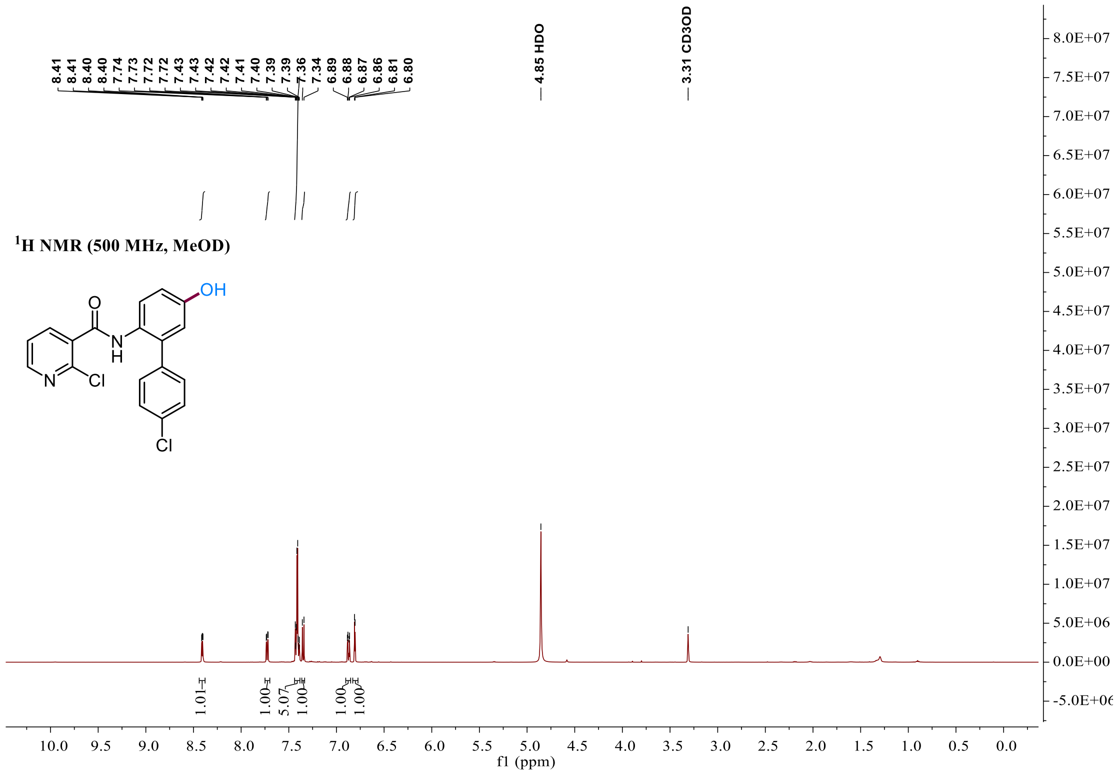

Supplementary Fig. 160. ^{1}H NMR spectra of compound **58** (500 MHz, rt, MeOD).

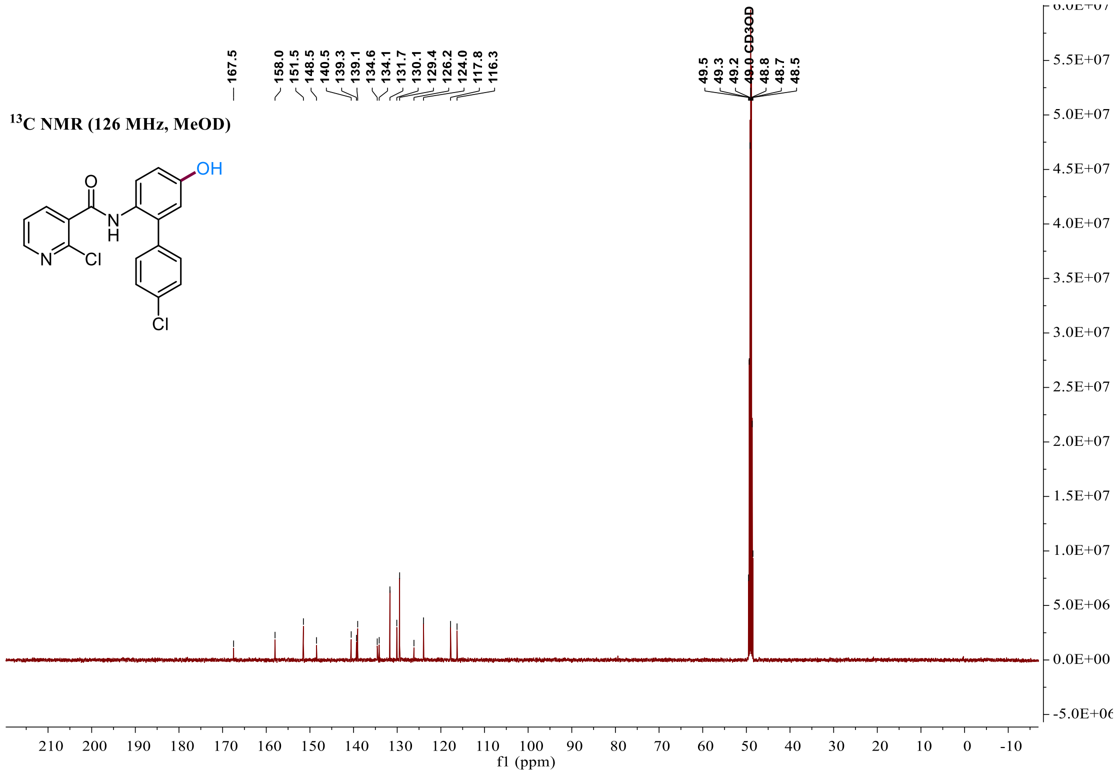

Supplementary Fig. 161. ^{13}C NMR spectra of compound **58** (126 MHz, rt, MeOD).

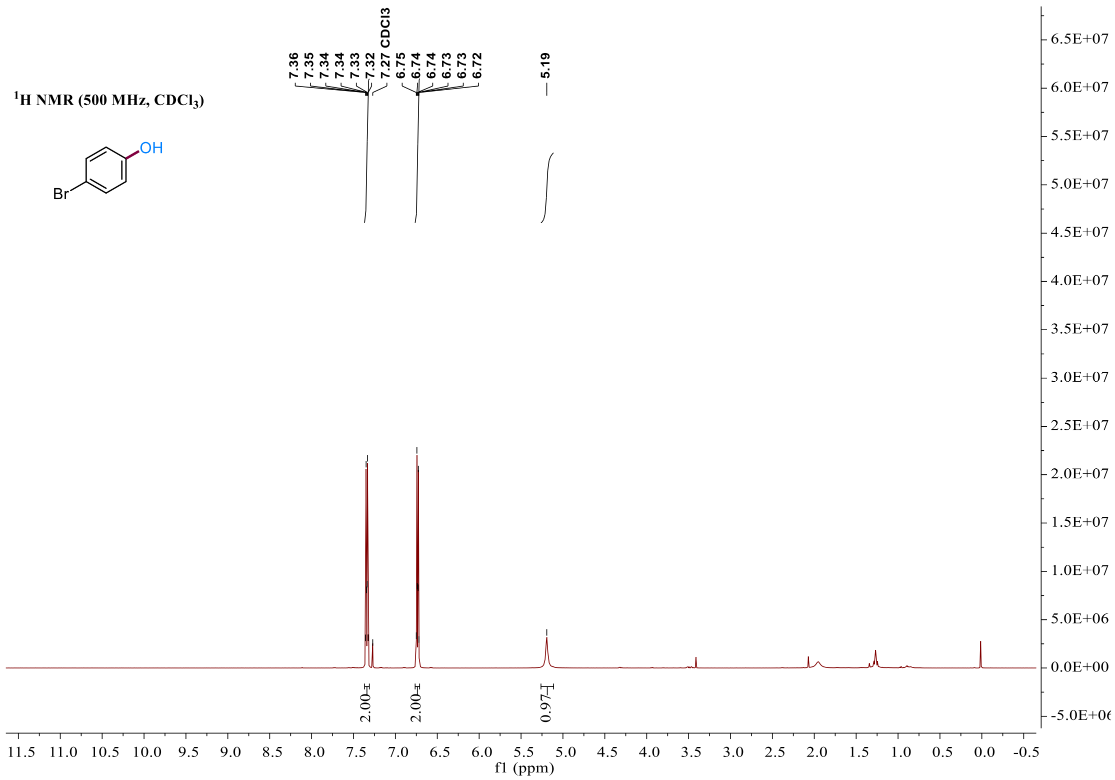

Supplementary Fig. 162. ^{1}H NMR spectra of compound **59** (500 MHz, rt, $CDCl_3$).

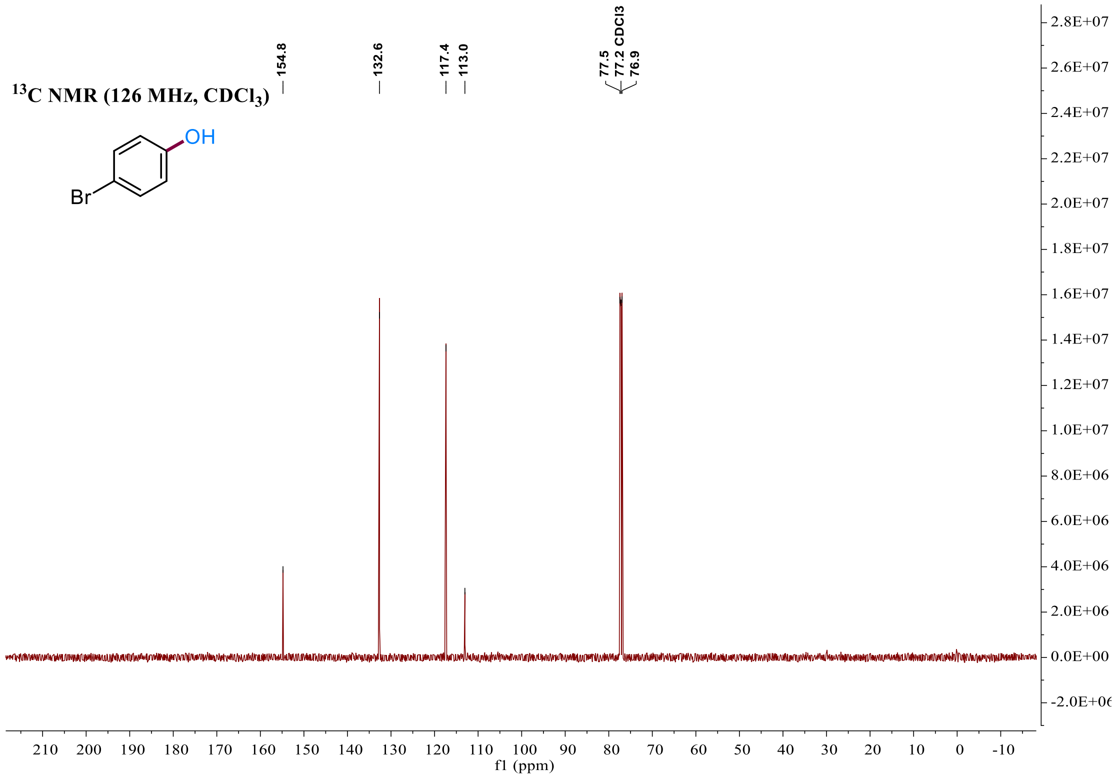

Supplementary Fig. 163. ^{13}C NMR spectra of compound **59** (126 MHz, rt, $CDCl_3$).

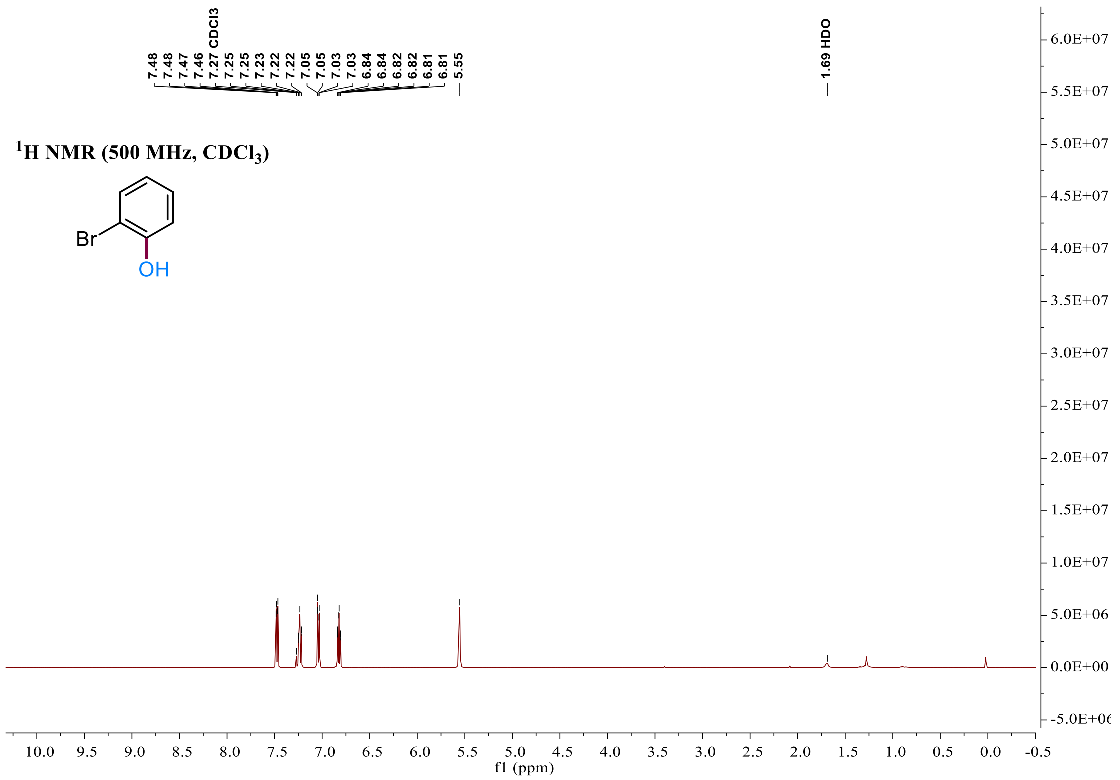

Supplementary Fig. 164. ^{1}H NMR spectra of compound **59** (500 MHz, rt, CDCl$_3$).

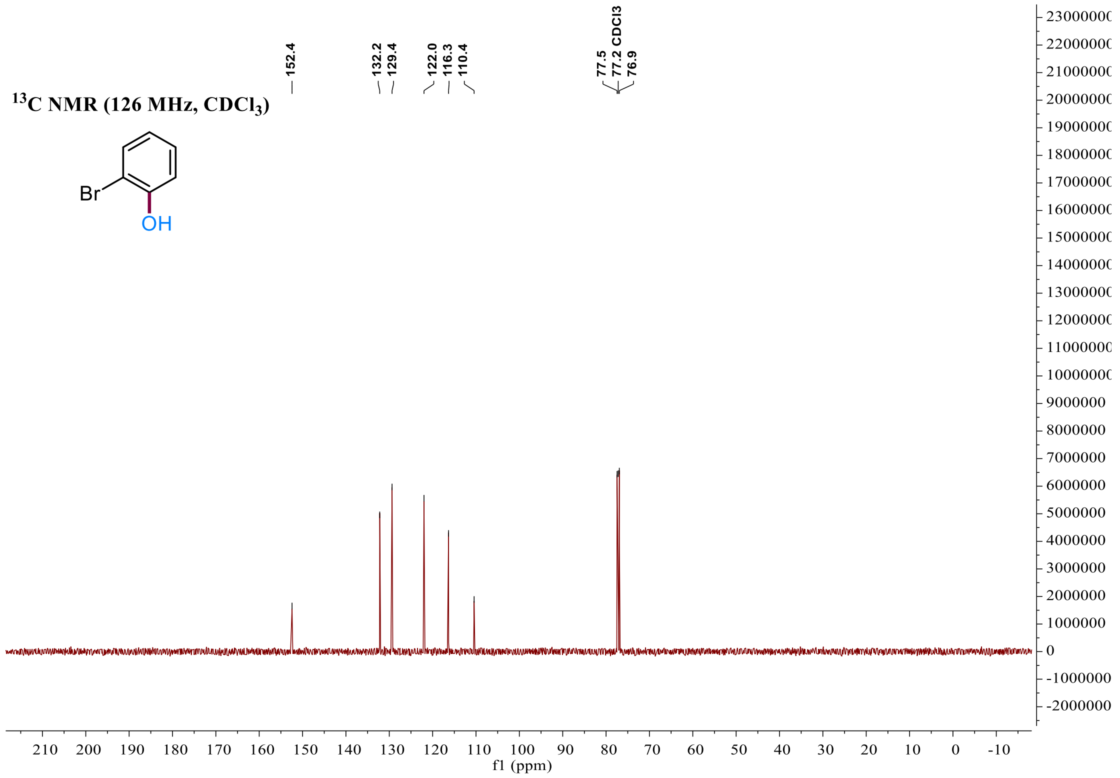

Supplementary Fig. 165. ^{13}C NMR spectra of compound **59** (126 MHz, rt, CDCl$_3$).

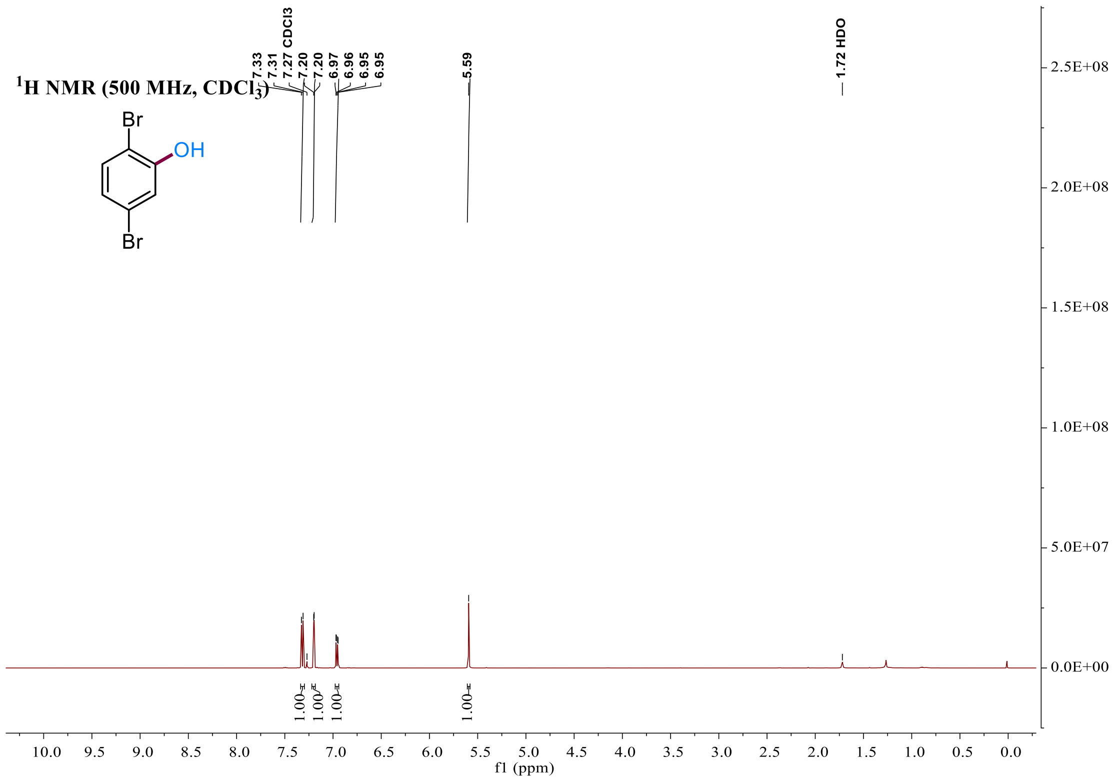

Supplementary Fig. 166. ^{1}H NMR spectra of compound **60** (500 MHz, rt, $CDCl_3$).

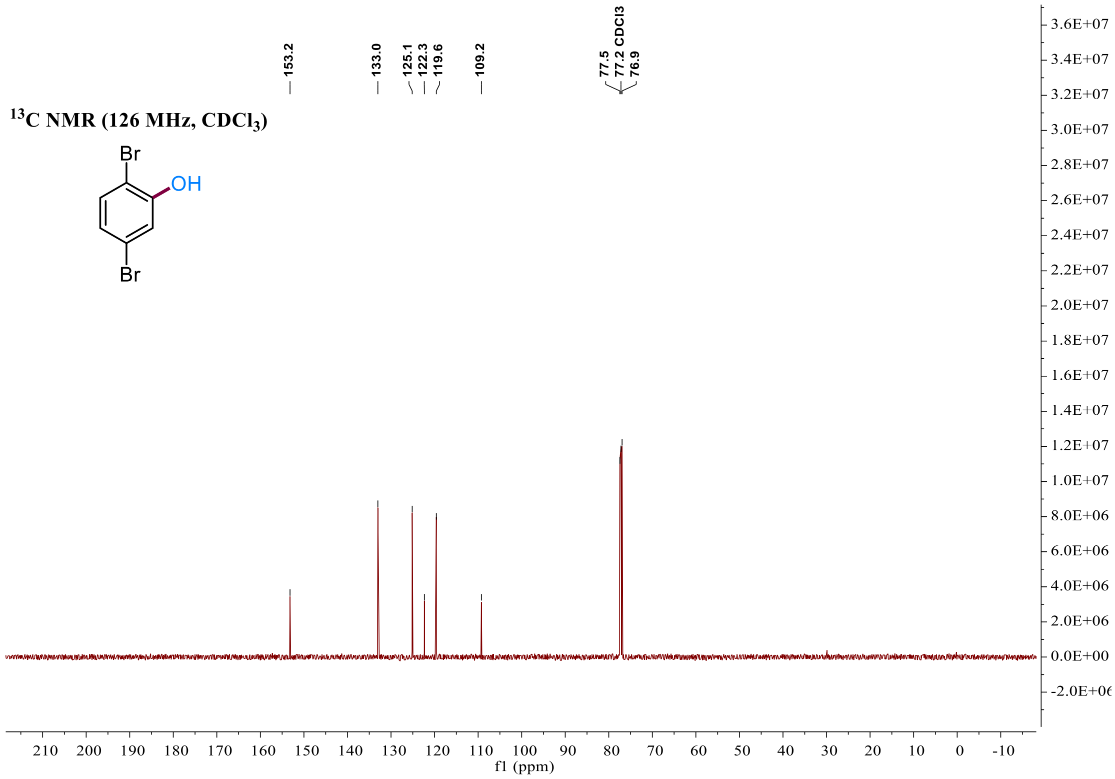

Supplementary Fig. 167. ^{13}C NMR spectra of compound **60** (126 MHz, rt, $CDCl_3$).

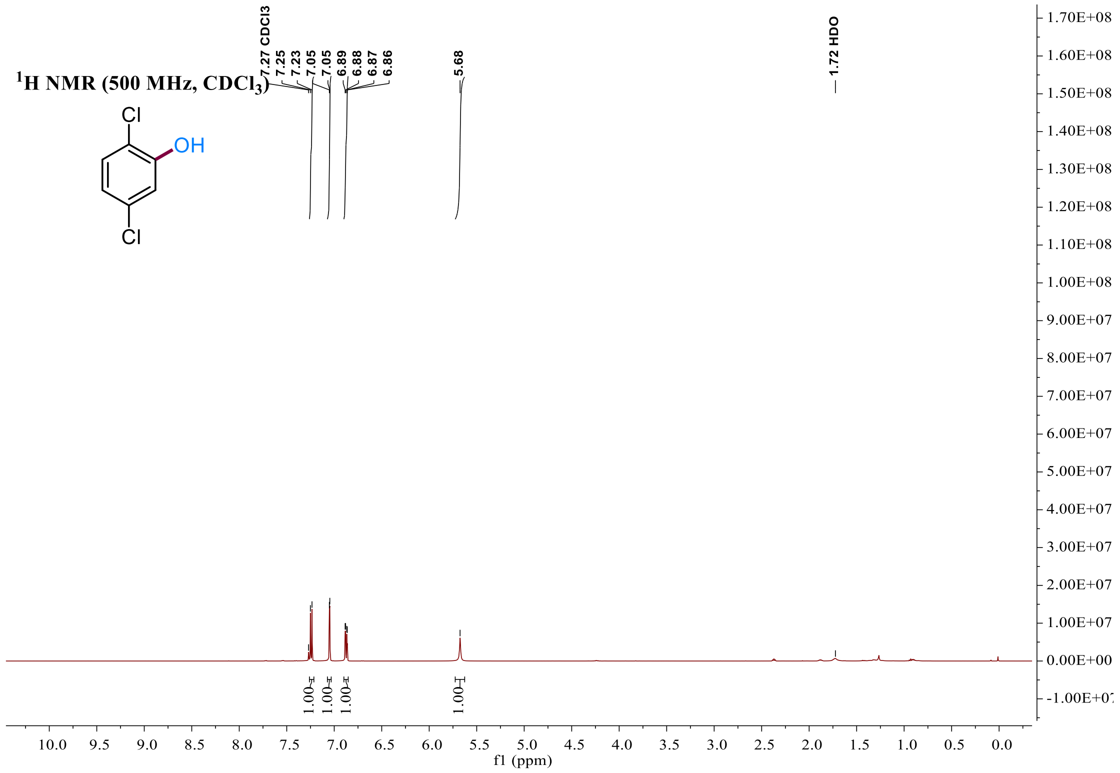

Supplementary Fig. 168. ^{1}H NMR spectra of compound **61** (500 MHz, rt, $CDCl_3$).

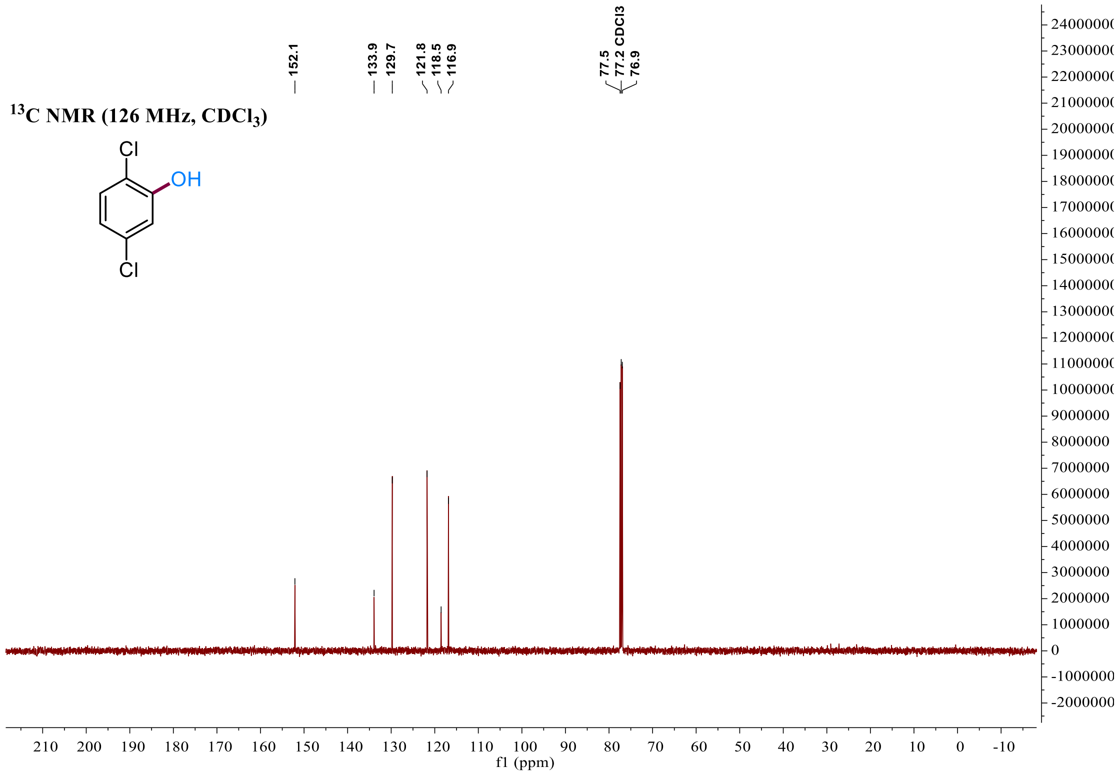

Supplementary Fig. 169. ^{13}C NMR spectra of compound **61** (126 MHz, rt, $CDCl_3$).

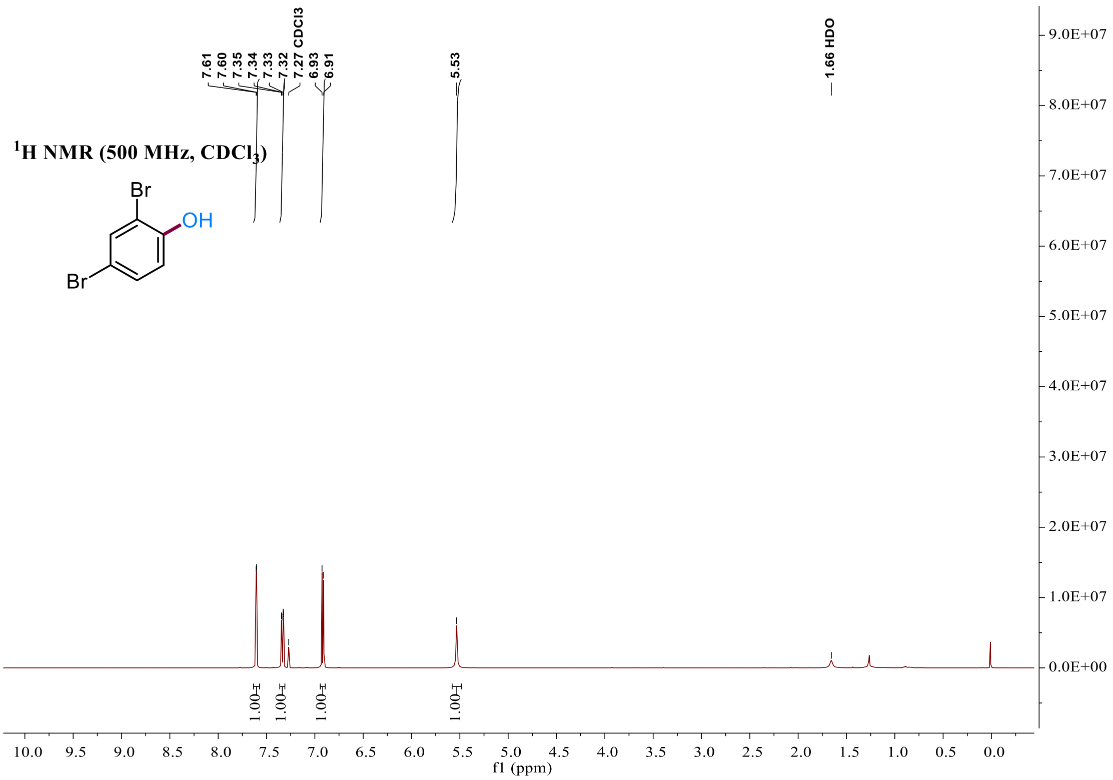

Supplementary Fig. 170. ^{1}H NMR spectra of compound **62** (500 MHz, rt, $CDCl_3$).

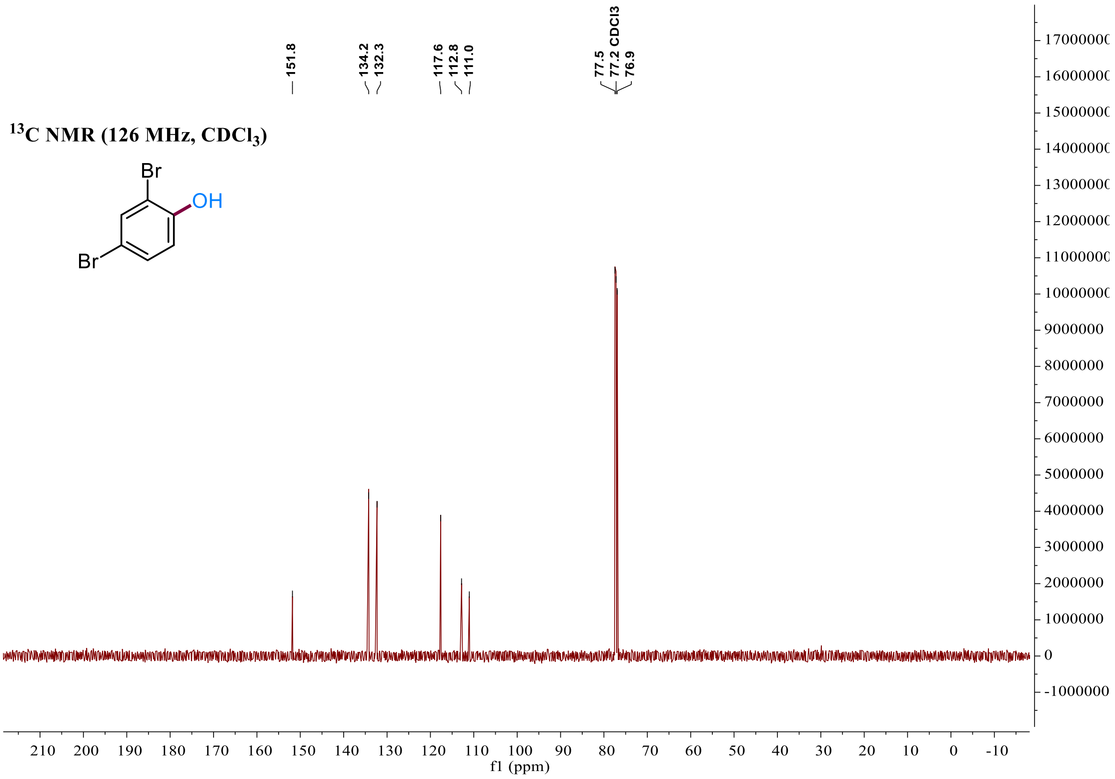

Supplementary Fig. 171. ^{13}C NMR spectra of compound **62** (126 MHz, rt, $CDCl_3$).

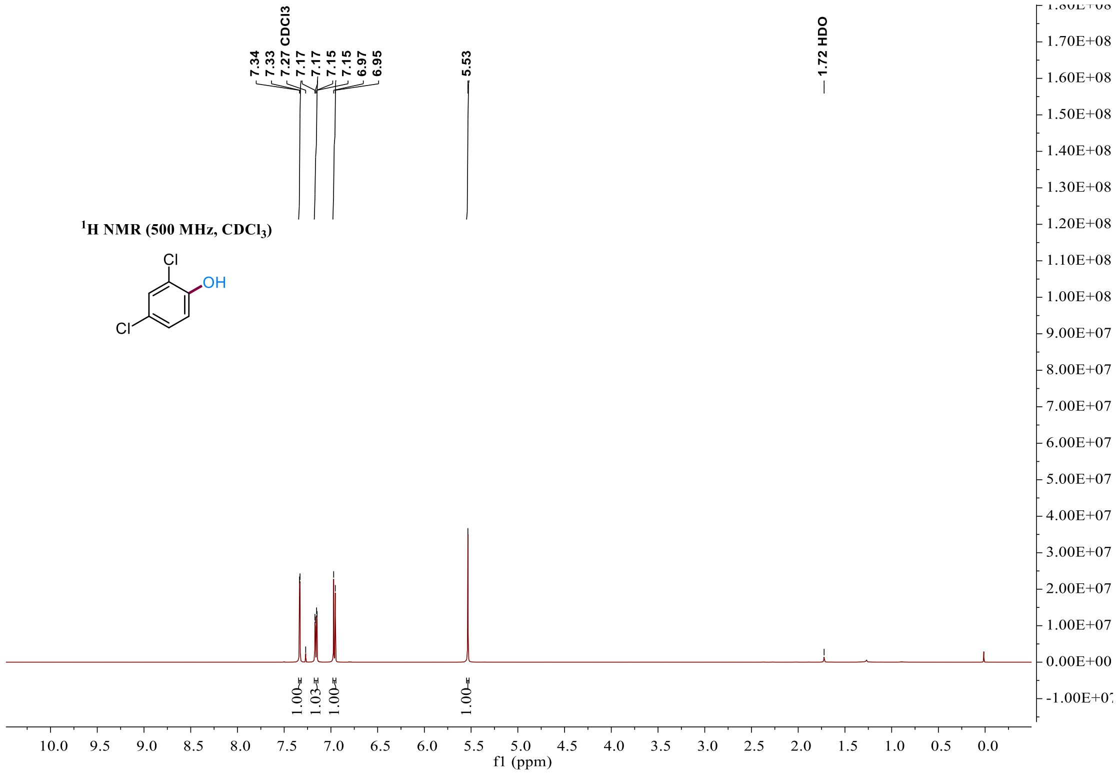

Supplementary Fig. 172. ^{1}H NMR spectra of compound **63** (500 MHz, rt, $CDCl_3$).

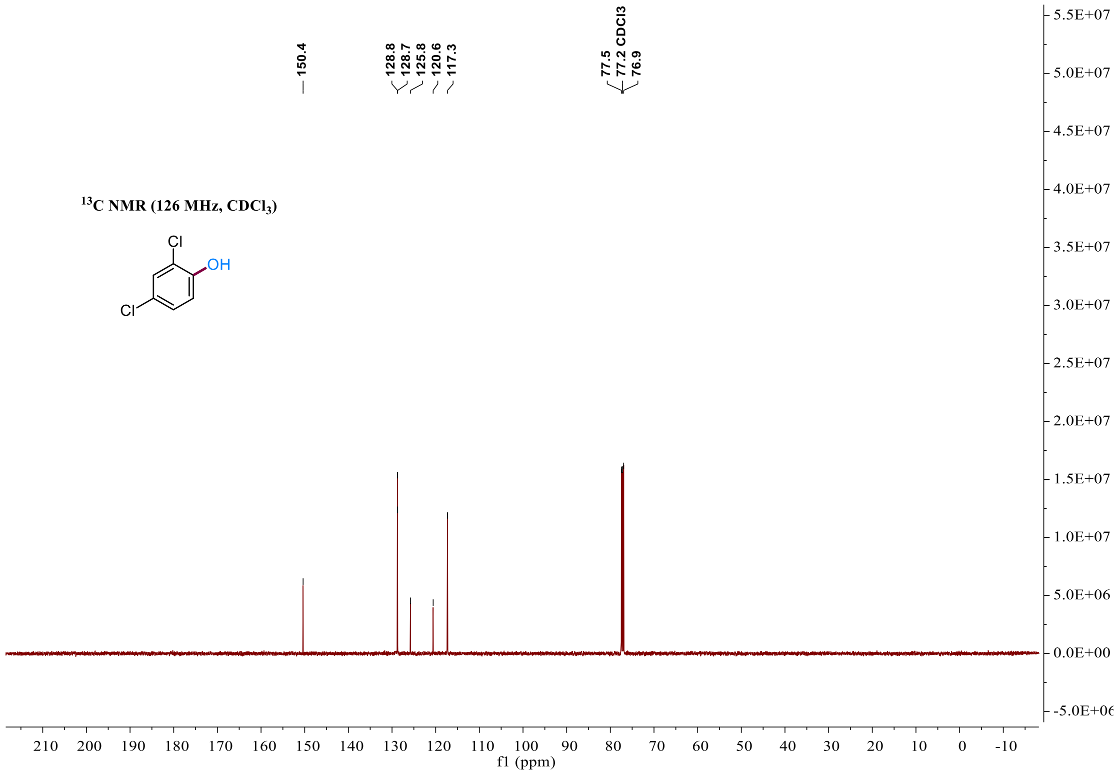

Supplementary Fig. 173. ^{13}C NMR spectra of compound **63** (126 MHz, rt, $CDCl_3$).

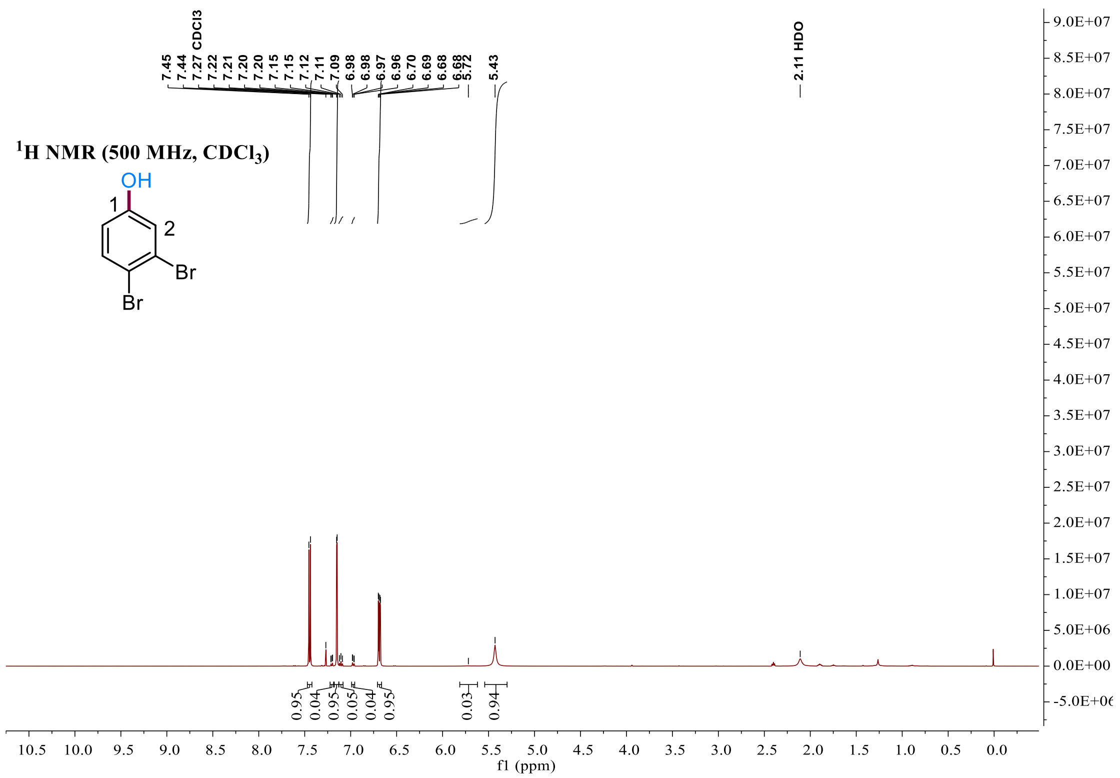

Supplementary Fig. 174. ^{1}H NMR spectra of compound **64** (500 MHz, rt, $CDCl_3$).

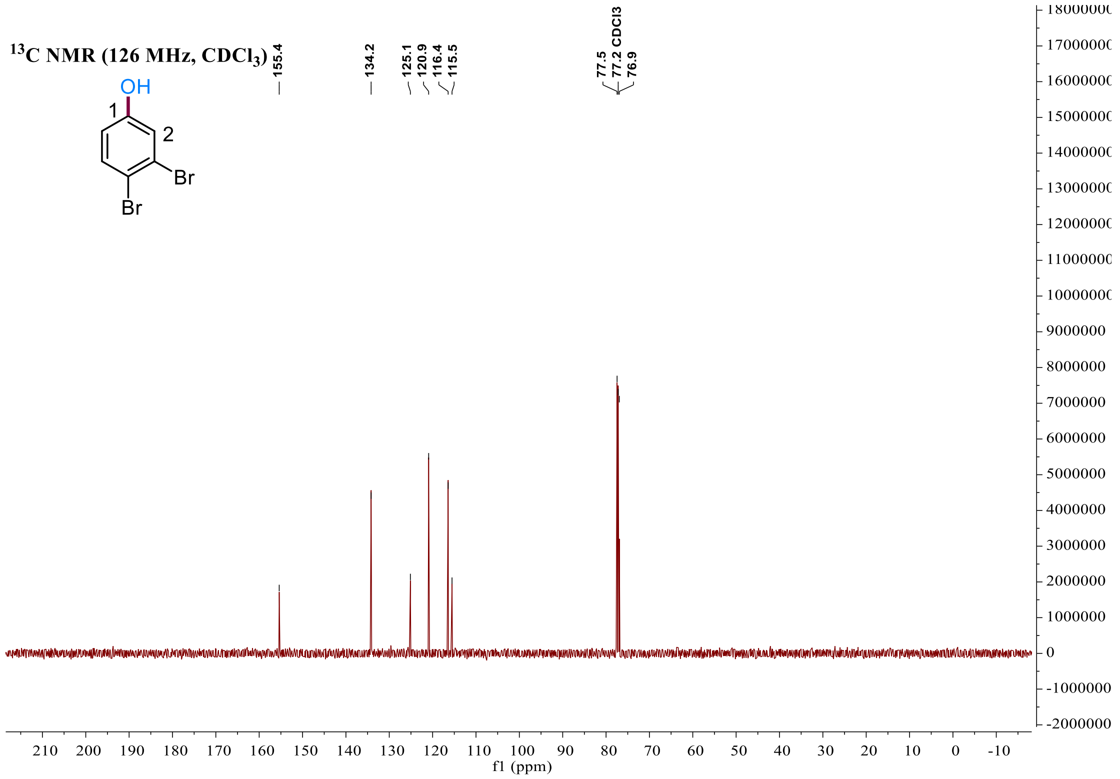

Supplementary Fig. 175. ^{13}C NMR spectra of compound **64** (126 MHz, rt, $CDCl_3$).

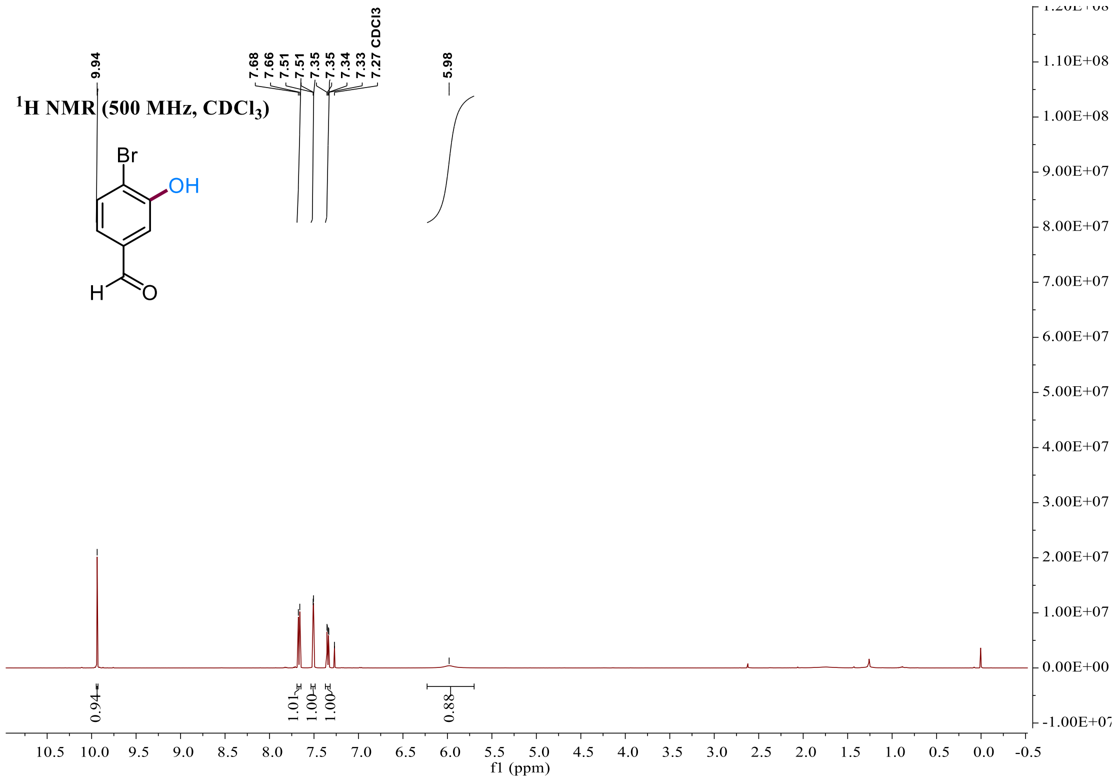

Supplementary Fig. 176. ^{1}H NMR spectra of compound **65** (500 MHz, rt, $CDCl_3$).

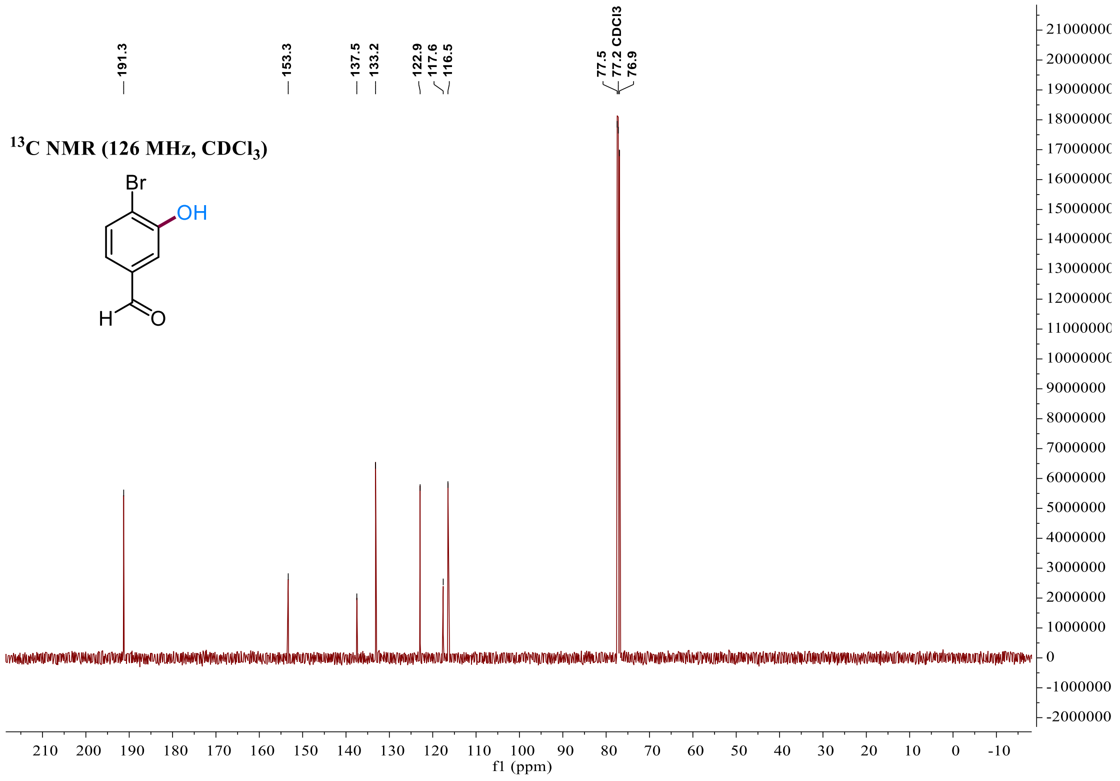

Supplementary Fig. 177. ^{13}C NMR spectra of compound **65** (126 MHz, rt, $CDCl_3$).

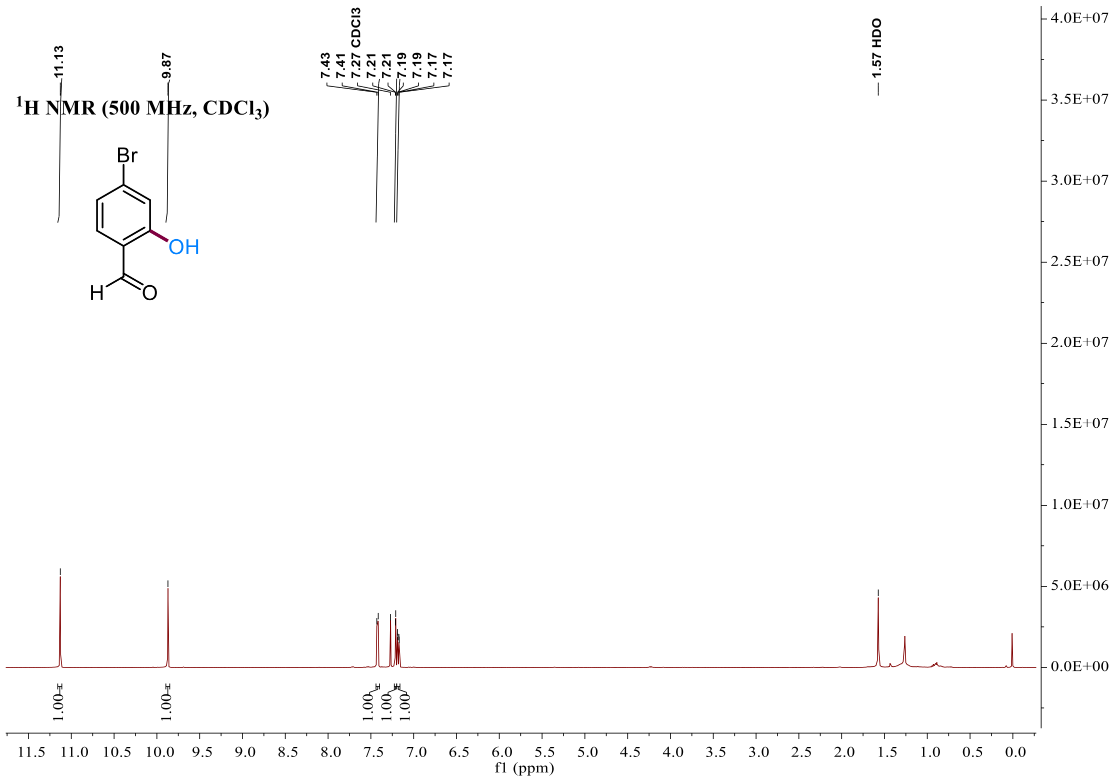

Supplementary Fig. 178. ^{1}H NMR spectra of compound **65** (500 MHz, rt, $CDCl_3$).

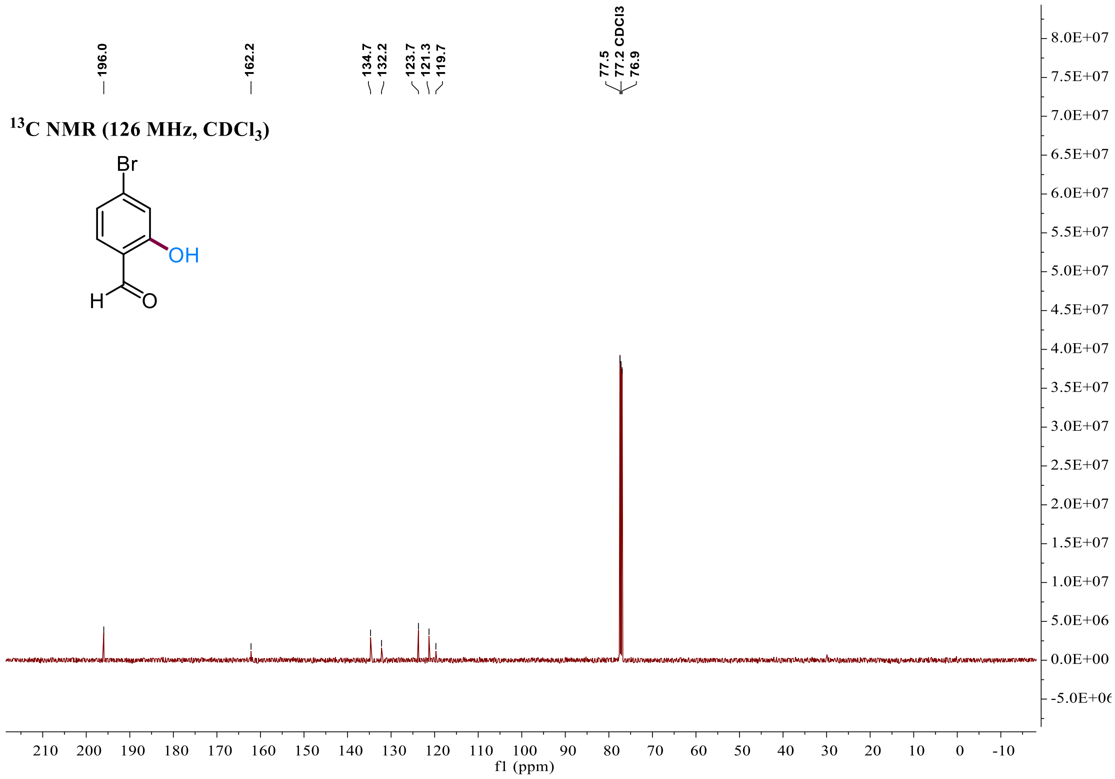

Supplementary Fig. 179. ^{13}C NMR spectra of compound **65** (126 MHz, rt, $CDCl_3$).

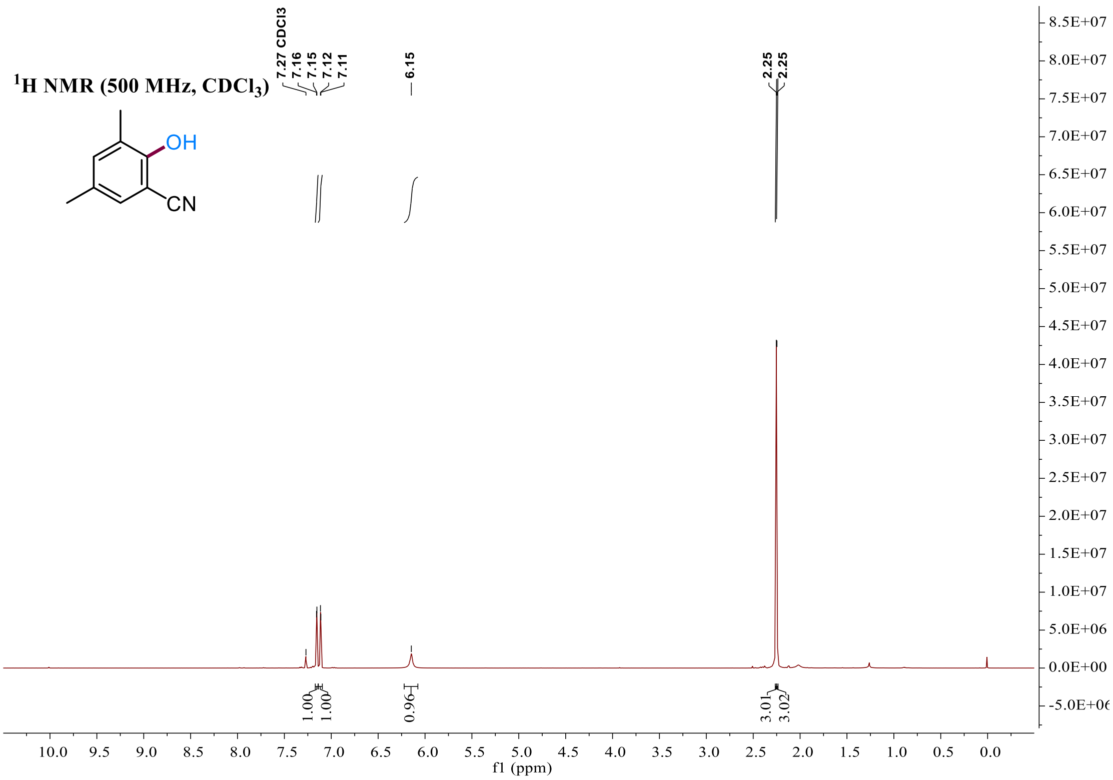

Supplementary Fig. 180. ^{1}H NMR spectra of compound **66** (500 MHz, rt, $CDCl_3$).

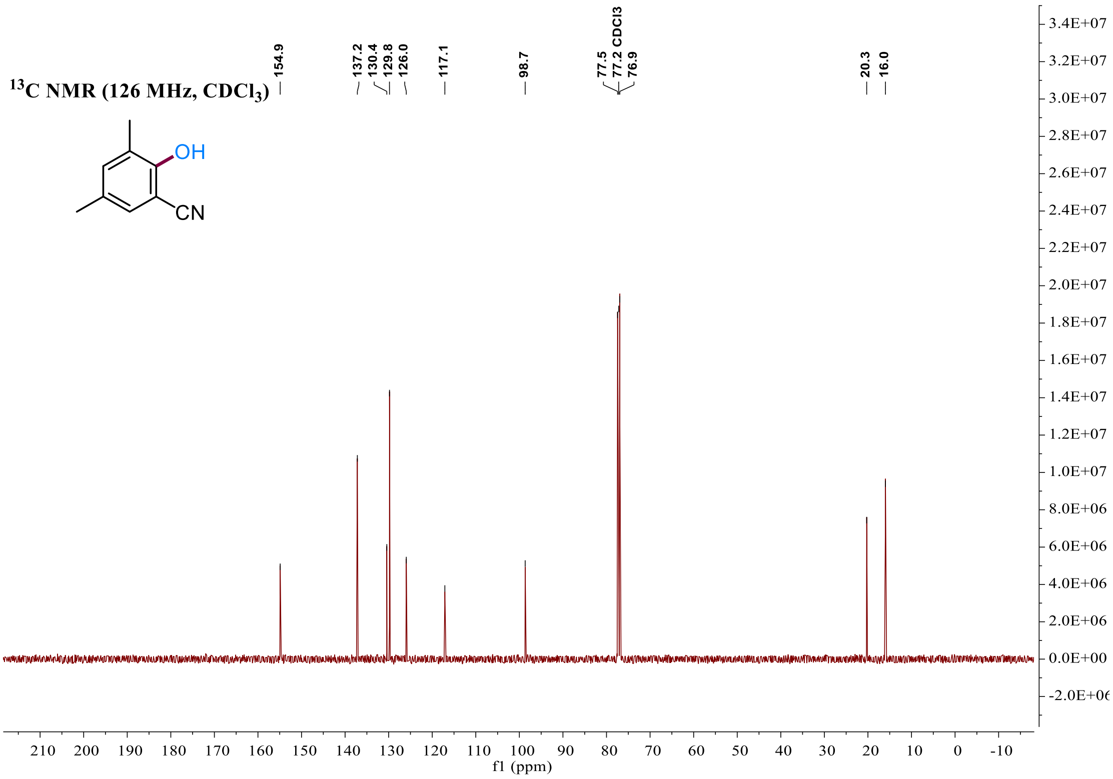

Supplementary Fig. 181. ^{13}C NMR spectra of compound **66** (126 MHz, rt, $CDCl_3$).

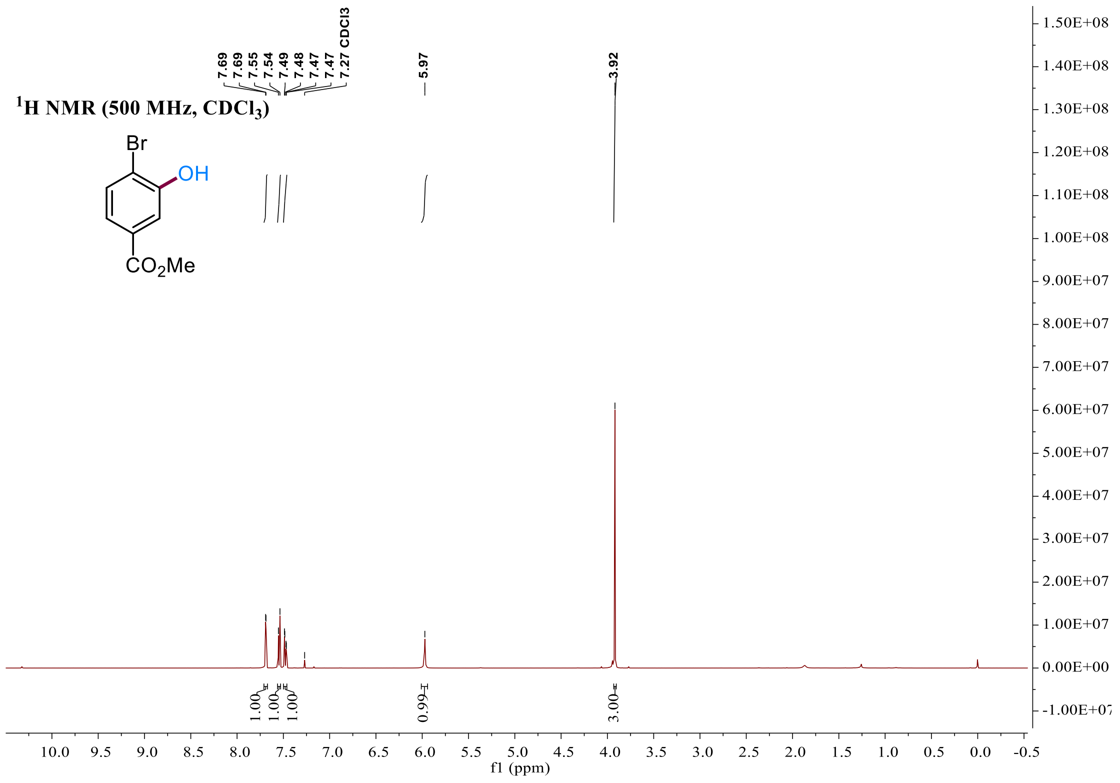

Supplementary Fig. 182. ^{1}H NMR spectra of compound **67** (500 MHz, rt, $CDCl_3$).

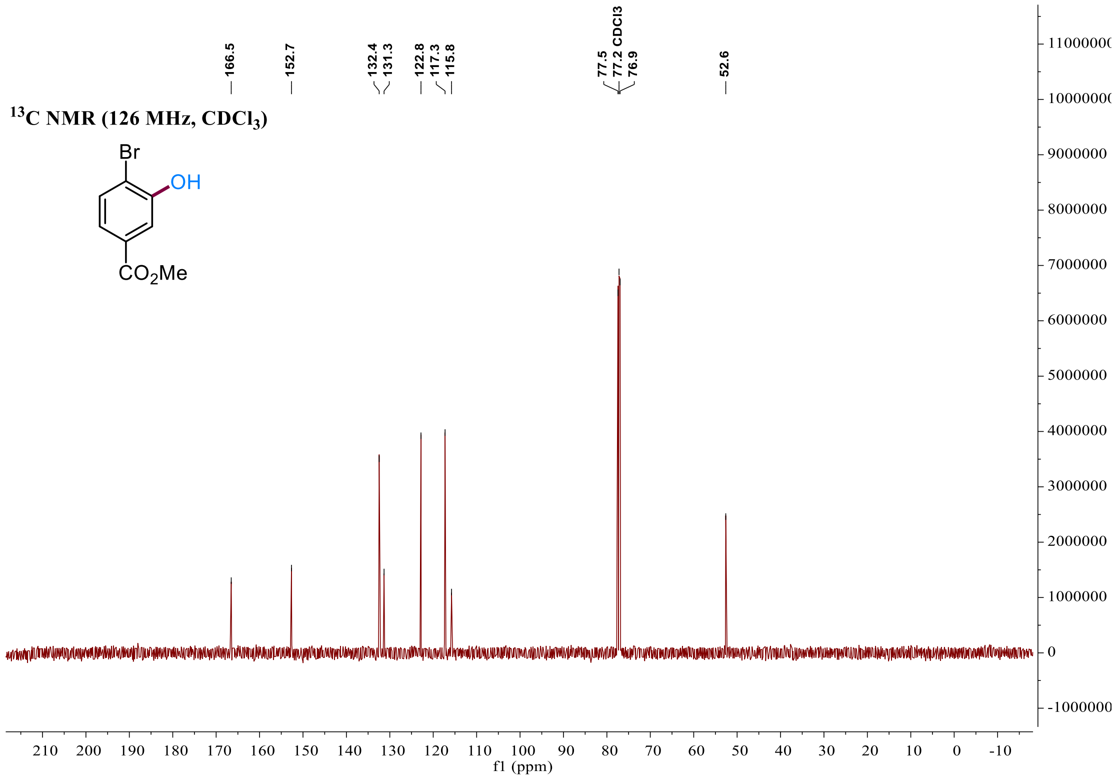

Supplementary Fig. 183. ^{13}C NMR spectra of compound **67** (126 MHz, rt, $CDCl_3$).

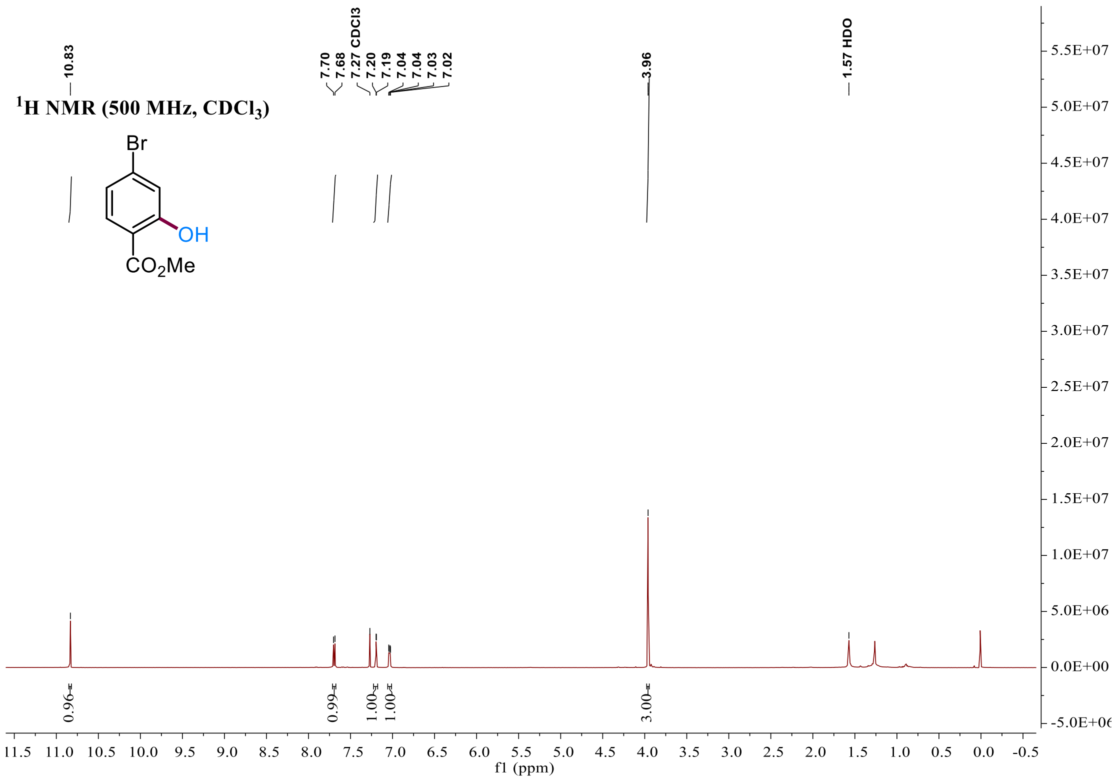

Supplementary Fig. 184. ^{1}H NMR spectra of compound **67** (500 MHz, rt, $CDCl_3$).

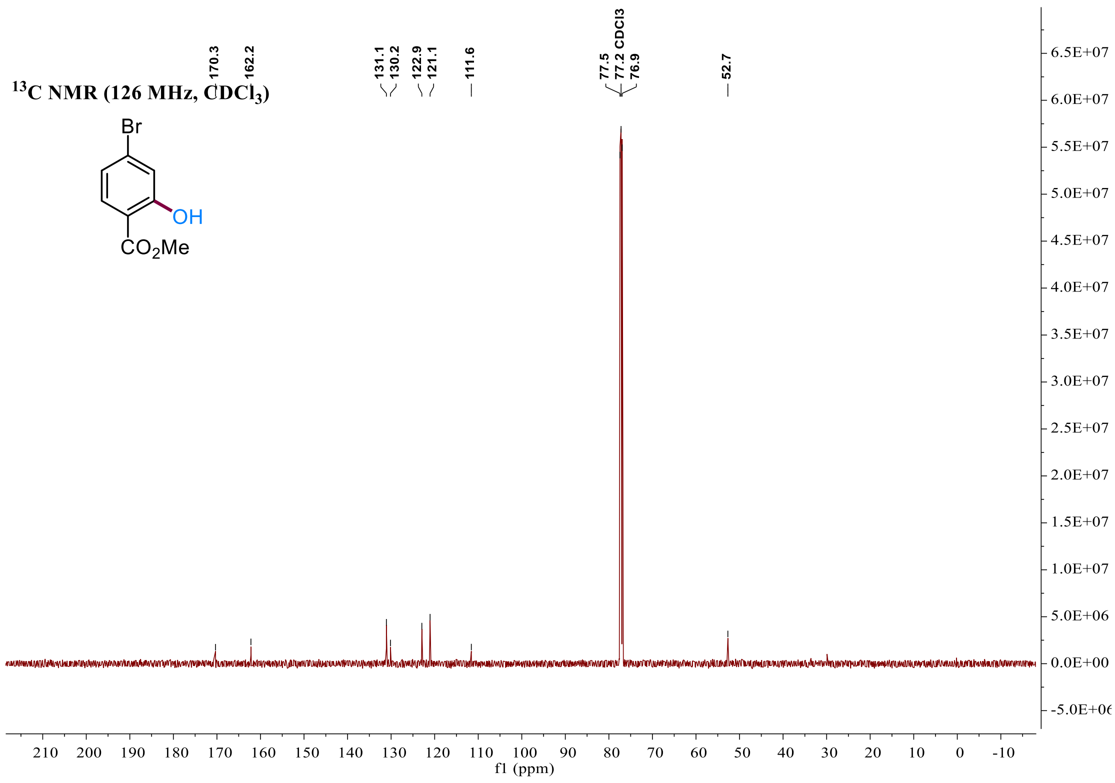

Supplementary Fig. 185. ^{13}C NMR spectra of compound **67** (126 MHz, rt, $CDCl_3$).

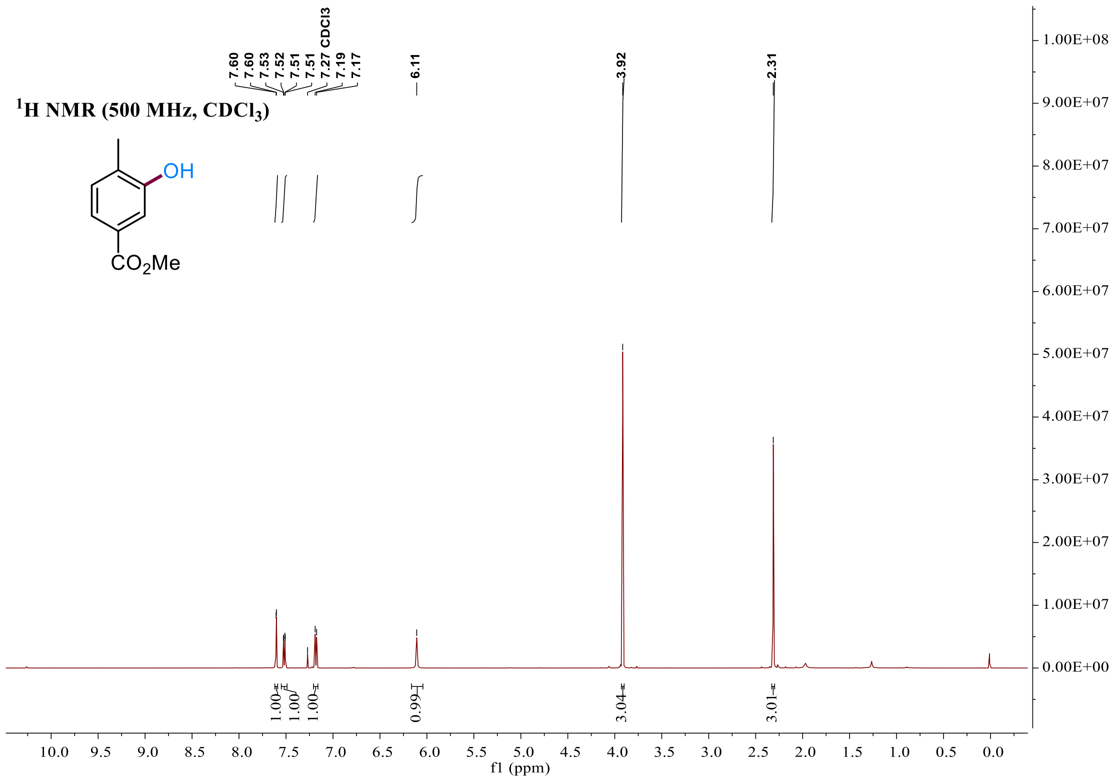

Supplementary Fig. 186. ^{1}H NMR spectra of compound **68** (500 MHz, rt, $CDCl_3$).

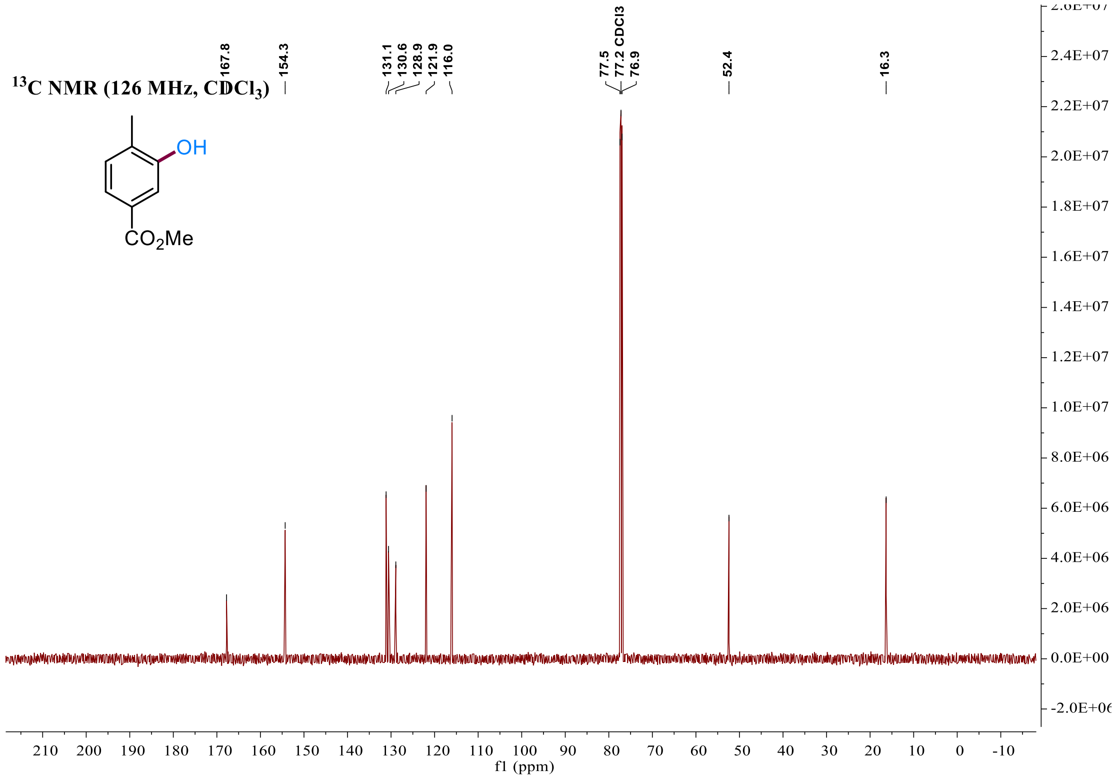

Supplementary Fig. 187. ^{13}C NMR spectra of compound **68** (126 MHz, rt, $CDCl_3$).

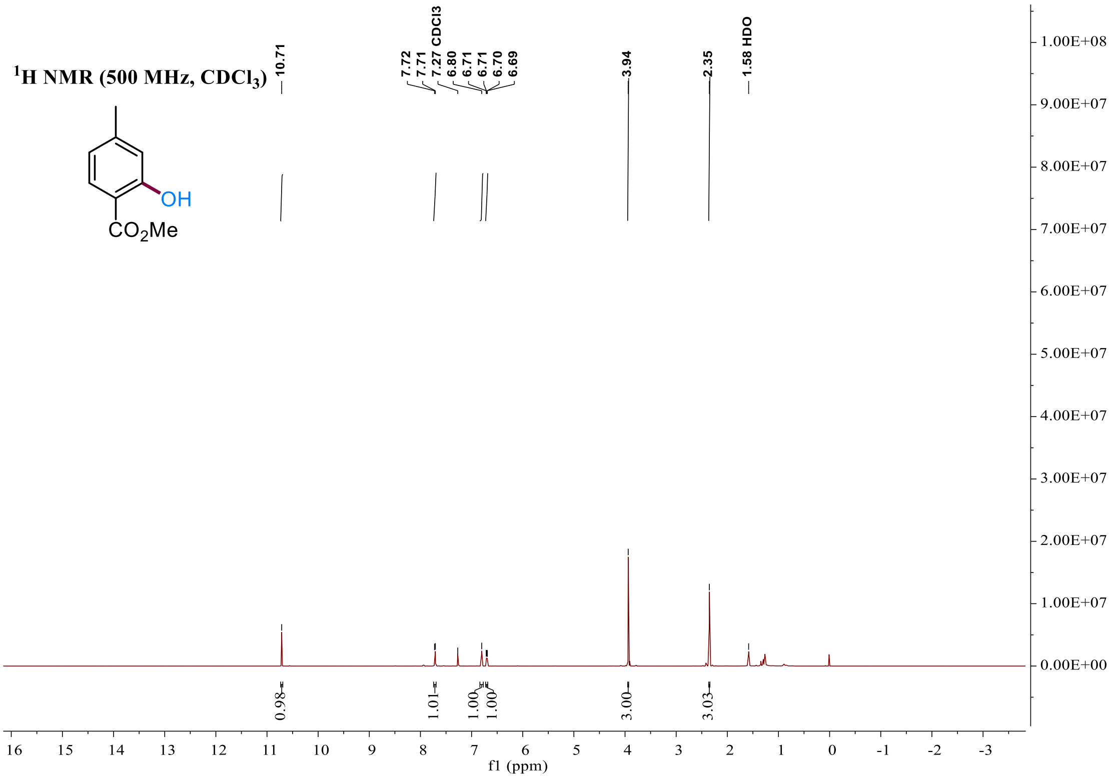

Supplementary Fig. 188. ^{1}H NMR spectra of compound **68** (500 MHz, rt, $CDCl_3$).

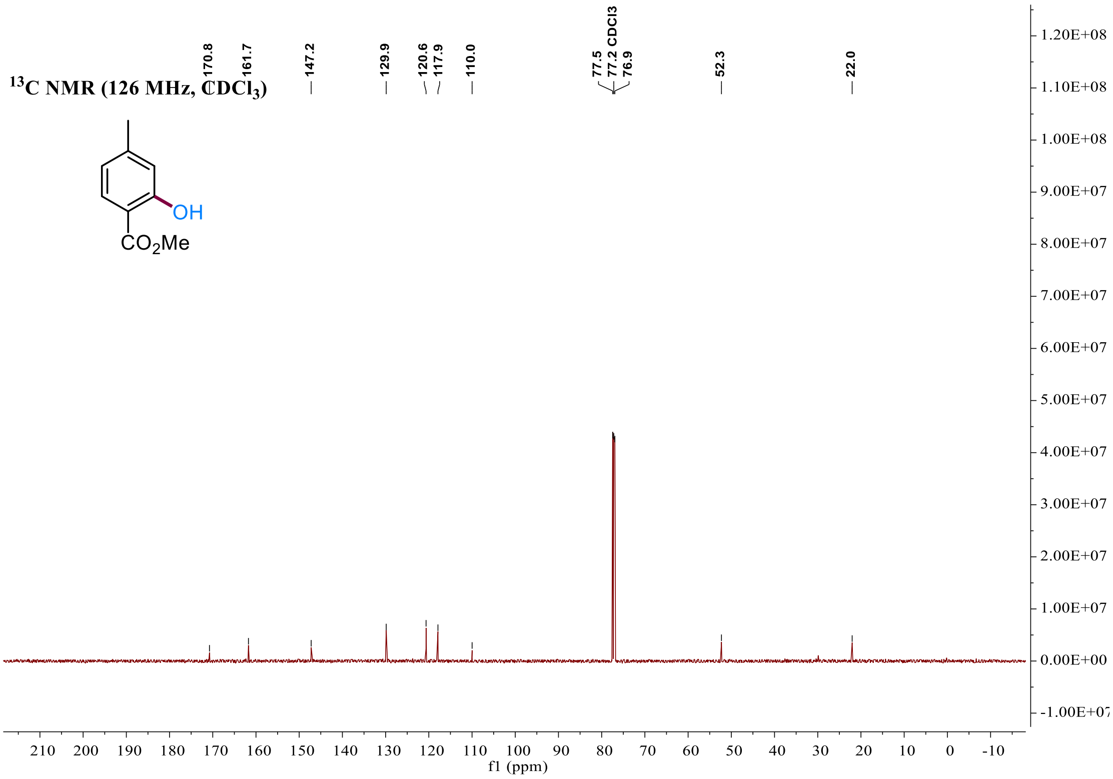

Supplementary Fig. 189. ^{13}C NMR spectra of compound **68** (126 MHz, rt, $CDCl_3$).

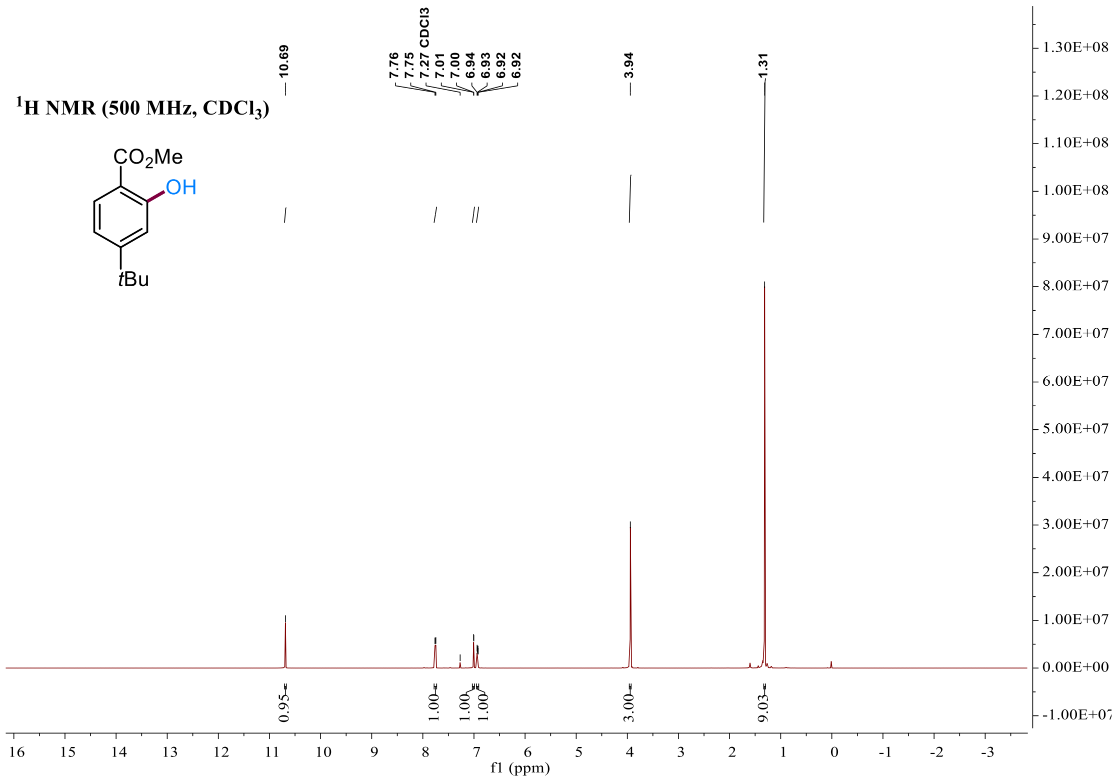

Supplementary Fig. 190. ^{1}H NMR spectra of compound **69** (500 MHz, rt, $CDCl_3$).

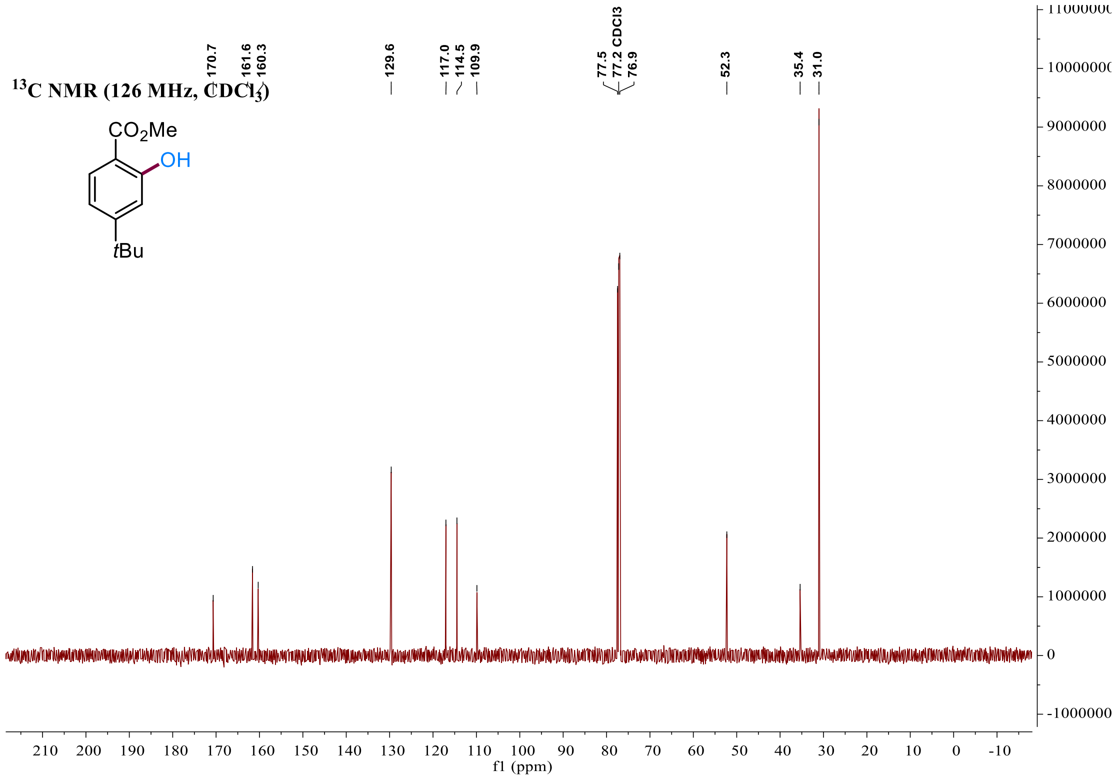

Supplementary Fig. 191. ^{13}C NMR spectra of compound **69** (126 MHz, rt, $CDCl_3$).

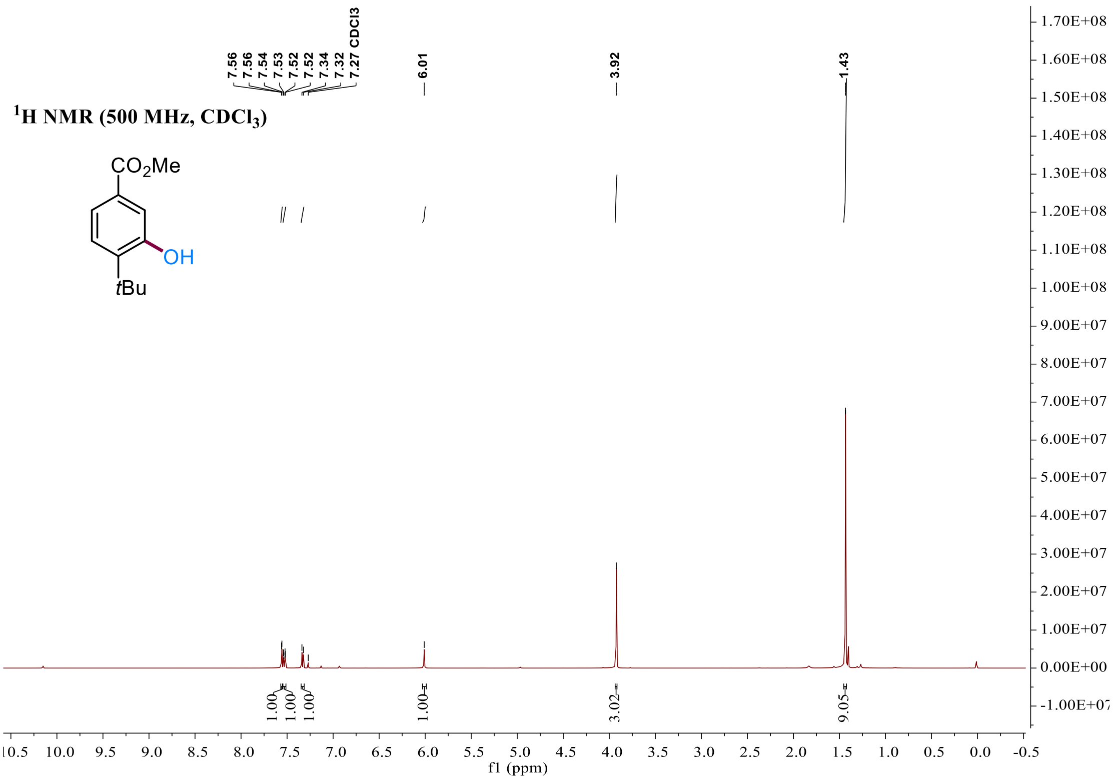

Supplementary Fig. 192. ^{1}H NMR spectra of compound **69** (500 MHz, rt, $CDCl_3$).

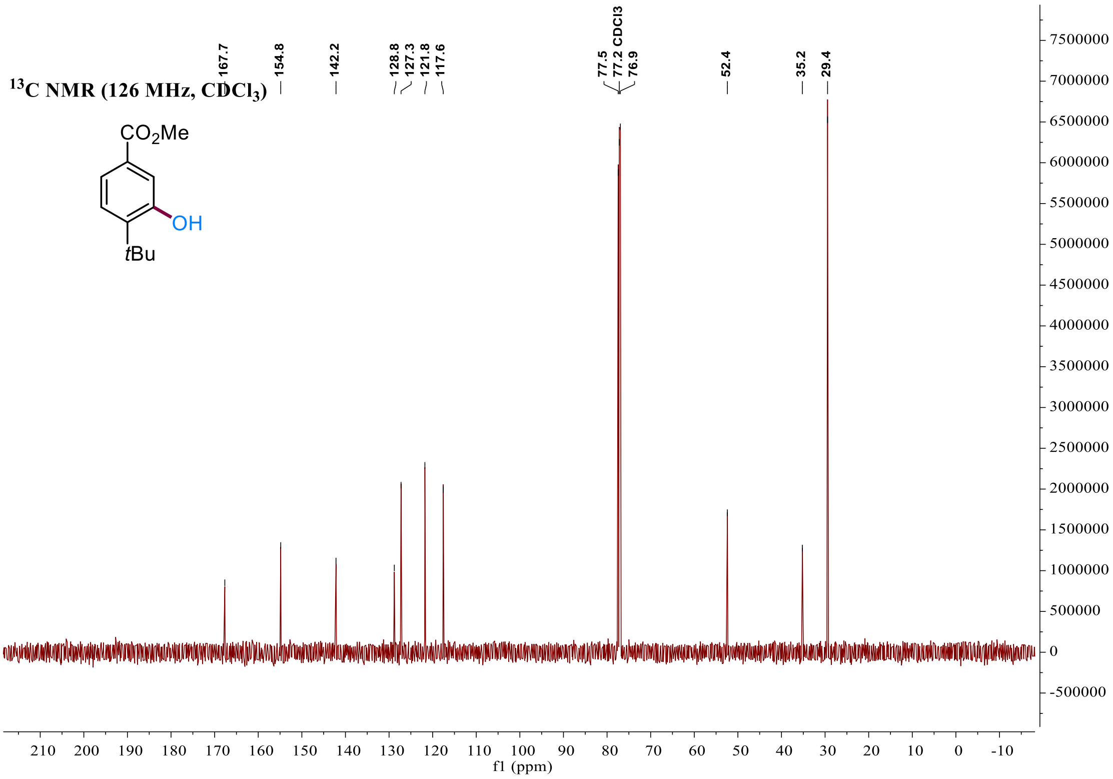

Supplementary Fig. 193. ^{13}C NMR spectra of compound **69** (126 MHz, rt, $CDCl_3$).

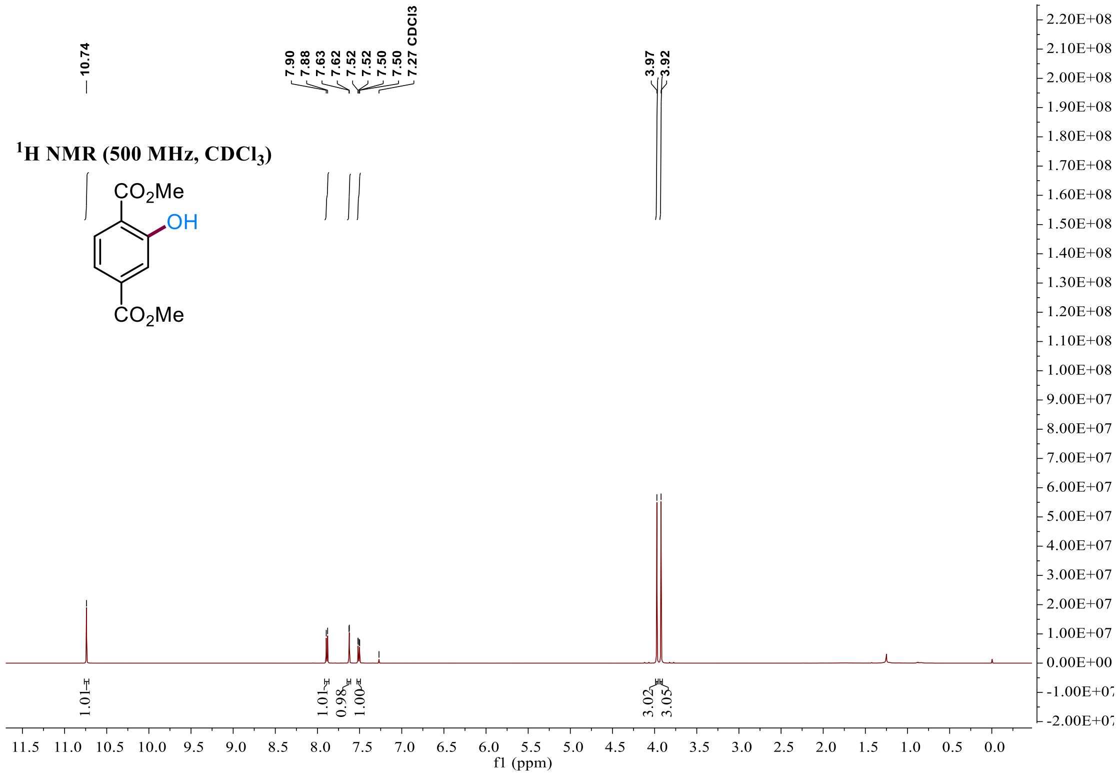

Supplementary Fig. 194. ^{1}H NMR spectra of compound **70** (500 MHz, rt, $CDCl_3$).

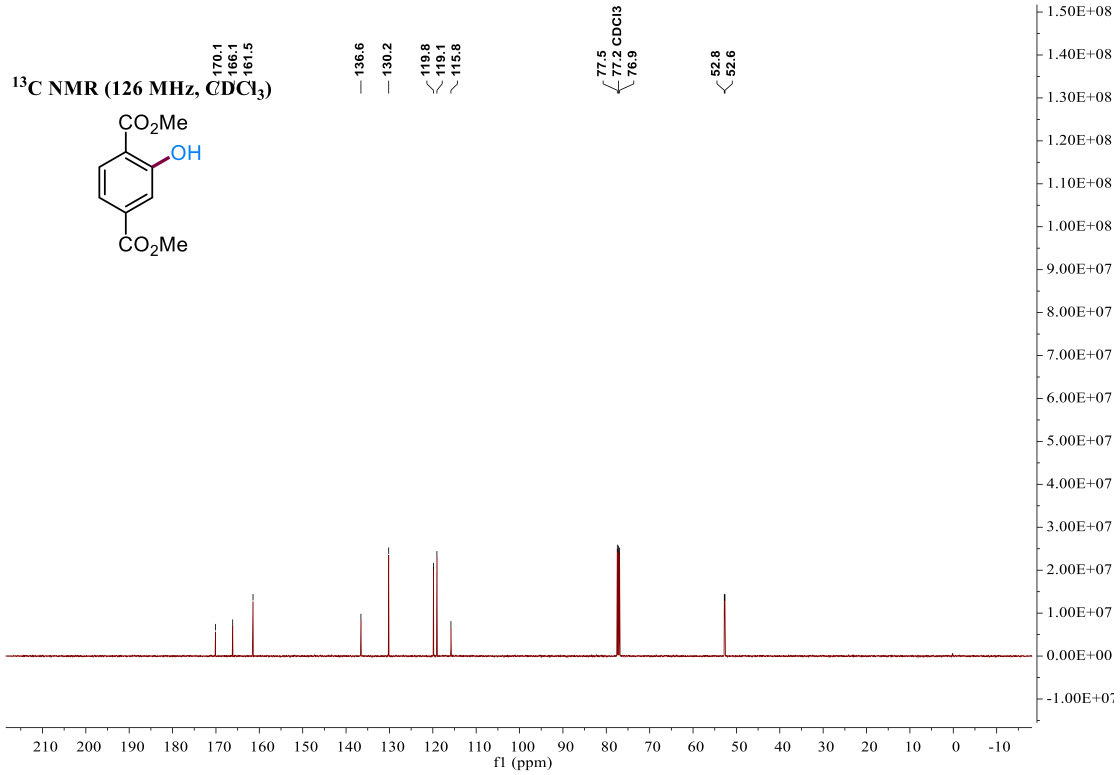

Supplementary Fig. 195. ^{13}C NMR spectra of compound **70** (126 MHz, rt, $CDCl_3$).

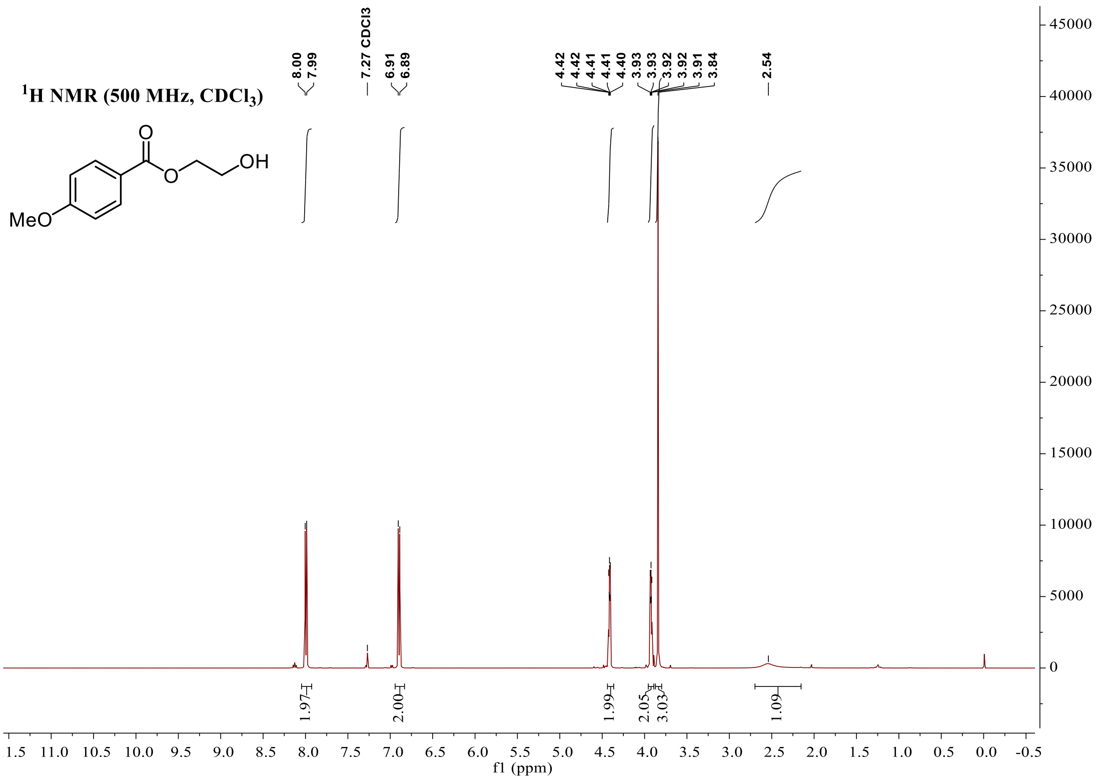

Supplementary Fig. 196. ^{1}H NMR spectra of compound **S1** (500 MHz, rt, $CDCl_3$).

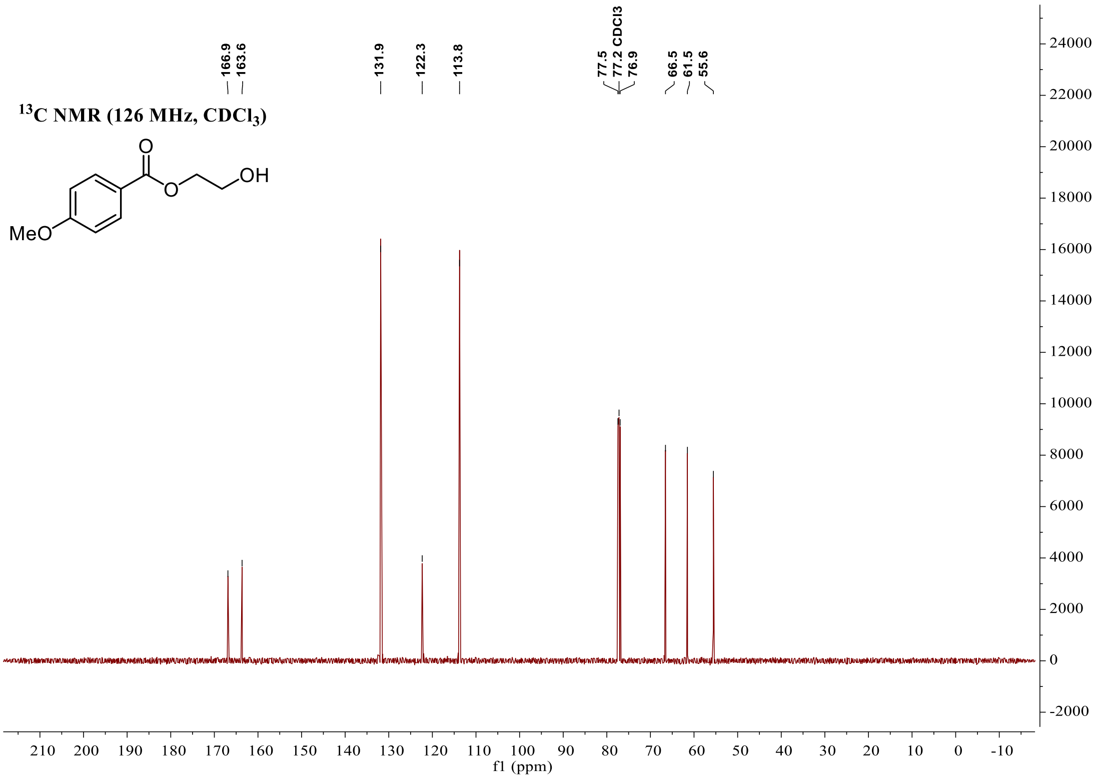

Supplementary Fig. 197. ^{13}C NMR spectra of compound **S1** (126 MHz, rt, $CDCl_3$).

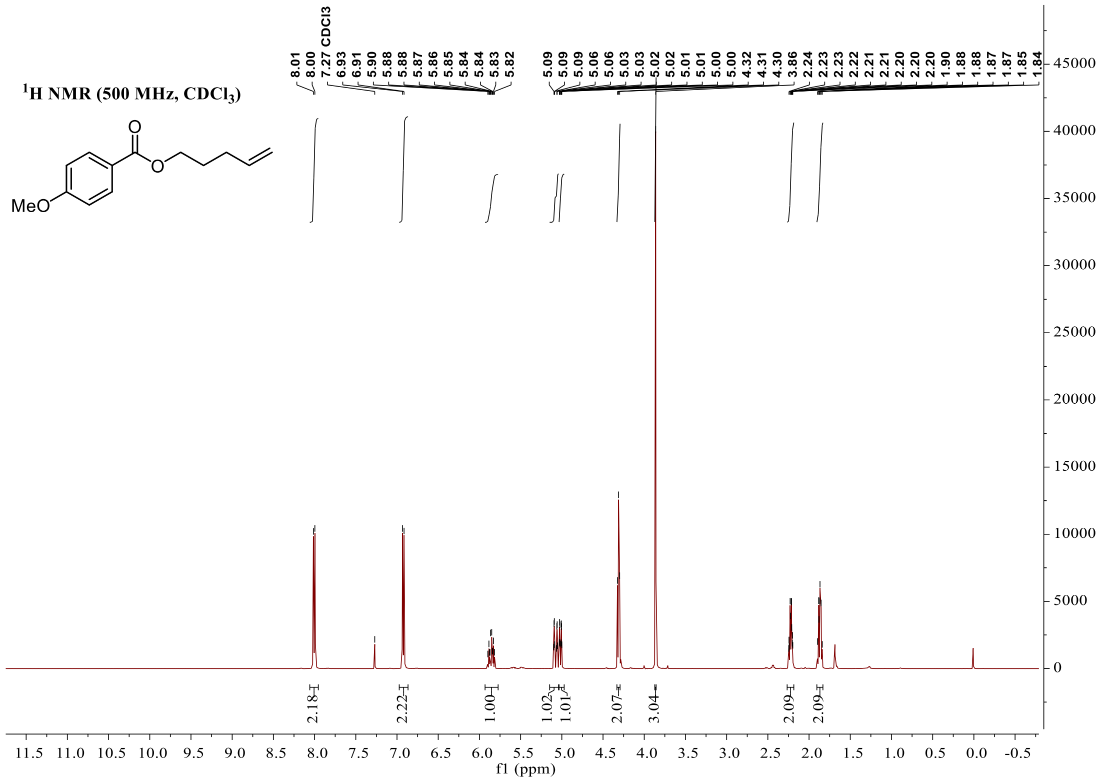

Supplementary Fig. 198. ^{1}H NMR spectra of compound **S2** (500 MHz, rt, $CDCl_3$).

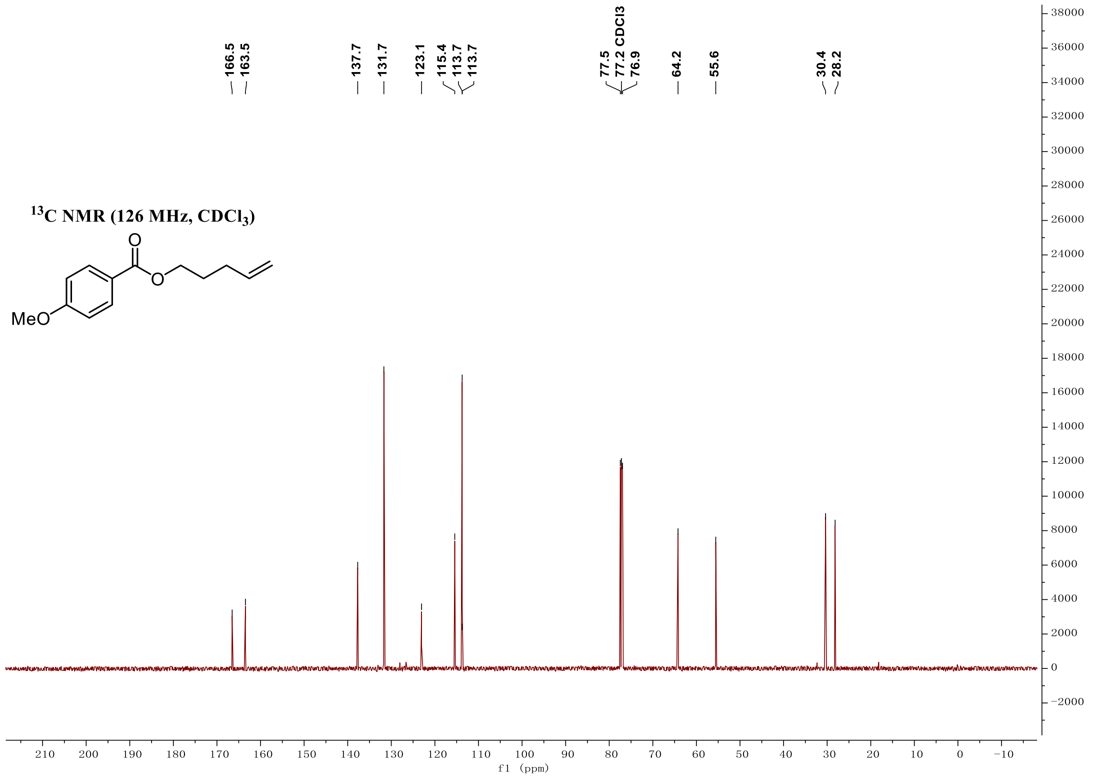

Supplementary Fig. 199. ^{13}C NMR spectra of compound **S2** (126 MHz, rt, $CDCl_3$).

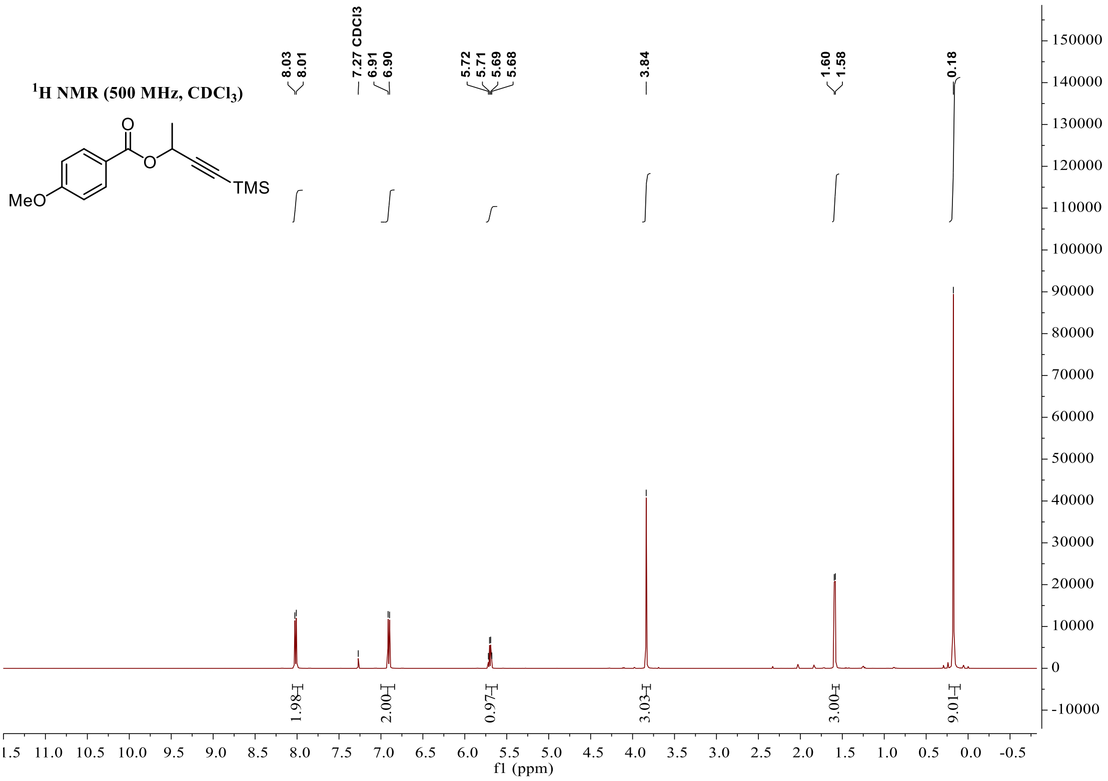

Supplementary Fig. 200. ^{1}H NMR spectra of compound **S3** (500 MHz, rt, $CDCl_3$).

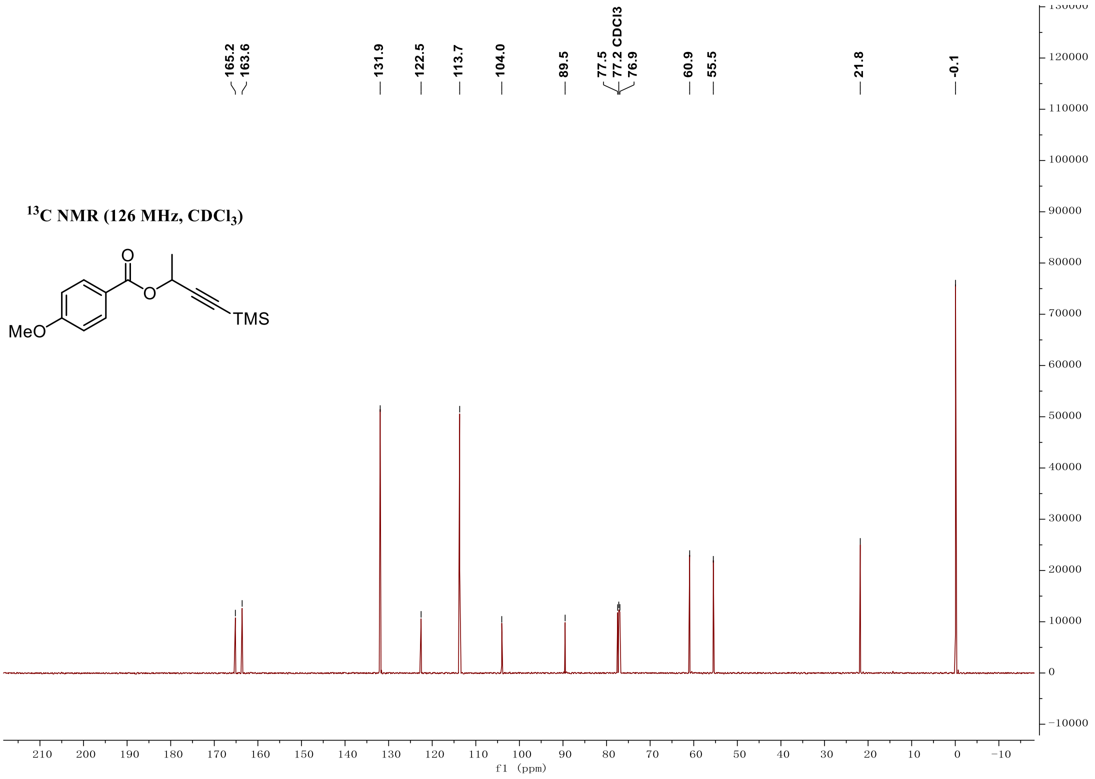

Supplementary Fig. 201. ^{13}C NMR spectra of compound **S3** (126 MHz, rt, $CDCl_3$).

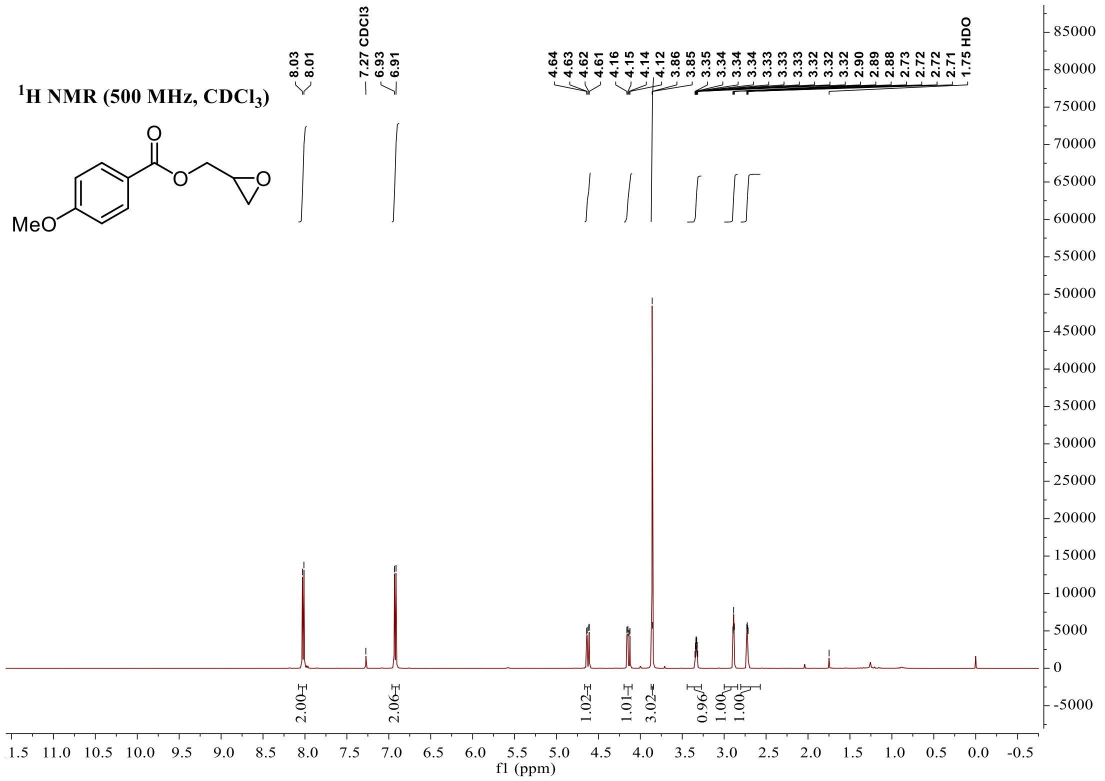

Supplementary Fig. 202. ^{1}H NMR spectra of compound **S4** (500 MHz, rt, $CDCl_3$).

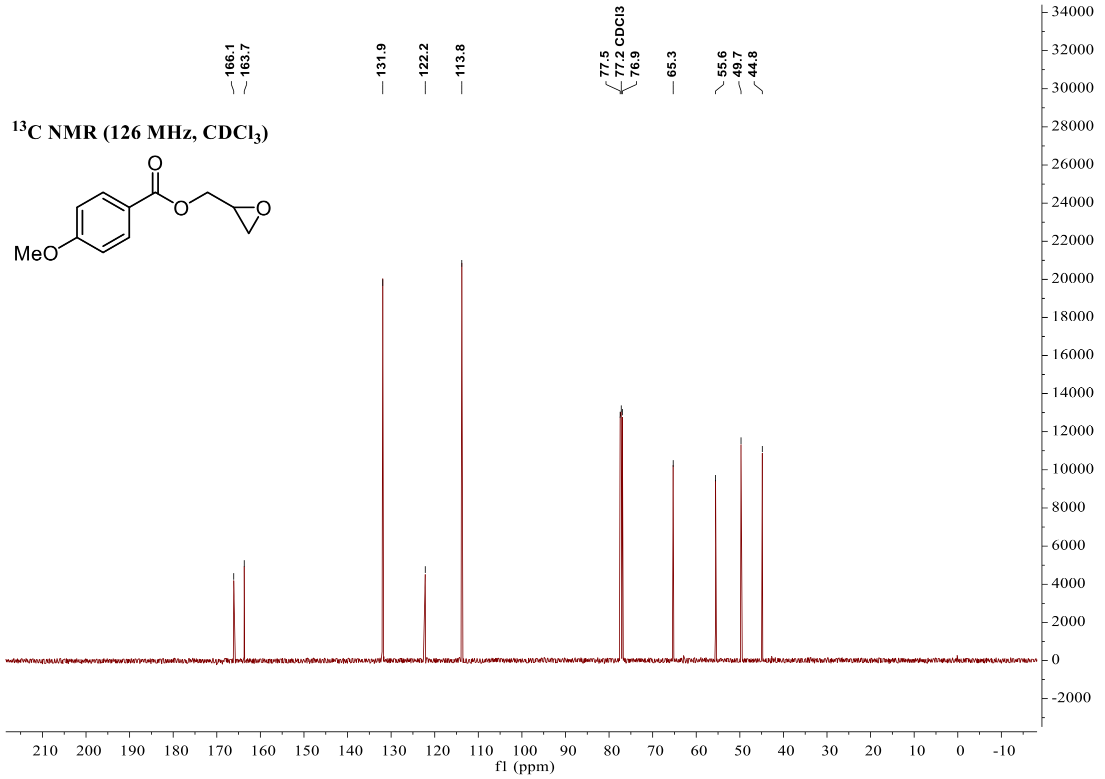

Supplementary Fig. 203. ^{13}C NMR spectra of compound **S4** (126 MHz, rt, $CDCl_3$).

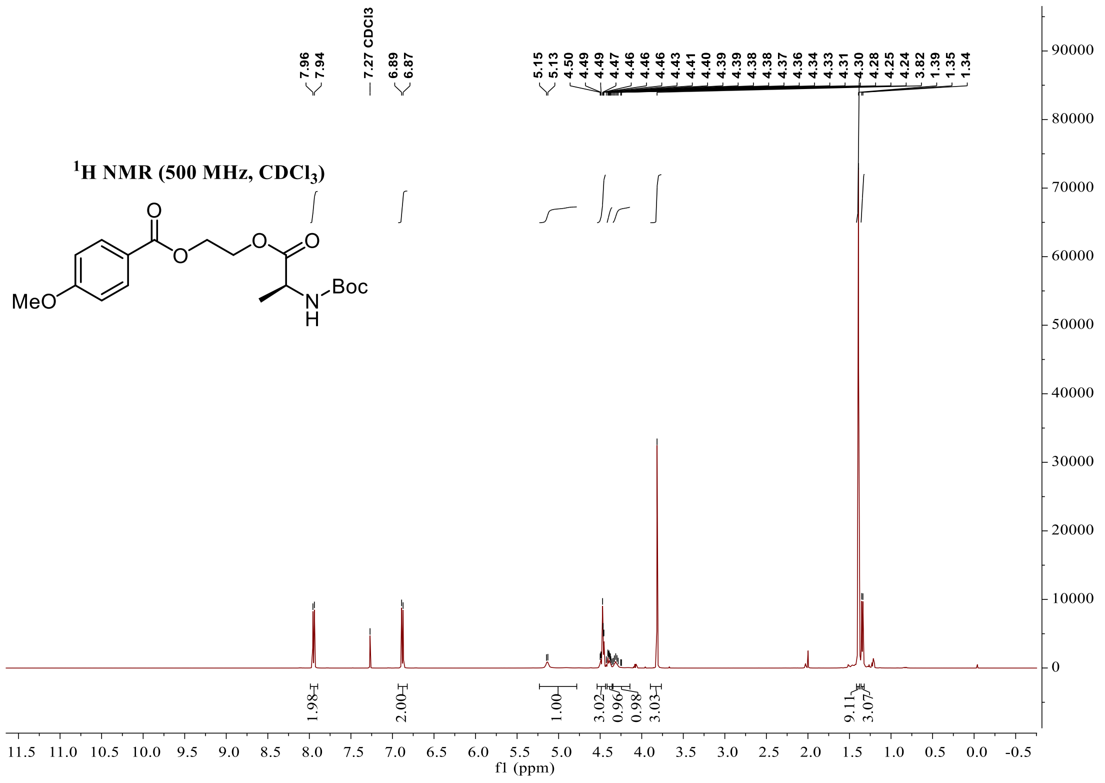

Supplementary Fig. 204. ^{1}H NMR spectra of compound **S5** (500 MHz, rt, $CDCl_3$).

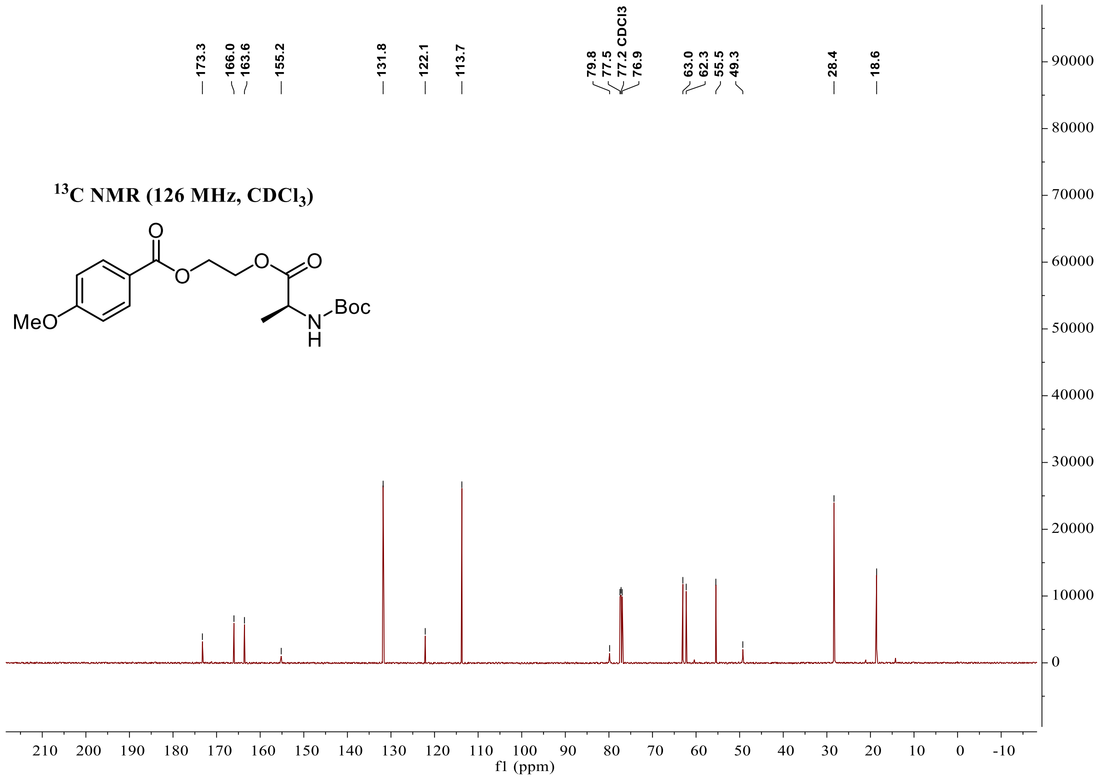

Supplementary Fig. 205. ^{13}C NMR spectra of compound **S5** (126 MHz, rt, $CDCl_3$).

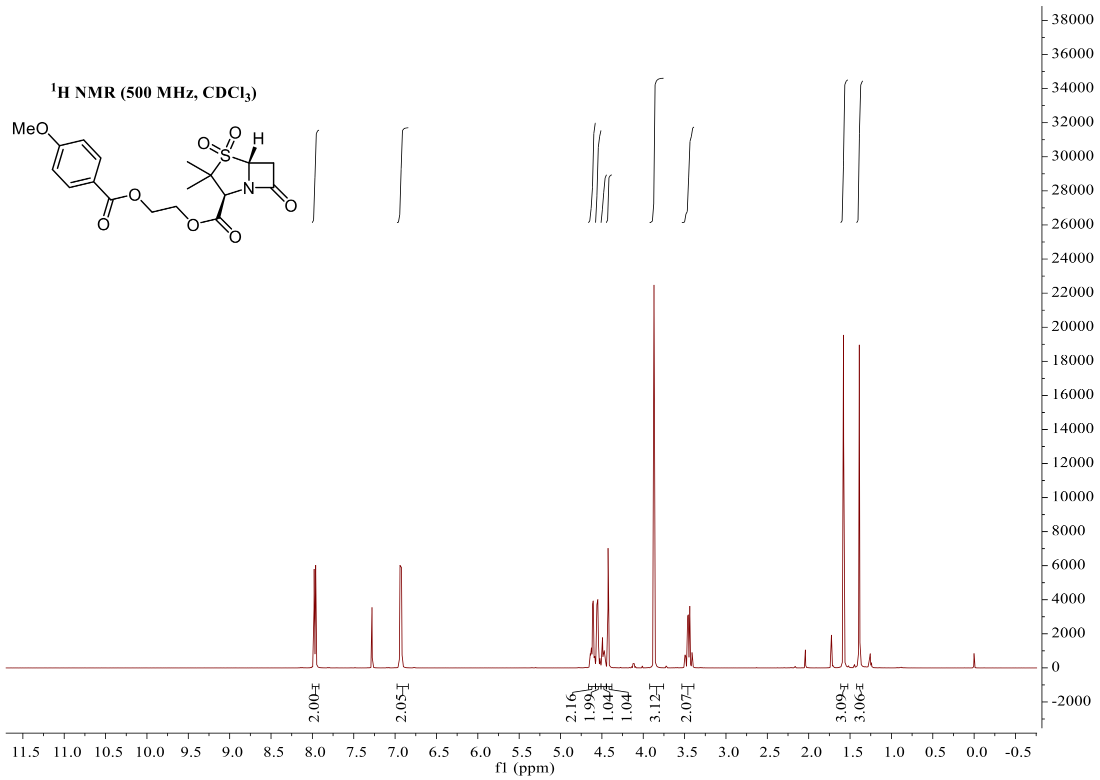

Supplementary Fig. 206. ^{1}H NMR spectra of compound **S6** (500 MHz, rt, $CDCl_3$).

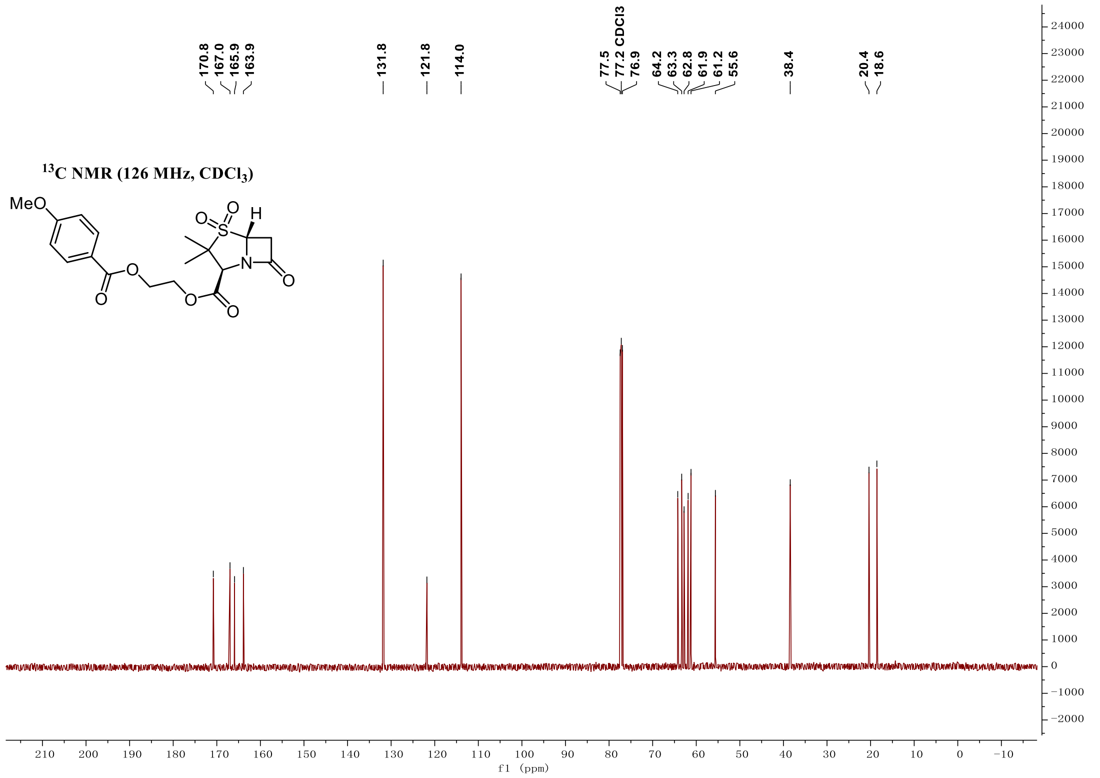

Supplementary Fig. 207. ^{13}C NMR spectra of compound **S6** (126 MHz, rt, $CDCl_3$).

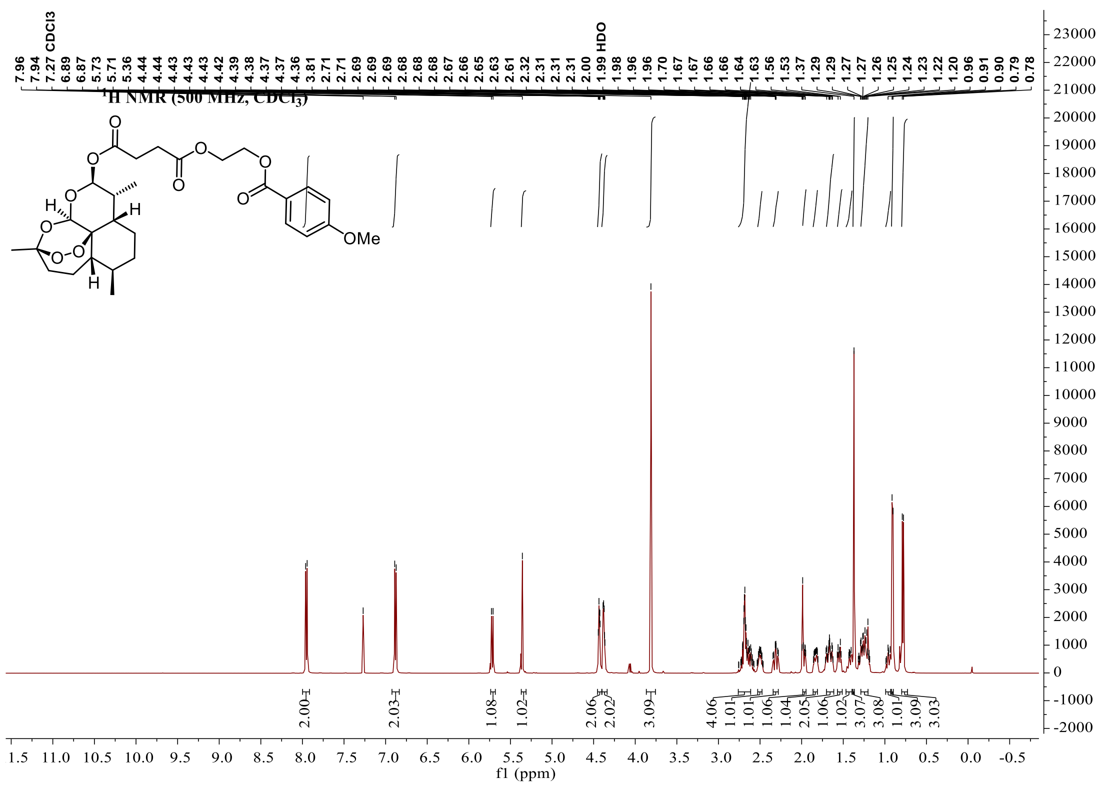

Supplementary Fig. 208. ^{1}H NMR spectra of compound **S7** (500 MHz, rt, $CDCl_3$).

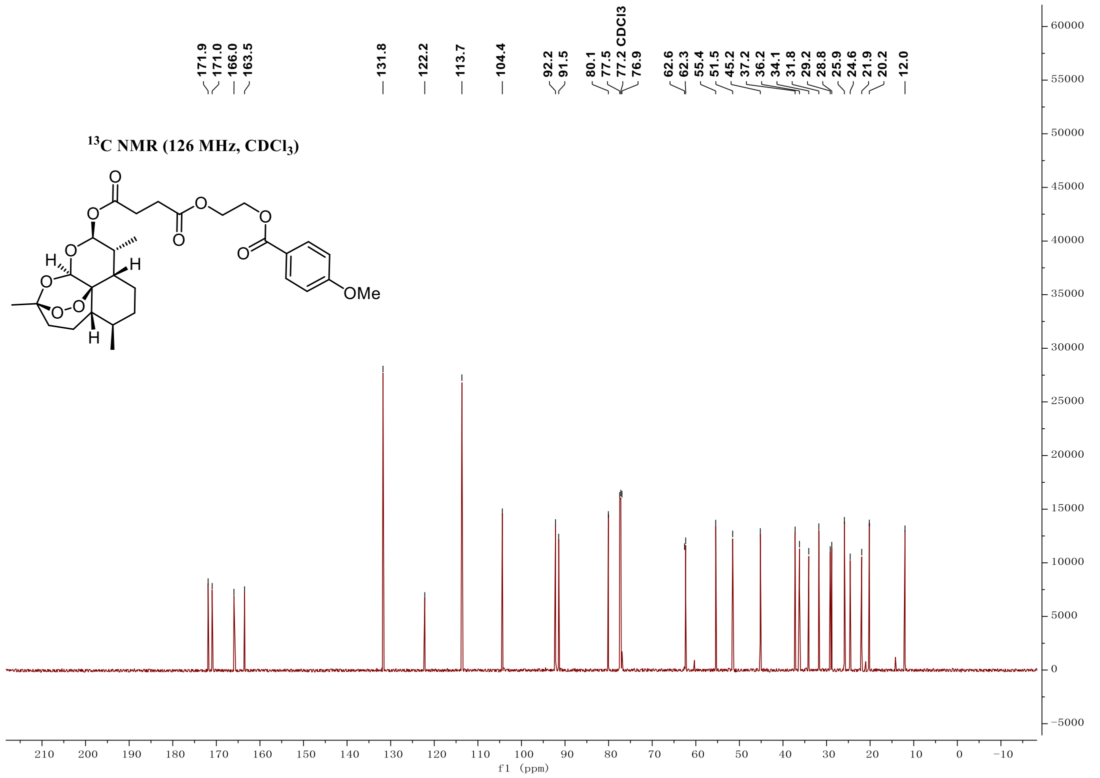

Supplementary Fig. 209. ^{13}C NMR spectra of compound **S7** (126 MHz, rt, $CDCl_3$).

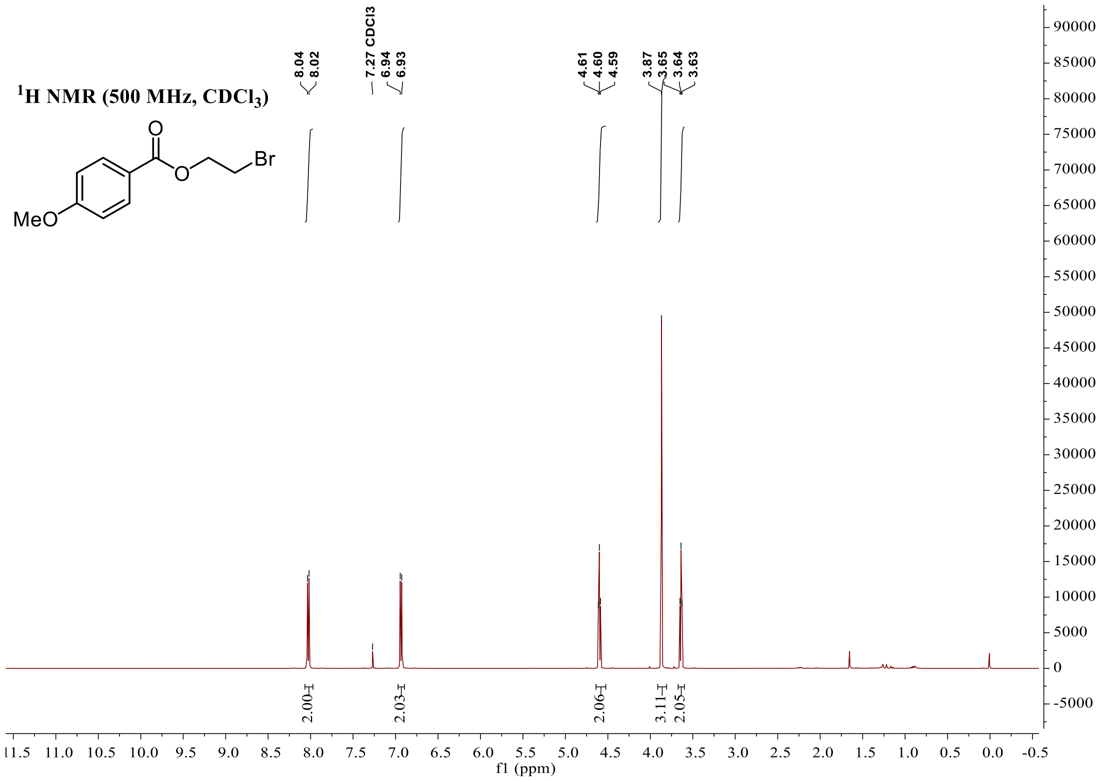

Supplementary Fig. 210. ^{1}H NMR spectra of compound **S8** (500 MHz, rt, $CDCl_3$).

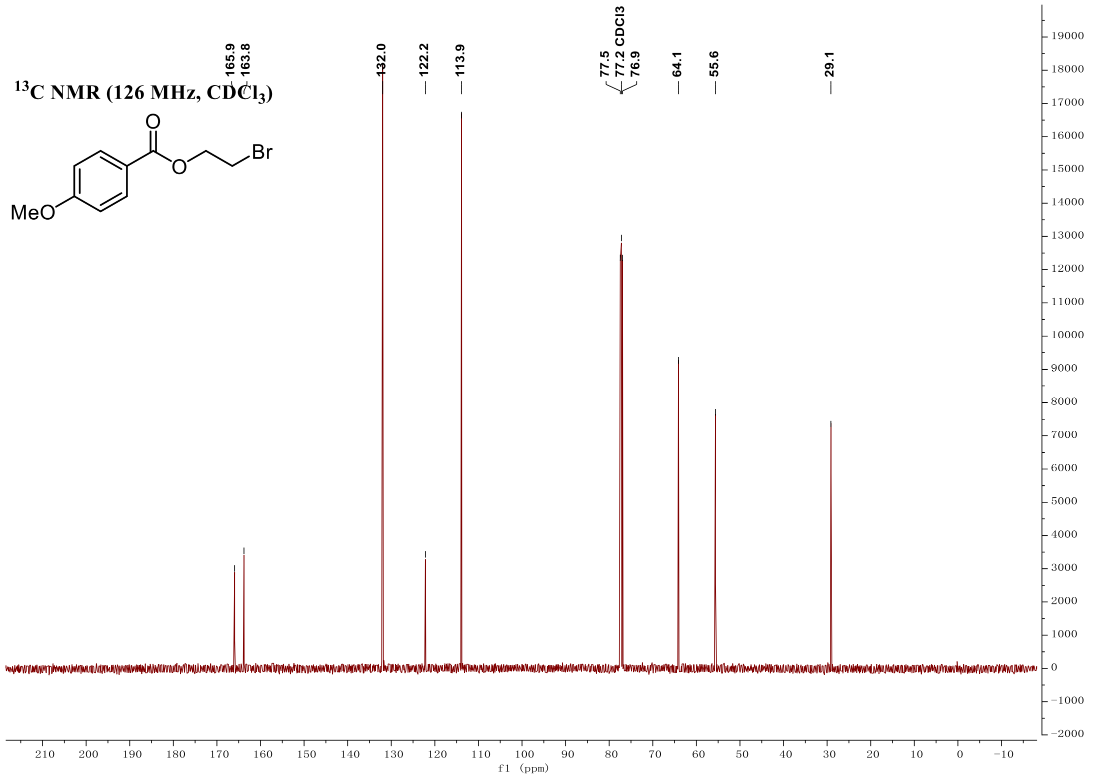

Supplementary Fig. 211. ^{13}C NMR spectra of compound **S8** (126 MHz, rt, $CDCl_3$).

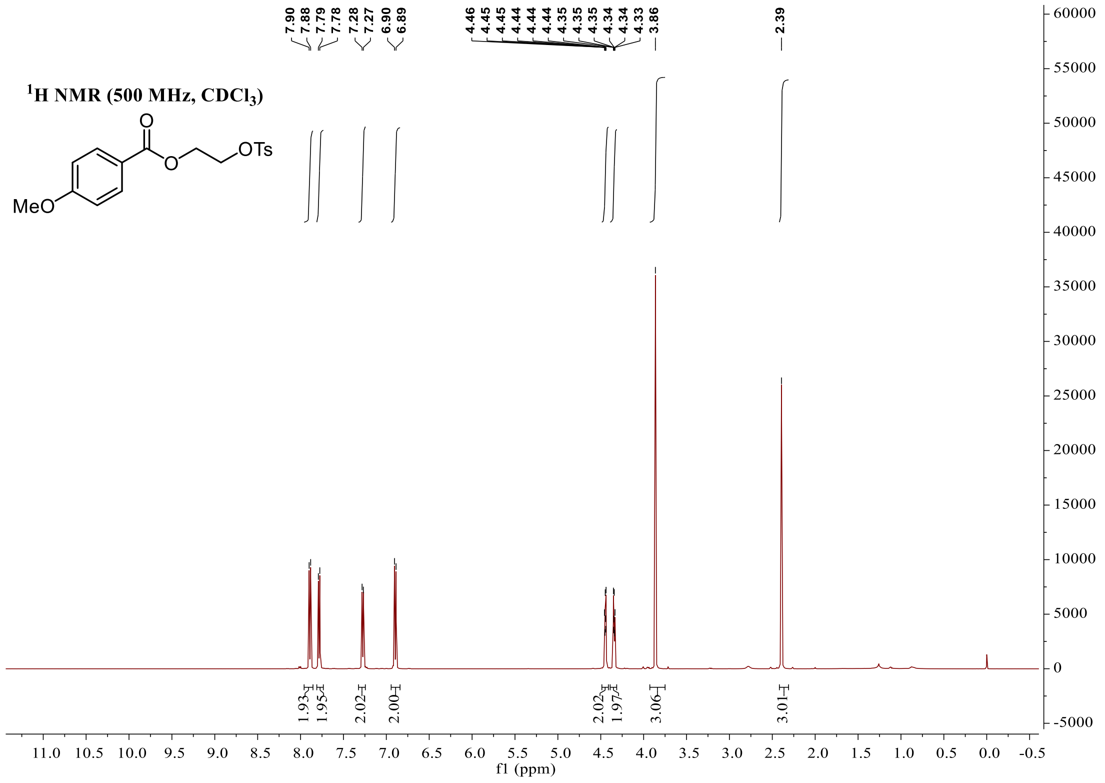

Supplementary Fig. 212. ^{1}H NMR spectra of compound **S9** (500 MHz, rt, $CDCl_3$).

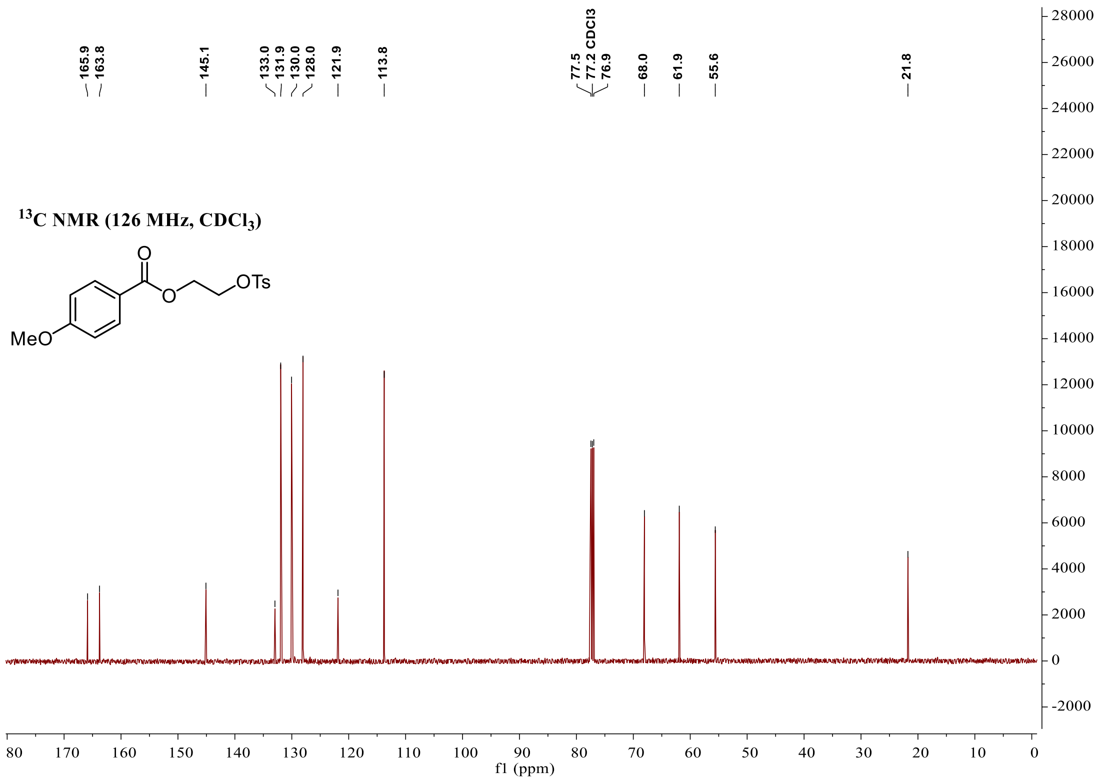

Supplementary Fig. 213. ^{13}C NMR spectra of compound **S9** (126 MHz, rt, $CDCl_3$).

Supplementary References

[1] K. Audouze, E. O. Nielsen, D. Peters, *J. Med. Chem.* **2004**, *47*, 3089-3104.

[2] A. Carbone, C. L. Lucas, C. J. Moody, *J. Org. Chem.* **2012**, *77*, 9179-9189.

[3] P. O. Steen, M. Grandbois, K. McNeill, W. A. Arnold, *Environ. Sci. Technol.* **2009**, *43*, 4405-4411.

[4] M. Van der Mey, A. Hatzelmann, G. P. M. Van Klink, I. J. Van der Laan, G. J. Sterk, U. Thibaut, W. R. Ulrich, H. Timmerman, *J. Med. Chem.* **2001**, *44*, 2523-2535.

[5] Y. Y. Shan, J. Lei, L. Zhang, T. Fan, M. Y. Wang, Y. Ma, *Chem. Nat. Compd.* **2015**, *51*, 620-625.

[6] X. L. Wang, T. T. Ju, X. D. Li, X. P. Cao, *Synlett* **2010**, 2947-2949.

[7] H. Dousova, Z. Ruzickova, P. Simunek, *J. Heterocycl. Chem.* **2018**, *55*, 670-684.

[8] K. C. Nicolaou, D. Das, Y. Lu, S. Rout, E. N. Pitsinos, J. Lyssikatos, A. Schammel, J. Sandoval, M. Hammond, M. Aujay, J. Gavrilyuk, *J. Am. Chem. Soc.* **2020**, *142*, 2549-2561.

[9] R. Boobalan, K. K. Liu, J. I. Chao, C. P. Chen, *Bioorg. Med. Chem. Lett.* **2017**, *27*, 1784-1788.

[10] R. C. Sang, S. E. Korkis, W. Q. Su, F. Ye, P. S. Engl, F. Berger, T. Ritter, *Angew. Chem. Int. Ed.* **2019**, *58*, 16161-16166.

[11] aJ. L. Boyer, J. E. Krum, M. C. Myers, A. N. Fazal, C. T. Wigal, *J. Org. Chem.* **2000**, *65*, 4712-4714; bJ. Phan, S. M. Ruser, K. Zeitler, J. Rehbein, *Eur. J. Org. Chem.* **2019**, *2019*, 557-561; cW. K. Su, C. Jin, *Synth. Commun.* **2004**, *34*, 4199-4205.

[12] C. M. Ting, Y. L. Hsu, R. S. Liu, *Chem. Commun.* **2012**, *48*, 6577-6579.

[13] A. K. Cook, M. H. Emmert, M. S. Sanford, *Org. Lett.* **2013**, *15*, 5428-5431.

[14] K. A. Nolan, J. R. Doncaster, M. S. Dunstan, K. A. Scott, A. D. Frenkel, D. Siegel, D. Ross, J. Barnes, C. Levy, D. Leys, R. C. Whitehead, I. J. Stratford, R. A. Bryce, *J Med Chem* **2009**, *52*, 7142-7156.

[15] X. Y. Zhan, H. Zhang, Y. Dong, J. Yang, S. He, Z. C. Shi, L. Tang, J. Y. Wang, *J. Org. Chem.* **2020**, *85*, 6578-6592.

[16] A. Telliez, M. Desroses, N. Pommery, O. Briand, A. Farce, G. Laconde, A. Lemoine, P. Depreux, J. P. Henichart, *ChemMedChem* **2007**, *2*, 318-332.

[17] aH. M. Yang, M. L. Liu, J. W. Tu, E. Miura-Stempel, M. G. Campbell, G. J. Chuang, *J. Org. Chem.* **2020**, *85*, 2040-2047; bS. Sivaraman, T. J. Sullivan, F. Johnson, P. Novichenok, G. L. Cui, C. Simmerling, P. J. Tonge, *J. Med. Chem.* **2004**, *47*, 509-518.

[18] aX. Li, W. Song, W. Tang, *J Am Chem Soc* **2013**, *135*, 16797-16800; bA. M. A. G. Oliveira, M. M. M. Raposo, A. M. F. Oliveira-Campos, J. Griffiths, A. E. H. Machado, *Helv. Chim. Acta.* **2003**, *86*, 2900-2907.

[19] aR. Sang, S. E. Korkis, W. Su, F. Ye, P. S. Engl, F. Berger, T. Ritter, *Angew. Chem. Int . Ed.* **2019**, *58*, 16161-16166; bX. Ma, Y. Liu, L. Du, J. Zhou, I. E. Marko, *Nat. Commun.* **2020**, *11*, 914.

[20] C. Yuan, Y. Liang, T. Hernandez, A. Berriochoa, K. N. Houk, D. Siegel, *Nature* **2013**, *499*, 192-196.

[21] A. Odedra, C. J. Wu, T. B. Pratap, C. W. Huang, Y. F. Ran, R. S. Liu, *J. Am. Chem. Soc.* **2005**, *127*, 3406-3412.

[22] A. M. Eliasen, R. P. Thedford, K. R. Claussen, C. Yuan, D. Siegel, *Org Lett* **2014**, *16*,

3628-3631.

[23] E. A. Neal, A. Y. R. Werling, C. R. Jones, *Chem. Commun.* **2021**, *57*, 1663-1666.

[24] V. Elumalai, J. H. Hansen, *Rsc. Adv.* **2020**, *10*, 40582-40587.

[25] Y. W. Zheng, B. Chen, P. Ye, K. Feng, W. Wang, Q. Y. Meng, L. Z. Wu, C. H. Tung, *J Am Chem Soc* **2016**, *138*, 10080-10083.

[26] S. Roy, A. Mukherjee, B. Paul, O. Rahaman, S. Roy, G. Maithri, B. Ramya, S. Pal, D. Ganguly, A. Talukdar, *Eur. J. Med. Chem.* **2017**, *134*, 334-347.

[27] G. B. Cole, G. Keum, J. Liu, G. W. Small, N. Satyamurthy, V. Kepe, J. R. Barrio, *Proc. Natl. Acad. Sci. U. S. A.* **2010**, *107*, 6222-6227.

[28] L. Cheng, H. H. Wang, H. R. Cai, J. Zhang, X. Gong, W. Han, *Science* **2021**, *374*, 77.

[29] G. J. Xiao, W. Q. Li, T. Chen, W. B. Hu, H. Yang, Y. A. Liu, K. Wen, *Eur. J. Org. Chem.* **2021**, *2021*, 3986-3991.

[30] E. Lee, J. K. Kim, M. Lee, *J. Am. Chem. Soc.* **2009**, *131*, 18242.

[31] S. M. Maddox, A. N. Dinh, F. Armenta, J. Um, J. L. Gustafson, *Org. Lett.* **2016**, *18*, 5476-5479.

[32] N. J. Van Zee, V. Dragojlovic, *Org. Lett.* **2009**, *11*, 3190-3193.

[33] W. Y. Yin, X. Z. Pan, W. X. Leng, J. Chen, H. F. He, *Green Chem.* **2019**, *21*, 4614-4618.

[34] Z. Iqbal, A. Lyubimtsev, M. Hanack, *Synlett* **2008**, 2287-2290.

[35] P. M. Leo, C. Morin, C. Philouze, *Org. Lett.* **2002**, *4*, 2711-2714.

[36] C. C. Li, K. Z. Zhang, M. H. Zhang, W. Zhang, W. X. Zhao, *Org. Lett.* **2021**, *23*, 8766-8771.

[37] A. D. Burrows, S. O. Hunter, M. F. Mahon, C. Richardson, *Chem. Commun.* **2013**, *49*, 990-992.

[38] J. Lin, L. Y. Hu, C. Chen, H. J. Feng, Y. Yu, Y. X. Yang, B. Zhou, *Org. Lett.* **2021**, *23*, 1194-1198.

[39] D. Mendel, A. L. Marquart, S. Joseph, P. Waid, Y. K. Yee, A. L. Tebbe, A. M. Ratz, D. K. Herron, T. Goodson, J. J. Masters, J. B. Franciskovich, J. M. Tinsley, M. R. Wiley, L. C. Weir, J. A. Kyle, V. J. Klimkowski, G. F. Smith, R. D. Towner, L. L. Froelich, J. Buben, T. J. Craft, *Bioorg. Med. Chem. Lett.* **2007**, *17*, 4832-4836.

[40] Y. Q. Yang, Y. Lin, Y. Rao, *Org. Lett.* **2012**, *14*, 2874-2877.
